# Genetically Encoded, Noise-Tolerant, Auxin Biosensors in Yeast

**DOI:** 10.1021/acssynbio.4c00186

**Published:** 2024-08-28

**Authors:** Patarasuda Chaisupa, Md Mahbubur Rahman, Sherry B. Hildreth, Saede Moseley, Chauncey Gatling, Matthew R. Bryant, Richard F. Helm, R. Clay Wright

**Affiliations:** † Department of Biological Systems Engineering, Virginia Tech, Blacksburg, Virginia 24061, United States; ‡ Fralin Life Sciences Institute, Virginia Tech, Blacksburg, Virginia 24061, United States; § Department of Biochemistry, Virginia Tech, Blacksburg, Virginia 24061, United States; ∥ The Translational Plant Sciences Center (TPSC), Virginia Tech, Blacksburg, Virginia 24061, United States

**Keywords:** genetically encoded biosensor, indole-3-acetic acid (IAA), auxin, quantification, ratiometric, yeast, dose−response assay

## Abstract

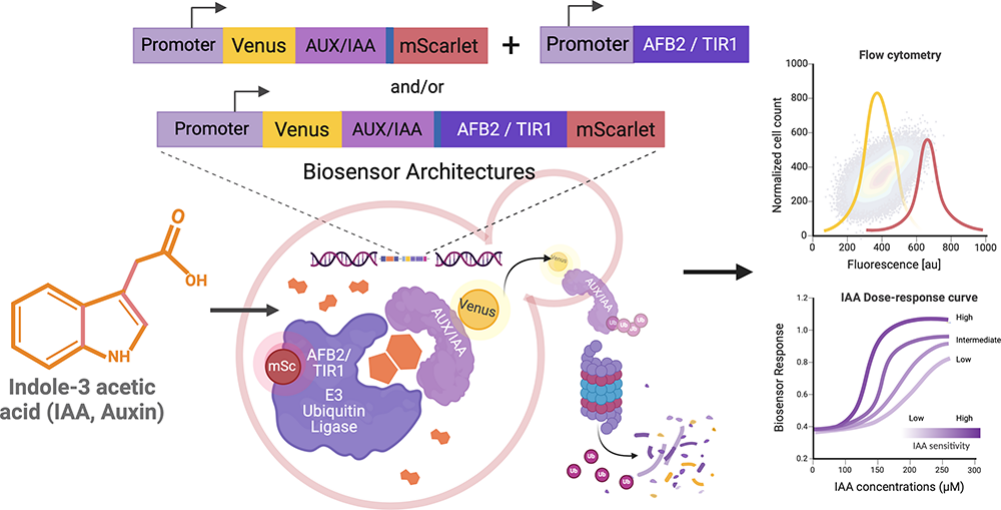

Auxins are crucial signaling molecules that regulate the growth, metabolism, and behavior of various organisms, most notably plants but also bacteria, fungi, and animals. Many microbes synthesize and perceive auxins, primarily indole-3-acetic acid (IAA, referred to as auxin herein), the most prevalent natural auxin, which influences their ability to colonize plants and animals. Understanding auxin biosynthesis and signaling in fungi may allow us to better control interkingdom relationships and microbiomes from agricultural soils to the human gut. Despite this importance, a biological tool for measuring auxin with high spatial and temporal resolution has not been engineered in fungi. In this study, we present a suite of genetically encoded, ratiometric, protein-based auxin biosensors designed for the model yeast *Saccharomyces cerevisiae*. Inspired by auxin signaling in plants, the ratiometric nature of these biosensors enhances the precision of auxin concentration measurements by minimizing clonal and growth phase variation. We used these biosensors to measure auxin production across diverse growth conditions and phases in yeast cultures and calibrated their responses to physiologically relevant levels of auxin. Future work will aim to improve the fold change and reversibility of these biosensors. These genetically encoded auxin biosensors are valuable tools for investigating auxin biosynthesis and signaling in *S. cerevisiae* and potentially other yeast and fungi and will also advance quantitative functional studies of the plant auxin perception machinery, from which they are built.

## Introduction

Auxins are important indole-derived signaling molecules that regulate nearly every aspect of plant growth and development.^1,2^ Auxins also play a role in metabolism and behavior of bacteria, fungi, and animals. Many microbes synthesize and perceive auxin, particularly indole-3-acetic acid (IAA), the most prevalent natural auxin, which may also play a role in the ability of these microbes to colonize plants or animals.^3−5^ Microbially produced auxin is associated with both beneficial and pathogenic microbe–plant interactions, influencing plant growth and microbiome composition and earning the label “a widespread physiological code” in interkingdom interactions.^6^ In *Saccharomyces cerevisiae*, exogenous auxin increases pseudohyphae formation, increasing adhesion and invasive growth.^7,8^ Similarly, auxin increases the virulence of rice blast fungus, *Magnaporthe oryzae*.^9−11^ Conversely, applying exogenous auxin to barley can reduce *Fusarium* head blight severity and yield losses.^12^ The use of auxins and beneficial/biocontrol yeasts, whether or not they produce significant auxin, has proven successful in combating pre- and postharvest pathogens in various fruits, with simultaneous application often yielding synergistic effects.^13−19^ Additionally, microbial production and perception of auxin play crucial roles in plant interactions with rhizobacteria and mycorrhizal fungi.^15−18^ While significant variation in auxin biosynthesis levels exists among strains of *S. cerevisiae*,^19^ the causal mechanism for this variation remains unknown. To gain insights into auxin signaling and biosynthesis in yeasts and fungi, a tool providing high spatial and temporal resolution for measuring intracellular auxin levels is essential.

**Figure 1:**
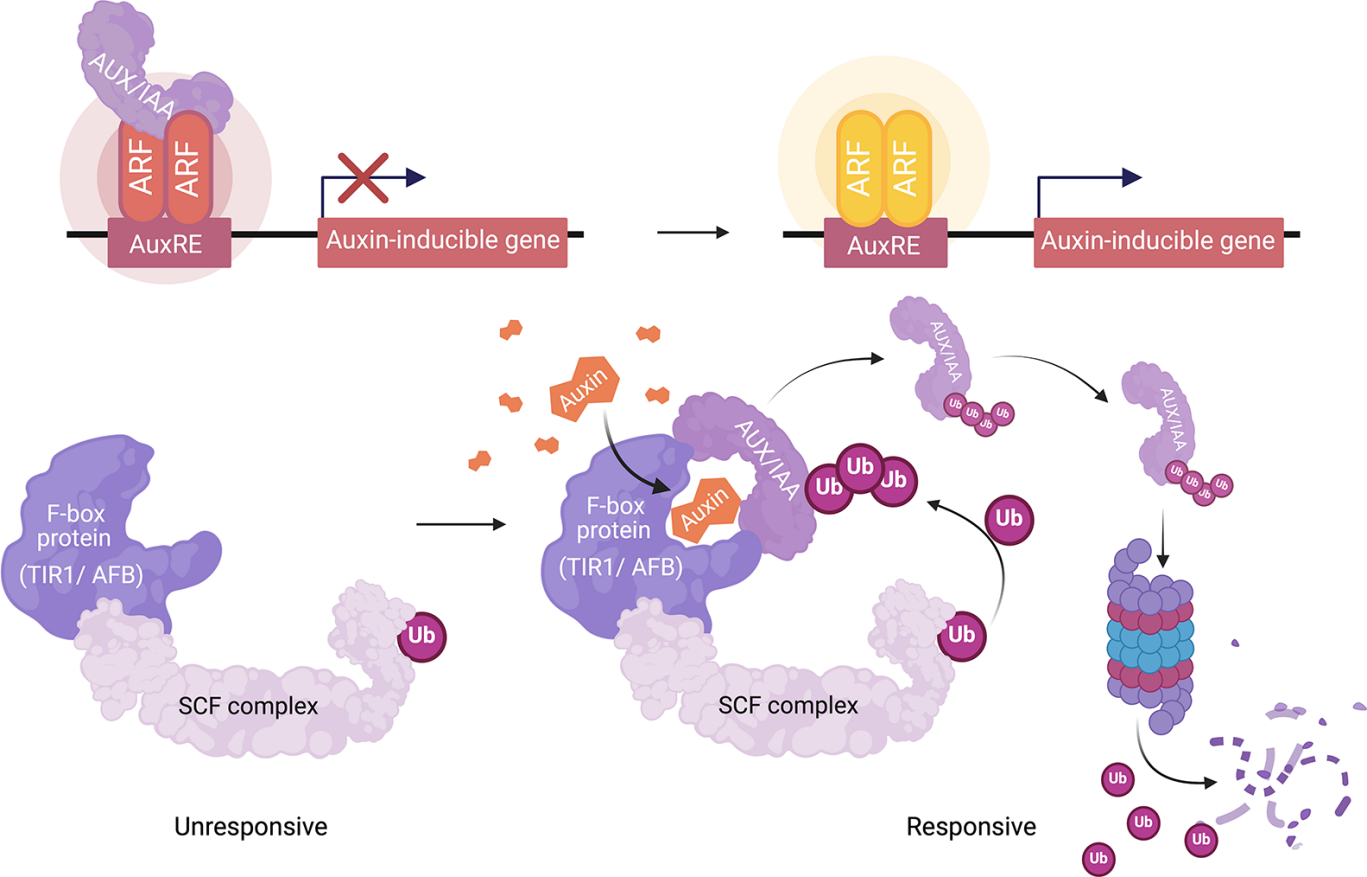
A simple model of the nuclear auxin signaling pathway of plants. Auxin binds to the TIR1/AFB auxin receptors, which are part of Skp1-Cullin-F-box (SCF) ubiquitin ligase complexes and have a weak basal affinity for Aux/IAA proteins that is greatly enhanced in the presence of auxin. This auxin-mediated interaction with the SCF^TIR1/AFB^ ubiquitin ligase complex promotes Aux/IAA ubiquitination and proteasomal degradation. Aux/IAA degradation relieves repression of class A auxin response factors (ARFs), which then activate transcription of auxin-inducible genes containing Auxin-responsive *cis*-regulatory elements (AuxREs).

The amount of auxin in cells or tissues is typically analyzed by conventional analytical methods such as HPLC, GC-MS, LC-MS, or enzyme-linked immunosorbent assays (ELISA).^20−24^ While these methods have high selectivity and sensitivity, they are destructive, invasive, and time-consuming and require laborious sample preparation. In contrast, genetically encoded biosensors (referred to herein as simply biosensors) offer noninvasive, high-throughput, and dynamic measurements. Biosensors respond rapidly and enable the coupling of molecules of interest to rapid, *in vivo*, quantitative output, such as fluorescence or luminescence.^23,25^ Various auxin reporters and biosensors, utilizing plants’ nuclear auxin signaling mechanism, have been developed to study the dynamics of auxin signaling in plants.^26^ The plant nuclear auxin perception complex is composed of the TIR1/AFB auxin receptors, which are part of SCF^TIR1/AFB^ ubiquitin ligase complexes, and the Aux/IAA family of coreceptors. Auxin binds to TIR1/AFBs, recruiting Aux/IAA proteins and promoting the ubiquitination and degradation of Aux/IAAs.^9−11^ Aux/IAA proteins also associate with auxin response factors (ARFs) and repress transcriptional activation. Aux/IAA degradation relieves repression of ARF leading to the activation of auxin-responsive gene expression (Figure 1).^27−29^

Numerous reporters and biosensors for auxin have been developed for their use in plants. Examples of auxin transcriptional reporters include DR5, DR5rev, GH3pro, SAURpro, and pIAAmotif, which rely on the auxin-responsive promoter element (AuxRE) and the native plant auxin signal transduction cascade.^29−31^ Auxin biosensors, such as DII-VENUS, fuse Aux/IAA degron sequences (originally identified as domain II) to a fluorescent protein, such as VENUS.^32^ Therefore, auxin concentration is directly proportional to the rate of DII-VENUS degradation, so a low DII-VENUS signal indicates high auxin concentrations. In plants, DII-VENUS and similar biosensors are dependent on the native TIR1/AFB function and not on transcription and translation of a reporter gene. Their output provides a more immediate and reliable estimate of relative auxin concentrations within a tissue. However, there are many potential confounding factors in using fluorescent protein-Aux/IAA fusion to predict auxin concentrations. The total fluorescence intensity within a cell is proportional to the accumulation of the matured fluorescent protein, which is the sum of the rate of transcription–translation and maturation, the rates of dilution by cell division and expansion, and the basal turnover rate and auxin-induced degradation rate. To control for these confounders of auxin-induced degradation measurements, quantitative ratiometric versions, R2D2 and qDII, have been developed.^33−35^ These ratiometric biosensors use a free fluorescent protein with a separate emission spectrum expressed from the same promoter as that for the fluorescent protein-Aux/IAA fusion. Ideally, the primary difference between the accumulation of the free and Aux/IAA-fused fluorescent proteins is the auxin-dependent degradation of the Aux/IAA fusion. These biosensors along with the commonly used DR5 reporter are sensitive to nanomolar levels of exogenous auxin in plants and report changes in the endogenous auxin and auxin sensitivity. Recently, a Förster resonance energy transfer (FRET) biosensor for auxin, called AuxSen, was engineered from a bacterial tryptophan binding protein.^36^ Through rounds of saturation mutagenesis in the tryptophan binding pocket, AuxSen specifically detects exogenous auxin in plant protoplasts from the micromolar to millimolar range. All in all, the auxin sensors mentioned above have been developed for studying auxin in plants, and not fungi or other microbes, although recently a version of DII-VENUS was implemented in rice blast fungus, *Magnaporthe oryzae*.^37^

**Figure 2:**
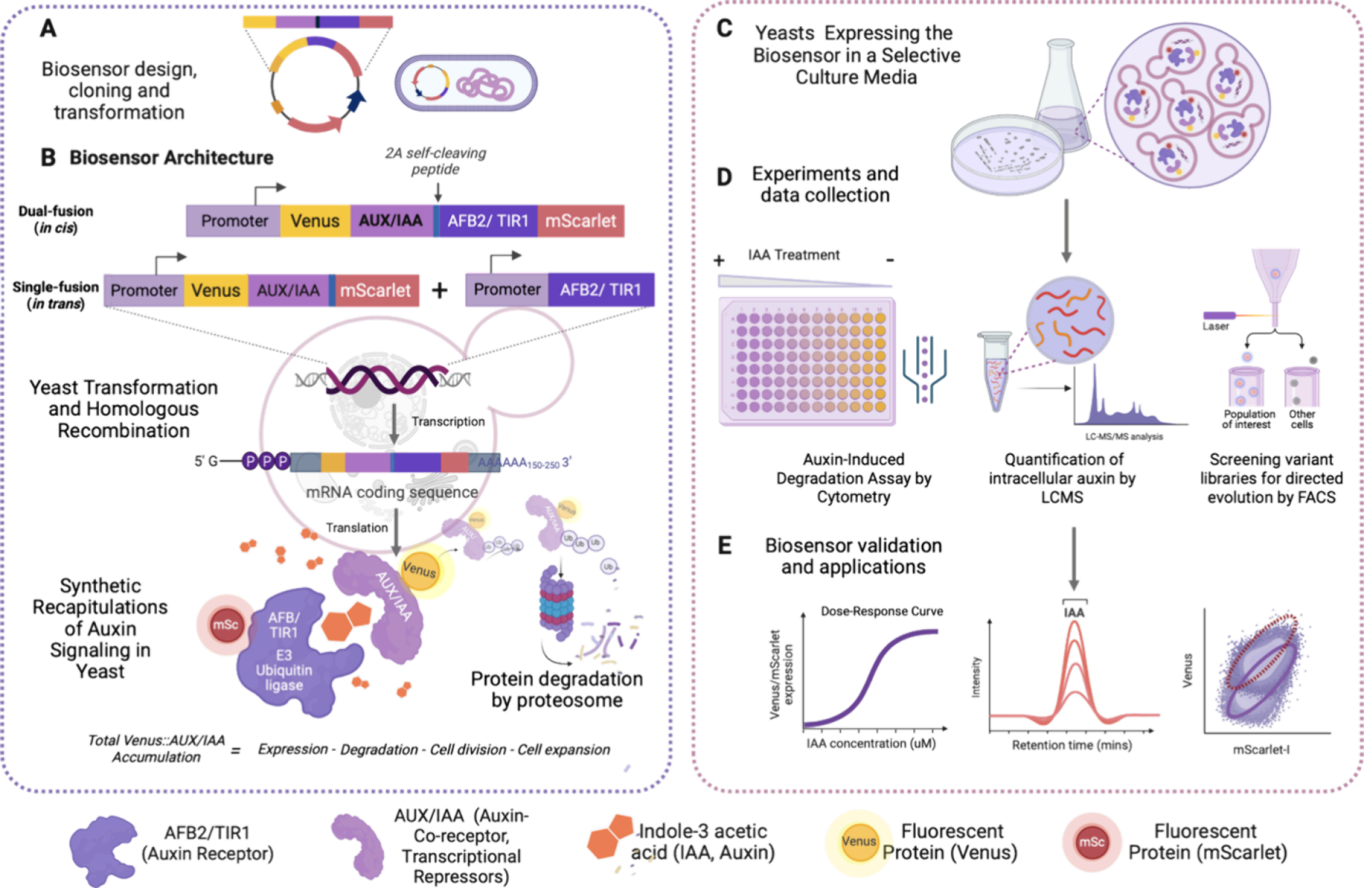
Schematic illustrating the workflow for engineering genetically encoded auxin biosensors. (A) A plasmid construct for the auxin biosensor is generated, chemically transformed, and amplified in *Escherichia coli*, and transformed and integrated into the yeast genome by homologous recombination. (B) The dual-fusion (in cis) ratiometric biosensor construct consists of an auxin receptor unit, TIR1 or AFB2, and a coreceptor Aux/IAA, each fused to a fluorescent protein and separated by a 2A self-cleaving peptide. Single-fusion (in trans) constructs have TIR1 or AFB2 expressed in trans to the ratiometric fluorescent reporter. Expression of these biosensors generates synthetic recapitulations of plant auxin signaling and auxin-induced Aux/IAA fusion protein degradation. The biosensor response is measured by the ratio of TIR1/AFB2-mScarlet-I (or free mScarlet-I) to Venus-Aux/IAA, which is proportional to the auxin concentration at a given time point. (C, D) Positive biosensor-expressing yeast colonies were inoculated into synthetic growth media and incubated overnight for auxin-induced degradation assays via flow cytometry. (E) The capability of the biosensor to detect and quantify auxin was analyzed by comparing the biosensor response (ratio of Aux/IAA-fused to free or TIR1/AFB2-fused fluorescent proteins) to intracellular auxin measurements via LC-MS. The biosensor may also be used to measure functional variation of mutants in TIR1/AFB or Aux/IAA genes with greater precision than prior methods.

In this study, we develop the first well-characterized suite of auxin biosensors in yeast, expanding upon earlier plant auxin ratiometric biosensors.^32−35^ We leverage prior work using recapitulations of plant auxin perception in yeast,^38−40^ to build and characterize a series of whole-cell yeast auxin biosensors (Figure 2). We show that our ratiometric biosensors decrease cell-to-cell and clonal variation due to expression and metabolic-state variation, addressing these confounding factors in auxin- and auxin perception measurements. We demonstrate that these biosensors can measure exogenous auxin from low nanomolar to high micromolar levels, covering the likely range of endogenous auxin production levels by yeast. Finally, we compare the response of our most sensitive version of our biosensor to intracellular measurements of auxin via LC-MS and demonstrate that this biosensor responds to auxin accumulation in yeast cultures at the stationary phase. Limitations of the current iterations of these biosensors include relatively small fold change and lack of useful reversibility. We anticipate that these biosensors and derivatives of them will be useful for measuring the functional effects of variants in the TIR1/AFB and Aux/IAA plant auxin perception machinery from which they are built and for measuring maxima in intracellular auxin accumulation in mutants in auxin biosynthesis, metabolism, and signaling genes in yeast and other fungi.

## Results and Discussion

### Rational Design and Engineering of Bicistronic, Ratiometric Indole-3-acetic Acid Sensor Circuits in *S. cerevisiae*

Our objective was to develop a suite of genetically encoded biosensors in yeast capable of detecting a wide range of auxin concentrations while addressing confounding factors in live cell measurements, specifically clonal noise and cell-to-cell variation. To achieve this, we leveraged previous work on the recapitulation of auxin perception by the plant TIR1/AFB receptors and Aux/IAA coreceptors through heterologous expression in yeast.^38,39^ In the presence of auxin, the TIR1/AFB receptors form a complex with the Aux/IAA coreceptors, initiating the ubiquitination and degradation of the Aux/IAA coreceptors by the proteasome (Figure 1). The degradation rate in response to auxin of fluorescently labeled Aux/IAAs can be measured via time-course flow cytometry in yeast^40^ and is influenced by the expressed Aux/IAA and TIR1/AFB isoforms, as well as auxin concentration.^38,41−45^ The *Arabidopsis thaliana* TIR1 or AFB2 and Aux/IAA17 were chosen for auxin receptors and coreceptor, respectively, in our auxin biosensors, as Aux/IAA17 is rapidly degraded in the presence of AFB2 but much more slowly in the presence of TIR1,^38^ perhaps allowing for the detection of a wide range of auxin concentrations.

**Figure 3:**
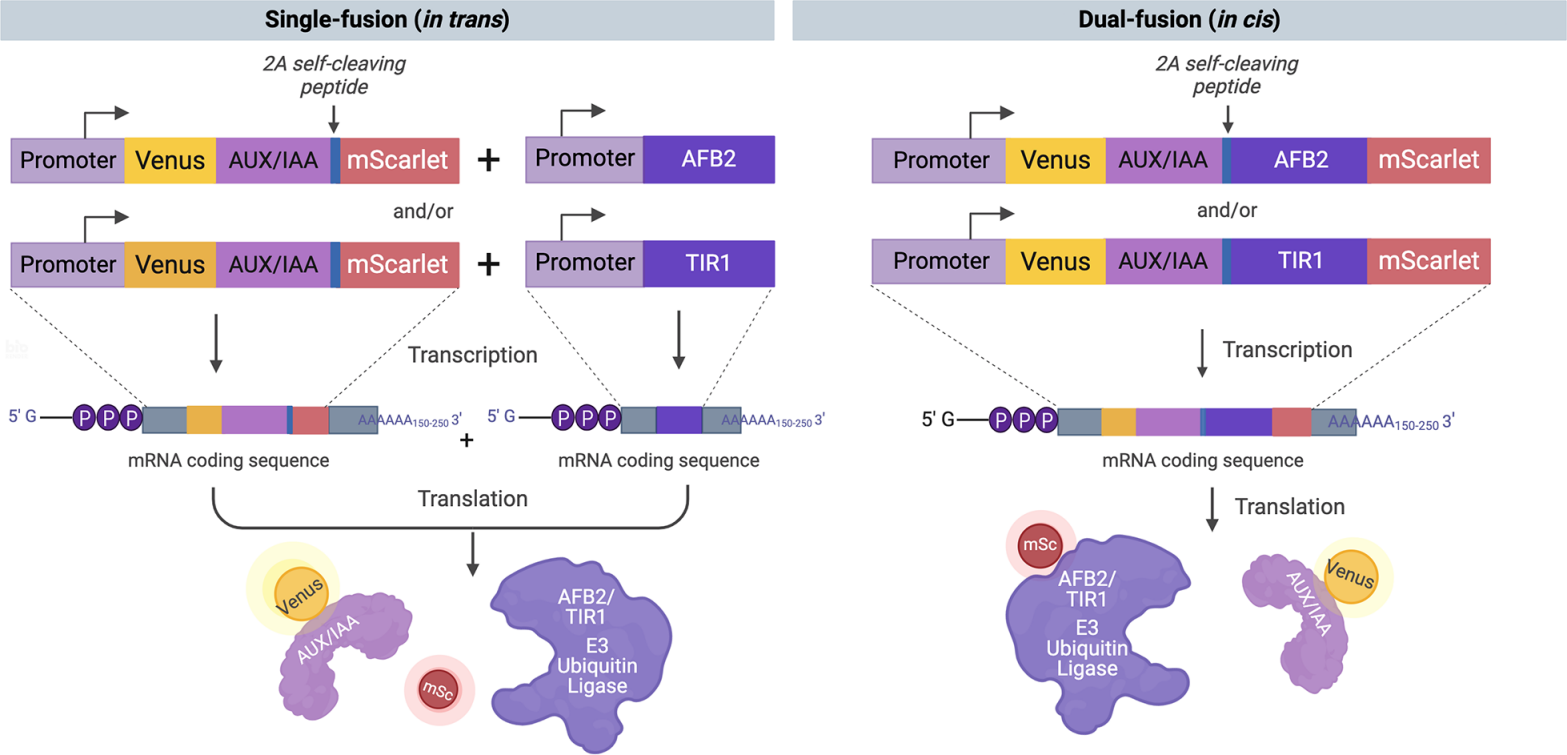
Construct design and protein schematics of the engineered auxin biosensors. The single-fusion (in trans) designs consist of a Venus-Aux/IAA17 fusion and free mScarlet-I expressed from the same cistron. The bicistronic equine rhinitis B virus (ERBV) 2A self-cleaving peptide is inserted into the cassettes. The auxin receptor, TIR or AFB2, is expressed separately from another construct. The dual-fusion (in cis) designs consist of a Venus-Aux/IAA17 fusion and TIR1- or AFB2-mScarlet-I fusion expressed from a single mRNA with ERBV-2A inserted between them, offering fewer steps of genetic manipulation and cell transformation.

We constructed a bicistronic expression cassette utilizing the equine rhinitis B virus (ERBV) 2A self-cleaving peptide^46^ to simultaneously express a Venus-Aux/IAA17 fusion and mScarlet-I under the control of the same promoter, pTDH3, based on previous auxin-induced degradation studies in yeast^38,39^ (Figure 3). The equine rhinitis B virus (ERBV) 2A peptide has previously shown 91% cleavage efficiency in yeast.^46^ We term this design “single-fusion” as we express untagged TIR1 or AFB2 from one expression cassette integrated at the *his3* locus and the Venus-Aux/IAA17-2A-mScarlet ratiometric auxin-responsive degradation reporter from another expression cassette integrated at the *trp1* locus. In a separate design strategy, we also inserted the TIR1 or AFB2 coding sequence between the 2A peptide and mScarlet-I coding sequences, generating a bicistronic expression cassette of Venus-Aux/IAA17 and TIR1/AFB2-mScarlet-I fusion proteins. We term this design “dual-fusion” as we express TIR1/AFB2-mScarlet-I fusions with Venus-Aux/IAA17 (Figure 3). We performed Western blots to confirm that the 2A peptide cleavage efficiency of these dual-fusion expression constructs is similar to previously reported and detected only very little uncleaved product in the TIR1 dual-fusion biosensor strain when loading 500 μg of total protein (Figure S1). The detection of the target analyte, auxin, is measured by the auxin-induced degradation of Venus-Aux/IAA17 relative to the free expression control mScarlet-I for single-fusion designs or the mScarlet-AFB2 receptor control for dual-fusion designs. Auxin-induced protein degradation is also demonstrated via Western blot (Figure S1), although degradation is far from complete. The amount of auxin should be directly proportional to the Venus-Aux/IAA degradation, or inversely proportional to the level of Venus-Aux/IAA detected at a given time point. Therefore, we typically report the response of these biosensors as the ratio of mScarlet-I to Venus fluorescence, which is proportional to auxin concentration, although in some cases, Venus to mScarlet-I will be used to provide comparison to previous auxin-induced degradation systems.

These ratiometric biosensor designs aim to mitigate noise from cell-to-cell variation and growth phase and metabolic status, as we observed previously. In particular, as culture growth rates decrease approaching the stationary phase, expression decreases from the strong pTDH3 promoter,^47^ which we used to drive the expression of the biosensor. With these ratiometric biosensors, changes in the intracellular accumulation of Venus-Aux/IAA17 due to transcription/translation rate, cell division, and cell expansion can be accounted for using the bicistronic mScarlet-I internal controls. Therefore, the ratio of Venus-Aux/IAA17 and TIR1/AFB2-mScarlet-I fusions should more accurately reflect auxin-induced degradation of Venus-Aux/IAA17. Although we expect there to be similar variation in expression with the two fusions integrated into the genome at one or two loci,^48^ we decided to engineer the fusions as a single cistron, again using the 2A peptide, to potentially decrease variation if this biosensor is moved to a plasmid. However, for all works herein, the biosensor was integrated into the genome. The dual-fusion biosensor design also has the advantage of being a single ratiometric expression cassette offering fewer steps of genetic manipulation and cell transformation. Additionally, the dual-fusion biosensor allows direct measurement of both protein components of TIR1/AFB2-auxin-Aux/IAA17 coreceptor complexes, which should provide more accurate measurements of expression and functional variation between coreceptor complex sequence variants.

### Ratiometric Biosensors Reduce Cell-to-Cell, Clonal, and Growth Phase Variation

**Figure 4:**
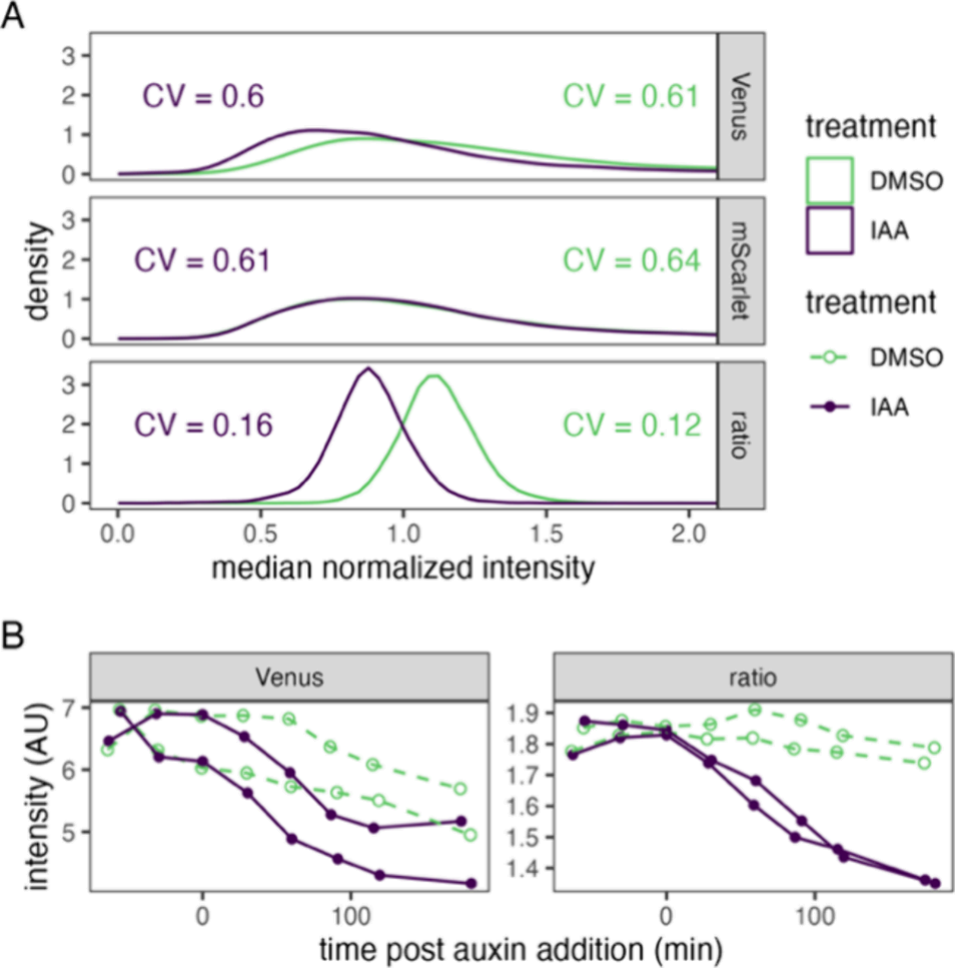
Ratiometric measurement of auxin-induced protein degradation reduces clonal and growth phase variation relative to single color measurement. (A) Cultures expressing single-fusion TIR1 biosensors were treated with 50 μM indole-3-acetic acid (IAA) or DMSO (vehicle control) for 2 h prior to measurement of fluorescent intensity of single cells by flow cytometry. Values were normalized to the median of each culture and fluorophore or the ratio of raw Venus to mScarlet measurements to center the distributions. Lines represent kernel density estimates. Coefficients of variation (CV) for each population are shown. (B) Cultures of two independent clones of the yeast strain in A were treated as in (A) at time zero. Fluorescence intensity was measured by time-course flow cytometry. Population means are presented for each time point with each clonal culture shown as a separate line.

Cell-to-cell variation can be represented by the coefficient of variation (CV) of the distribution of single-cell fluorescence measurements for sample populations of each culture. For the single-fusion biosensor design, the CVs of individual Venus-Aux/IAA17 and mScarlet-I measurements were approximately fourfold greater than that of the ratio of Venus-Aux/IAA17 to mScarlet-I (Figure 4A and Figure S2). This reduction in cell-to-cell variation improves differentiation of the auxin-treated population from the vehicle control, in turn improving potential measurements of auxin or protein variant function using this ratiometric biosensor. To assess the ability of the single-fusion ratiometric auxin biosensors to reduce clonal variation in signal output, two cultures of independent transformants of the TIR1 versions of these biosensors in *S. cerevisiae* W303-1a were treated with auxin (50 μM IAA) or DMSO vehicle control and fluorescence of these cultures was measured over time by flow cytometry (Figure 4B). The clonal and growth phase variation was also qualitatively reduced by using the ratiometric measurement, which shows very similar behavior, compared to Venus-Aux/IAA17 alone, by which the two cultures show noticeably different dynamics (Figure 4B). This improvement will allow more accurate measurements across growth phases and of mutant yeast strains with altered metabolisms. For the dual-fusion biosensor design, we obtained similar decreases in cell-to-cell variation (Figure S3). Ratiometric measurements of Venus-Aux/IAA17 to TIR1/AFB2-mScarlet-I had CV values ∼3-fold lower than those of individual fluorescence measurements.

### Exploring Differences in Biosensor Designs and Portability across *S. cerevisiae* Host Strains

**Figure 5:**
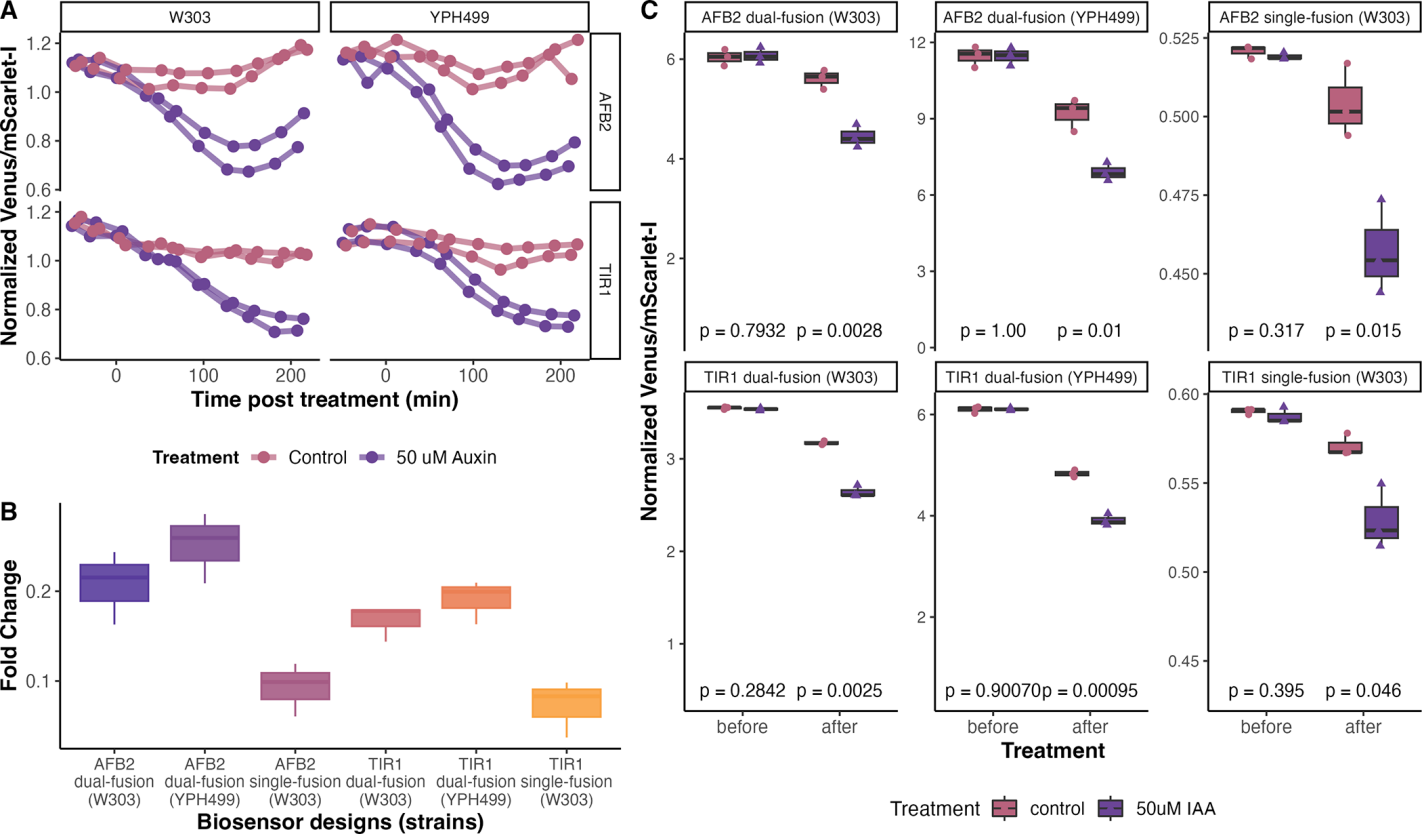
Biosensor designs and portability across yeast strains. (A) Dual-fusion biosensors in two genetically distinct yeast strains, W303 and YPH499, show similar Venus-Aux/IAA17 degradation in response to exogenous 50 μM auxin (depicted in purple) compared to an equivalent dilution of ethanol as a vehicle control (shown in pink). (B) The bar plot illustrates the fold change between the 50 μM auxin treatment and solvent control for each biosensor design. (C) Comparison of biosensors in different yeast strains. Fluorescent measurements of three samples 90 min before the treatments and after 180 min post treatments are shown. Each dot represents the mean fluorescence values measured by flow cytometry. *t* test comparing the response to 50 μM auxin (pink) versus control (purple) for each biosensor design and/or strain are shown.

We further investigated how the sensitivity of the auxin receptor affects biosensor responses by comparing TIR1 and AFB2 in both biosensor designs. Additionally, we examined the portability of these biosensors between two distinct yeast strains of *S. cerevisiae*, YPH499 (which is congenic to S288C)^49−51^ and W303 (which is a hybrid of S288C, ∑1278B, and perhaps other strains).^52,53^ We found that AFB2 induces a more rapid decrease in the Venus/mScarlet-I ratio than TIR1, as expected based on previous observations.^38^ The fluorescence ratios of the cell population shift lower for the strains treated with 50 μM of auxin. However, the fluorescent ratio decreased slightly in the control treatment but much less than in auxin treatment. This decrease in control treatments might be due to *S. cerevisiae* endogenous auxin biosynthesis^19^ (Figure 5A,C). The fluorescence was measured over time every 30 min by flow cytometry for samples of ∼10^4^ cells, and mean fluorescence values were analyzed. Although the dual-fusion biosensors in the two genetically distinct yeast strains had different levels of basal fluorescence, they showed similar behaviors in response to exogenous auxin (Figure S3). This result is supported by normalizing the mean fluorescence values to the overall mean of values on each subplot, which facilitates a relative comparison of dynamics (Figure 5A and Figure S3). The qualitative behavior is similar in these strains, particularly when normalized to the average ratio of the vehicle control. The basal expression of the biosensors varied. Specifically, the Venus/mScarlet-I ratio was nearly twofold higher in YPH499 compared to W303 (Figure S3). We expect that the difference in absolute expression level is due to differences in cell size and shape as well as expression, fluorophore maturation, and basal degradation rates between these strains. It may also be due to variation in metabolic profiles and accumulation of auxin in cultures, which has been previously measured in yeast by the colorimetric Salkowski reagent.^54^ Regardless of the mechanism, calibration of these biosensors is likely required when working with different strains. However, based on the similar dynamic behavior, we expect these biosensors to function similarly in comparative measurements, such as dose–response or mutant analysis. This result suggests the potential for using these biosensors to screen mutant libraries for relative changes in auxin biosynthesis.

Additionally, we compared biosensor design architectures in these yeast strains and confirmed that all biosensor designs developed in this study displayed sensitivity to exogenous auxin (Figure 5B,C). AFB2 biosensors showed greater fold change upon auxin treatment than TIR1, and dual-fusion biosensors showed greater fold change than single fusion, with AFB2 dual-fusion designs having a nearly twofold difference between auxin and control treatment at steady state (Figure 5B and Figure S4). All strains treated with 50 μM auxin showed a significantly lower Venus/mScarlet-I ratio compared to that of control treatments (Figure 5C and Figure S5). The *t* test statistics comparing the response between the two treatments for each strain and design indicated that all biosensor designs developed in this study effectively responded to auxin (Figure 5C). We also examined the reversibility of the dual-fusion AFB2 biosensor, although reversibility has been previously identified as a limitation of auxin-induced protein degradation.^55^ We similarly see that our biosensor is reversible, but several generation times, more than 12 h, are required to recover Venus-Aux/IAA fluorescence levels after washing cells following auxin-induced degradation (Figure S6). However, our experimental conditions of 50 μM auxin treatment is quite stringent for the AFB2 dual-fusion biosensor and not optimized for reversibility. Regardless, this very slow reversibility limits these biosensors to detecting the maximal intracellular auxin concentration over the timespan of the culture. Fortunately, for the intended use cases of measuring the function of TIR1/AFB2 and Aux/IAA mutants in response to exogenous auxin and screening auxin biosynthesis mutants for auxin accumulation, this is the desired measurement.

According to the individual fluorescence expression and accumulation of each biosensor design in the same yeast strain (W303), Venus-Aux/IAA17 degraded significantly over time in response to 50 μM auxin (*p* < 0.05) compared to the control (Figure S4). When comparing the dual-fusion and single-fusion biosensor designs, both Venus-Aux/IAA17 and mScarlet-I fluorescence (free for single-fusion vs TIR1/AFB2-mScarlet-I fusion for dual-fusion) are lower for the dual-fusion biosensors (Figure S4). This could be due to decreased stability of the longer dual-fusion biosensor transcript, as it contains the additional ∼2 kb TIR1 or AFB2 coding sequence as well as other transcriptional and translational burdens that are compounded in this single cistron construct. The lower Venus-Aux/IAA17 fluorescence in the dual-fusion constructs compared to single-fusion supports this hypothesis. However, the dual-fusion design also allows quantification of the relative levels of TIR1- and AFB2-mScarlet-I fusions, potentially providing a proxy for specific molecular functions of TIR1 and AFB2. The AFB2 biosensors also resulted in lower absolute values of Venus-Aux/IAA17 fluorescence after auxin treatment than those after TIR1 for each biosensor design (Figure S3). The lower levels of Venus-IAA17 observed in the presence of AFB2 than TIR1, are likely due increased affinity of AFB2 for Aux/IAA17 in the absence of auxin and associated higher basal turnover rate as well as higher sensitivity of the AFB2-based biosensors for auxin. This high sensitivity of the AFB2 biosensors may allow accurate measurement of perturbations in auxin biosynthesis during yeast growth.

### Ratiometric Auxin Biosensors Can Detect Auxin from Nanomolar to Micromolar Levels

**Figure 6:**
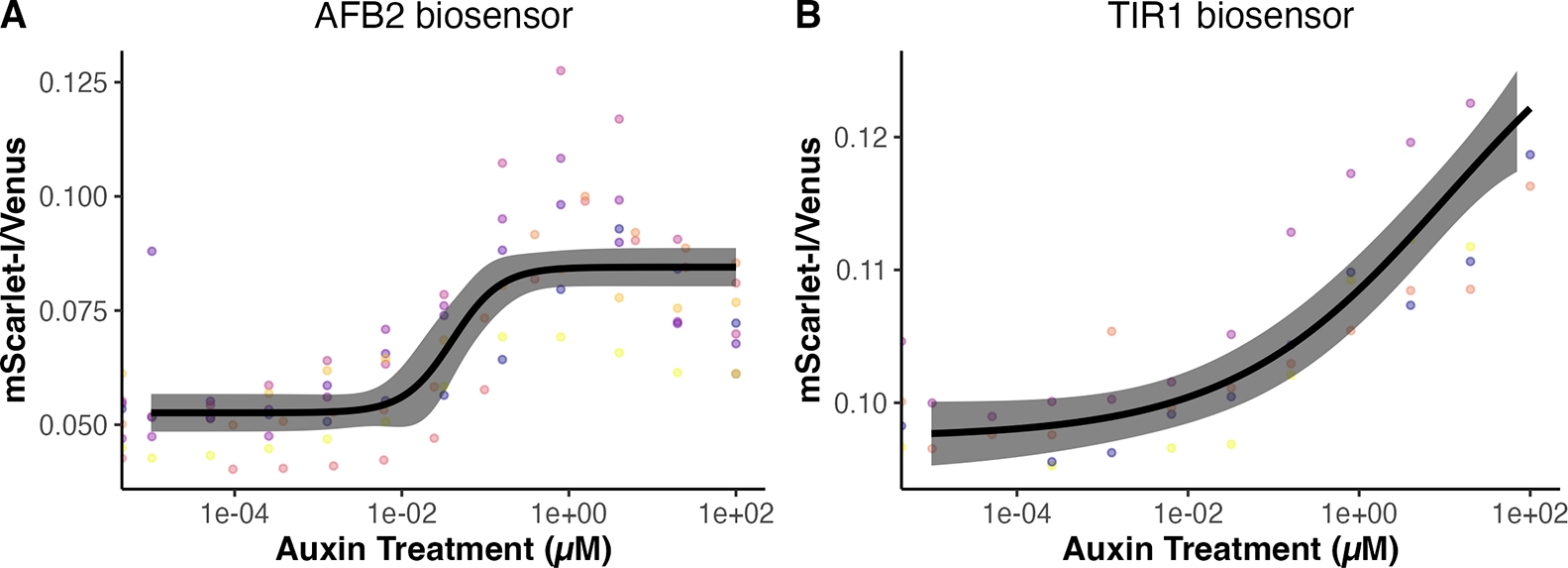
Auxin dose–response curves of dual-fusion ratiometric biosensors. The ratio of mScarlet/Venus-Aux/IAA17 is shown, as it is proportional to auxin. In combination, the TIR1 and AFB2 dual-fusion biosensors respond to auxin concentrations spanning more than five orders of magnitude. Auxin dose–response curves were generated during the exponential phase of cultures expressing the TIR1 and AFB2 dual-fusion biosensors. (A) Auxin dose–response curve for the AFB2 dual-fusion biosensor, based on seven experimental replicates with different colonies on different days (points in different colors). Log–logistic models are shown as black lines, with 95% confidence intervals in gray. The overall EC_50_ for the AFB2 dual-fusion biosensor was 0.040 μM (SE = 0.016). (B) TIR1 dual-fusion biosensor response to various concentrations of extracellular auxin after 3 h (EC_50_ = 10.91 μM, SE = 20.39).

We next examined the ability of these biosensors to detect auxin by measuring the biosensor response to a range of exogenous auxin additions in exponential phase cultures (Figure 6). We envision these biosensors may be useful for screening mutant yeast and perhaps other fungi that differentially accumulate auxin. To examine this, we focus on single-plasmid/construct dual-fusion biosensors to minimize genetic manipulation related to the auxin biosensor. Yeast cultures expressing the dual-fusion TIR1 or AFB2 biosensor in the exponential growth phase were treated with varying concentrations of auxin, and the biosensor responses were measured over time by flow cytometry until steady-state fluorescence and/or stationary phase was reached (Figures S7 and S8). As expected, the AFB2 biosensor is more sensitive, having a lower effective concentration for 50% response (EC_50_) than TIR1 in correspondence with the higher Aux/IAA17 degradation rate in the presence of AFB2^42^ (Figure 6A). The EC_50_ for the AFB2 biosensor is ∼40 nM, whereas for TIR1, it is ∼11 μM, a span of nearly three orders of magnitude—likely covering the physiological range of auxin concentrations in plant cells^20,56^ (Figure 6). The EC_50_ for the TIR1 biosensor is less well-defined as saturating levels of auxin defining the top of the dose–response curve inhibit yeast growth and were not used in these experiments.^57^ The TIR1 biosensor has a much less steep response curve than AFB2 (Figure 6B) and is therefore sensitive to a wider range of concentrations, spanning from ∼100 nM to >100 μM, and perhaps up to the highest physiologically relevant auxin treatment levels, in the low mM range.^34,58^ The steep slope of the AFB2 curve spans concentrations from ∼10 nM to ∼1 μM. It is also possible to build additional biosensors spanning and expanding upon this range of auxin sensitivity for a variety of applications by testing other Aux/IAAs or through mutagenesis of the biosensors described here.

### *In Vivo* Quantification of Auxin and Calibration of the AFB2 Dual-Fusion Ratiometric Biosensor

**Figure 7:**
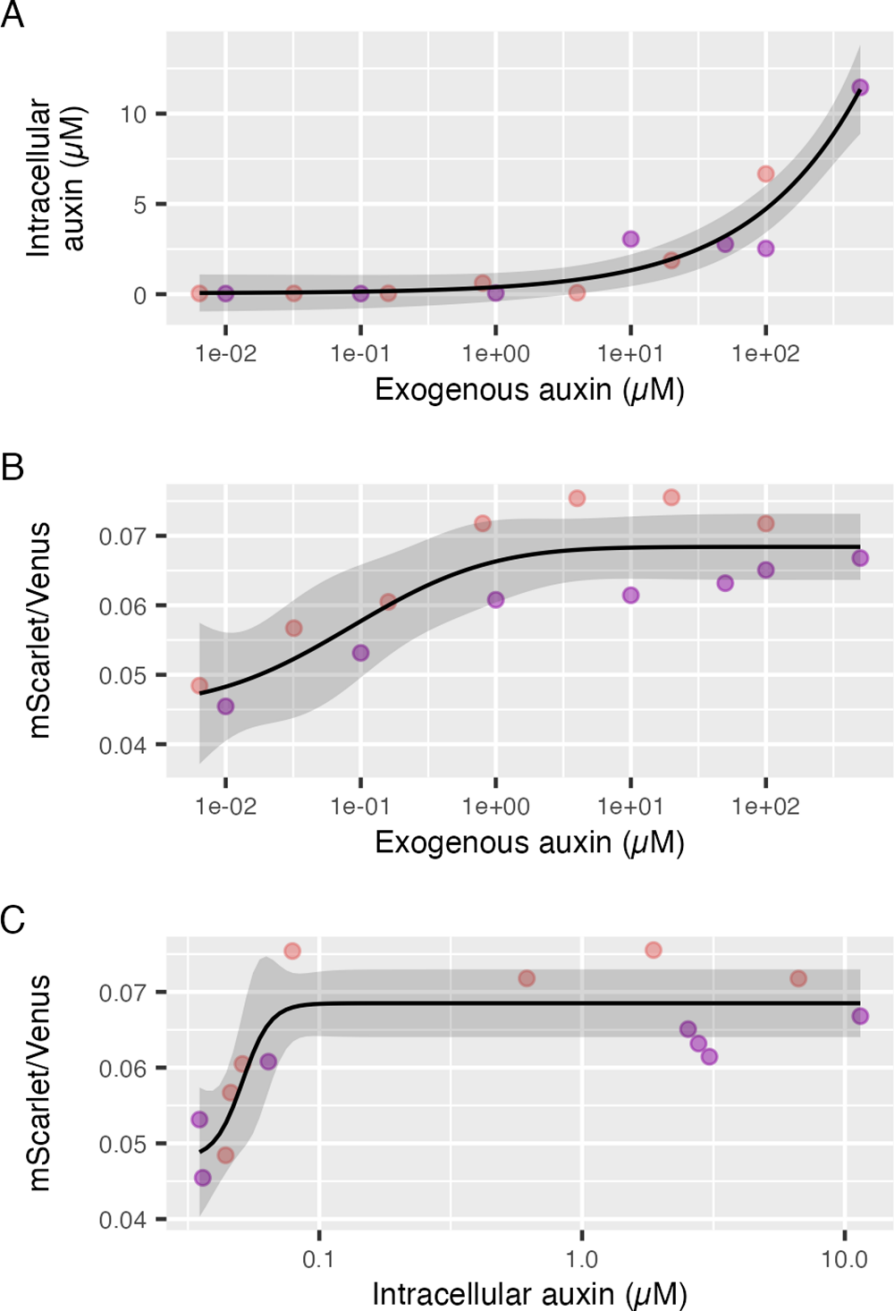
Comparison of LC-MS measurements of intracellular auxin to AFB2 dual-fusion biosensor response across different doses of exogenous auxin. AFB2 dual-fusion biosensor yeast cultures were treated with auxin across four orders of magnitude, and the biosensor response was measured by flow cytometry throughout the exponential growth phase to define steady-state biosensor responses. Following these measurements, cultures were washed and lysed for measurement of intracellular auxin by LC-MS. In each plot, pink and purple points represent two replicate experiments on two different days. Dose–response models are shown as black lines with 95% confidence intervals as gray ribbons. (A) Intracellular auxin as measured by LC-MS versus dose of exogenous auxin after 3 h of treatment. (B) Steady-state biosensor response as the ratio of AFB2-mScarlet-I to Venus-Aux/IAA, which is proportional to auxin concentration, versus dose of exogenous auxin after 3 h of treatment. (C) Biosensor response, as above, versus in intracellular auxin concentrations as determined by LC-MS.

The AFB2 dual-fusion biosensor has proven to be highly sensitive, capable of measuring very fine-grain changes at low levels, around 40 nM exogenous auxin. Its response to increasing doses of auxin were described by a sigmoidal curve (Figure 6A and Figure S5). To verify the biosensor’s reliability in measuring intracellular auxin, we measured intracellular auxin from yeast lysates using LC-MS (liquid chromatography–mass spectrometry), following two auxin dose–response experiments. We employed deuterated IAA as the internal standard and established a standard curve to ensure precise quantification. The limit of quantification (LOQ) was determined to be 10 nM. Through LC-MS, intracellular auxin was successfully detected, and we observed a sigmoidal (logistic) relationship between intracellular auxin and auxin dose, although we did not define the top of the sigmoidal dose–response curve (Figure 7A). Notably, we observed an auxin peak in the control-treated yeast lysate during the LC-MS measurement. This can likely be attributed to endogenous auxin biosynthesis in yeasts.^7^

We further quantified auxin accumulation in yeast at the late-exponential/early stationary phase when cell growth began to decelerate by LC-MS and found that the observed intracellular auxin concentration was 40 nM (SD = 5.6 nM, *N* = 4), nearly equivalent to the EC_50_ of the AFB2 biosensor (Figure 6A). According to our LC-MS data, extracellular auxin does not have a strong effect on intracellular auxin accumulation until at least 1 μM and increases sharply after 50 μM (Figure 7A). The AFB2 dual-fusion biosensor showed the expected response to auxin dose in these experiments with the linear range between roughly 10 nM and 1 μM auxin (Figure 7B). Interestingly, the response range of this biosensor is at least an order of magnitude lower than when we begin to see auxin accumulate via LC-MS (Figure 7A vs B). This may be due to changes in subcellular localization of auxin as the Avt family of auxin permeases are also known to exist on the vacuolar and plasma membranes.^7,59^ Further microscopy and mutagenesis studies with these biosensors could help define the complexities of auxin transport.

The goal of this experiment was to determine the function relating the biosensor response to the concentration of intracellular auxin. However, the mismatch between the linear ranges of the biosensor response to exogenous auxin and LC-MS measurements of intracellular auxin, as well as the 10 nM limit of detection for auxin via LC-MS, constrains the useful range of this experiment. The AFB2 dual-fusion biosensor responds to very small changes in intracellular auxin centered around 40 nM in the characterization above (85 nM in this pair of experiments although due to the limited concentration range the EC_50_ is poorly defined) (Figure 7B). The AFB2 biosensor is essentially saturated at 1 μM exogenous auxin, which is where we begin to see significant accumulation of intracellular auxin via LC-MS. Plotting the biosensor response against the intracellular concentration of auxin as determined by LC-MS, we see a sharp increase in the biosensor response from approximately 30 nM to less than 100 nM intracellular auxin, corresponding to the basal intracellular auxin concentration and the intracellular concentration corresponding to approximately 1 μM exogenous auxin, respectively (Figure 7C). Above 100 nM intracellular auxin, the biosensor response is saturated and unchanged. While this data set provides only limited validation of our biosensors' ability to measure intracellular auxin accumulation, this does suggest that the AFB2 dual-fusion biosensor could be useful in measuring endogenous auxin biosynthesis and transport.

### Auxin Accumulation in the Stationary Phase Is Demonstrated by the AFB2 Dual-Fusion Auxin Biosensor

We and others have previously observed a steady decrease in fluorescence of cells expressing Aux/IAA-fluorescent protein fusions and TIR1/AFB auxin receptors as cultures enter the stationary phase.^60−62^ This could be due to auxin accumulation^8,19^ or changes in the pTDH3 promoter activity used to drive the expression of these proteins as cultures enter the stationary phase. The bicistronic internal control, *mScarlet-I* or *TIR1/AFB2-mScarlet-I*, in our ratiometric auxin biosensors allows us to rule out the alternative hypothesis by controlling for changes in the expression level (Figure 3).

**Figure 8:**
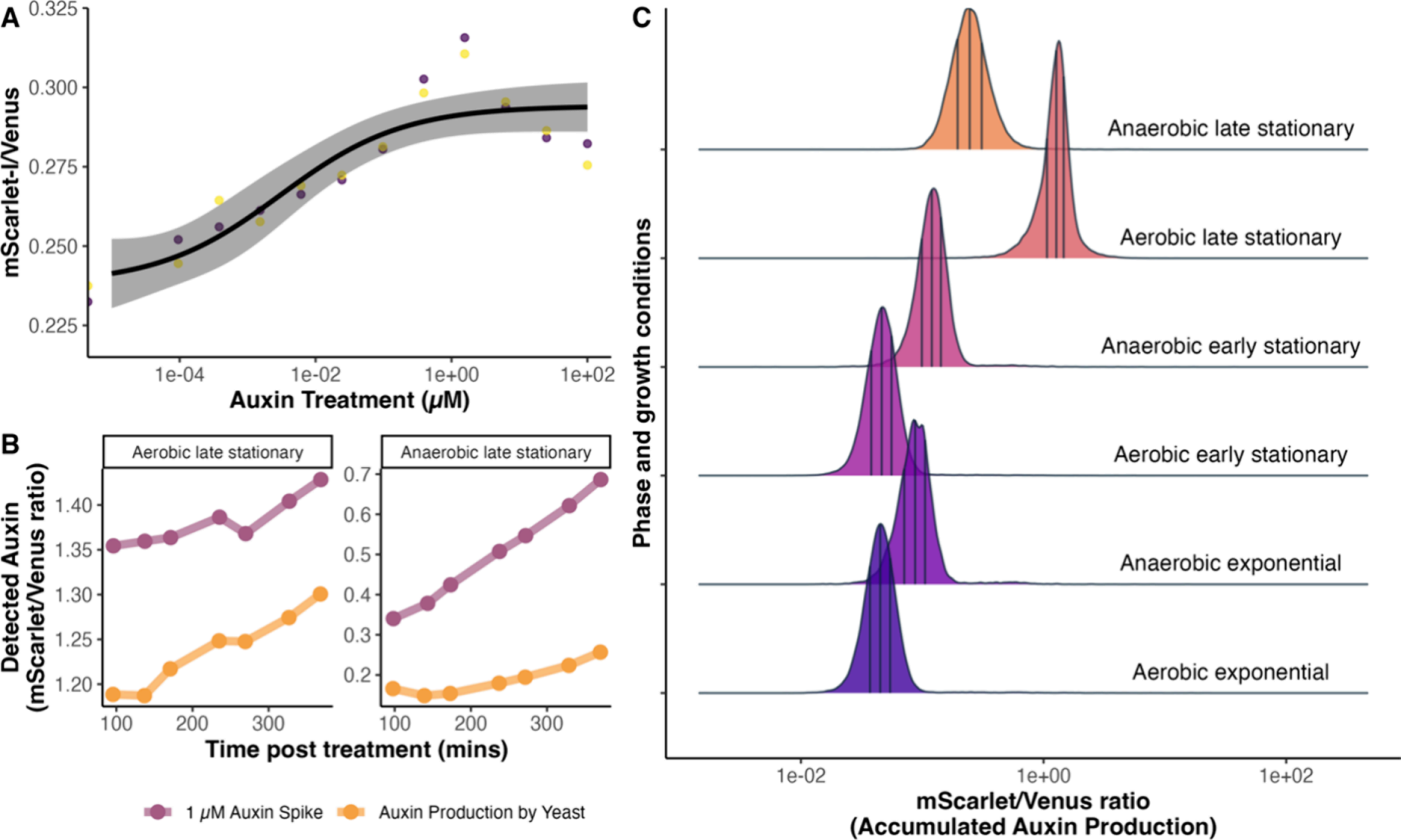
The dual-fusion AFB2 biosensor predicts that auxin accumulates in stationary phase cultures. (A) Dose–response curve for auxin at the early stationary phase. Two yeast colonies carrying the AFB2 dual-fusion biosensor respond to exogenous auxin during this phase with an EC_50_ of 0.003 μM exogenous auxin (SE = 0.00338). (B) Auxin accumulation in the cultures at the late stationary phase (yellow) and a 1 μM auxin spike in the cultures (purple), after 48 h of inoculation, measured by the dual-fusion AFB2 biosensor every 45 min for 6 h. (C) Yeast cultures expressing the AFB2 dual-fusion auxin biosensor were inoculated at varying rates to reach different phases of growth after 16 h of incubation in static fermentative (anaerobic) conditions or shaken (aerobic) conditions. Distributions represent flow cytometric measurements of the ratio of fluorescent intensity areas of AFB2-mScarlet to Venus-Aux/IAA17, which is proportional to the predicted/perceived auxin. Each curve represents the kernel density plot of 10^4^ individual cells. The vertical lines divide the density into quartiles with the middle line representing the median and outer lines representing 25 and 75% of the density.

To test whether our AFB2 dual-fusion biosensor is able to detect this auxin accumulation as cultures enter the stationary phase, as predicted in LC-MS experiments above, we prepared cultures of our biosensor strains starting at typical cell density and continuously cultured from the preceding exponential phase to the stationary phase. Cell density was monitored for 6 h immediately after inoculation or 48 h after inoculation (Figure S9). To confirm that the biosensor is still responsive to auxin in stationary phase cultures, we performed an exogenous auxin dose–response assay with these cultures (Figure 8A). The biosensor responded similarly to exponential phase cultures under these stationary phase conditions when accounting for the auxin accumulated from biosynthesis, increasing the basal response on the low end of the dose–response curve. When we spiked auxin into the cultures at the late stationary phase, the biosensor also responded to this exogenous auxin (Figure 8B). Our AFB2 dual-fusion auxin biosensor predicts that auxin accumulates in stationary phase cultures (Figure 8B,C), in agreement with previous literature.^8,19^ The mean ratio of AFB2-mScarlet-I to Venus-IAA17, which is proportional to the auxin concentration in our dose–response studies, increases from exponential phase to early and then late stationary phase and from aerobic to anaerobic conditions for cultures with the same initial conditions.

## Conclusion and Future Perspectives

We developed a series of ratiometric auxin biosensors in *S. cerevisiae*. To our knowledge, these are the first auxin biosensors in yeast. These biosensors are based on the plant nuclear auxin signaling machinery, which varies widely within and between plant species. These ratiometric biosensors improve upon previous systems for measuring auxin signal transduction capacity of combinations of TIR1/AFB and Aux/IAA family members in yeast^38,39,63−65^ by reducing cell-to-cell and clonal variability, making these biosensors more robust to different growth conditions and genetic backgrounds. Additionally, our dual-fusion biosensor design reports the accumulation of TIR1/AFB auxin receptors, providing mechanistic information. Through calibration of the response of these biosensors to exogenous auxin and LC-MS measurements, as well as biosensor measurements at the stationary phase when yeast cultures accumulate auxin, we have demonstrated that the dual-fusion AFB2 biosensor is capable of reporting auxin biosynthesis and accumulation in yeast. While biosynthetic pathways for auxin in yeast and other fungi are known, these pathways are incomplete and their regulation has only been preliminarily studied.^6−8,66−73^ This successful application of our biosensor in measuring intracellular auxin levels addresses a critical bottleneck in the Engineering Biology Design-Build-Test-Learn cycle, especially in the Test phase. Our biosensors offer numerous advantages over traditional LC-MS screening, including higher-throughput, real-time, and continuous quantification, as well as significantly reducing the consumption of time, reagents, and resources required for the quantification process.

However, the biosensors demonstrated here are only the initial iterations of the Design-Build-Test-Learn cycle for these auxin biosensor designs. Degradation of Venus-Aux/IAA fusions in our current biosensors is far from complete, suggesting that decreasing the overall expression level of these biosensors will increase the fold change in Venus-Aux/IAA fluorescence upon auxin treatment. The optimal production rate of biosensor proteins would allow high levels of Venus-Aux/IAA accumulation and fluorescence in the absence of auxin, while in the presence of auxin allowing nearly complete degradation of Venus-Aux/IAA. In the future, we plan to build modular cloning versions of these biosensors to facilitate rapid optimization using numerous available genetic parts and assembly via liquid handling robotics.^48,74−76^ Rebuilding these biosensors within the constraints of a modular cloning standard will also allow for the construction of combinatorial libraries of TIR1/AFB and Aux/IAA variants. Another likely means of improvement to these biosensors would be to use truncations or only the degron domain of the Aux/IAA proteins as opposed to the full-length protein as in our initial designs. Aux/IAA variants lacking their PB1 oligomerization domains generally have shorter half-lives in the presence of auxin than full-length Aux/IAAs.^63^ To optimize auxin measurement in other yeast and fungi, further optimization of auxin coreceptor proteins, 2A self-cleaving peptides, fluorescent proteins, and codon usage will likely be necessary.

The biosensors we developed here provide new tools to understand how and potentially why fungi synthesize and respond to auxin. Many open research questions could be further studied by utilizing these auxin biosensors, such as how fungi regulate auxin production in response to environmental cues and what molecular and cellular mechanisms underlie these effects as well as how auxin production and perception differ between pathogenic and beneficial microbes. These biosensors will allow exploration of the signaling and physiology of auxin in plant–fungi interactions, as well. Single fluorescent protein reporters based on the same signaling mechanism as those presented here have been used to study auxin’s involvement in *Magnaporthe oryzae* infection of rice.^37^ Auxin-induced protein degradation has been used in many eukaryotic species and will likely function across fungi, with minimal alterations.^77−79^ It may be possible to make single-plasmid constructs that could be used to simultaneously examine a wide variety of yeast species.^80^

We envision and provide preliminary proof of concept for studying auxin biosynthesis in yeast and mutational scanning and directed evolution of auxin coreceptor pairs. Through biosensor-guided engineering, auxin biosynthesis in yeasts could be increased or decreased to examine the role of auxins in different microbiomes. However, our understanding of the metabolic pathways that fungi use to produce auxin is still incomplete. Previous attempts to knock down one biosynthetic route to auxin in yeast resulted in a counterintuitive increase in auxin accumulation revealing complex regulation within the several biosynthesis pathways.^7^ Because the mechanism of this biosensor requires a functional ubiquitin–proteasome system, genetic screens for auxin biosynthesis will likely need to be targeted using CRISPR–Cas mutagenesis or CRIPSR-A/I. However, it may be possible to devise a chemical screening strategy using auxinole, a TIR1/AFB antagonist,^81^ and exogenous auxin to verify biosensor function for more wide-scale genetic screens. Performing mutational scanning of known auxin metabolic enzymes and transporters native to yeast, or heterologously expressed, is certainly feasible with these biosensors. In these cases, a similar dual-fusion construct with a fluorescent protein fused to the gene of interest may be useful. However, it may also lead to unanticipated results, as with the decrease in overall fluorescence of our dual-fusion constructs. While the dual-fusion constructs are capable of reporting auxin concentration, the large increase in TIR1/AFB2-mScarlet fluorescence compared to the slight decrease in Venus-IAA17 demonstrates greatly reduced expression or function of TIR1/AFB2-mScarlet compared to free TIR1/AFB2. Similar mCitrine fusions however complement mutants in *Arabidopsis thaliana* when expressed from native promoters.^82^ Perhaps this observation for dual-fusion biosensors is a yeast-specific phenomenon. The single-fusion biosensor, which is more closely related to previous tools for assessing the function of nuclear auxin signaling pathway components in yeast,^38−40^ may be more useful in assessing auxin coreceptor function, both due to its more canonical behavior and the ability to separately modify *TIR1/AFB* and *Aux/IAA.* Follow-up experiments with similar modifications in dual-fusion biosensors can then be used to parse the effects of TIR1/AFB accumulation versus Aux/IAA degradation.

Our biosensors were developed from a synthetic recapitulation of plant auxin signaling in yeast used to determine the function of TIR1/AFB and Aux/IAA variants.^38,39,63−65^ In the future, we plan to perform comprehensive mutational scanning and directed evolution of these proteins, using the biosensors we developed here, to further refine the sequence function map of these multifaceted signaling proteins^83−88^ and to engineer novel functions.^89^ For mutational studies in *Aux/IAAs*, the single-fusion ratiometric sensor also provides a simple quantitative reporter of Aux/IAA degradation, which would prove useful in studying the numerous functional elements of these transcriptional regulators.^90−96^ As we have observed here through comparisons of TIR1 and AFB2 auxin dose and time responses, pairing of these single- and dual-fusion biosensors to measure auxin coreceptor ubiquitin ligase function from multiple perspectives can provide mechanistic insight into their function.

From a protein engineering perspective, the three-body problem presented by the formation of the TIR1/AFB-auxin-Aux/IAA complex^28,97^ is a fascinating system to study. Similar chemically activated ubiquitin ligase complexes also allow plants to perceive many other internal and environmental chemical signals^98^ including jasmonates,^99,100^ gibberellins,^101−104^ strigolactones,^105,106^ and karrikins,^107,108^ which spatially and temporally control gene expression and coordinate plant growth, development, and behavior, as well as shape microbial interactions.^109^ It is possible that similar ratiometric biosensors could also be established for these signaling pathways, and some have already been established in plants.^33,35,110^ We hope the biosensors we present here or future iterations will shed additional light on how auxin is produced and perceived by fungi, as well as allow us to re-engineer this interkingdom signaling pathway for applications in agriculture, medicine, and biotechnology.

## Methods

### Plasmid Construction

Primers for cloning DNA fragments for the ratiometric biosensor constructs were designed using the online DIVA J5 DNA assembly design tools (public-diva.jbei.org).^111^ The complete list of primers used in this study can be found in Table S1. The DNA fragments and constructs containing *TIR1* or *AFB2*, *Aux/IAA17* fused to the Venus and mScarlet fluorescent protein coding sequences were amplified and inserted into pGP4G2 and/or pGP8G2 downstream of a GPD promoter.^38,40^ The single-fusion (trans) configuration biosensors consist of a Venus-Aux/IAA fusion and free mScarlet-I expressed from the same cistron. This cassette contains a prototrophic *TRP1* gene, and 500 bp of homology to the auxotrophic trp1 locus was integrated. Separately, *in trans* another cassette, containing either the *TIR1* or *AFB2* auxin receptor and including *HIS3* prototrophy and integration arms to the *his3* locus, was integrated. These two cassettes, expressed on separate plasmids, were sequentially transformed. The final transformants were plated on dual -HIS and -TRP dropout plates. The colonies were streaked to isolation and successful integrations at the *his3* and *trp1* loci were confirmed by diagnostic PCR. On the other hands, the dual-fusion (*in cis*) configuration biosensors consist of a Venus-Aux/IAA and AFB2/TIR1-mScarlet-I fusion expressed a single construct with the prototrophic *TRP1* gene and 500 bp of homology to the auxotrophic *trp1* locus upstream of the cassette for genomic integration. The PCRs were performed with Q5 High-Fidelity 2X Master Mix (New England Biolabs) with the designed synthetic primers (Sigma-Aldrich). The PCR products of each synthetic part were purified, assembled, and inserted into the vectors via Gibson assembly. The mixture of DNA fragments was purified by Zymo DNA Clean & Concentrate 5 kits before transforming into NEB 10-beta competent *E. coli* cells via chemical transformation and plated onto the selective LB agar plates containing 100 μg/mL of ampicillin (Fisher Scientific). The list of plasmids and *E. coli* strains can be found in Table S2. Negative control and positive control pUC19 were included in the transformations. The transformants were grown on ampicillin-selective LB agar plates overnight at 37 °C. Colonies were selected and subjected to colony PCR prior to Sanger sequencing and whole plasmid sequencing.

### Yeast Transformation

The successfully cloned plasmids were digested with *PmeI* restriction enzyme (New England Biolabs) to linearize the plasmid and expose the homology arms for genomic integration prior to transformations into either W303 or YPH499 (MATa) yeast strains. All yeast strains were routinely struck out on a sterile YPAD plate consisting of 20 g/L dextrose (Fisher Scientific), 20 g/L peptone (Fisher Scientific), 20 g/L yeast extract (Fisher Scientific), 20 g/L agar (Fisher Scientific), and 40 mg/L adenine hemisulfate (Fisher Scientific). The procedure for making competent yeast cells and *n* were performed according to Gietz and Schiestl.^112^ The yeast transformants with an auxotrophic marker were expected to grow at 30 °C on selective synthetic media plates. Yeast transformants were confirmed for correct genomic integration by isolation on appropriate selective plates at 30 °C in addition to yeast colony PCR. Yeast colony PCR was performed using Zymolyase Yeast lytic enzyme (Zymo Research) to lyse the cells. Briefly, three units of zymolyase were mixed with a barely turbid solution of cells in a 15 μL volume. In a thermocycler, the reaction was incubated for 30 min at 30 °C followed by 10 min at 95 °C. 85 μL of water was added to each lysate, and 2 μL of this was used as template for a 20 μL Taq PCR containing primers to amplify from the terminator of the transgene expression cassette across the site of homologous recombination and ∼200 bases into genomic DNA. The presence of appropriate fluorescent protein expression was confirmed using the iBright FL1500 Imaging System (Thermo Fisher Scientific) and an Attune NxT B–Y flow cytometer (Thermo Fisher Scientific). Successful yeast transformants were stored in 15% glycerol at −70 °C. The complete list of *S. cerevisiae* strains in this study can be found in Table S3.

### Flow Cytometry Measurements and Data Analysis

#### Auxin-Induced Degradation Time-Course Assays

Each yeast strain carrying the ratiometric biosensor was isolated on fresh YPAD plates. After 2–3 days of incubation at 30 °C, ∼1/4 of a healthy uniform colony was suspended in synthetic complete medium (SCM) made by supplementing -LEU synthetic dropout medium with 50 μg/mL leucine (Takara Bio, USA). The cell concentration of each inoculum was measured by flow cytometry and then diluted to 1 cell/μL in an Erlenmeyer flask. The cultures were grown overnight at 30 °C and 300 rpm. On the following day, at the exponential growth phase, the cell cultures were aliquoted into a 96-deep well plate. An IAA working solution from 50 mM IAA (Fisher Scientific) in 48% ethanol stock solution was freshly prepared and added to the cell cultures to obtain a final concentration of 50 μM IAA. Another set of cultures were treated with the equivalent solvent control. All cultures were cultivated at 30 °C and 300 rpm, and samples were measured for the change in fluorescence via cytometry (Attune NxT B–Y flow cytometer, Thermo Fisher Scientific) every 30 min. All recorded events were annotated and analyzed using the flowTime R package.^40,113^ Data analysis is documented in Text S1. Intra- and interday replications were performed using different yeast colonies.

#### Dose–Response Assays

Biosensor-expressing yeast cultures were prepared by following the protocol above. A stock solution 50 mM IAA in 48% ethanol was prepared and freshly diluted serially to obtain the final concentrations in the cultures ranging from 100 to 0 μM (typically 100, 20, 4, 0.8, 0.16, 0.032, 0.0064, 0.00128, 0.000256, 0.0000512, and 0.00001020 μM). At the exponential growth phase, different concentrations of IAA working solutions, including the solvent control, were added to each culture. The fluorescence ratio of TIR1/AFB2-mScarlet-I to Venus-Aux/IAA17 was measured at steady state ∼4 h after treatment. For the stationary phase dose–response, the yeast was cultured in SCM in an Erlenmeyer flask for 48 h at 30 °C, 300 rpm. 1200 μL of the culture was aliquoted into each well of a 96-deep well plate and incubated at 30 °C, 300 rpm. IAA was added to the aliquoted cultures as described above. The cultures were diluted 50-fold in SCM and gently mixed with a multichannel pipet immediately prior to cytometer measurements to ensure isolated events. The growth of yeast cells and fluorescence signals in response to different doses of IAA were measured over time and plotted to determine when a new steady-state fluorescence was reached. All recorded events were annotated, and the data were analyzed using the flowTime R package^40,113^ and the drc R package^114^ was used to fit four-parameter log–logistic dose–response curves including the median effective concentration (EC_50_).

#### Auxin Biosynthesis Assays

Cultures were prepared following the protocol above and incubated at 30 °C and 300 rpm for aerobic conditions. Fermentative/anaerobic cultures were not shaken. After 48 h of incubation, the cultures were diluted 100-fold in SCM and gently mixed with a multichannel pipet immediately prior to cytometer measurements to ensure isolated events. The cultures were also aliquoted and grown under the same conditions simultaneously to determine the sensing ability of the biosensor at the stationary phase. At the exponential phase (∼24 h), either 1 μM IAA or control solvent was spiked into the cultures. At the stationary phase (∼48 h), the same cultures were spiked again with either measured 1 μM IAA or control solvent. After the spike, the cultures were measured for auxin accumulations every 45 min.

#### Biosensor Reversibility Test

The time-course assay of auxin-induced Venus-Aux/IAA degradation over a 10 h experiment as described above was carried out. Yeast cultures expressing the AFB2 dual-fusion biosensor were treated with either 50 μM auxin or a solvent control. After conducting the auxin-induced Venus-Aux/IAA degradation assay during the exponential phase, 3 mL of yeast cultures was harvested by centrifugation at 3000*g* for 5 min, followed by resuspension in 1 mL of SCM. The washed yeast cultures were then centrifuged at 3000*g* for 2 min and resuspended in 3 mL of fresh SCM. These cultures were each split into two separate cultures for subsequent post-wash treatment with 50 μM auxin of solvent control. This resulted in four total cultures with different “pre-wash/post-wash” treatment combinations of control and auxin. The cultures were subsequently incubated at 30 °C with shaking at 300 rpm. On the following day (∼12 h after the cells were washed), the cultures were measured again over time for the fluorescent signals via flow cytometry. The data was collected and analyzed.

### Quantification of Intracellular Auxin from Yeast by LC-MS

Following biosensor dose–response experiments and another experiment, cells were harvested from cultures at various extracellular concentrations. 7 mL of each culture was collected, centrifuged, washed with 1000 μL sterile water, and resuspended in 200 μL of 50% acetonitrile. The suspension was mixed vigorously and loaded into a 2 mL lysing matrix Y tube (MP Biomedicals). After loading the sample, the tube was placed in a high-speed homogenizer (MP Biomedicals Fastprep-24 Sample Preparation System) to disrupt the cells at 6.5 m/s, 10 s, for 10 cycles. The lysed samples were incubated on ice for 30 s prior to centrifugation at 16,000*g* for 2 min. Complete lysis was confirmed via light microscopy, by lack of intact cells in several fields of view. The lysate was then collected and stored at −20 °C for further analysis. Immediately prior to analysis the samples were centrifuged at 16,000*g* for 5 min, diluted 1:100 with internal standard in 50% acetonitrile and transferred to a LC-MS vial. LC-MS analysis was performed on a Shimadzu Nextera X2 UPLC interfaced with a Shimadzu 8060 triple quadrupole mass spectrometer. A 5 min binary gradient starting at 80% solvent A, water with 0.1% formic acid, and 20% solvent B, methanol with 0.1% formic acid was used for the analysis with a flow rate of 0.4 mL/min. The gradient conditions were isocratic for 1 min, a linear gradient to 90% B at 3.5 min, and returning to initial conditions at 4.1 min. A 5 μL aliquot was injected onto a Shimadzu Nexcol C18 1.8 μm, 50 × 2.1 mm column maintained at 35 °C. Deuterated indole-3-acetic acid (d7-IAA, Cambridge Isotope Laboratories) was used as an internal standard at a concentration of 75 nM in all samples and standards. The mass spectrometer was operated in positive mode, and the MRM transition for IAA quantification was 176 → 130 and the MRM transition for d7-IAA was 183 → 136. The limit of detection was determined to be 5 nM, and all standards and samples were analyzed in triplicate. The peak area of IAA was normalized to the internal standard and quantification was based upon a standard curve prepared with a purchased IAA standard (Sigma-Aldrich, I15148).

#### Yeast Lysis and Protein Extraction

Cultures of yeast expressing the dual-fusion AFB2 and TIR1 biosensors were treated with either 50 μM auxin or a control solvent at the exponential phase. A dose–response assay was performed, and data were collected. 5 mL of each culture was harvested by centrifuging at 3000*g* for 5 min to pellet the cells, washing with 2 mL of sterile water, and centrifuging again at 13,000*g* for 2 min. The pellet was then collected, weighed, and resuspended in Y-PER Yeast Protein Extraction Reagent (Thermo Fisher) in a 1.5 mL centrifuge tube, with a ratio of 50 mg of wet pellet to 125 μL of Y-PER reagent. Each mixture was vortexed at room temperature for 20 min, followed by centrifugation at 14,000*g* for 10 min. The supernatant from each sample was collected and stored at −70 °C for Western blot analysis the following day.

#### SDS-PAGE and Membrane Transfer

A 500 μg portion of extracted protein from each yeast culture was mixed with 3.75 μL of dye and 6.25 μL of molecular-grade water to make a total volume of 15 μL. The tubes of the mixture were then incubated at 70 °C for 10 min and subsequently loaded into a protein gel (Bolt 4–12% Bis-Tris Plus Gel, Thermo Fisher). Electrophoresis was run at 200 V for 30 min in Bolt MOPS SDS running buffer (Thermo Fisher). Power Blotter Select Transfer Stack (Thermo Fisher) membrane and filters were prepared following the manufacturer’s instructions before assembling each layer for a transfer sandwich. Between each layer, air bubbles were removed with a roller. The gel was then transferred to a PVDF membrane at mixed voltage for 7 min via Thermo Fisher Power Blotter.

#### Immunoblotting

The transferred membrane was stained with Ponceau S (Thermo Fisher) for 30 s to confirm the success of the protein transfer. After being stained, the membrane was washed with 1× TBST until it became clear. The membrane was then removed and washed with ultrapure water four times, each for 5 min, before immersing in blocking buffer (Blocker FL Fluorescent Blocking Buffer, Thermo Fisher) for immunoblotting. The transferred membrane was then soaked in blocking buffer mixed with the primary antibody at 1:1000 (eGFP Monoclonal Antibody, Thermo Fisher or GAPDH Alexa Flour 647, Thermo Fisher) overnight at 4 °C with rocking. The following day, the membrane was washed five times, 5 min for each wash, with TBST. The secondary antibody (Goat anti-Mouse HRP conjugate, Bio-Rad) at 1:1000 dilution in blocking buffer was then added and incubated with agitation for 90 min at room temperature. Next, the blotted membrane was washed six times, 5 min for each wash, with TBST. Finally, the membrane was developed with a chemiluminescence reaction for images (Clarity Western ECL substrate, Bio-Rad).

## Supporting Information Available

Western blot analyses using two antibodies; ratiometric dual-fusion auxin biosensors reduce cell-to-cell variation in yeast strains; auxin-induced Venus-Aux/IAA17 degradation response comparison in yeast strains; comparison of steady-state fluorescent expression levels of single- and dual-fusion biosensors in W303 yeast; comparison of steady-state biosensors responses in different yeast strains; testing the reversibility of the dual-fusion AFB2 biosensor; dose and time response of the AFB2 dual-fusion biosensor; defining conditions for AFB2 dual-fusion biosensor dose–response assay; cell growth conditions during auxin biosynthesis assays; list of primers; list of plasmids and *E. coli*; list of *S. cerevisiae* strains; and reproducible analysis for genetically encoded, noise-tolerant, auxin biosensors in yeast (PDF)

## Notes

Code for analyzing all data and creating all plots is available on github (https://github.com/PlantSynBioLab/auxin-biosensor-data) and in Text S1. All source data is provided in the paper and supplemental code via github repository linked above.
