# Supplementary material for: Genetically Encoded, Noise-Tolerant, Auxin Biosensors in Yeast
Source: ACS Synth Biol. 2024 Aug 28;13(9):2804–19. doi: 10.1021/acssynbio.4c00186 (PMC11421217; doi:10.1021/acssynbio.4c00186)
Supplement: Supplementary file 1 — sb4c00186_si_001.pdf [file sb4c00186_si_001.pdf]

Supporting Information

Genetically encoded, noise-tolerant, auxin biosensors in yeast

Authors: Patarasuda Chaisupa[1], Md Mahbubur Rahman[1], Sherry B. Hildreth[2], Saede Moseley[1], Chauncey Gatling[1], Matthew R. Bryant[1], Richard F. Helm[2,3], R. Clay Wright[1,2,4*]

Affiliations:

[1] Department of Biological Systems Engineering, Virginia Tech, Blacksburg, VA 24061, United States,
[2] Fralin Life Sciences Institute, Virginia Tech, Blacksburg, VA 24061, United States,
[3] Department of Biochemistry, Virginia Tech, Blacksburg, VA 24061, United States,
[4] The Translational Plant Sciences Center (TPSC), Virginia Tech, Blacksburg, VA 24061, United States
[*] To whom correspondence should be addressed: wrightrc@vt.edu

SUPPORTING FIGURES

Supporting Figure S1. Western blot analyses using two antibodies

Supporting Figure S2. Ratiometric dual-fusion auxin biosensors reduce cell-to-cell variation in yeast strains

Supporting Figure S3. Auxin-induced Venus-Aux/IAA17 degradation response comparison in yeast strains.

Supporting Figure S4. Comparison of steady-state fluorescent expression levels of single- and dual-fusion biosensors in W303 yeast.

Supporting Figure S5. Comparison of steady-state biosensors responses in different yeast strains.

Supporting Figure S6. Testing the reversibility of the dual-fusion AFB2 biosensor.

Supporting Figure S7. Dose and time response of the AFB2 dual-fusion biosensor.

Supporting Figure S8. Defining conditions for AFB2 dual-fusion biosensor dose-response assay.

Supporting Figure S9. Cell growth conditions during auxin biosynthesis assays.

SUPPORTING TABLES

Supporting Table S1. List of primers

Supporting Table S2. List of plasmids and *E.coli*

Supporting Table S3. List of *S. cerevisiae* strains

SUPPORTING TEXT

Supporting Text S1. Supplemental reproducible analysis for: Genetically encoded, noise-tolerant, auxin biosensors in yeast.

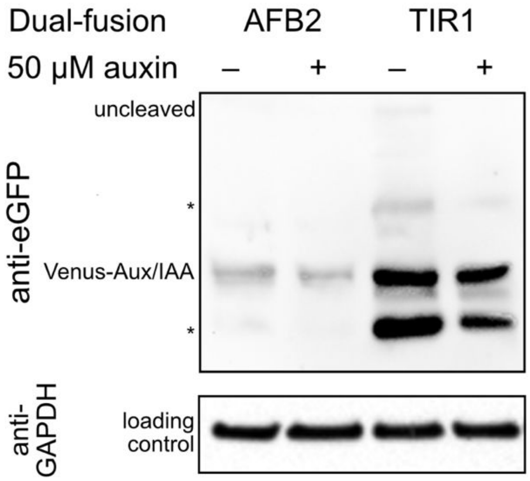

Supporting Figure S1. Western blot analyses using two antibodies. *Cultures of yeast expressing the dual-fusion AFB2 and TIR1 biosensor were treated with either 50 µM auxin or a control solvent for 3 hours at exponential growth phase. Cells were harvested from these cultures and lysed. Protein samples of 500 µg were used for western blotting with anti-eGFP antibodies (which bind to Venus as well) and GAPDH (loading control). The expected band size for Venus-Aux/IAA17 is ~52 kDa, while for TIR1- or AFB2-mScarlet, it is ~91 kDa (which should not stain with the anti-eGFP antibody used as mScarlet is derived from dsRed). The uncleaved product would then be ~143 kDa. Some non-specific bands or degradation products (indicated with *) were also seen, particularly in the TIR1 samples. We did not observe these bands when loading lower amounts of total protein, but also did not observe any uncleaved product.*

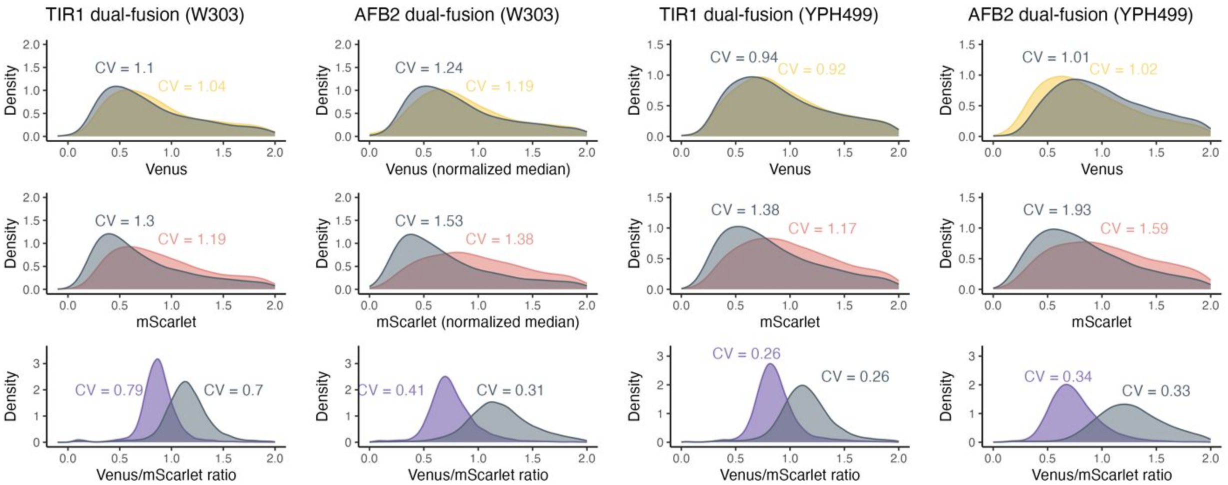

Supporting Figure S2. Ratiometric dual-fusion auxin biosensors reduce cell-to-cell variation in two distinct yeast strains. *Distributions of Venus-Aux/IAA17 (top), TIR1- or AFB2-mScarlet (middle), and the ratio of Venus/mScarlet (bottom) fluorescence in samples of 10^4 cells 4 hours after treatment with 50 µM auxin (colored) or vehicle control (gray). All data were normalized to the median of the combined treated and control distributions on each plot to center the distributions. Distributions are shown as kernel density estimates. The coefficients of variation (CV) of the Venus/mScarlet ratio are reduced about three-fold relative to the single Venus-Aux/IAA17 fluorescence measurements.*

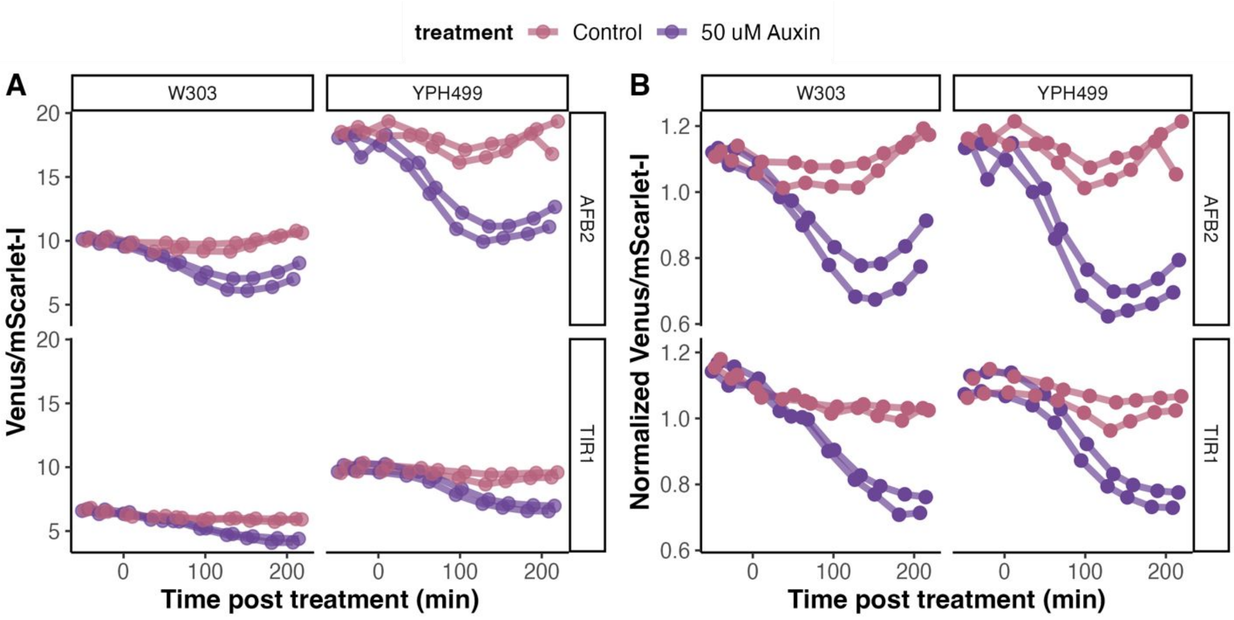

***Supporting Figure S3. Auxin-induced Venus-Aux/IAA17 degradation response comparison in two distinct yeast strains**. YPH499 and W303 yeast with genomically integrated TIR1 or AFB2 dual-fusion biosensor constructs were cultured throughout exponential growth phase and treated with 50 µM auxin or vehicle control treatments at time zero**.** Venus-Aux/IAA17 and TIR1- or AFB2-mScarlet-I fluorescence was measured every 30 minutes by flow cytometry. **(A)** Raw ratios of Venus/mScarlet-I fluorescence show that AFB2 induces a more rapid decrease in the Venus/mScarlet-I ratio than TIR1, as expected based on the more rapid degradation of Aux/IAA17 in the presence of AFB2. Interestingly, basal fluorescence ratio levels of both biosensors were lower in W303 than YPH499. **(B)** To facilitate relative comparison of dynamics the fluorescence ratio values were normalized to the overall mean fluorescence ratio on each subplot. Although the dual-fusion biosensors in the two genetically distinct yeast strains had different levels of basal fluorescence, they showed similar behaviors in response to exogenous auxin.*

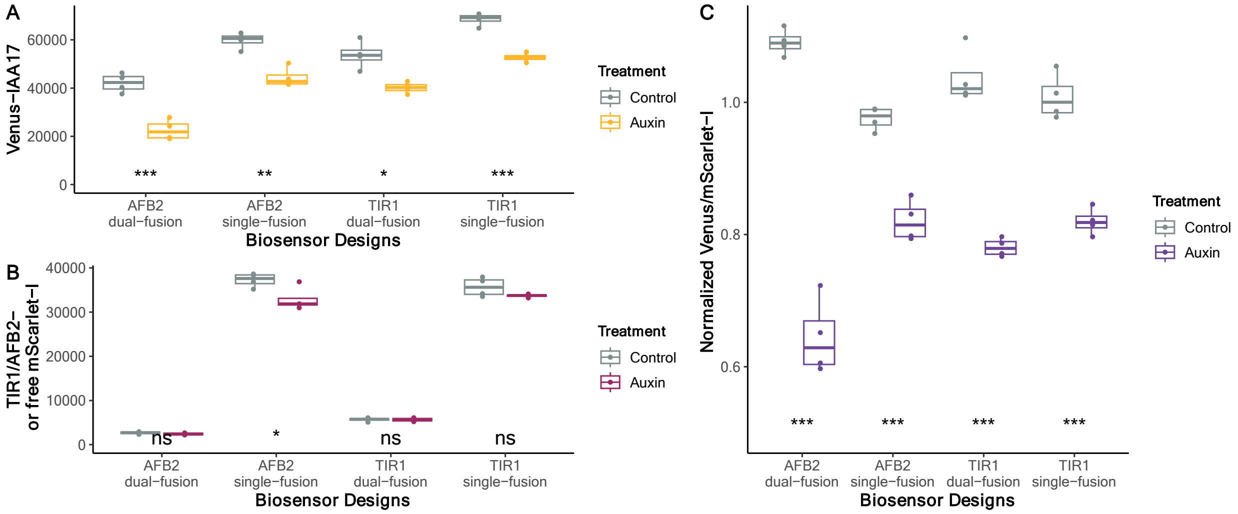

***Supporting Figure S4. Comparison of steady-state fluorescent expression levels of single- and dual-fusion biosensors in W303 yeast.** Comparison of **(A)** Venus-Aux/IAA17, **(B)** TIR1/AFB2-mScarlet-I or free*

mScarlet-I, and ***(C)*** *Normalized Venus/mScarlet-I ratios at steady state (about 2.5 hours after treatment) are shown. Points represent the mean fluorescence of samples of ~10*^{4} *cells from cultures measured every 30 minutes over 120 minutes after the treatments. T-test statistics comparing the response to 50 µM auxin versus control for each biosensor design are shown with "ns" meaning "not significantly different", one star and three stars meaning significantly different with p < 0.05 and p < 0.005, respectively.*

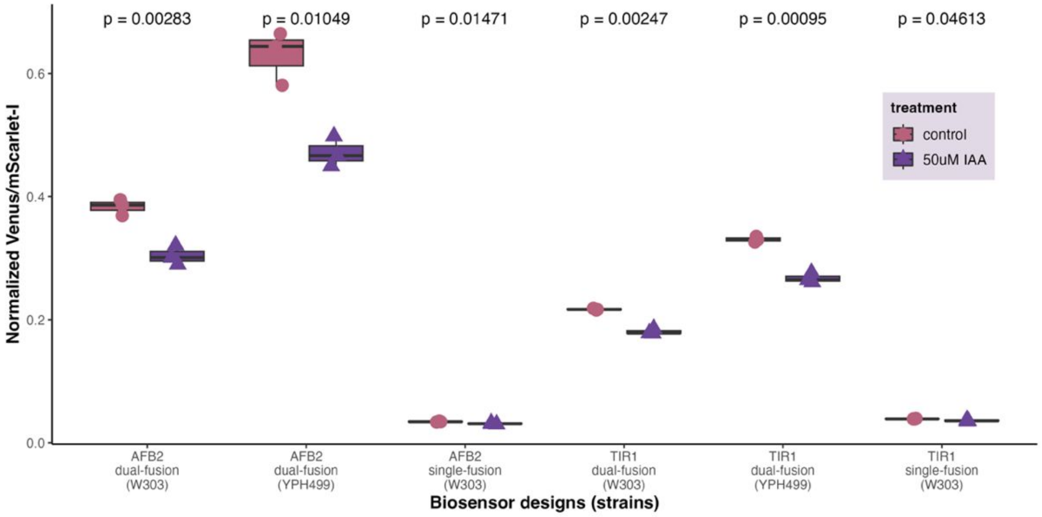

Supporting Figure S5. Comparison of steady-state biosensors responses in different yeast strains. *Fluorescent measurements of three samples at after 180 minutes post treatments are shown. Each dot represents the mean fluorescence values measured by flow cytometry. Some points are overlapping. P-values for T-tests comparing the response to 50 µM auxin (pink) versus control (purple) for each biosensor design and/or strain are shown.*

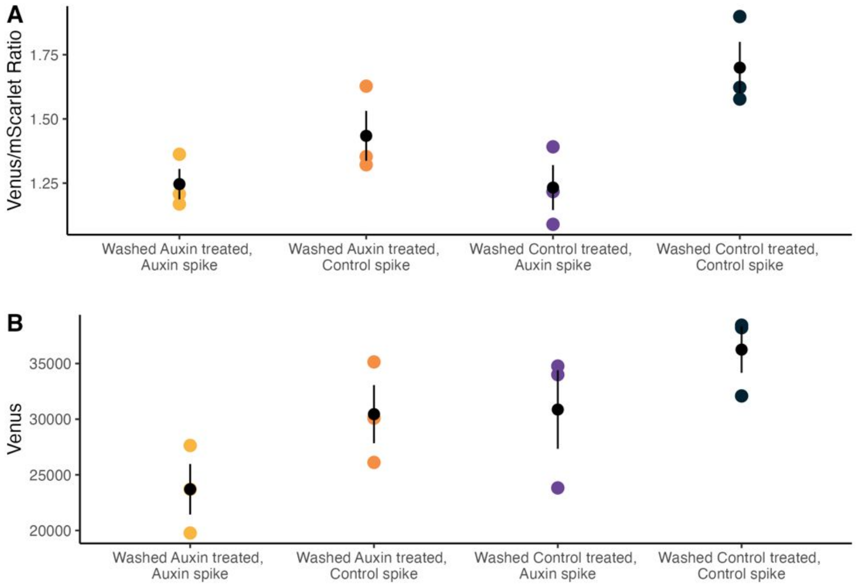

Supporting Figure S6. Testing the reversibility of the dual-fusion AFB2 biosensor. *After the auxin-induced VENUS-Aux/IAA degradation assay at the exponential phase, the cultures were washed to remove auxin and resuspended in 3 mL of fresh SCM. These cultures were split evenly into two tubes to allow them to be treated with control or auxin treatments. Approximately 12 hours after washing, the signal from the previously auxin-treated culture (orange) had recovered about 50% of the difference between the washed auxin-treated cultures (yellow and purple) and the washed control culture (black).*

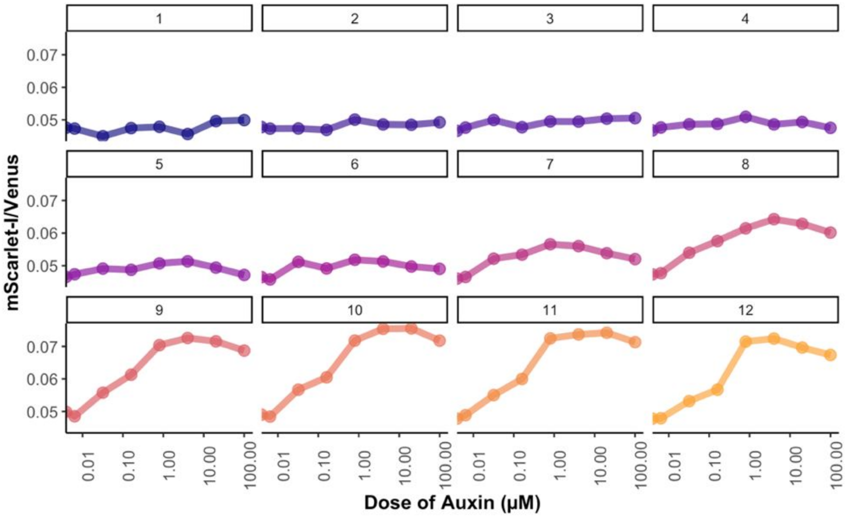

Supporting Figure S7. Dose and time response of the AFB2 dual-fusion biosensor. *Exponential phase cultures of AFB2 dual-fusion biosensor expressing yeast were measured via flow cytometry. Each numbered panel represents a 30-minute time interval. Cultures were treated with different doses of auxin (x-axis) at time-point 5. Each point represents the mean fluorescence of a sample, comprising approximately 10^4 cells.*

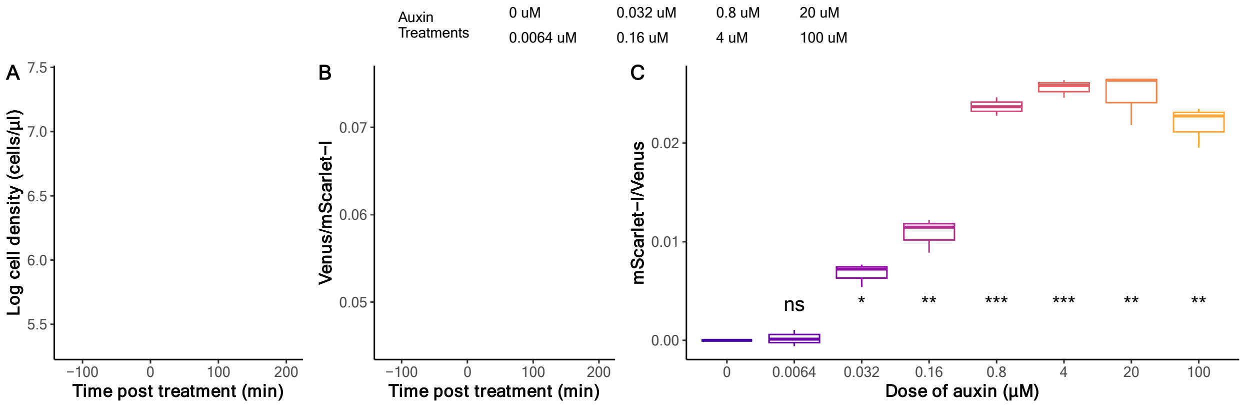

Supporting Figure S8. Defining conditions for AFB2 dual-fusion biosensor dose-response assay. *Time response of* ***(A)*** *cell growth and* ***(B)*** *Venus-Aux/IAA17 to AFB2-mScarlet-I ratio in response to different doses of auxin. Points represent the mean fluorescence of samples of approximately 10^4 cells from cultures measured every 30 minutes over a 6-hour experiment. (C) The box plot illustrates the foldchange between*

different doses of auxin treatment and solvent control at steady-state, which we defined as the last four measurements for each culture, more that 2 hours after treatment. T-test statistics show the comparison for each auxin treatment versus control (0 µM), with "ns" meaning "not significantly different", one, two, and three stars meaning significantly different with p < 0.05, p < 0.01, and p < 0.005, respectively.

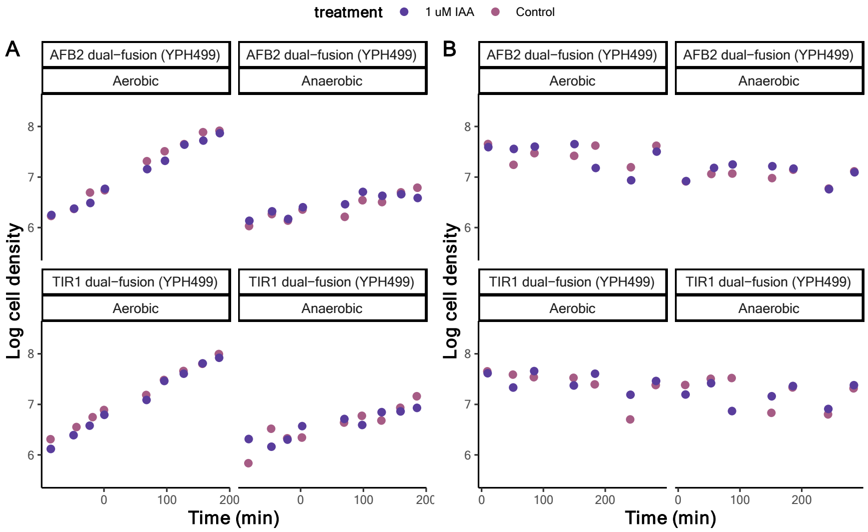

Supporting Figure S9. Cell growth conditions during auxin biosynthesis assays. *(A) Cell density measurements during the exponential phase of yeast cultures expressing different biosensors under aerobic (shaken at 300 RPM) and anaerobic (fermentative, static) growth conditions. Density was measured by flow cytometry. (B) Cell density measurements during stationary phase under aerobic and anaerobic growth conditions. The yeast cells carrying biosensors were continuously cultured from the preceding exponential phase and measurements began after 48-hours of incubation for determining auxin accumulation. The cultures were diluted 10x before the measurements via cytometry.*

Table S1: List of primers

Oligo ID	Name	Purpose	Sequence
oWL272	GPDP-to_TIR1_reverse	Biosensors cloning	CACACCACACCTGCACAAAACCTAATCCGTTAGTAGTAATGATTTGCCTGG
oWL271	backbone2_(BbvCI_mut)_reverse	Biosensors cloning	CACACCACACCTGCACAAAGGATCAGTTGGGTGCACGAGTGGG
oWL 270	backbone2_forward	Biosensors cloning	CACACCACACCTGCACAATGGCCCGGGGGATCCACTAG
oWL 269	mScarlet-I_TCYC_reverse	Biosensors cloning	CACACCACACCTGCACAAGCCAAATTAAAGCCTTCGAGCGTCCCA
oWL 268	mScarlet-I_TCYC_forward	Biosensors cloning	CACACCACACCTGCACAACAATGGTGAGCAAGGGCGAGGCA
oWL 267	ERBV-1-2a_reverse	Biosensors cloning	CACACCACACCTGCACAAATTGGACCTGGATTCAATTCAACATCACC
oWL 266	ERBV-1-2a_forward	Biosensors cloning	CACACCACACCTGCACAAGGTTCTGGTGGTGCTACTAATTTTTC
oWL 265	GPDP_to_AFB2_reverse	Biosensors cloning	CACACCACACCTGCACAAAACCGAGAATCCACACAAATGGCGGC
oWL 264	GPDP_to_AFB2_forward	Biosensors cloning	CACACCACACCTGCACAATGAGTTTATCATTATCAATACTCGCCATTTC
oWL 263	bakcbone1_reverse	Biosensors cloning	CACACCACACCTGCACAACTCAAGCTTATCGATACCGTCGACCTCGAGG
oWL 262	bakcbone1_forward	Biosensors cloning	CACACCACACCTGCACAATCCTCAGCATCTTTTACTTTCACCAGCGTTTCTGGG
oWL14	TCYC1_seq_R	Plasmid sequencing	GGGCGTGAATGTAAGCGTGACATAACTAAT
oWL15	PGPD_seq_F	Plasmid sequencing	CGGTAGGTATTGATTGTAATTCTG
oWL5	URA3_R	Checking genome integration	CTGTTCCAGCCCATATCCAACTTCC
oWL4	LEU2_R	Checking genome integration	CTACAGAAGCAGAAATACACGCAGTCA
oWL3	HIS3_R	Checking genome integration	CGAAAATTTGGGTGGCCAACTCTC
oWL2	TRP1_R	Checking genome integration	ATGGCTTGGCATGGCGATTTC
oWL1	tCYC1_F	Checking genome integration	ATTAGTTATGTCACGCTTACATTCACGCCC
oWL107	10B2_Seq_F	Orthorep plasmid sequencing	GCTGTCGCCGAAGAAGTTAAG
oWL106	TADH1_Seq_R	Orthorep plasmid sequencing	GAGAAAGCAACCTGACCTACAGG
oWL084	FDP_p10B2_COI1_F	Orthorep cloning	ATTGAGCGGCCGCGGATCCTCATGTGAGACGGAGG
oWL083	FDP_p10B2_COI1_(NheI)_R	Orthorep cloning	CAGGATCCTCCATGCTAGCTTTACATGTCTATGAGCTTATCACATGTTTC
oWL075	FDP_p10B2_TIR1_F	Orthorep cloning	ATTGAGCGGCCGCGGATCCTCATGTGAGACGGAGG
oWL074	FDP_p10B2_TIR1_(NheI)_R	Orthorep cloning	AGGCTATTCGCTTCTGCATGCTAGCTTTACATGTCTATGAGCTTATCACATGTTTC
oWL073	TIR1_3XFLAG6XHis_F	Orthorep cloning	AGACATGTAAAGCTAGCATGCAGAAGCGAATAGCCTTGTCGTTTCC
oWL072	TIR1_3XFLAG6XHis)_(NotI)_R	Orthorep cloning	GTCTCACATGAGGATCCGCGGCCGCTCAATGGTGGTGATGATGGTGCTTGTCATCG

Table S2: List of plasmids and *E.coli* cells

Plasmid ID	Name/Genotype	Source
TOP10	F- mcrA (mrr-hsdRMS-mcrBC) 80lacZ M15 lacX74 recA1 ara 139 (ara-leu)7697 galU galK rpsL (StrR) endA1 nupG	ThermoFisher
NEB® 10-beta	Δ(ara-leu) 7697 araD139 fhuA ΔlacX74 galK16 galE15 e14- ϕ80dlacZΔM15 recA1 relA1 endA1 nupG rpsL (StrR) rph spoT1 Δ(mrr-hsdRMS-mcrBC)	NEB®
pWL218	pAR318: TP-DNAP1-HIS3	This paper
pWL219	pAR633: TP-DNAP1-HIS3	This paper
pWL220	FDP-p10B2-URA3	This paper
pWL235	FDP_p10B2_TIR1_3XFLAG_6XHis	This paper
pWL233	pGP4G2-Venus-IAA17-ERBV2a-mScarlet	This paper
pWL273	pGP4G2-Venus-IAA17-ERBV2a-AFB2-mScarlet	This paper
pWL274	pGP4G2-Venus-IAA17-ERBV2a-TIR1-mScarlet	This paper
pWL63	pGP8G2_TIR1f_TIR1l_3xF6xH	Nemhauser lab
pWL57	pGP8G2_AFB2f_AFB2l_3xF6xH	Nemhauser lab

Table S3: List of *S. cerevisiae* strains

Yeast ID	Name/Genotype	Source
yWL161	pGP8G2_TIR1f_TIR1l_3xF6xH x pGP4G2-VENUS-IAA17-ERBV-1-2a-mScarlet	This paper
yWL162	pGP8G2_AFB2f_AFB2l_3xF6xH x pGP4G2-VENUS-IAA17-ERBV-1-2a-mScarlet	This paper
yWL185	pGP4G2-eGFP-ERBV-IAA17-TIR1-mScarlet x W303	This paper
yWL186	pGP4G2-Venus-IAA17-2A-AFB2-mScarlet x W303	This paper
yWL209	pGP4G2-eGFP-ERBV-IAA17-TIR1-mScarlet x YPH499	This paper
yWL210	pGP4G2-Venus-IAA17-2A-AFB2-mScarlet x YPH499	This paper
yWL159	pGP4G2-VENUS-IAA17-ERBV2a-mScarlet x W814-29B	This paper
yWL156	FDP_p10B2_TIR1_3XFLAG_6XHis x pAR633: TP-DNAP1-HIS3 x pGP4G2-Venus-IAA17-ERBV2a-mScarlet x F102 MATa rho0 (petite) ura3 his3 leu2 trp1 flo1 with WT p1 and WT p2	This paper
yWL155	FDP_p10B2_TIR1_3XFLAG_6XHis x pAR318: TP-DNAP1-HIS3 x pGP4G2-yEGFP-IAA17-ERBV-1-2a-mScarlet x F102 MATa rho0 (petite) ura3 his3 leu2 trp1 flo1 with WT p1 and WT p2	This paper
yWL145	pGP4G2-Venus-IAA17-ERBV2a-mScarlet x F102	This paper
yWL97	F102 MATa rho0 (petite) ura3 his3 leu2 trp1 flo1 with WT p1 and WT p2	This paper
yWL136	W303 (MATα ade2-1 ura3-1 his3-11,15 trp1-1 leu2-3,112 can1-100)	GG Lab
yWL1	W814-29B (MATα ade2-1 trp1-1 can1-100 ura3-1 leu2-3,112 his3-11,15)	Nemhauser lab
yWL2	W303-1A ADE2+ (MATa, leu2-3,112 trp1-1 can1-100 ura3-1 his3-11,15 ybp1-1)	Nemhauser lab
yWL64	YPH499 (MATa ura3-52 lys2-801_amber ade2-101_ochre trp1-Δ63 his3-Δ200 leu2-Δ1)	Nemhauser lab

Supporting Text S1: Supplemental reproducible analysis for: Genetically encoded, noise-tolerant, auxin biosensors in yeast

Table of Contents

Figure 4: Time-course response and ratiometric measurement of the single-fusion biosensors 10
Time-course degradation 10
Single-fusion CV plot 12
Data and analysis for dose-response curves of AFB2 and TIR1 biosensors 14
AFB2-based biosensor (yWL210 AFB2 in cis, single ratiometric construct) 14
TIR1-based biosensor (yWL209 TIR in cis, single ratiometric construct) 19
Figure 6A and 6B: Plot dose-response curve with ggplot 28
Figure 5A and Supporting Figure S3: Auxin-induced degradation and time-course assay for the biosensors in cis in multiple yeast strains. 32
Supporting Figure S2: CV plots 38
Figure 5B: Foldchange between the 50 µM auxin treatment and solvent control for each biosensor design 78
Figure 6: Dose-response models of biosensors 87
Figure 7: Auxin quantification by AFB2 dual-fustion Biosensor vs LC-MS 89
Figure 8A: Dose-response curve for auxin at early stationary phase, 2 replicates 102
Figure 8B: Auxin accumulation in different yeast cultures measured by the AFB2 dual-fusion biosensor 107
Figure 8C: The dual-fusion AFB2 biosensor predicts that auxin accumulates in stationary phase cultures 113
Supplement 6B: The bar plot of the foldchange between different doses of auxin treatment and solvent control 129
Session Info and Packages/Libraries 141

Figure 4: Time-course response and ratiometric measurement of the single-fusion biosensors

Time-course degradation

```
# Read in flow sets from 20200611 and 20200614
flowSet <- read.plateSet(path = "~/Google Drive/Shared
drives/PlantSynBioLab/Data/Mahbub/FlowSets/",
    pattern = "202006", phenoData = "annotation.txt")
flowSet <- flowSet[which(flowSet@phenoData@data$strain %in% c("T1T1",
"A2A2"))]


write.flowSet(flowSet, "flowSets/single-time-course")

flowSet <- read.flowSet(path = "Data for publication/single-time-course",
phenoData = "annotation.txt")
# load gates for this strain/cytometer
load("Data for publication/PSB_Accuri_W303.RData")
data_sum <- summarizeFlow(flowset = flowSet, ploidy = "diploid", only =
"singlets",
    channel = c("FL1.A", "FL4.A"))
# # [1] "Gating with diploid singlet gates..."
time0_14 <- data_sum %>%
    dplyr::filter(name == "21D10.fcs") %>%
    pull(btime)
time0_11 <- data_sum %>%
    dplyr::filter(name == "11G02.fcs") %>%
    pull(btime)
data_sum <- data_sum %>%
    mutate(time = case_when(folder == "20200611_AFB_epistasis" ~ .$btime -
time0_11,
        folder == "20200614_AFB_epistasis" ~ .$btime - time0_14))

shapes <- c(DMSO = 1, IAA = 16)
lines <- c(DMSO = 2, IAA = 1)


data_sum$ratio <- data_sum$FL1.Amean/data_sum$FL4.Amean
data_sum$Venus <- data_sum$FL1.Amean/10000
data_sum_long <- data_sum %>%
    dplyr::select(time, treatment, yWL, folder, strain, ratio, Venus) %>%
    pivot_longer(cols = c(ratio, Venus), names_to = "parameter")
deg_plot <- ggplot(data = subset(data_sum_long, yWL == "166"), aes(x = time,
y = value,
    shape = treatment, color = treatment, group = interaction(treatment,
folder))) +
    geom_point() + labs(y = "Intensity (AU)", x = "Time post treatment
(min)") +
```

```r
    facet_wrap(~fct_rev(parameter), scales = "free") +
scale_shape_manual(values = shapes) +
    geom_line(aes(linetype = treatment)) + scale_linetype_manual(values =
lines) +
    scale_color_viridis_d(option = "D", end = 0.75, direction = -1) +
theme_test()
deg_plot
```

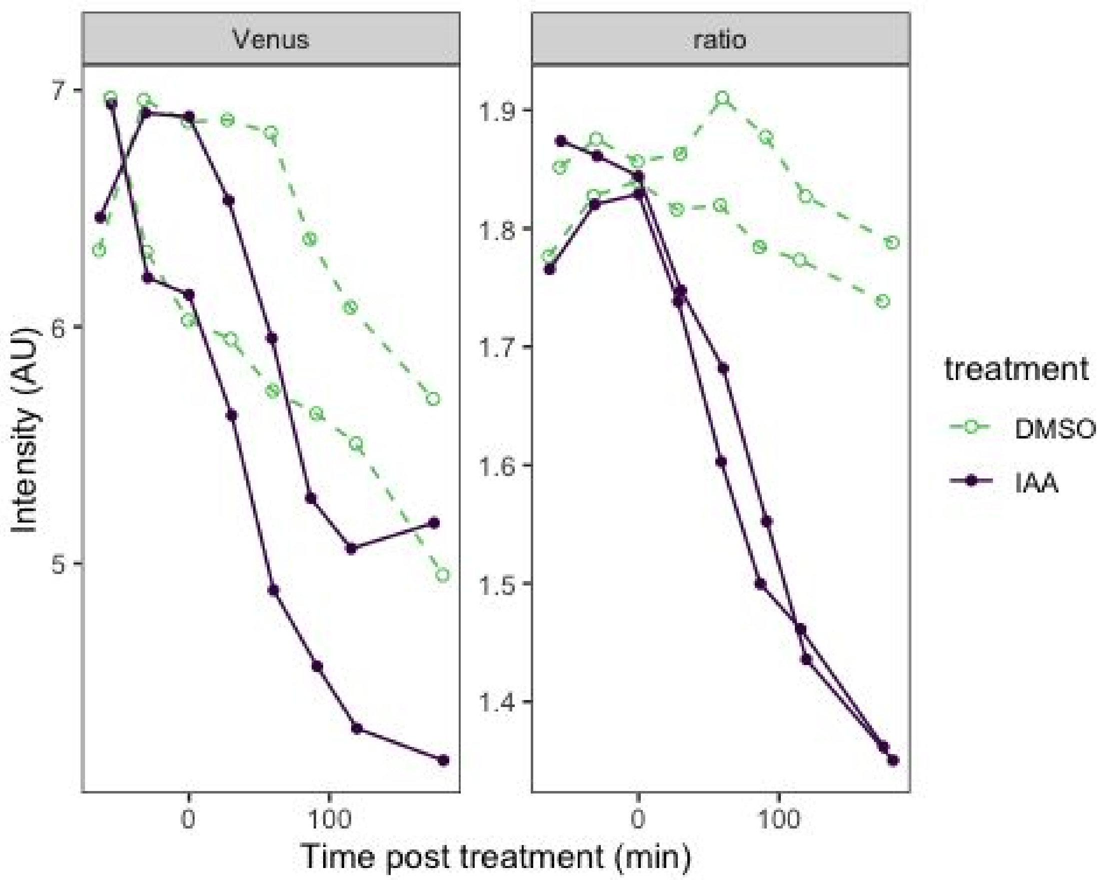


```r
data <- flowTime::tidyFlow(flowSet, ploidy = "diploid", only = "singlets")
# # [1] "No further gating applied."
# # [1] "Converting events..."
# # Joining with `by = join_by(name)`

# get late time points for one strain
last_reading <- tail(unique(data[which(data$yWL == 166), "name"]), 4) %>%
    head(2)
data <- dplyr::filter(data, yWL == "166" & name %in% last_reading)
# clean this up, cut off zeros
data <- subset(data, FL1.A > 1 & FL4.A > 1)
data$FLratio <- data$FL1.A/data$FL4.A
range(data$FL1.A)
# # [1]     21 697262
# calculate cvs
```

```
sd(data$FLratio)/mean(data$FLratio)
# # [1] 0.1842678
range(data$FLratio)
# # [1] 0.002628614 3.836085188
```

Single-fusion CV plot

```
# calculate normalized values
data$Venus <- data$FL1.A/median(data$FL1.A)
data$mScarlet <- data$FL4.A/median(data$FL4.A)
data$ratio <- data$FLratio/median(data$FLratio)
# make a tidy, long dataset
data_long <- data %>%
    dplyr::select(treatment, Venus, mScarlet, ratio) %>%
    pivot_longer(cols = c(Venus, mScarlet, ratio), names_to = "parameter",
values_to = "value")


# need to also format CVs approriately for annotating
cv <- function(x) return(round(sd(x)/mean(x), 2))
CVs <- data %>%
    group_by(treatment) %>%
    summarise(across(where(is_double), cv))
CVs <- CVs %>%
    dplyr::select(treatment, Venus, mScarlet, ratio) %>%
    pivot_longer(cols = c(Venus, mScarlet, ratio), names_to = "parameter",
values_to = "value")


CV_plot <- ggplot(data = data_long, mapping = aes(x = value, color =
treatment)) +
    geom_density() + coord_cartesian(x = c(0, 2)) + labs(x = "median
normalized intensity",
    color = "treatment") + facet_grid(fct_relevel(parameter, "Venus") ~ .) +
theme_test() +
    geom_text(data = subset(CVs, treatment == "IAA"), aes(label = paste0("CV
= ",
        value)), x = 0.3, y = 2) + geom_text(data = subset(CVs, treatment ==
"DMSO"),
    aes(label = paste0("CV = ", value)), x = 1.8, y = 2) +
scale_color_viridis_d(option = "D",
    end = 0.75, direction = -1)
CV_plot
```

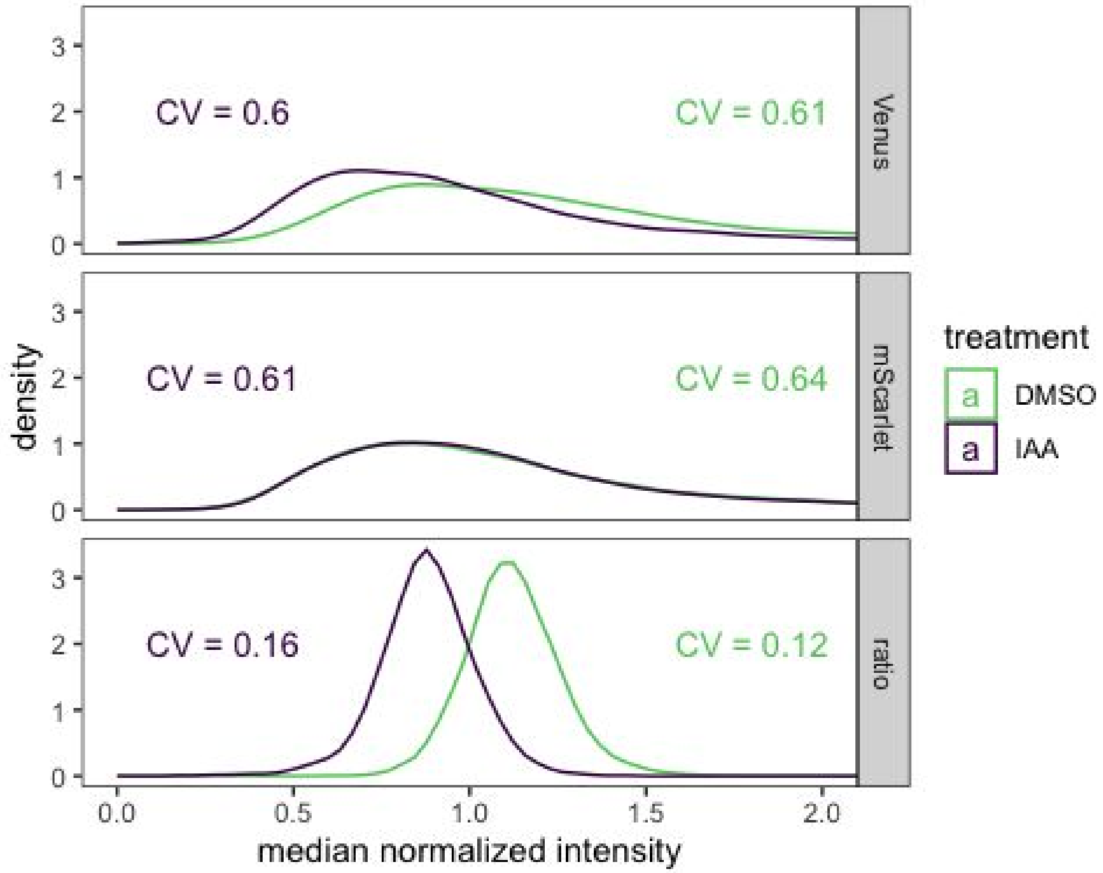


```
layout <- "
AAAAB
CCCCC
"
CV_plot + guide_area() + deg_plot + plot_annotation(tag_levels = "A") +
plot_layout(guides = "collect",
    heights = c(5, 2), design = layout)
```

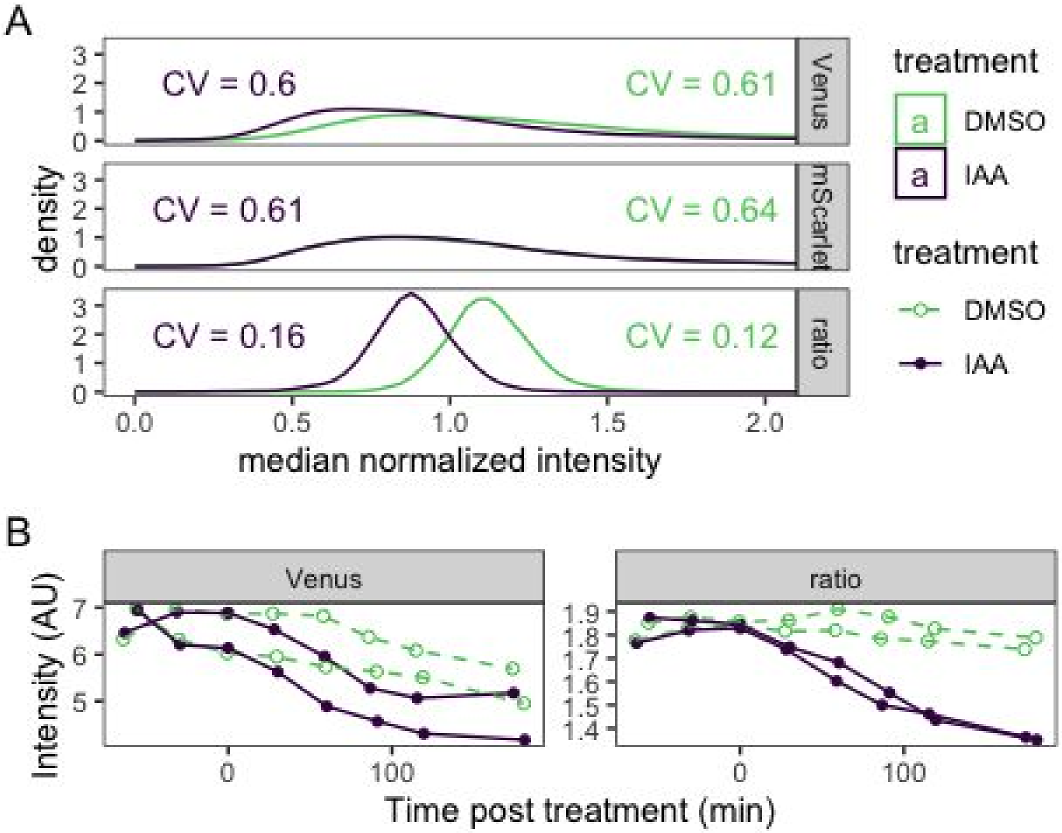


```
ggsave("ratio-deg.pdf", width = 5, height = 5)
ggsave("ratio-deg.png", width = 5, height = 5)
```

Data and analysis for dose-response curves of AFB2 and TIR1 biosensors

AFB2-based biosensor (yWL210 AFB2 in cis, single ratiometric construct)

```
plate_all_210 <- read.plateSet(path = "Data for
publication/11212022_DRA_overlaydata/Combine Data_yWL210/All data/",
    pattern = "DRA-*")


annotation <- createAnnotation(yourFlowSet = plate_all_210)
write.csv(annotation, "Data for
publication/11212022_DRA_overlaydata/overlaydata_annotation_yWL210_datagated_
exJan31.csv")


annotation <- read.csv("Data for
publication/11212022_DRA_overlaydata/overlaydata_annotation_yWL210_datagated_
exJan31.csv")
```

```
aplate_all_210 <- annotateFlowSet(yourFlowSet = plate_all_210, annotation_df
= annotation,
    mergeBy = "name")
head(rownames(pData(aplate_all_210)))
# # [1] "1Pat-01182023-Biosensors_DRA09_Experiment_yWL210_IAA 0 uM.fcs"
# # [2] "1Pat-01182023-Biosensors_DRA09_Experiment_yWL210_IAA 0.0000102
uM.fcs"
# # [3] "1Pat-01182023-Biosensors_DRA09_Experiment_yWL210_IAA 0.0000512
uM.fcs"
# # [4] "1Pat-01182023-Biosensors_DRA09_Experiment_yWL210_IAA 0.000256
uM.fcs"
# # [5] "1Pat-01182023-Biosensors_DRA09_Experiment_yWL210_IAA 0.00128 uM.fcs"
# # [6] "1Pat-01182023-Biosensors_DRA09_Experiment_yWL210_IAA 0.0064 uM.fcs"
head(pData(aplate_all_210))
# #
name
# # 1Pat-01182023-Biosensors_DRA09_Experiment_yWL210_IAA 0 uM.fcs
1Pat-01182023-Biosensors_DRA09_Experiment_yWL210_IAA 0 uM.fcs
# # 1Pat-01182023-Biosensors_DRA09_Experiment_yWL210_IAA 0.0000102 uM.fcs
1Pat-01182023-Biosensors_DRA09_Experiment_yWL210_IAA 0.0000102 uM.fcs
# # 1Pat-01182023-Biosensors_DRA09_Experiment_yWL210_IAA 0.0000512 uM.fcs
1Pat-01182023-Biosensors_DRA09_Experiment_yWL210_IAA 0.0000512 uM.fcs
# # 1Pat-01182023-Biosensors_DRA09_Experiment_yWL210_IAA 0.000256 uM.fcs
1Pat-01182023-Biosensors_DRA09_Experiment_yWL210_IAA 0.000256 uM.fcs
# # 1Pat-01182023-Biosensors_DRA09_Experiment_yWL210_IAA 0.00128 uM.fcs
1Pat-01182023-Biosensors_DRA09_Experiment_yWL210_IAA 0.00128 uM.fcs
# # 1Pat-01182023-Biosensors_DRA09_Experiment_yWL210_IAA 0.0064 uM.fcs
1Pat-01182023-Biosensors_DRA09_Experiment_yWL210_IAA 0.0064 uM.fcs
# #
folder
# # 1Pat-01182023-Biosensors_DRA09_Experiment_yWL210_IAA 0 uM.fcs
DRA-01182023_read9
# # 1Pat-01182023-Biosensors_DRA09_Experiment_yWL210_IAA 0.0000102 uM.fcs
DRA-01182023_read9
# # 1Pat-01182023-Biosensors_DRA09_Experiment_yWL210_IAA 0.0000512 uM.fcs
DRA-01182023_read9
# # 1Pat-01182023-Biosensors_DRA09_Experiment_yWL210_IAA 0.000256 uM.fcs
DRA-01182023_read9
# # 1Pat-01182023-Biosensors_DRA09_Experiment_yWL210_IAA 0.00128 uM.fcs
DRA-01182023_read9
# # 1Pat-01182023-Biosensors_DRA09_Experiment_yWL210_IAA 0.0064 uM.fcs
DRA-01182023_read9
# #                                                                       X
# # 1Pat-01182023-Biosensors_DRA09_Experiment_yWL210_IAA 0 uM.fcs         1
# # 1Pat-01182023-Biosensors_DRA09_Experiment_yWL210_IAA 0.0000102 uM.fcs 2
# # 1Pat-01182023-Biosensors_DRA09_Experiment_yWL210_IAA 0.0000512 uM.fcs 3
# # 1Pat-01182023-Biosensors_DRA09_Experiment_yWL210_IAA 0.000256 uM.fcs  4
# # 1Pat-01182023-Biosensors_DRA09_Experiment_yWL210_IAA 0.00128 uM.fcs   5
# # 1Pat-01182023-Biosensors_DRA09_Experiment_yWL210_IAA 0.0064 uM.fcs    6
# #
```

```
treatment
# # 1Pat-01182023-Biosensors_DRA09_Experiment_yWL210_IAA 0 uM.fcs
0 uM
# # 1Pat-01182023-Biosensors_DRA09_Experiment_yWL210_IAA 0.0000102 uM.fcs
0.0000102 uM
# # 1Pat-01182023-Biosensors_DRA09_Experiment_yWL210_IAA 0.0000512 uM.fcs
0.0000512 uM
# # 1Pat-01182023-Biosensors_DRA09_Experiment_yWL210_IAA 0.000256 uM.fcs
0.000256 uM
# # 1Pat-01182023-Biosensors_DRA09_Experiment_yWL210_IAA 0.00128 uM.fcs
0.00128 uM
# # 1Pat-01182023-Biosensors_DRA09_Experiment_yWL210_IAA 0.0064 uM.fcs
0.0064 uM
# #
dose
# # 1Pat-01182023-Biosensors_DRA09_Experiment_yWL210_IAA 0 uM.fcs
0.00e+00
# # 1Pat-01182023-Biosensors_DRA09_Experiment_yWL210_IAA 0.0000102 uM.fcs
1.02e-05
# # 1Pat-01182023-Biosensors_DRA09_Experiment_yWL210_IAA 0.0000512 uM.fcs
5.12e-05
# # 1Pat-01182023-Biosensors_DRA09_Experiment_yWL210_IAA 0.000256 uM.fcs
2.56e-04
# # 1Pat-01182023-Biosensors_DRA09_Experiment_yWL210_IAA 0.00128 uM.fcs
1.28e-03
# # 1Pat-01182023-Biosensors_DRA09_Experiment_yWL210_IAA 0.0064 uM.fcs
6.40e-03
# #
replicate
# # 1Pat-01182023-Biosensors_DRA09_Experiment_yWL210_IAA 0 uM.fcs
1
# # 1Pat-01182023-Biosensors_DRA09_Experiment_yWL210_IAA 0.0000102 uM.fcs
1
# # 1Pat-01182023-Biosensors_DRA09_Experiment_yWL210_IAA 0.0000512 uM.fcs
1
# # 1Pat-01182023-Biosensors_DRA09_Experiment_yWL210_IAA 0.000256 uM.fcs
1
# # 1Pat-01182023-Biosensors_DRA09_Experiment_yWL210_IAA 0.00128 uM.fcs
1
# # 1Pat-01182023-Biosensors_DRA09_Experiment_yWL210_IAA 0.0064 uM.fcs
1
# #
date
# # 1Pat-01182023-Biosensors_DRA09_Experiment_yWL210_IAA 0 uM.fcs         18-
Jan-23
# # 1Pat-01182023-Biosensors_DRA09_Experiment_yWL210_IAA 0.0000102 uM.fcs 18-
Jan-23
# # 1Pat-01182023-Biosensors_DRA09_Experiment_yWL210_IAA 0.0000512 uM.fcs 18-
Jan-23
# # 1Pat-01182023-Biosensors_DRA09_Experiment_yWL210_IAA 0.000256 uM.fcs  18-
```

```
Jan-23
# # 1Pat-01182023-Biosensors_DRA09_Experiment_yWL210_IAA 0.00128 uM.fcs   18-
Jan-23
# # 1Pat-01182023-Biosensors_DRA09_Experiment_yWL210_IAA 0.0064 uM.fcs    18-
Jan-23

plate_all_210_sum <- summarizeFlow(aplate_all_210, channel = c("BL1.A",
"YL1.A"),
    gated = TRUE)
# # [1] "Summarizing all events..."
### Dose-response curve Comparing log-logistic and Weibull models (Figure 2
in
### Ritz (2009))

fitdrc.m1 <- drm(YL1.Amean/BL1.Amean ~ dose, data = plate_all_210_sum, fct =
LL.4())
fitdrc.m2 <- drm(YL1.Amean/BL1.Amean ~ dose, data = plate_all_210_sum, fct =
W1.4())
# fitdrc.m3 <- drm(YL1.Amean/BL1.Amean~dose, data=plate_all_210_sum, fct =
# W2.4())


model.LL4_all_210 <- drm(YL1.Amean/BL1.Amean ~ dose, data =
plate_all_210_sum, fct = LL.4(names = c("Slope",
    "Lower Limit", "Upper Limit", "ED50")))
plot(model.LL4_all_210, broken = TRUE, type = "none", lty = 1, lwd = 5, xlab
= "Extracellular IAA concentration (uM)",
    ylab = "Response Signal")
# plot(model.LL4_all_210, broken = TRUE, col = 'black', add=TRUE)
plot(model.LL4_all_210, broken = TRUE, type = "confidence", col = "black",
add = TRUE)
summary(model.LL4_all_210)
# #
# # Model fitted: Log-logistic (ED50 as parameter) (4 parms)
# #
# # Parameter estimates:
# #
# #                              Estimate Std. Error t-value p-value
# # Slope:(Intercept)          -1.5032417  0.6338695 -2.3715 0.01985 *
# # Lower Limit:(Intercept)     0.0526074  0.0020790 25.3043 < 2e-16 ***
# # Upper Limit:(Intercept)     0.0845079  0.0021128 39.9976 < 2e-16 ***
# # ED50:(Intercept)            0.0403739  0.0159429  2.5324 0.01306 *
# # ---
# # Signif. codes:  0 '***' 0.001 '**' 0.01 '*' 0.05 '.' 0.1 ' ' 1
# #
# # Residual standard error:
# #
# #  0.01246335 (90 degrees of freedom)
```

```
replicate1_210 <- drm(YL1.Amean/BL1.Amean ~ dose, data =
subset(plate_all_210_sum,
    replicate == "1"), fct = LL.4())
replicate2_210 <- drm(YL1.Amean/BL1.Amean ~ dose, data =
subset(plate_all_210_sum,
    replicate == "2"), fct = LL.4())
replicate3_210 <- drm(YL1.Amean/BL1.Amean ~ dose, data =
subset(plate_all_210_sum,
    replicate == "3"), fct = LL.4())
replicate4_210 <- drm(YL1.Amean/BL1.Amean ~ dose, data =
subset(plate_all_210_sum,
    replicate == "4"), fct = LL.4())
replicate5_210 <- drm(YL1.Amean/BL1.Amean ~ dose, data =
subset(plate_all_210_sum,
    replicate == "5"), fct = LL.4())
replicate6_210 <- drm(YL1.Amean/BL1.Amean ~ dose, data =
subset(plate_all_210_sum,
    replicate == "6"), fct = LL.4())
replicate7_210 <- drm(YL1.Amean/BL1.Amean ~ dose, data =
subset(plate_all_210_sum,
    replicate == "7"), fct = LL.4())

plot(replicate1_210, broken = TRUE, add = TRUE, type = "all", col = "dark
green",
    lty = 2)
plot(replicate2_210, broken = TRUE, add = TRUE, type = "all", col = "dark
blue",
    lty = 2)
plot(replicate3_210, broken = TRUE, add = TRUE, type = "all", col = "yellow",
lty = 2)
plot(replicate4_210, broken = TRUE, add = TRUE, type = "all", col = "dark
grey",
    lty = 2)
plot(replicate5_210, broken = TRUE, add = TRUE, type = "all", col = "dark
orange",
    lty = 2)
plot(replicate6_210, broken = TRUE, add = TRUE, type = "all", col = "brown",
lty = 2)
plot(replicate7_210, broken = TRUE, add = TRUE, type = "all", col = "dark
red", lty = 2)
```

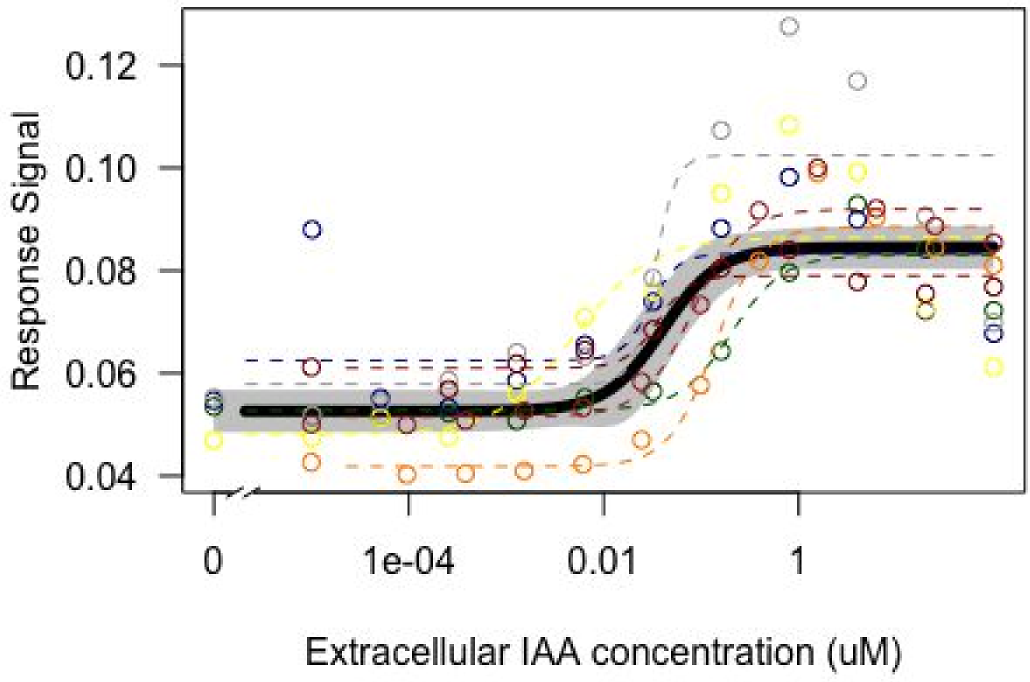

TIR1-based biosensor (yWL209 TIR in cis, single ratiometric construct)

```
plate_all_209 <- read.plateSet(path = "Data for
publication/11212022_DRA_overlaydata/Combine Data_yWL209/",
    pattern = "DRA-*")

annotation <- createAnnotation(yourFlowSet = plate_all_209)
write.csv(annotation, "Data for
publication/11212022_DRA_overlaydata/overlaydata_annotation_yWL209.csv")

annotation <- read.csv("Data for
publication/11212022_DRA_overlaydata/overlaydata_annotation_yWL209.csv")

aplate_all_209 <- annotateFlowSet(yourFlowSet = plate_all_209, annotation_df
= annotation,
    mergeBy = "name")
head(rownames(pData(aplate_all_209)))
# # [1] "1Pat-03182023-Biosensors_DRA08_Experiment_yWL209-PC_IAA 0 uM.fcs"
# # [2] "1Pat-03182023-Biosensors_DRA08_Experiment_yWL209-PC_IAA 0.0000102
uM.fcs"
# # [3] "1Pat-03182023-Biosensors_DRA08_Experiment_yWL209-PC_IAA 0.0000512
uM.fcs"
# # [4] "1Pat-03182023-Biosensors_DRA08_Experiment_yWL209-PC_IAA 0.000256
```

```
uM.fcs"
# # [5] "1Pat-03182023-Biosensors_DRA08_Experiment_yWL209-PC_IAA 0.00128
uM.fcs"
# # [6] "1Pat-03182023-Biosensors_DRA08_Experiment_yWL209-PC_IAA 0.0064
uM.fcs"
head(pData(aplate_all_209))
# #
name
# # 1Pat-03182023-Biosensors_DRA08_Experiment_yWL209-PC_IAA 0 uM.fcs
1Pat-03182023-Biosensors_DRA08_Experiment_yWL209-PC_IAA 0 uM.fcs
# # 1Pat-03182023-Biosensors_DRA08_Experiment_yWL209-PC_IAA 0.0000102 uM.fcs
1Pat-03182023-Biosensors_DRA08_Experiment_yWL209-PC_IAA 0.0000102 uM.fcs
# # 1Pat-03182023-Biosensors_DRA08_Experiment_yWL209-PC_IAA 0.0000512 uM.fcs
1Pat-03182023-Biosensors_DRA08_Experiment_yWL209-PC_IAA 0.0000512 uM.fcs
# # 1Pat-03182023-Biosensors_DRA08_Experiment_yWL209-PC_IAA 0.000256 uM.fcs
1Pat-03182023-Biosensors_DRA08_Experiment_yWL209-PC_IAA 0.000256 uM.fcs
# # 1Pat-03182023-Biosensors_DRA08_Experiment_yWL209-PC_IAA 0.00128 uM.fcs
1Pat-03182023-Biosensors_DRA08_Experiment_yWL209-PC_IAA 0.00128 uM.fcs
# # 1Pat-03182023-Biosensors_DRA08_Experiment_yWL209-PC_IAA 0.0064 uM.fcs
1Pat-03182023-Biosensors_DRA08_Experiment_yWL209-PC_IAA 0.0064 uM.fcs
# #
folder
# # 1Pat-03182023-Biosensors_DRA08_Experiment_yWL209-PC_IAA 0 uM.fcs
DRA-03182023_read8
# # 1Pat-03182023-Biosensors_DRA08_Experiment_yWL209-PC_IAA 0.0000102 uM.fcs
DRA-03182023_read8
# # 1Pat-03182023-Biosensors_DRA08_Experiment_yWL209-PC_IAA 0.0000512 uM.fcs
DRA-03182023_read8
# # 1Pat-03182023-Biosensors_DRA08_Experiment_yWL209-PC_IAA 0.000256 uM.fcs
DRA-03182023_read8
# # 1Pat-03182023-Biosensors_DRA08_Experiment_yWL209-PC_IAA 0.00128 uM.fcs
DRA-03182023_read8
# # 1Pat-03182023-Biosensors_DRA08_Experiment_yWL209-PC_IAA 0.0064 uM.fcs
DRA-03182023_read8
# #
X
# # 1Pat-03182023-Biosensors_DRA08_Experiment_yWL209-PC_IAA 0 uM.fcs
1
# # 1Pat-03182023-Biosensors_DRA08_Experiment_yWL209-PC_IAA 0.0000102 uM.fcs
2
# # 1Pat-03182023-Biosensors_DRA08_Experiment_yWL209-PC_IAA 0.0000512 uM.fcs
3
# # 1Pat-03182023-Biosensors_DRA08_Experiment_yWL209-PC_IAA 0.000256 uM.fcs
4
# # 1Pat-03182023-Biosensors_DRA08_Experiment_yWL209-PC_IAA 0.00128 uM.fcs
5
# # 1Pat-03182023-Biosensors_DRA08_Experiment_yWL209-PC_IAA 0.0064 uM.fcs
6
# #
treatment
```

```
# # 1Pat-03182023-Biosensors_DRA08_Experiment_yWL209-PC_IAA 0 uM.fcs
0 uM
# # 1Pat-03182023-Biosensors_DRA08_Experiment_yWL209-PC_IAA 0.0000102 uM.fcs
0.0000102 uM
# # 1Pat-03182023-Biosensors_DRA08_Experiment_yWL209-PC_IAA 0.0000512 uM.fcs
0.0000512 uM
# # 1Pat-03182023-Biosensors_DRA08_Experiment_yWL209-PC_IAA 0.000256 uM.fcs
0.000256 uM
# # 1Pat-03182023-Biosensors_DRA08_Experiment_yWL209-PC_IAA 0.00128 uM.fcs
0.00128 uM
# # 1Pat-03182023-Biosensors_DRA08_Experiment_yWL209-PC_IAA 0.0064 uM.fcs
0.0064 uM
# #
dose
# # 1Pat-03182023-Biosensors_DRA08_Experiment_yWL209-PC_IAA 0 uM.fcs
0.00e+00
# # 1Pat-03182023-Biosensors_DRA08_Experiment_yWL209-PC_IAA 0.0000102 uM.fcs
1.02e-05
# # 1Pat-03182023-Biosensors_DRA08_Experiment_yWL209-PC_IAA 0.0000512 uM.fcs
5.12e-05
# # 1Pat-03182023-Biosensors_DRA08_Experiment_yWL209-PC_IAA 0.000256 uM.fcs
2.56e-04
# # 1Pat-03182023-Biosensors_DRA08_Experiment_yWL209-PC_IAA 0.00128 uM.fcs
1.28e-03
# # 1Pat-03182023-Biosensors_DRA08_Experiment_yWL209-PC_IAA 0.0064 uM.fcs
6.40e-03
# #
replicate
# # 1Pat-03182023-Biosensors_DRA08_Experiment_yWL209-PC_IAA 0 uM.fcs
4
# # 1Pat-03182023-Biosensors_DRA08_Experiment_yWL209-PC_IAA 0.0000102 uM.fcs
4
# # 1Pat-03182023-Biosensors_DRA08_Experiment_yWL209-PC_IAA 0.0000512 uM.fcs
4
# # 1Pat-03182023-Biosensors_DRA08_Experiment_yWL209-PC_IAA 0.000256 uM.fcs
4
# # 1Pat-03182023-Biosensors_DRA08_Experiment_yWL209-PC_IAA 0.00128 uM.fcs
4
# # 1Pat-03182023-Biosensors_DRA08_Experiment_yWL209-PC_IAA 0.0064 uM.fcs
4
# #
point
# # 1Pat-03182023-Biosensors_DRA08_Experiment_yWL209-PC_IAA 0 uM.fcs
8
# # 1Pat-03182023-Biosensors_DRA08_Experiment_yWL209-PC_IAA 0.0000102 uM.fcs
8
# # 1Pat-03182023-Biosensors_DRA08_Experiment_yWL209-PC_IAA 0.0000512 uM.fcs
8
# # 1Pat-03182023-Biosensors_DRA08_Experiment_yWL209-PC_IAA 0.000256 uM.fcs
8
```

```
# # 1Pat-03182023-Biosensors_DRA08_Experiment_yWL209-PC_IAA 0.00128 uM.fcs
8
# # 1Pat-03182023-Biosensors_DRA08_Experiment_yWL209-PC_IAA 0.0064 uM.fcs
8
# #
colony
# # 1Pat-03182023-Biosensors_DRA08_Experiment_yWL209-PC_IAA 0 uM.fcs
1
# # 1Pat-03182023-Biosensors_DRA08_Experiment_yWL209-PC_IAA 0.0000102 uM.fcs
1
# # 1Pat-03182023-Biosensors_DRA08_Experiment_yWL209-PC_IAA 0.0000512 uM.fcs
1
# # 1Pat-03182023-Biosensors_DRA08_Experiment_yWL209-PC_IAA 0.000256 uM.fcs
1
# # 1Pat-03182023-Biosensors_DRA08_Experiment_yWL209-PC_IAA 0.00128 uM.fcs
1
# # 1Pat-03182023-Biosensors_DRA08_Experiment_yWL209-PC_IAA 0.0064 uM.fcs
1

dat_sumGp_overlaydata_209 <- summarizeFlow(aplate_all_209, gated = TRUE,
channel = "BL1.A")
# # [1] "Summarizing all events..."

dat_sumRp_overlaydata_209 <- summarizeFlow(aplate_all_209, gated = TRUE,
channel = "YL1.A")
# # [1] "Summarizing all events..."

dat_sumGp_overlaydata_209$YL1.Amean <- dat_sumRp_overlaydata_209$YL1.Amean

### Dose-response curve

model.LL4_rep1_209 <- drm(YL1.Amean/BL1.Amean ~ dose, data =
subset(dat_sumGp_overlaydata_209,
    replicate == "1"), fct = LL.4(names = c("Slope", "Lower Limit", "Upper
Limit",
    "ED50")))
plot(model.LL4_rep1_209, type = "all", col = "red", lty = 1, lwd = 2, xlab =
"Extracellular IAA concentration (uM)",
    ylab = "Red/Green Fluorescent Ratio")
plot(model.LL4_rep1_209, broken = TRUE, type = "confidence", col = "red", add
= TRUE)
```

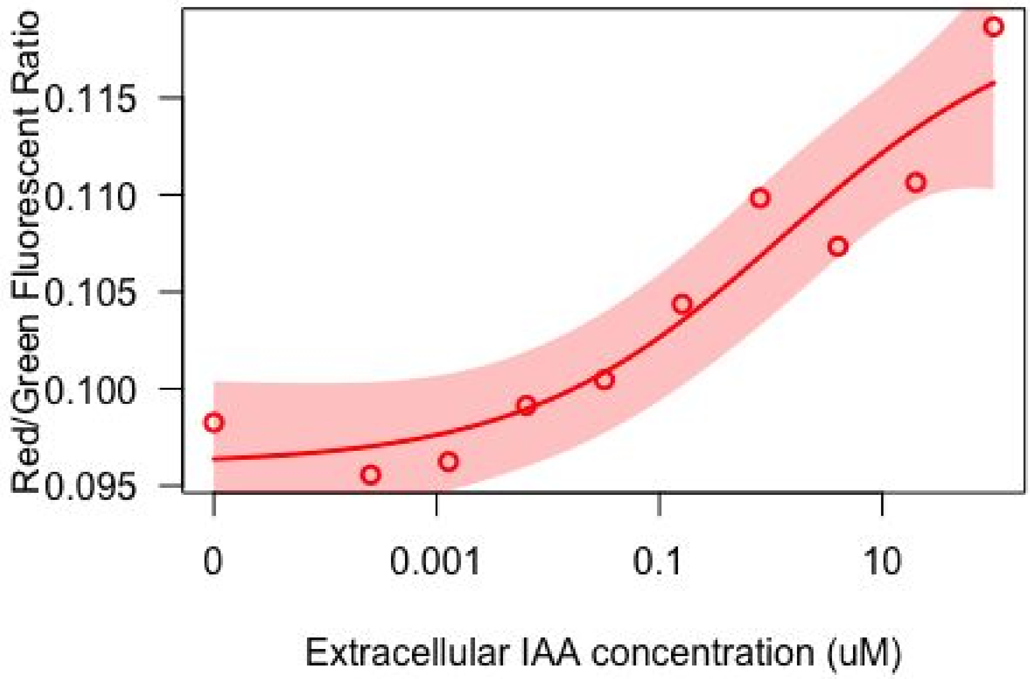


```
print(summary(model.LL4_rep1_209))
# #
# # Model fitted: Log-logistic (ED50 as parameter) (4 parms)
# #
# # Parameter estimates:
# #
# #                            Estimate Std. Error t-value   p-value
# # Slope:(Intercept)        -0.3721096  0.1407488 -2.6438   0.03835 *
# # Lower Limit:(Intercept)   0.0960856  0.0019155 50.1617 4.211e-09 ***
# # Upper Limit:(Intercept)   0.1196734  0.0064435 18.5728 1.572e-06 ***
# # ED50:(Intercept)          1.2919540  2.3836220  0.5420   0.60734
# # ---
# # Signif. codes:  0 '***' 0.001 '**' 0.01 '*' 0.05 '.' 0.1 ' ' 1
# #
# # Residual standard error:
# #
# #  0.002712198 (6 degrees of freedom)


model.LL4_rep2_209 <- drm(YL1.Amean/BL1.Amean ~ dose, data =
subset(dat_sumGp_overlaydata_209,
    replicate == "2"), fct = LL.4(names = c("Slope", "Lower Limit", "Upper
Limit",
```

```
    "ED50")))
plot(model.LL4_rep2_209, type = "all", col = "blue", lty = 1, lwd = 2, xlab =
"Extracellular IAA concentration (uM)",
    ylab = "Red/Green Fluorescent Ratio")
plot(model.LL4_rep2_209, broken = TRUE, type = "confidence", col = "blue",
add = TRUE)
```

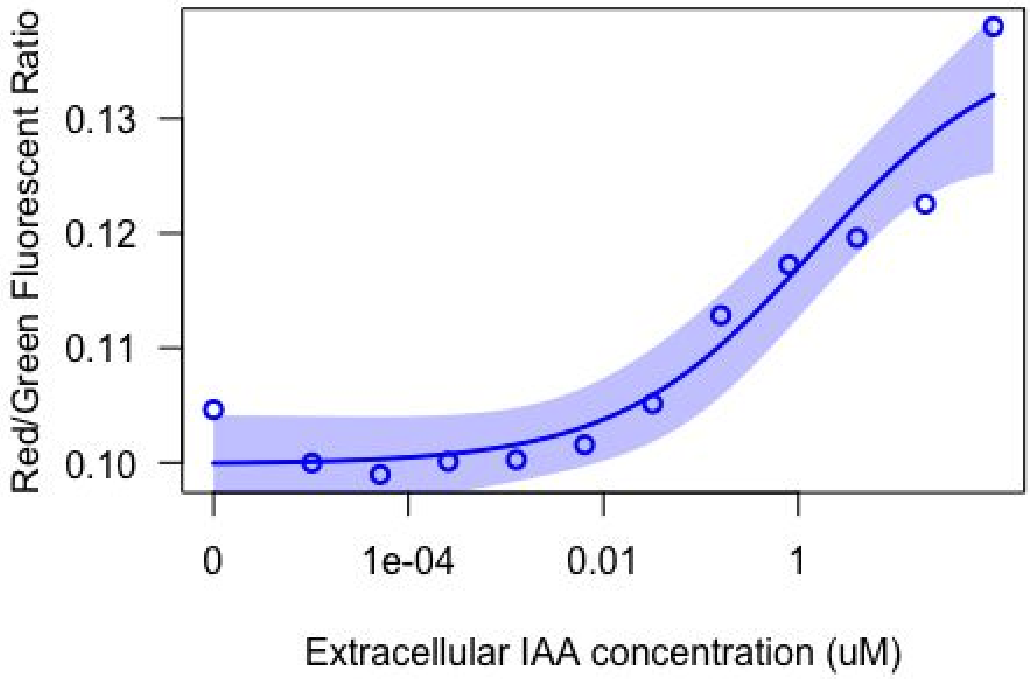


```
print(summary(model.LL4_rep2_209))
# #
# # Model fitted: Log-logistic (ED50 as parameter) (4 parms)
# #
# # Parameter estimates:
# #
# #                              Estimate Std. Error t-value   p-value
# # Slope:(Intercept)        -0.4323121  0.1153679 -3.7472  0.005646 **
# # Lower Limit:(Intercept)  0.0998826  0.0018914 52.8084 1.833e-11 ***
# # Upper Limit:(Intercept)  0.1372100  0.0062312 22.0197 1.910e-08 ***
# # ED50:(Intercept)         1.4546642  1.4213107  1.0235  0.336036
# # ---
# # Signif. codes:  0 '***' 0.001 '**' 0.01 '*' 0.05 '.' 0.1 ' ' 1
# #
# # Residual standard error:
# #
```

```
## #  0.003755989 (8 degrees of freedom)
```

```
model.LL4_rep3_209 <- drm(YL1.Amean/BL1.Amean ~ dose, data =
subset(dat_sumGp_overlaydata_209,
    replicate == "3"), fct = LL.4(names = c("Slope", "Lower Limit", "Upper
Limit",
    "ED50")))
plot(model.LL4_rep3_209, type = "all", col = "dark green", lty = 1, lwd = 2,
xlab = "Extracellular IAA concentration (uM)",
    ylab = "Red/Green Fluorescent Ratio")
plot(model.LL4_rep3_209, broken = TRUE, type = "confidence", col = "dark
green",
    add = TRUE)
```

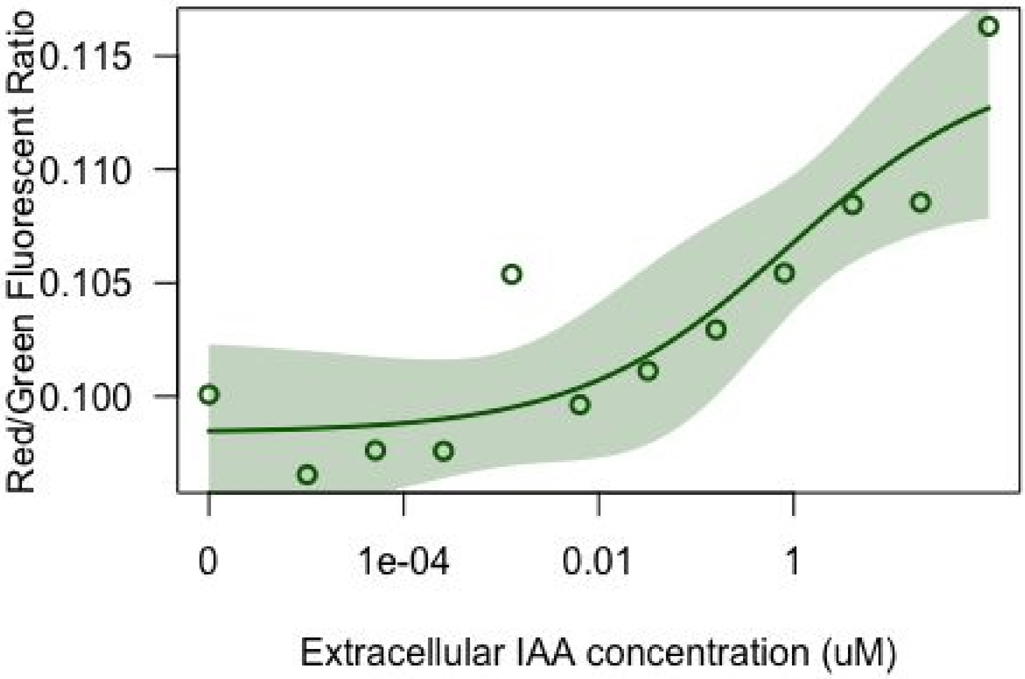


```
print(summary(model.LL4_rep3_209))
## #
## # Model fitted: Log-logistic (ED50 as parameter) (4 parms)
## #
## # Parameter estimates:
## #
## #                               Estimate Std. Error t-value   p-value
```

```
# # Slope:(Intercept)        -0.4007190  0.2177393 -1.8404    0.1030
# # Lower Limit:(Intercept)  0.0984103  0.0018186 54.1128 1.509e-11 ***
# # Upper Limit:(Intercept)  0.1148849  0.0037802 30.3916 1.492e-09 ***
# # ED50:(Intercept)         0.9199369  1.1883595  0.7741    0.4611
# # ---
# # Signif. codes:  0 '***' 0.001 '**' 0.01 '*' 0.05 '.' 0.1 ' ' 1
# #
# # Residual standard error:
# #
# #  0.002908608 (8 degrees of freedom)
```

```
model.LL4_rep4_209 <- drm(YL1.Amean/BL1.Amean ~ dose, data =
subset(dat_sumGp_overlaydata_209,
    replicate == "4"), fct = LL.4(names = c("Slope", "Lower Limit", "Upper
Limit",
    "ED50")))
plot(model.LL4_rep4_209, type = "all", col = "dark green", lty = 1, lwd = 2,
xlab = "Extracellular IAA concentration (uM)",
    ylab = "Red/Green Fluorescent Ratio")
plot(model.LL4_rep4_209, broken = TRUE, type = "confidence", col = "yellow",
add = TRUE)
```

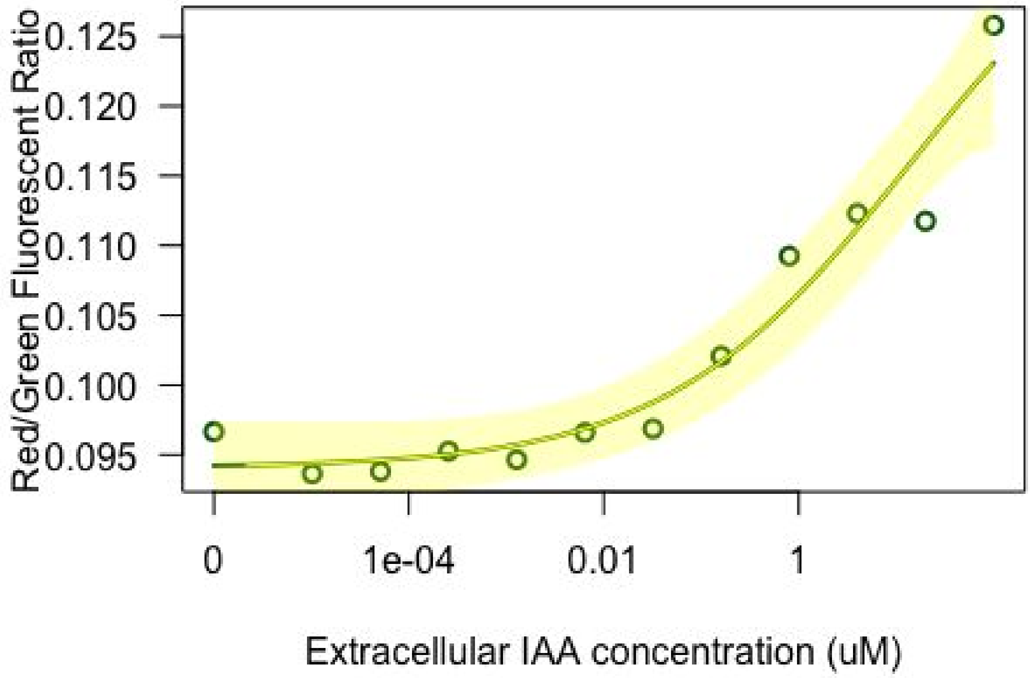

```
print(summary(model.LL4_rep4_209))
# #
# # Model fitted: Log-logistic (ED50 as parameter) (4 parms)
# #
# # Parameter estimates:
# #
# #                              Estimate Std. Error t-value   p-value
# # Slope:(Intercept)          -0.3484214  0.1076460 -3.2367   0.01194 *
# # Lower Limit:(Intercept)     0.0940801  0.0015034 62.5773 4.728e-12 ***
# # Upper Limit:(Intercept)     0.1376317  0.0174428  7.8904 4.822e-05 ***
# # ED50:(Intercept)           13.8165097 33.8084173  0.4087   0.69350
# # ---
# # Signif. codes:  0 '***' 0.001 '**' 0.01 '*' 0.05 '.' 0.1 ' ' 1
# #
# # Residual standard error:
# #
# #  0.002812448 (8 degrees of freedom)


model.LL4_all_209 <- drm(YL1.Amean/BL1.Amean ~ dose, data =
dat_sumGp_overlaydata_209,
    fct = LL.4(names = c("Slope", "Lower Limit", "Upper Limit", "ED50")))
plot(model.LL4_all_209, type = "all", col = "black", lty = 1, lwd = 3)
plot(model.LL4_all_209, broken = TRUE, col = "black", add = TRUE)
plot(model.LL4_all_209, broken = TRUE, type = "confidence", col = "black",
add = TRUE)
print(summary(model.LL4_all_209))
# #
# # Model fitted: Log-logistic (ED50 as parameter) (4 parms)
# #
# # Parameter estimates:
# #
# #                              Estimate Std. Error t-value   p-value
# # Slope:(Intercept)          -0.3420811  0.0895735 -3.8190 0.0004352 ***
# # Lower Limit:(Intercept)     0.0973731  0.0014017 69.4690 < 2.2e-16 ***
# # Upper Limit:(Intercept)     0.1337956  0.0109329 12.2379 1.971e-15 ***
# # ED50:(Intercept)           10.9140480 20.3969607  0.5351 0.5954150
# # ---
# # Signif. codes:  0 '***' 0.001 '**' 0.01 '*' 0.05 '.' 0.1 ' ' 1
# #
# # Residual standard error:
# #
# #  0.004904408 (42 degrees of freedom)

replicate1_209 <- drm(YL1.Amean/BL1.Amean ~ dose, data =
subset(dat_sumGp_overlaydata_209,
    replicate == "1"), fct = LL.4())
replicate2_209 <- drm(YL1.Amean/BL1.Amean ~ dose, data =
```

```
subset(dat_sumGp_overlaydata_209,
    replicate == "2"), fct = LL.4())
replicate3_209 <- drm(YL1.Amean/BL1.Amean ~ dose, data =
subset(dat_sumGp_overlaydata_209,
    replicate == "3"), fct = LL.4())
replicate4_209 <- drm(YL1.Amean/BL1.Amean ~ dose, data =
subset(dat_sumGp_overlaydata_209,
    replicate == "4"), fct = LL.4())

plot(replicate1_209, broken = TRUE, add = TRUE, type = "none", col = "red",
lty = 2)
plot(replicate2_209, broken = TRUE, add = TRUE, type = "none", col = "blue",
lty = 2)
plot(replicate3_209, broken = TRUE, add = TRUE, type = "none", col = "dark
green",
    lty = 2)
plot(replicate4_209, broken = TRUE, add = TRUE, type = "none", col =
"yellow", lty = 2)
```

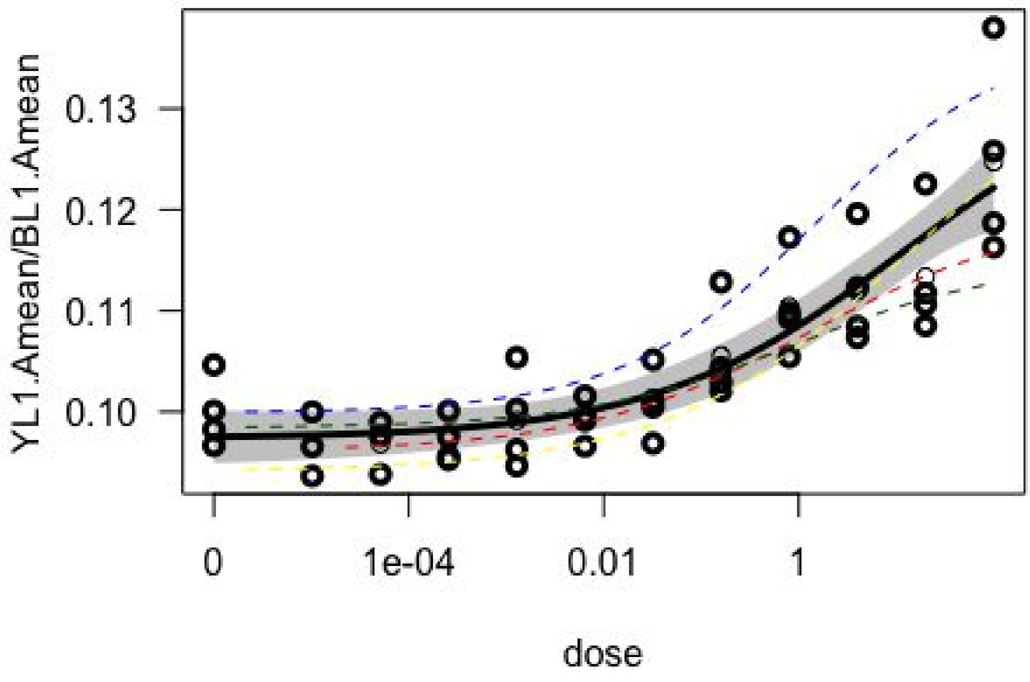

Figure 6A and 6B: Plot dose-response curve with ggplot

```
pm210 <- expand.grid(treatment = exp(seq(log(1e-05), log(100), length =
```

```
1000)))
pm210 <- cbind(pm210, predict(model.LL4_all_210, newdata = pm210, interval =
"confidence"))  #m2all = model of 210 data

plot210_dose <- ggplot(plate_all_210_sum, aes(x = dose, y =
YL1.Amean/BL1.Amean)) +
    scale_x_log10() + ylab("mScarlet-I/Venus") + xlab("Auxin Treatment (µM)")
+
    scale_x_log10() + scale_color_viridis_c(option = "C") +
geom_point(aes(color = replicate),
    size = 1, alpha = 0.4) + ylim(0.035, 0.12) + theme_classic() +
theme(legend.position = "none",
    axis.title.y = element_text(size = 12, face = "bold"), axis.title.x =
element_text(size = 12,
        face = "bold"), axis.text = element_text(size = 10), legend.title =
element_text(size = 10,
        face = "bold"), legend.text = element_text(size = 10, face = "bold"))
+ geom_ribbon(data = pm210,
    aes(x = treatment, y = Prediction, ymin = Lower, ymax = Upper), alpha =
0.6) +
    geom_line(data = pm210, aes(x = treatment, y = Prediction), size = 1.2) +
ggtitle("AFB2 biosensor") +
    ggeasy::easy_center_title() + ylim(0.03, 0.1) + scale_y_continuous(breaks
= seq(0,
    0.125, 0.025))
# # Scale for x is already present.
# # Adding another scale for x, which will replace the existing scale.
# # Scale for y is already present.
# # Adding another scale for y, which will replace the existing scale.
# # Scale for y is already present.
# # Adding another scale for y, which will replace the existing scale.


plot210_dose
```

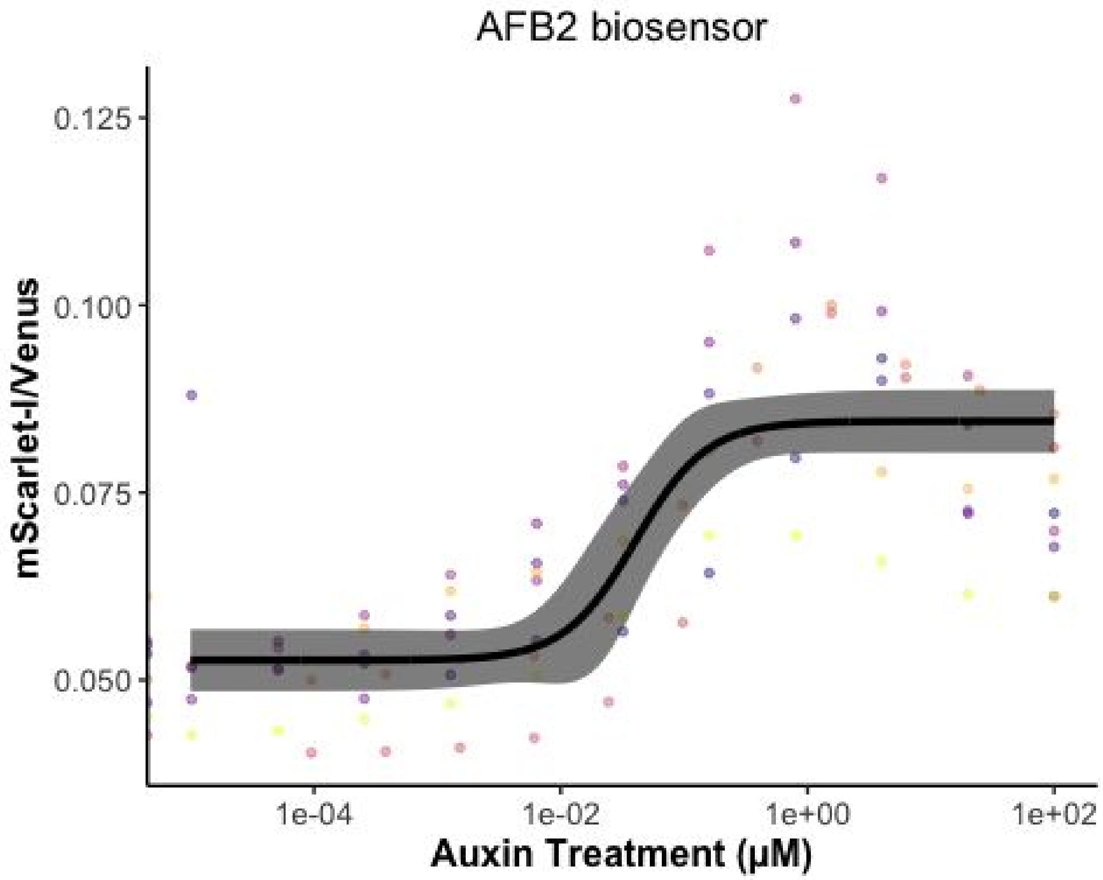


```
pm209 <- expand.grid(treatment = exp(seq(log(1e-05), log(100), length =
1000)))
pm209 <- cbind(pm209, predict(model.LL4_all_209, newdata = pm209, interval =
"confidence"))



plot209_dose <- ggplot(dat_sumGp_overlaydata_209, aes(x = dose, y =
YL1.Amean/BL1.Amean)) +
    scale_x_log10() + ylab("mScarlet-I/Venus") + xlab("Auxin Treatment (µM)")
+
    scale_x_log10() + scale_color_viridis_c(option = "C") +
geom_point(aes(color = replicate),
    size = 1, alpha = 0.4) + theme_classic() + theme(legend.position =
"none", axis.title.y = element_text(size = 12,
    face = "bold"), axis.title.x = element_text(size = 12, face = "bold"),
axis.text = element_text(size = 10),
    legend.title = element_text(size = 10, face = "bold"), legend.text =
element_text(size = 10,
        face = "bold")) + geom_ribbon(data = pm209, aes(x = treatment, y =
Prediction,
    ymin = Lower, ymax = Upper), alpha = 0.6) + geom_line(data = pm209, aes(x
```

```
= treatment,
    y = Prediction), size = 1.2) + ggtitle("TIR1 biosensor") +
ggeasy::easy_center_title() +
    ylim(0.095, 0.125)
# # Scale for x is already present.
# # Adding another scale for x, which will replace the existing scale.


plot209_dose
```

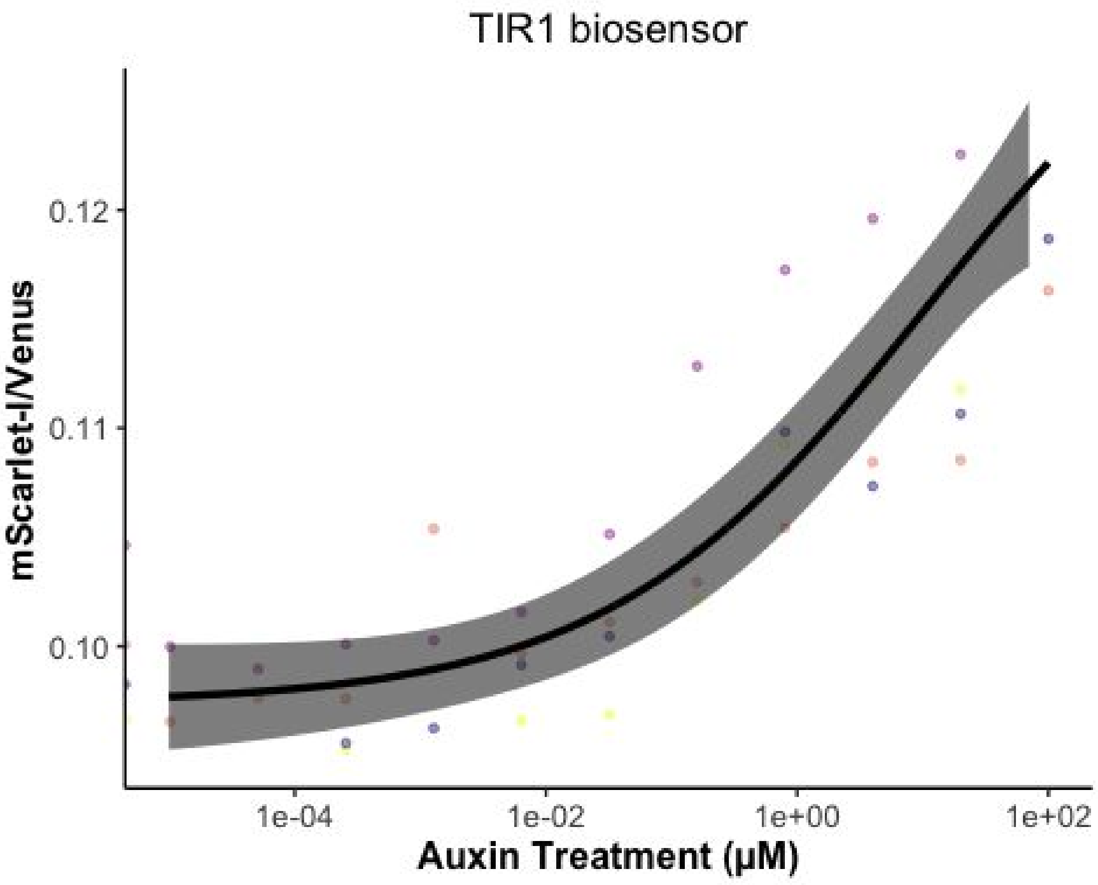


```
# grid.arrange(plot210_dose, plot209_dose, nrow=2, ncol=2)

DRAplots209_210 <- ggarrange(plot210_dose, plot209_dose, nrow = 1, ncol = 2,
labels = c("A",
    "B"))
DRAplots209_210
```

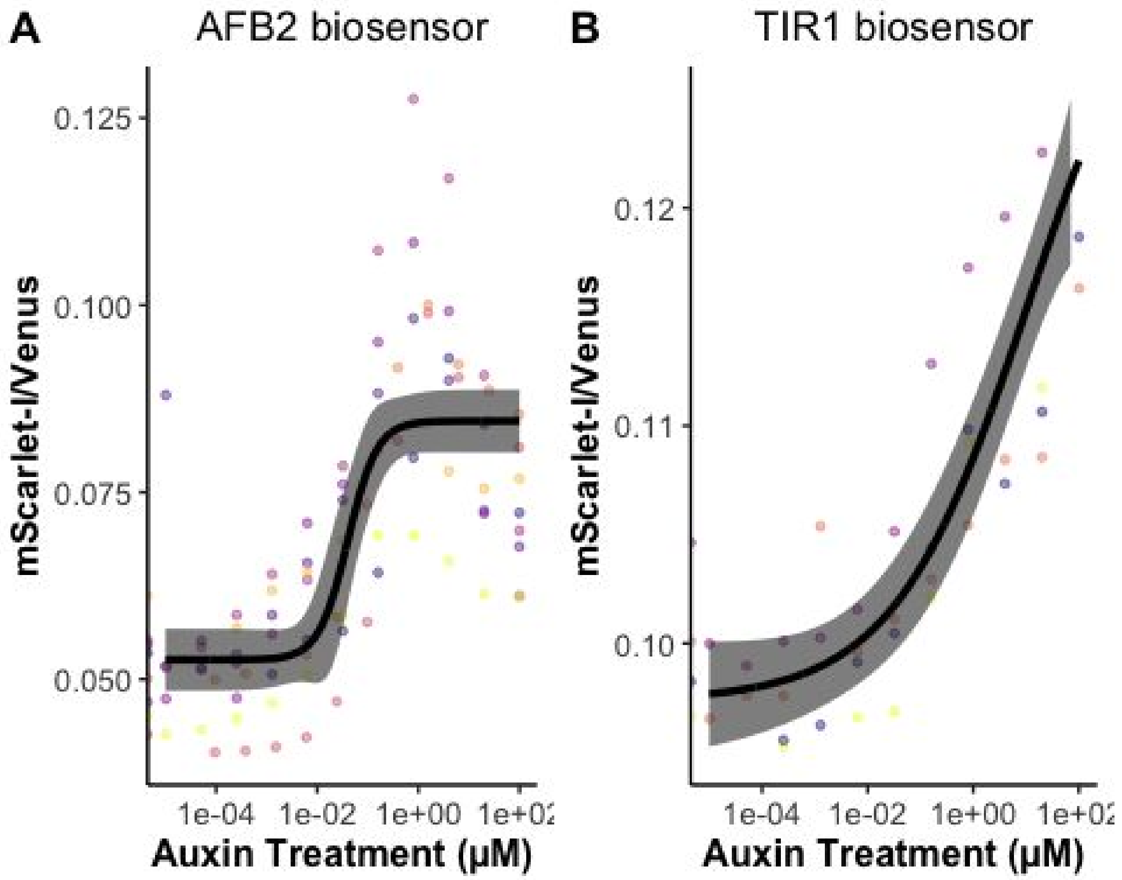


```
# ggsave('DRAplots209_210.png', width = 8, height = 3)
```

Figure 5A and Supporting Figure S3: Auxin-induced degradation and time-course assay for the biosensors in cis in multiple yeast strains.

Two random isolated colonies from each strain were tested on auxin-induced protein degradation assay. IAA working solution was added to obtain the IAA concentration at 50 uM in each culture. The assay was carried out using ThermoFisher flow cytometer.

- yWL185 (TIR1 cis in W303)
- yWL186 (AFB2 cis in W303)
- yWL209 (TIR1 cis in YPH499)
- yWL210 (AFB2 cis in YPH499)

```
plate_03112022 <- read.plateSet(path = "Data for publication/03112022_Time-
course assay/10readings/onlyPatstrains/",
    pattern = "TCA*")

annotation <- createAnnotation(yourFlowSet = plate_03112022)
write.csv(annotation, "Data for publication/03112022_Time-course
assay/03112022_Time-course assay_10platesPatStrains2.csv")
```

```
annotation <- read.csv("Data for publication/03112022_Time-course
assay/03112022_Time-course assay_10platesPatStrains2.csv")
aplate_03112022 <- annotateFlowSet(yourFlowSet = plate_03112022,
annotation_df = annotation,
    mergeBy = "name")
head(rownames(pData(aplate_03112022)))
# # [1] "103112022-Pat-TCA01_Time-course assay_Auxin_yWL185-C1.fcs"
# # [2] "103112022-Pat-TCA01_Time-course assay_Auxin_yWL185-C2.fcs"
# # [3] "103112022-Pat-TCA01_Time-course assay_Auxin_yWL186-C1.fcs"
# # [4] "103112022-Pat-TCA01_Time-course assay_Auxin_yWL186-C2.fcs"
# # [5] "103112022-Pat-TCA01_Time-course assay_Auxin_yWL209-C1.fcs"
# # [6] "103112022-Pat-TCA01_Time-course assay_Auxin_yWL209-C2.fcs"
head(pData(aplate_03112022))
# #
name
# # 103112022-Pat-TCA01_Time-course assay_Auxin_yWL185-C1.fcs 103112022-Pat-
TCA01_Time-course assay_Auxin_yWL185-C1.fcs
# # 103112022-Pat-TCA01_Time-course assay_Auxin_yWL185-C2.fcs 103112022-Pat-
TCA01_Time-course assay_Auxin_yWL185-C2.fcs
# # 103112022-Pat-TCA01_Time-course assay_Auxin_yWL186-C1.fcs 103112022-Pat-
TCA01_Time-course assay_Auxin_yWL186-C1.fcs
# # 103112022-Pat-TCA01_Time-course assay_Auxin_yWL186-C2.fcs 103112022-Pat-
TCA01_Time-course assay_Auxin_yWL186-C2.fcs
# # 103112022-Pat-TCA01_Time-course assay_Auxin_yWL209-C1.fcs 103112022-Pat-
TCA01_Time-course assay_Auxin_yWL209-C1.fcs
# # 103112022-Pat-TCA01_Time-course assay_Auxin_yWL209-C2.fcs 103112022-Pat-
TCA01_Time-course assay_Auxin_yWL209-C2.fcs
# #                                                            folder X strain
# # 103112022-Pat-TCA01_Time-course assay_Auxin_yWL185-C1.fcs  TCA01 1 yWL185
# # 103112022-Pat-TCA01_Time-course assay_Auxin_yWL185-C2.fcs  TCA01 2 yWL185
# # 103112022-Pat-TCA01_Time-course assay_Auxin_yWL186-C1.fcs  TCA01 3 yWL186
# # 103112022-Pat-TCA01_Time-course assay_Auxin_yWL186-C2.fcs  TCA01 4 yWL186
# # 103112022-Pat-TCA01_Time-course assay_Auxin_yWL209-C1.fcs  TCA01 5 yWL209
# # 103112022-Pat-TCA01_Time-course assay_Auxin_yWL209-C2.fcs  TCA01 6 yWL209
# #                                                             treatment
colony
# # 103112022-Pat-TCA01_Time-course assay_Auxin_yWL185-C1.fcs 50 uM Auxin
1
# # 103112022-Pat-TCA01_Time-course assay_Auxin_yWL185-C2.fcs 50 uM Auxin
2
# # 103112022-Pat-TCA01_Time-course assay_Auxin_yWL186-C1.fcs 50 uM Auxin
1
# # 103112022-Pat-TCA01_Time-course assay_Auxin_yWL186-C2.fcs 50 uM Auxin
2
# # 103112022-Pat-TCA01_Time-course assay_Auxin_yWL209-C1.fcs 50 uM Auxin
1
# # 103112022-Pat-TCA01_Time-course assay_Auxin_yWL209-C2.fcs 50 uM Auxin
2
# #                                                             design  yeast
# # 103112022-Pat-TCA01_Time-course assay_Auxin_yWL185-C1.fcs TIR1 cis   W303
```

```
# # 103112022-Pat-TCA01_Time-course assay_Auxin_yWL185-C2.fcs TIR1 cis   W303
# # 103112022-Pat-TCA01_Time-course assay_Auxin_yWL186-C1.fcs AFB2 cis   W303
# # 103112022-Pat-TCA01_Time-course assay_Auxin_yWL186-C2.fcs AFB2 cis   W303
# # 103112022-Pat-TCA01_Time-course assay_Auxin_yWL209-C1.fcs TIR1 cis YPH499
# # 103112022-Pat-TCA01_Time-course assay_Auxin_yWL209-C2.fcs TIR1 cis YPH499
# #
design_yeast
# # 103112022-Pat-TCA01_Time-course assay_Auxin_yWL185-C1.fcs   TIR1 dual-
fusion (W303)
# # 103112022-Pat-TCA01_Time-course assay_Auxin_yWL185-C2.fcs   TIR1 dual-
fusion (W303)
# # 103112022-Pat-TCA01_Time-course assay_Auxin_yWL186-C1.fcs   AFB2 dual-
fusion (W303)
# # 103112022-Pat-TCA01_Time-course assay_Auxin_yWL186-C2.fcs   AFB2 dual-
fusion (W303)
# # 103112022-Pat-TCA01_Time-course assay_Auxin_yWL209-C1.fcs TIR1 dual-
fusion (YPH499)
# # 103112022-Pat-TCA01_Time-course assay_Auxin_yWL209-C2.fcs TIR1 dual-
fusion (YPH499)
# #                                                              before_after
reading
# # 103112022-Pat-TCA01_Time-course assay_Auxin_yWL185-C1.fcs       before
1
# # 103112022-Pat-TCA01_Time-course assay_Auxin_yWL185-C2.fcs       before
1
# # 103112022-Pat-TCA01_Time-course assay_Auxin_yWL186-C1.fcs       before
1
# # 103112022-Pat-TCA01_Time-course assay_Auxin_yWL186-C2.fcs       before
1
# # 103112022-Pat-TCA01_Time-course assay_Auxin_yWL209-C1.fcs       before
1
# # 103112022-Pat-TCA01_Time-course assay_Auxin_yWL209-C2.fcs       before
1
# #                                                              data_selected
# # 103112022-Pat-TCA01_Time-course assay_Auxin_yWL185-C1.fcs            no
# # 103112022-Pat-TCA01_Time-course assay_Auxin_yWL185-C2.fcs            no
# # 103112022-Pat-TCA01_Time-course assay_Auxin_yWL186-C1.fcs            no
# # 103112022-Pat-TCA01_Time-course assay_Auxin_yWL186-C2.fcs            no
# # 103112022-Pat-TCA01_Time-course assay_Auxin_yWL209-C1.fcs            no
# # 103112022-Pat-TCA01_Time-course assay_Auxin_yWL209-C2.fcs            no

plate_03112022_sum <- summarizeFlow(aplate_03112022, channel = c("BL1.A",
"YL1.A"),
    gated = TRUE)
# # [1] "Summarizing all events..."

plate_03112022_sum$treatment <- factor(plate_03112022_sum$treatment, levels =
c("Control",
    "50 uM Auxin"))
```

```
plate_03112022_sum <- plate_03112022_sum %>%
    mutate(background_p = case_when(strain %in% c("yWL185", "yWL186") ~
"W303", strain %in%
        c("yWL209", "yWL210") ~ "YPH499"), receptor_p = case_when(strain %in%
c("yWL185",
        "yWL209") ~ "TIR1", strain %in% c("yWL186", "yWL210") ~ "AFB2"))

# The time auxin addition is equal to time zero
time0 <- "303112022-Pat-TCA03_Time-course assay_Auxin_yWL185-C1.fcs"
# or whatever well was being read when auxin was added

plate_03112022_sum$time <- plate_03112022_sum$btime -
plate_03112022_sum[[which(plate_03112022_sum$name ==
    time0), "btime"]]
# single bracket -->extracting the all name, 2 brackets extract just single
# 'value' or 'values'

# To normalize data

plate_03112022_sum <- plate_03112022_sum %>%
    mutate(ratio = BL1.Amean/YL1.Amean) %>%
    group_by(background_p, receptor_p) %>%
    mutate(normalizedratio = ratio/mean(ratio))

unnorm_ratio <- ggplot(plate_03112022_sum, aes(x = time, y =
BL1.Amean/YL1.Amean,
    group = interaction(factor(colony), factor(treatment)), color =
treatment)) +
    geom_line(aes(color = treatment), size = 1.5) + geom_point(aes(color =
treatment),
    size = 2.5) + scale_color_manual(values = c("#b8627db2", "#6b4596b2")) +
scale_fill_manual(values = c("#b8627db2",
    "#6b4596b2")) + xlab("Time post treatment (min)") + ylab("Venus/mScarlet-
I") +
    facet_grid(receptor_p ~ background_p) + theme_classic() +
theme(legend.position = "bottom",
    axis.title.y = element_text(size = 12, face = "bold"), axis.title.x =
element_text(size = 12,
        face = "bold"), axis.text = element_text(size = 10), legend.title =
element_text(size = 10,
        face = "bold"), legend.text = element_text(size = 10))
unnorm_ratio
```

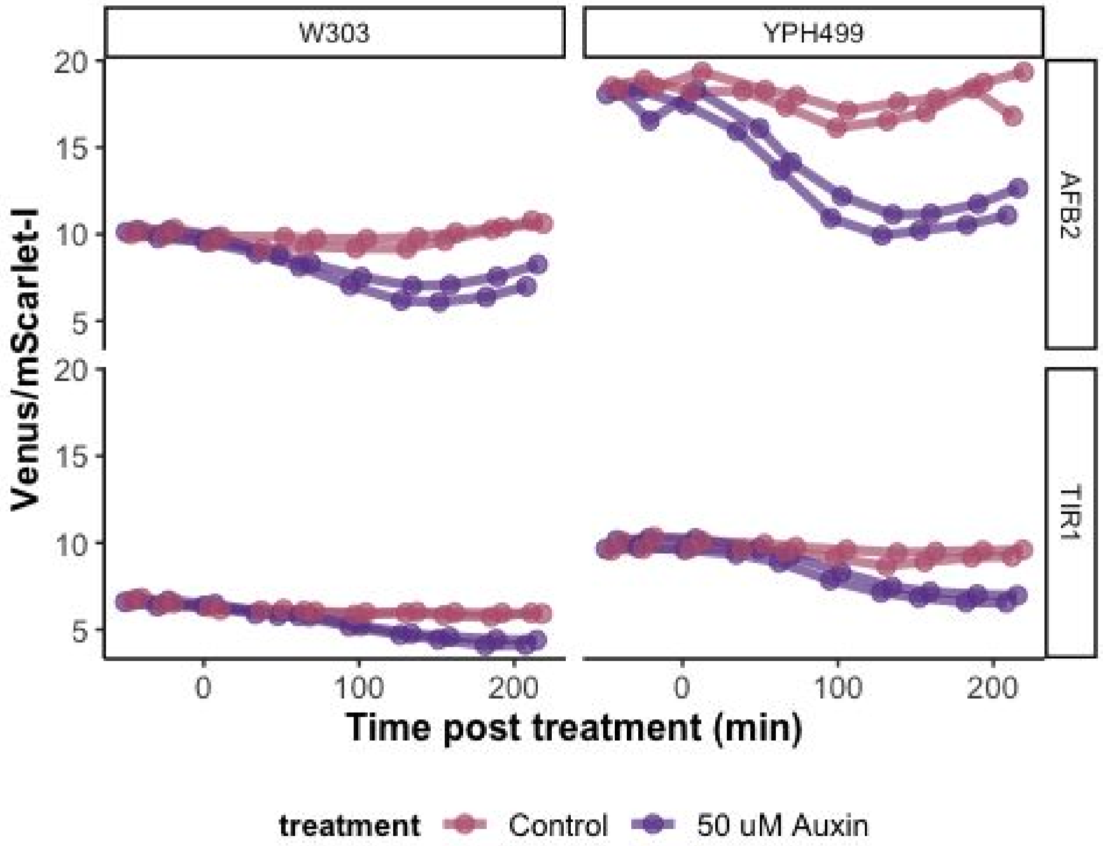


```
# ggsave('unnorm_ratio.png', width = 4, height = 3.5)



norm_ratio <- ggplot(plate_03112022_sum, aes(x = time, y = normalizedratio,
group = interaction(factor(colony),
    factor(treatment)), color = treatment)) + geom_line(aes(color =
treatment), size = 1.5,
    alpha = 0.7) + geom_point(aes(color = treatment), size = 2.5) +
scale_color_manual(values = c("#b8627dff",
    "#6b4596ff")) + scale_fill_manual(values = c("#b8627dff", "#6b4596ff")) +
xlab("Time post treatment (min)") +
    ylab("Normalized Venus/mScarlet-I") + facet_grid(receptor_p ~
background_p) +
    theme_classic() + theme(legend.position = "bottom", axis.title.y =
element_text(size = 12,
    face = "bold"), axis.title.x = element_text(size = 12, face = "bold"),
axis.text = element_text(size = 10),
    legend.title = element_text(size = 10, face = "bold"), legend.text =
element_text(size = 10)) +
    labs(color = "Treatment")
```

```
norm_ratio
```

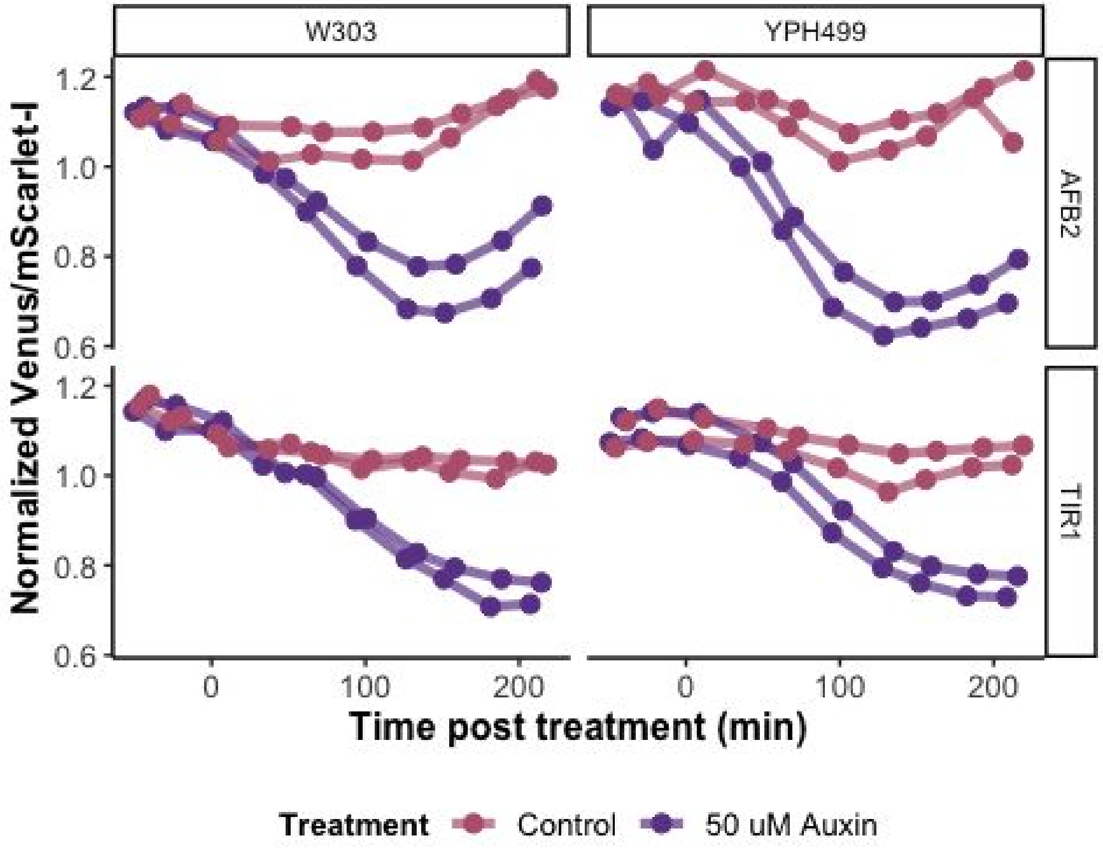


```
# ggsave('norm_ratio.png', width = 4, height = 3.5)


timecourse_plot1 <- ggarrange(unnorm_ratio, norm_ratio, nrow = 1, ncol = 2,
widths = c(1,
    1), labels = c("A", "B"), common.legend = TRUE)

timecourse_plot1
```

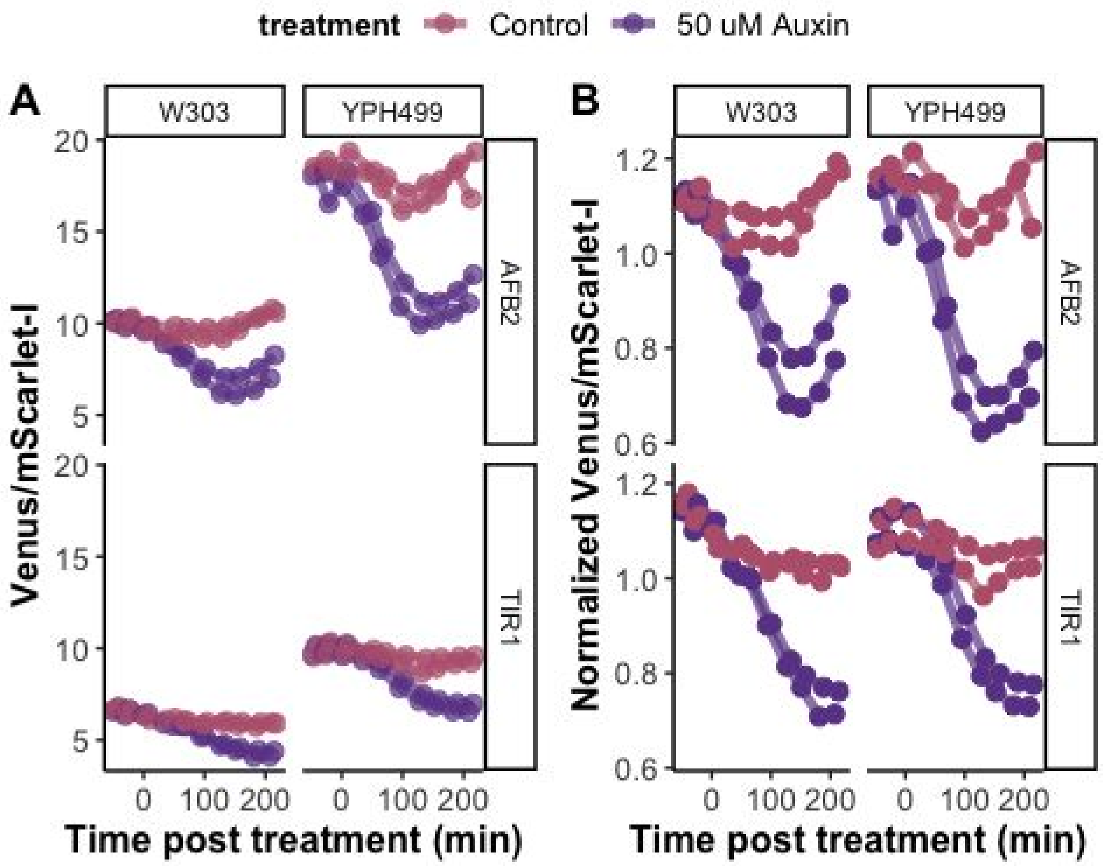


```
# ggsave('timecourse_plot1.png', width = 8, height = 4)
# ggsave('Supplement2.png', width = 8, height = 4)
```

Supporting Figure S2: CV plots

```
plate_03112022 <- read.plateSet(path = "Data for publication/03112022_Time-
course assay/10readings/onlyPatstrains/",
    pattern = "TCA*")

annotation <- createAnnotation(yourFlowSet = plate_03112022)
write.csv(annotation, "/Users/patchaisupa/Google Drive/Shared
drives/PlantSynBioLab/Pat/Experiments/Time course assays/03112022_Time-course
assay/03112022_Time-course assay_10platesPatStrains2.csv")

annotation <- read.csv("Data for publication/03112022_Time-course
assay/03112022_Time-course assay_10platesPatStrains2.csv")
aplate_03112022 <- annotateFlowSet(yourFlowSet = plate_03112022,
annotation_df = annotation,
    mergeBy = "name")
head(rownames(pData(aplate_03112022)))
# # [1] "103112022-Pat-TCA01_Time-course assay_Auxin_yWL185-C1.fcs"
# # [2] "103112022-Pat-TCA01_Time-course assay_Auxin_yWL185-C2.fcs"
# # [3] "103112022-Pat-TCA01_Time-course assay_Auxin_yWL186-C1.fcs"
```

```
# # [4] "103112022-Pat-TCA01_Time-course assay_Auxin_yWL186-C2.fcs"
# # [5] "103112022-Pat-TCA01_Time-course assay_Auxin_yWL209-C1.fcs"
# # [6] "103112022-Pat-TCA01_Time-course assay_Auxin_yWL209-C2.fcs"
head(pData(aplate_03112022))
# #
name
# # 103112022-Pat-TCA01_Time-course assay_Auxin_yWL185-C1.fcs 103112022-Pat-
TCA01_Time-course assay_Auxin_yWL185-C1.fcs
# # 103112022-Pat-TCA01_Time-course assay_Auxin_yWL185-C2.fcs 103112022-Pat-
TCA01_Time-course assay_Auxin_yWL185-C2.fcs
# # 103112022-Pat-TCA01_Time-course assay_Auxin_yWL186-C1.fcs 103112022-Pat-
TCA01_Time-course assay_Auxin_yWL186-C1.fcs
# # 103112022-Pat-TCA01_Time-course assay_Auxin_yWL186-C2.fcs 103112022-Pat-
TCA01_Time-course assay_Auxin_yWL186-C2.fcs
# # 103112022-Pat-TCA01_Time-course assay_Auxin_yWL209-C1.fcs 103112022-Pat-
TCA01_Time-course assay_Auxin_yWL209-C1.fcs
# # 103112022-Pat-TCA01_Time-course assay_Auxin_yWL209-C2.fcs 103112022-Pat-
TCA01_Time-course assay_Auxin_yWL209-C2.fcs
# #                                                            folder X strain
# # 103112022-Pat-TCA01_Time-course assay_Auxin_yWL185-C1.fcs  TCA01 1 yWL185
# # 103112022-Pat-TCA01_Time-course assay_Auxin_yWL185-C2.fcs  TCA01 2 yWL185
# # 103112022-Pat-TCA01_Time-course assay_Auxin_yWL186-C1.fcs  TCA01 3 yWL186
# # 103112022-Pat-TCA01_Time-course assay_Auxin_yWL186-C2.fcs  TCA01 4 yWL186
# # 103112022-Pat-TCA01_Time-course assay_Auxin_yWL209-C1.fcs  TCA01 5 yWL209
# # 103112022-Pat-TCA01_Time-course assay_Auxin_yWL209-C2.fcs  TCA01 6 yWL209
# #                                                             treatment
colony
# # 103112022-Pat-TCA01_Time-course assay_Auxin_yWL185-C1.fcs 50 uM Auxin
1
# # 103112022-Pat-TCA01_Time-course assay_Auxin_yWL185-C2.fcs 50 uM Auxin
2
# # 103112022-Pat-TCA01_Time-course assay_Auxin_yWL186-C1.fcs 50 uM Auxin
1
# # 103112022-Pat-TCA01_Time-course assay_Auxin_yWL186-C2.fcs 50 uM Auxin
2
# # 103112022-Pat-TCA01_Time-course assay_Auxin_yWL209-C1.fcs 50 uM Auxin
1
# # 103112022-Pat-TCA01_Time-course assay_Auxin_yWL209-C2.fcs 50 uM Auxin
2
# #                                                             design  yeast
# # 103112022-Pat-TCA01_Time-course assay_Auxin_yWL185-C1.fcs TIR1 cis   W303
# # 103112022-Pat-TCA01_Time-course assay_Auxin_yWL185-C2.fcs TIR1 cis   W303
# # 103112022-Pat-TCA01_Time-course assay_Auxin_yWL186-C1.fcs AFB2 cis   W303
# # 103112022-Pat-TCA01_Time-course assay_Auxin_yWL186-C2.fcs AFB2 cis   W303
# # 103112022-Pat-TCA01_Time-course assay_Auxin_yWL209-C1.fcs TIR1 cis YPH499
# # 103112022-Pat-TCA01_Time-course assay_Auxin_yWL209-C2.fcs TIR1 cis YPH499
# #
design_yeast
# # 103112022-Pat-TCA01_Time-course assay_Auxin_yWL185-C1.fcs   TIR1 dual-
fusion (W303)
```

```
# # 103112022-Pat-TCA01_Time-course assay_Auxin_yWL185-C2.fcs   TIR1 dual-
fusion (W303)
# # 103112022-Pat-TCA01_Time-course assay_Auxin_yWL186-C1.fcs   AFB2 dual-
fusion (W303)
# # 103112022-Pat-TCA01_Time-course assay_Auxin_yWL186-C2.fcs   AFB2 dual-
fusion (W303)
# # 103112022-Pat-TCA01_Time-course assay_Auxin_yWL209-C1.fcs TIR1 dual-
fusion (YPH499)
# # 103112022-Pat-TCA01_Time-course assay_Auxin_yWL209-C2.fcs TIR1 dual-
fusion (YPH499)
# #                                                             before_after
reading
# # 103112022-Pat-TCA01_Time-course assay_Auxin_yWL185-C1.fcs       before
1
# # 103112022-Pat-TCA01_Time-course assay_Auxin_yWL185-C2.fcs       before
1
# # 103112022-Pat-TCA01_Time-course assay_Auxin_yWL186-C1.fcs       before
1
# # 103112022-Pat-TCA01_Time-course assay_Auxin_yWL186-C2.fcs       before
1
# # 103112022-Pat-TCA01_Time-course assay_Auxin_yWL209-C1.fcs       before
1
# # 103112022-Pat-TCA01_Time-course assay_Auxin_yWL209-C2.fcs       before
1
# #                                                             data_selected
# # 103112022-Pat-TCA01_Time-course assay_Auxin_yWL185-C1.fcs            no
# # 103112022-Pat-TCA01_Time-course assay_Auxin_yWL185-C2.fcs            no
# # 103112022-Pat-TCA01_Time-course assay_Auxin_yWL186-C1.fcs            no
# # 103112022-Pat-TCA01_Time-course assay_Auxin_yWL186-C2.fcs            no
# # 103112022-Pat-TCA01_Time-course assay_Auxin_yWL209-C1.fcs            no
# # 103112022-Pat-TCA01_Time-course assay_Auxin_yWL209-C2.fcs            no


plate_03112022_sum <- summarizeFlow(aplate_03112022, channel = c("BL1.A",
"YL1.A"),
    gated = TRUE)
# # [1] "Summarizing all events..."


# The time auxin addition is equal to time zero
time0 <- "303112022-Pat-TCA03_Time-course assay_Auxin_yWL185-C1.fcs"
# or whatever well was being read when auxin was added

plate_03112022_sum$time <- plate_03112022_sum$btime -
plate_03112022_sum[[which(plate_03112022_sum$name ==
    time0), "btime"]]
# single bracket -->extracting the all name, 2 brackets extract just single
# 'value' or 'values'
```

```
plate_03112022_sum <- plate_03112022_sum %>%
    mutate(background_p = case_when(strain %in% c("yWL185", "yWL186") ~
"W303", strain %in%
        c("yWL209", "yWL210") ~ "YPH499"), receptor_p = case_when(strain %in%
c("yWL185",
        "yWL209") ~ "TIR1", strain %in% c("yWL186", "yWL210") ~ "AFB2"))
```

Supporting Figure S2A: yWL185 (TIR1 in cis, W303 yeast)

```
data185 <- steadyState(aplate_03112022, gated = TRUE)
# # [1] "No further gating applied."
# # [1] "Converting events..."
data185 <- subset(data185, strain == "yWL185" & name %in% c("803112022-Pat-
TCA08_Time-course assay_Auxin_yWL185-C1.fcs",
    "803112022-Pat-TCA08_Time-course assay_Control_yWL185-C1.fcs"))

cv <- function(x) return(round(sd(x)/mean(x), 2))


data185 <- subset(data185, BL1.A > 1 & YL1.A > 1)
data185$FLratio <- data185$BL1.A/data185$YL1.A
range(data185$BL1.A)
# # [1]      45 1048575
cv <- function(x) return(round(sd(x)/mean(x), 2))

data185$Venus <- data185$BL1.A/median(data185$BL1.A)
data185$mScarlet <- data185$YL1.A/median(data185$YL1.A)
data185$FLratio <- data185$BL1.A/data185$YL1.A
data185$ratio <- data185$FLratio/median(data185$FLratio)



data_long185 <- data185 %>%
    dplyr::select(treatment, Venus, mScarlet, ratio, strain) %>%
    pivot_longer(cols = c(Venus, mScarlet, ratio), names_to = "parameter",
values_to = "value") %>%
    dplyr::mutate(parameter = fct_relevel(parameter, "Venus"))

# need to also format CVs approriately for annotating

CV185 <- data185 %>%
    group_by(treatment) %>%
    dplyr::summarise(across(dplyr::where(is_double), cv)) %>%
    dplyr::select(treatment, Venus, mScarlet, ratio) %>%
    pivot_longer(cols = c(Venus, mScarlet, ratio), names_to = "parameter",
values_to = "value")

plot185 <- ggplot(data = data_long185, mapping = aes(x = value, color =
treatment)) +
```

```
    geom_density() + xlim(c(-1, 4)) + labs(x = "median normalized intensity",
color = "treatment") +
    theme_test() + geom_text(data = subset(CV185, treatment == "50 uM
Auxin"), aes(label = paste0("CV = ",
    value)), x = 2, y = 1) + geom_text(data = subset(CV185, treatment ==
"Control"),
    aes(label = paste0("CV = ", value)), x = 0, y = 1) +
scale_color_viridis_d(option = "D",
    end = 0.75, direction = -1)




venus185 <- ggplot(data185, aes(x = data185$Venus, group = treatment, fill =
treatment,
    color = treatment)) + geom_density(adjust = 1.5, alpha = 0.5) + labs(x =
"Venus",
    y = "Density") + xlim(-0.1, 2) + ylim(0, 2) + theme_classic() +
theme(legend.position = "none") +
    geom_text(data = subset(CV185, parameter == "Venus" & treatment == "50 uM
Auxin"),
        aes(label = paste0("CV = ", value)), x = 1.2, y = 1.1) +
geom_text(data = subset(CV185,
    parameter == "Venus" & treatment == "Control"), aes(label = paste0("CV =
", value)),
    x = 0.55, y = 1.5) + scale_color_manual(values = c("#f7cb44b2",
"#566573")) +
    scale_fill_manual(values = c("#f7cb44b2", "#566573"))
venus185
```

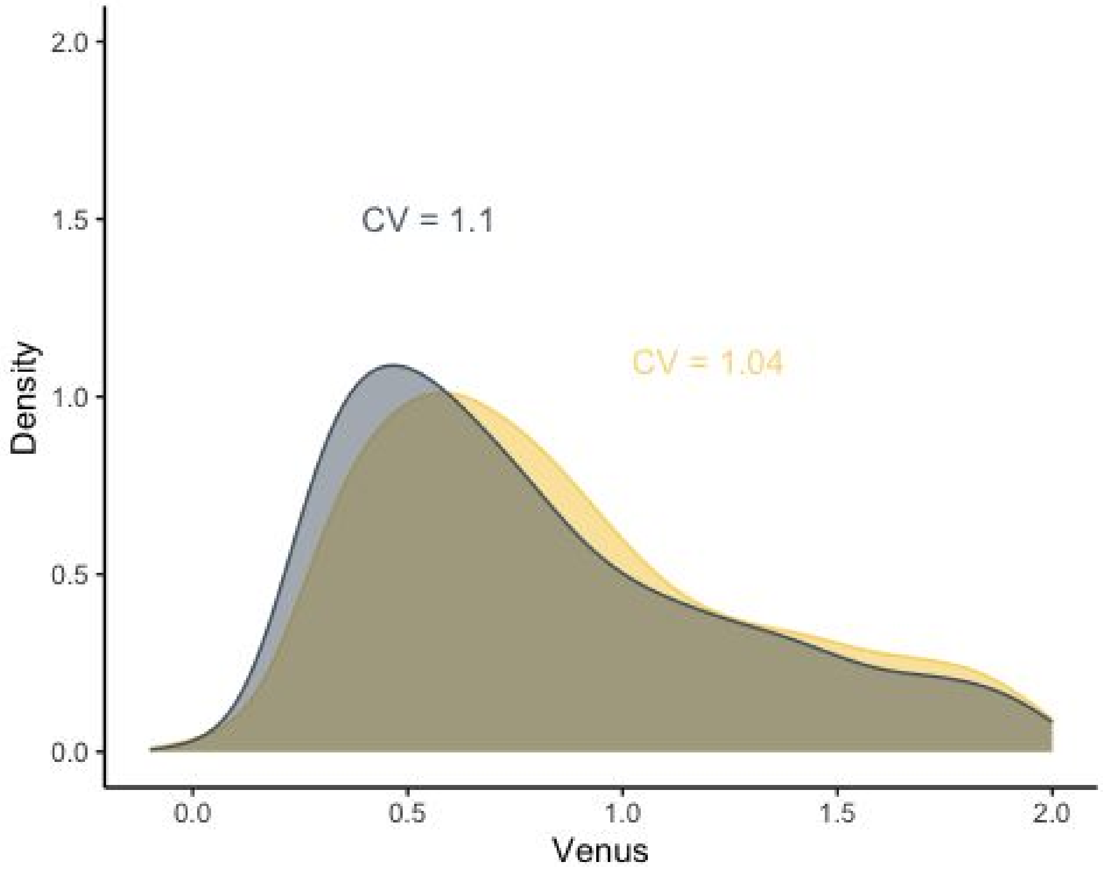


```
red185 <- ggplot(data185, aes(x = data185$mScarlet, group = treatment, fill =
treatment,
    color = treatment)) + geom_density(adjust = 1.5, alpha = 0.5) + labs(x =
"mScarlet",
    y = "Density") + xlim(-0.1, 2) + ylim(0, 2) + theme_classic() +
theme(legend.position = "none") +
    geom_text(data = subset(CV185, parameter == "mScarlet" & treatment == "50
uM Auxin"),
        aes(label = paste0("CV = ", value)), x = 1.2, y = 1.1) +
geom_text(data = subset(CV185,
    parameter == "mScarlet" & treatment == "Control"), aes(label = paste0("CV
= ",
    value)), x = 0.55, y = 1.5) + scale_color_manual(values = c("#de7065b2",
"#566573")) +
    scale_fill_manual(values = c("#de7065b2", "#566573"))
red185
```

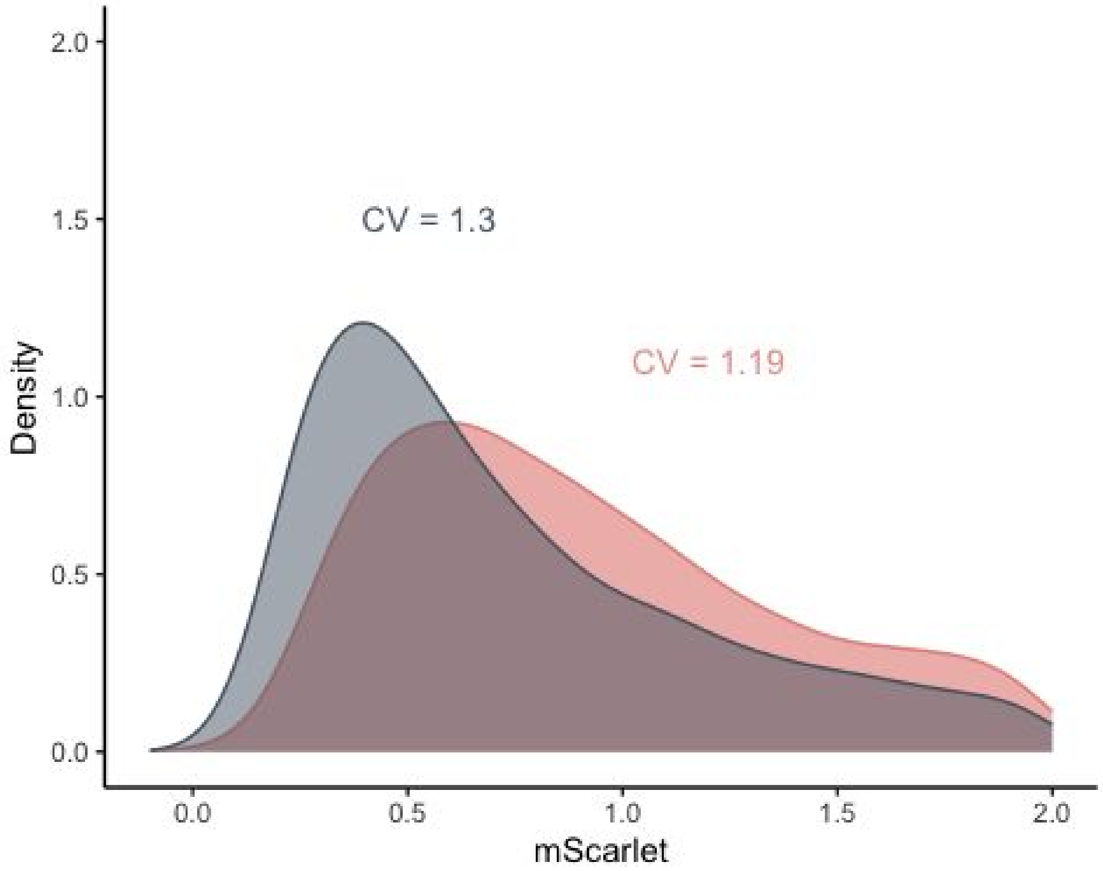


```
ratio185 <- ggplot(data185, aes(x = data185$ratio, group = treatment, fill =
treatment,
    color = treatment)) + geom_density(adjust = 1.5, alpha = 0.5) + labs(x =
"Venus/mScarlet ratio",
    y = "Density") + xlim(-0.1, 2) + ylim(0, 3.5) + theme_classic() +
theme(legend.position = "none") +
    geom_text(data = subset(CV185, parameter == "ratio" & treatment == "50 uM
Auxin"),
        aes(label = paste0("CV = ", value)), x = 0.4, y = 2) + geom_text(data
= subset(CV185,
    parameter == "ratio" & treatment == "Control"), aes(label = paste0("CV =
", value)),
    x = 1.6, y = 2) + scale_color_manual(values = c("#593d9cb2", "#566573"))
+ scale_fill_manual(values = c("#593d9cb2",
    "#566573"))

ratio185
```

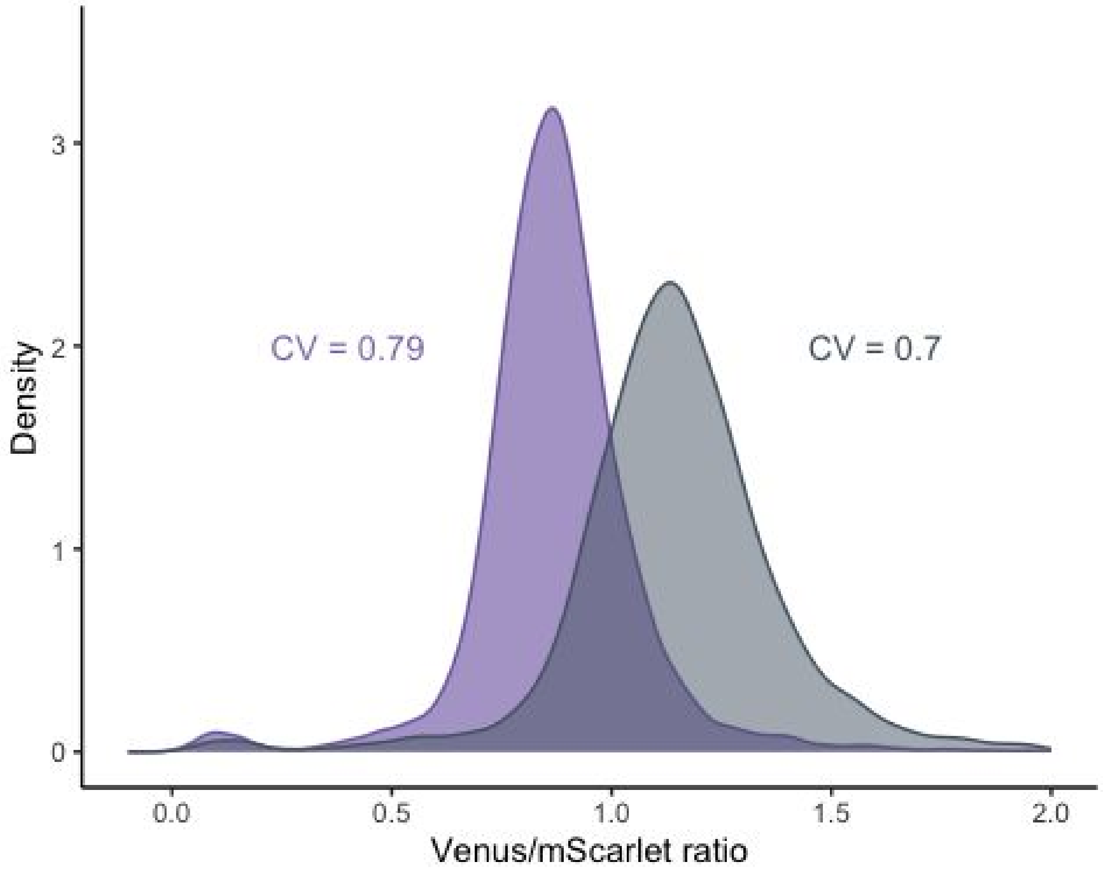


```
plot185 <- grid.arrange(venus185, red185, ratio185, nrow = 3, ncol = 1)
```

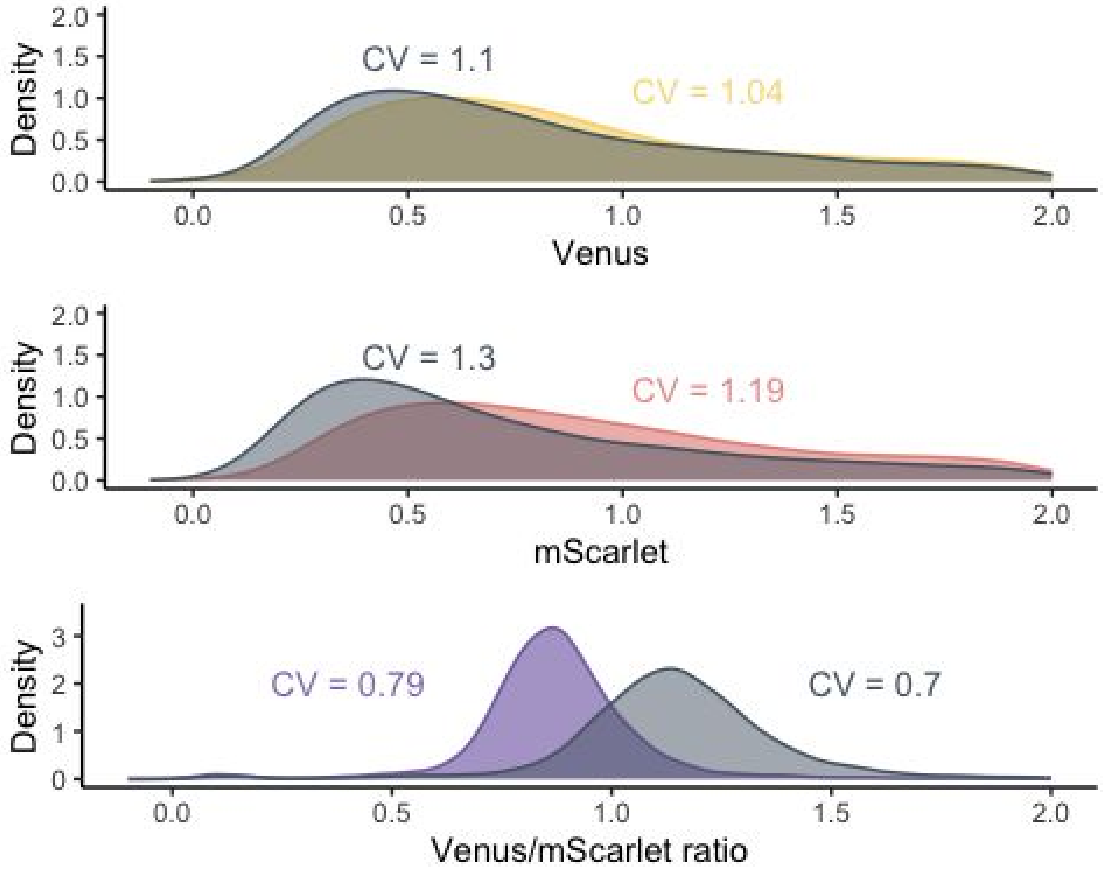


```
plot185
# # TableGrob (3 x 1) "arrange": 3 grobs
# #   z     cells    name           grob
# # 1 1 (1-1,1-1) arrange gtable[layout]
# # 2 2 (2-2,1-1) arrange gtable[layout]
# # 3 3 (3-3,1-1) arrange gtable[layout]

plot185_all <- venus185/red185/ratio185 + plot_annotation(title = "TIR1 dual-
fusion (W303)") &
    theme(plot.title = element_text(hjust = 0.5))
plot185_all
```

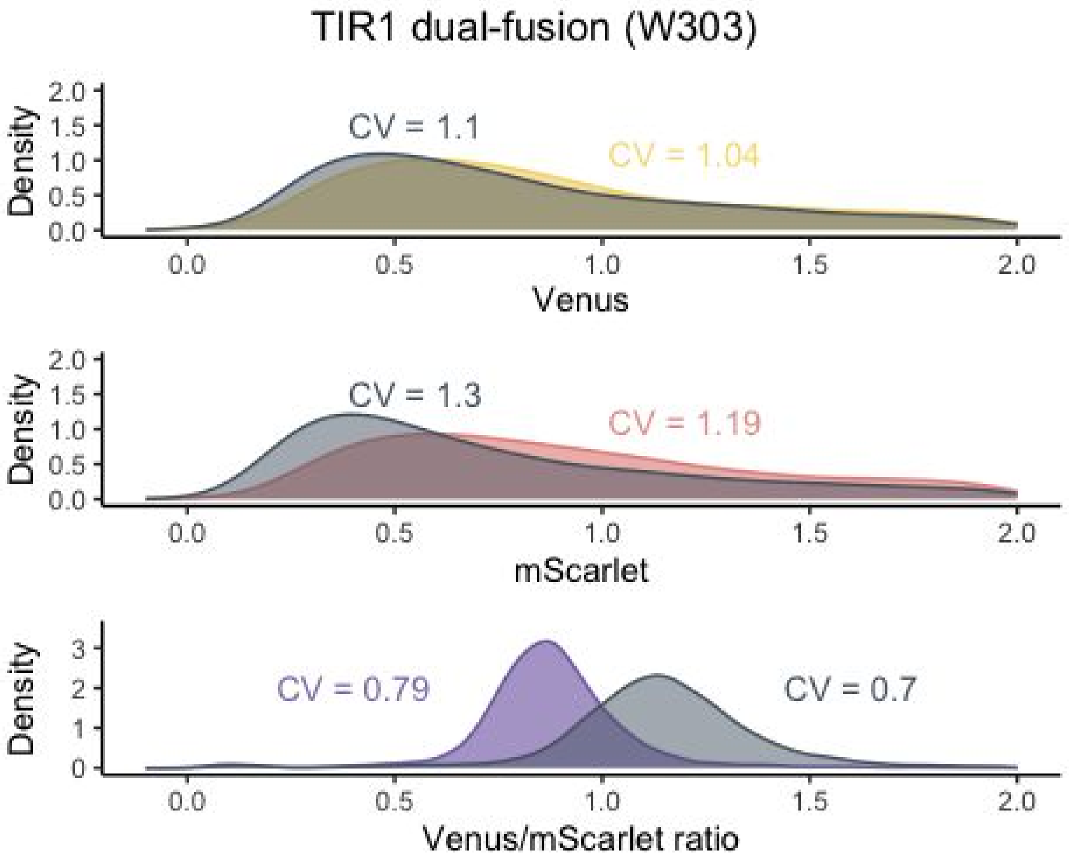


```
# ggsave('plot185_all.png', width = 6, height = 5)
```

Supporting Figure S2B: yWL186 (AFB2 in cis, W303 yeast)

```
data186 <- steadyState(aplate_03112022, gated = TRUE)
# # [1] "No further gating applied."
# # [1] "Converting events..."
data186 <- subset(data186, strain == "yWL186" & name %in% c("803112022-Pat-
TCA08_Time-course assay_Auxin_yWL186-C1.fcs",
    "803112022-Pat-TCA08_Time-course assay_Control_yWL186-C1.fcs"))

cv <- function(x) return(round(sd(x)/mean(x), 2))


data186 <- subset(data186, BL1.A > 1 & YL1.A > 1)
data186$FLratio <- data186$BL1.A/data186$YL1.A
range(data186$BL1.A)
# # [1]      25 1048575
cv <- function(x) return(round(sd(x)/mean(x), 2))

data186$Venus <- data186$BL1.A/median(data186$BL1.A)
data186$mScarlet <- data186$YL1.A/median(data186$YL1.A)
data186$FLratio <- data186$BL1.A/data186$YL1.A
```

```
data186$ratio <- data186$FLratio/median(data186$FLratio)



data_long186 <- data186 %>%
    dplyr::select(treatment, Venus, mScarlet, ratio, strain) %>%
    pivot_longer(cols = c(Venus, mScarlet, ratio), names_to = "parameter",
values_to = "value") %>%
    dplyr::mutate(parameter = fct_relevel(parameter, "Venus"))

# need to also format CVs approriately for annotating

CV186 <- data186 %>%
    group_by(treatment) %>%
    dplyr::summarise(across(dplyr::where(is_double), cv)) %>%
    dplyr::select(treatment, Venus, mScarlet, ratio) %>%
    pivot_longer(cols = c(Venus, mScarlet, ratio), names_to = "parameter",
values_to = "value")

plot186 <- ggplot(data = data_long186, mapping = aes(x = value, color =
treatment)) +
    geom_density() + xlim(c(-1, 4)) + labs(x = "median normalized intensity",
color = "treatment") +
    theme_test() + geom_text(data = subset(CV186, treatment == "50 uM
Auxin"), aes(label = paste0("CV = ",
    value)), x = 2, y = 1) + geom_text(data = subset(CV186, treatment ==
"Control"),
    aes(label = paste0("CV = ", value)), x = 0, y = 1) +
scale_color_viridis_d(option = "D",
    end = 0.75, direction = -1)


venus186 <- ggplot(data186, aes(x = data186$Venus, group = treatment, fill =
treatment,
    color = treatment)) + geom_density(adjust = 1.5, alpha = 0.5) + labs(x =
"Venus (normalized median)",
    y = "Density") + xlim(0, 2) + ylim(0, 2) + theme_classic() +
theme(legend.position = "none") +
    geom_text(data = subset(CV186, parameter == "Venus" & treatment == "50 uM
Auxin"),
        aes(label = paste0("CV = ", value)), x = 1.2, y = 1.1) +
geom_text(data = subset(CV186,
    parameter == "Venus" & treatment == "Control"), aes(label = paste0("CV =
", value)),
    x = 0.55, y = 1.5) + scale_color_manual(values = c("#f7cb44b2",
"#566573")) +
    scale_fill_manual(values = c("#f7cb44b2", "#566573"))


red186 <- ggplot(data186, aes(x = data186$mScarlet, group = treatment, fill =
```

```
treatment,
    color = treatment)) + geom_density(adjust = 1.5, alpha = 0.5) + labs(x =
"mScarlet (normalized median)",
    y = "Density") + xlim(0, 2) + ylim(0, 2) + theme_classic() +
theme(legend.position = "none") +
    geom_text(data = subset(CV186, parameter == "mScarlet" & treatment == "50
uM Auxin"),
        aes(label = paste0("CV = ", value)), x = 1.2, y = 1.1) +
geom_text(data = subset(CV186,
    parameter == "mScarlet" & treatment == "Control"), aes(label = paste0("CV
= ",
    value)), x = 0.55, y = 1.5) + scale_color_manual(values = c("#de7065b2",
"#566573")) +
    scale_fill_manual(values = c("#de7065b2", "#566573"))


ratio186 <- ggplot(data186, aes(x = data186$ratio, group = treatment, fill =
treatment,
    color = treatment)) + geom_density(adjust = 1.5, alpha = 0.5) + labs(x =
"Venus/mScarlet ratio",
    y = "Density") + xlim(0, 2) + ylim(0, 3.5) + theme_classic() +
theme(legend.position = "none") +
    geom_text(data = subset(CV186, parameter == "ratio" & treatment == "50 uM
Auxin"),
        aes(label = paste0("CV = ", value)), x = 0.2, y = 2) + geom_text(data
= subset(CV186,
    parameter == "ratio" & treatment == "Control"), aes(label = paste0("CV =
", value)),
    x = 1.3, y = 2) + scale_color_manual(values = c("#593d9cb2", "#566573"))
+ scale_fill_manual(values = c("#593d9cb2",
    "#566573"))

plot186 <- grid.arrange(venus186, red186, ratio186, nrow = 3, ncol = 1)
```

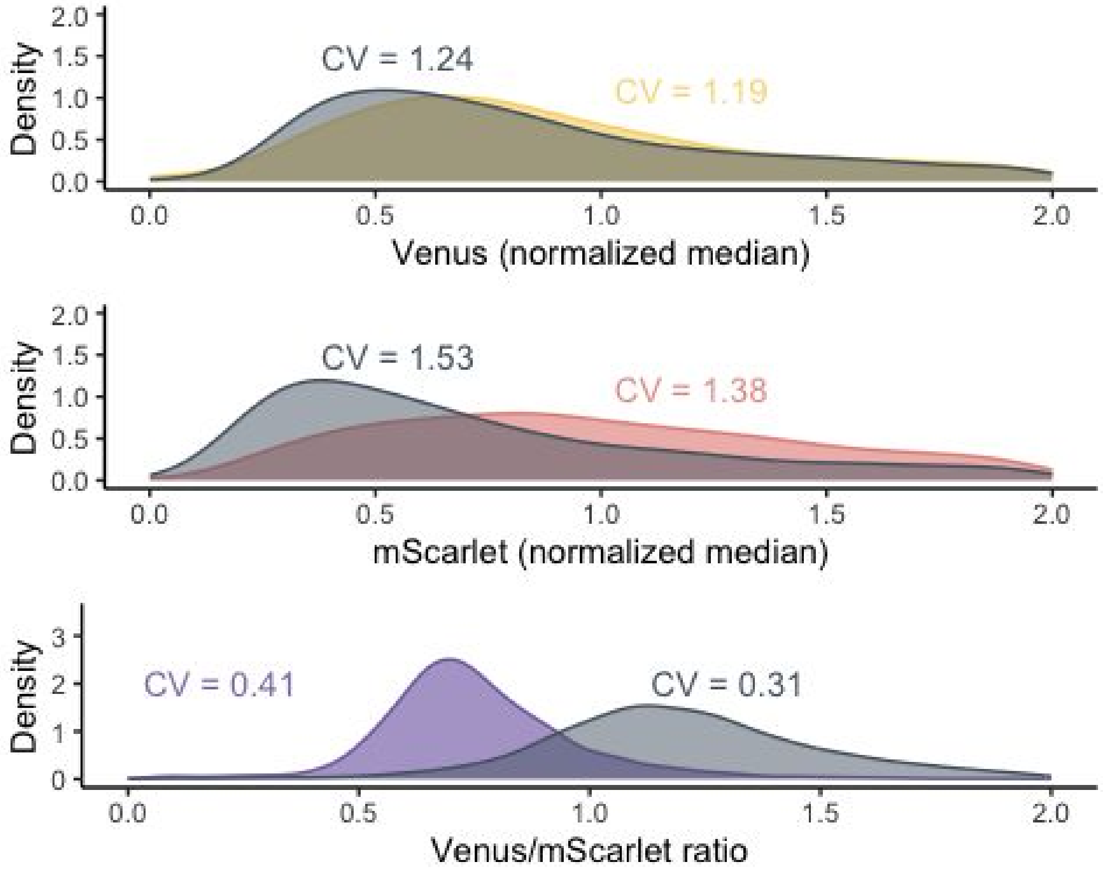


```
plot186
# # TableGrob (3 x 1) "arrange": 3 grobs
# #   z     cells    name           grob
# # 1 1 (1-1,1-1) arrange gtable[layout]
# # 2 2 (2-2,1-1) arrange gtable[layout]
# # 3 3 (3-3,1-1) arrange gtable[layout]

plot186_all <- venus186/red186/ratio186 + plot_annotation(title = "AFB2 dual-
fusion (W303)") &
    theme(plot.title = element_text(hjust = 0.5))
plot186_all
```

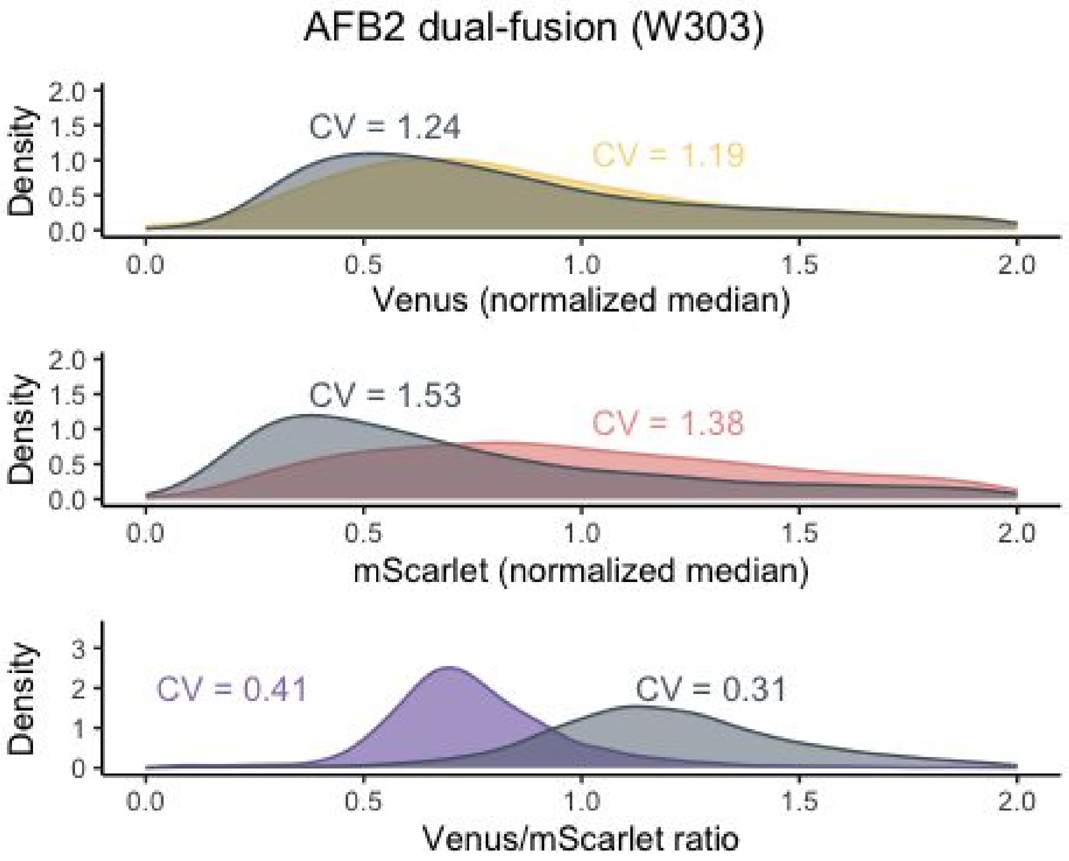


```r
# ggsave('plot186_all.png', width = 6, height = 5)
```

Supporting Figure S2C: yWL209 (TIR1 in cis, YPH499 yeast)

```r
data209 <- steadyState(aplate_03112022, gated = TRUE)
# # [1] "No further gating applied."
# # [1] "Converting events..."
data209 <- subset(data209, strain == "yWL209" & name %in% c("803112022-Pat-TCA08_Time-course assay_Auxin_yWL209-C1.fcs",
    "803112022-Pat-TCA08_Time-course assay_Control_yWL209-C1.fcs"))

cv <- function(x) return(round(sd(x)/mean(x), 2))


data209 <- subset(data209, BL1.A > 1 & YL1.A > 1)
data209$FLratio <- data209$BL1.A/data209$YL1.A
range(data209$BL1.A)
# # [1]       9 1048575
cv <- function(x) return(round(sd(x)/mean(x), 2))

data209$Venus <- data209$BL1.A/median(data209$BL1.A)
data209$mScarlet <- data209$YL1.A/median(data209$YL1.A)
```

```
data209$FLratio <- data209$BL1.A/data209$YL1.A
data209$ratio <- data209$FLratio/median(data209$FLratio)



data_long209 <- data209 %>%
    dplyr::select(treatment, Venus, mScarlet, ratio, strain) %>%
    pivot_longer(cols = c(Venus, mScarlet, ratio), names_to = "parameter",
values_to = "value") %>%
    dplyr::mutate(parameter = fct_relevel(parameter, "Venus"))

# need to also format CVs approriately for annotating

CV209 <- data209 %>%
    group_by(treatment) %>%
    dplyr::summarise(across(dplyr::where(is_double), cv)) %>%
    dplyr::select(treatment, Venus, mScarlet, ratio) %>%
    pivot_longer(cols = c(Venus, mScarlet, ratio), names_to = "parameter",
values_to = "value")

plot209 <- ggplot(data = data_long209, mapping = aes(x = value, color =
treatment)) +
    geom_density() + xlim(c(-1, 4)) + labs(x = "median normalized intensity",
color = "treatment") +
    theme_test() + geom_text(data = subset(CV209, treatment == "50 uM
Auxin"), aes(label = paste0("CV = ",
    value)), x = 1.2, y = 1) + geom_text(data = subset(CV209, treatment ==
"Control"),
    aes(label = paste0("CV = ", value)), x = 0, y = 1) +
scale_color_viridis_d(option = "D",
    end = 0.75, direction = -1)



venus209 <- ggplot(data209, aes(x = data209$Venus, group = treatment, fill =
treatment,
    color = treatment)) + geom_density(adjust = 1.5, alpha = 0.5) + labs(x =
"Venus",
    y = "Density") + xlim(0, 2) + ylim(0, 1.5) + theme_classic() +
theme(legend.position = "none") +
    geom_text(data = subset(CV209, parameter == "Venus" & treatment == "50 uM
Auxin"),
        aes(label = paste0("CV = ", value)), x = 1.2, y = 1.1) +
geom_text(data = subset(CV209,
    parameter == "Venus" & treatment == "Control"), aes(label = paste0("CV =
", value)),
    x = 0.58, y = 1.3) + scale_color_manual(values = c("#f7cb44b2",
"#566573")) +
    scale_fill_manual(values = c("#f7cb44b2", "#566573"))
```

```
red209 <- ggplot(data209, aes(x = data209$mScarlet, group = treatment, fill =
treatment,
    color = treatment)) + geom_density(adjust = 1.5, alpha = 0.5) + labs(x =
"mScarlet",
    y = "Density") + xlim(0, 2) + ylim(0, 1.5) + theme_classic() +
theme(legend.position = "none") +
    geom_text(data = subset(CV209, parameter == "mScarlet" & treatment == "50
uM Auxin"),
        aes(label = paste0("CV = ", value)), x = 1.3, y = 1) + geom_text(data
= subset(CV209,
    parameter == "mScarlet" & treatment == "Control"), aes(label = paste0("CV
= ",
    value)), x = 0.59, y = 1.3) + scale_color_manual(values = c("#de7065b2",
"#566573")) +
    scale_fill_manual(values = c("#de7065b2", "#566573"))


ratio209 <- ggplot(data209, aes(x = data209$ratio, group = treatment, fill =
treatment,
    color = treatment)) + geom_density(adjust = 1.5, alpha = 0.5) + labs(x =
"Venus/mScarlet ratio",
    y = "Density") + xlim(0, 2) + ylim(0, 3.2) + theme_classic() +
theme(legend.position = "none") +
    geom_text(data = subset(CV209, parameter == "ratio" & treatment == "50 uM
Auxin"),
        aes(label = paste0("CV = ", value)), x = 0.6, y = 3.1) +
geom_text(data = subset(CV209,
    parameter == "ratio" & treatment == "Control"), aes(label = paste0("CV =
", value)),
    x = 1.6, y = 2.3) + scale_color_manual(values = c("#593d9cb2",
"#566573")) +
    scale_fill_manual(values = c("#593d9cb2", "#566573"))


plot209 <- grid.arrange(venus209, red209, ratio209, nrow = 3, ncol = 1)
```

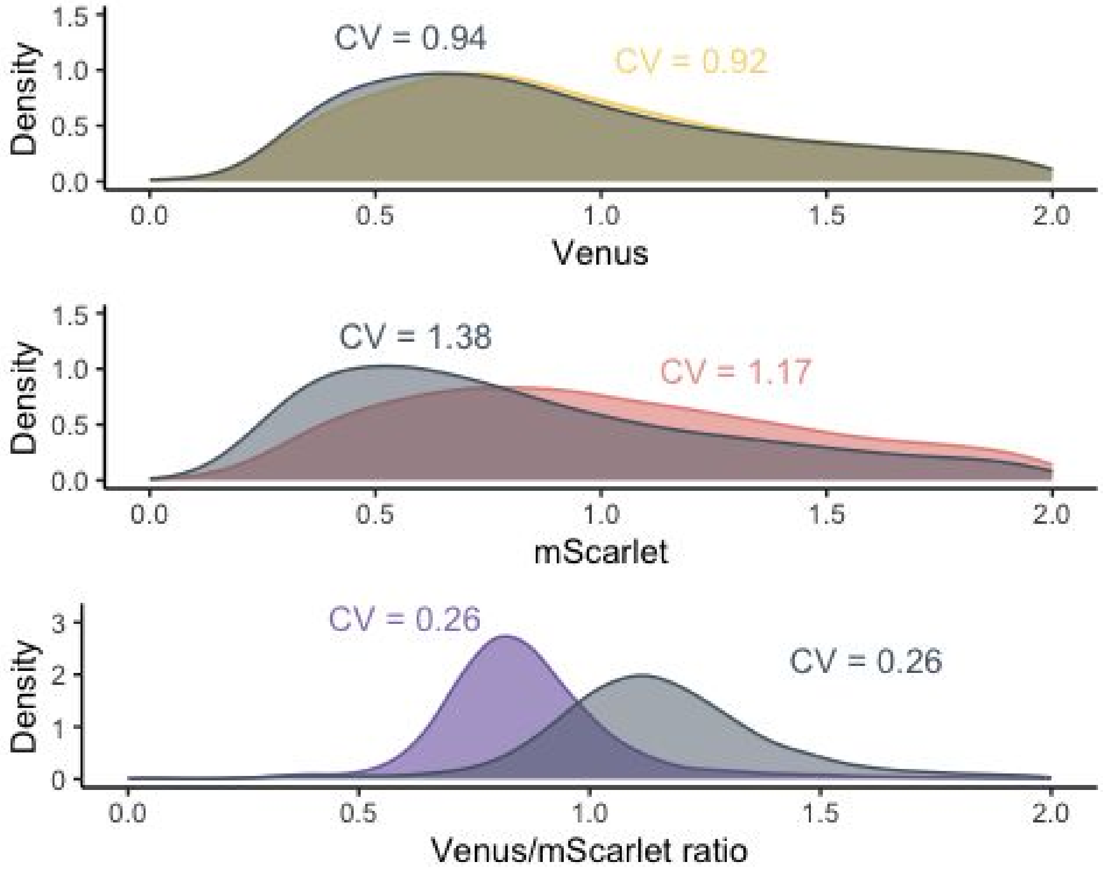


```
plot209 <- annotate_figure(plot209, top = text_grob("TIR1", color = "black",
size = 12))
plot209
```

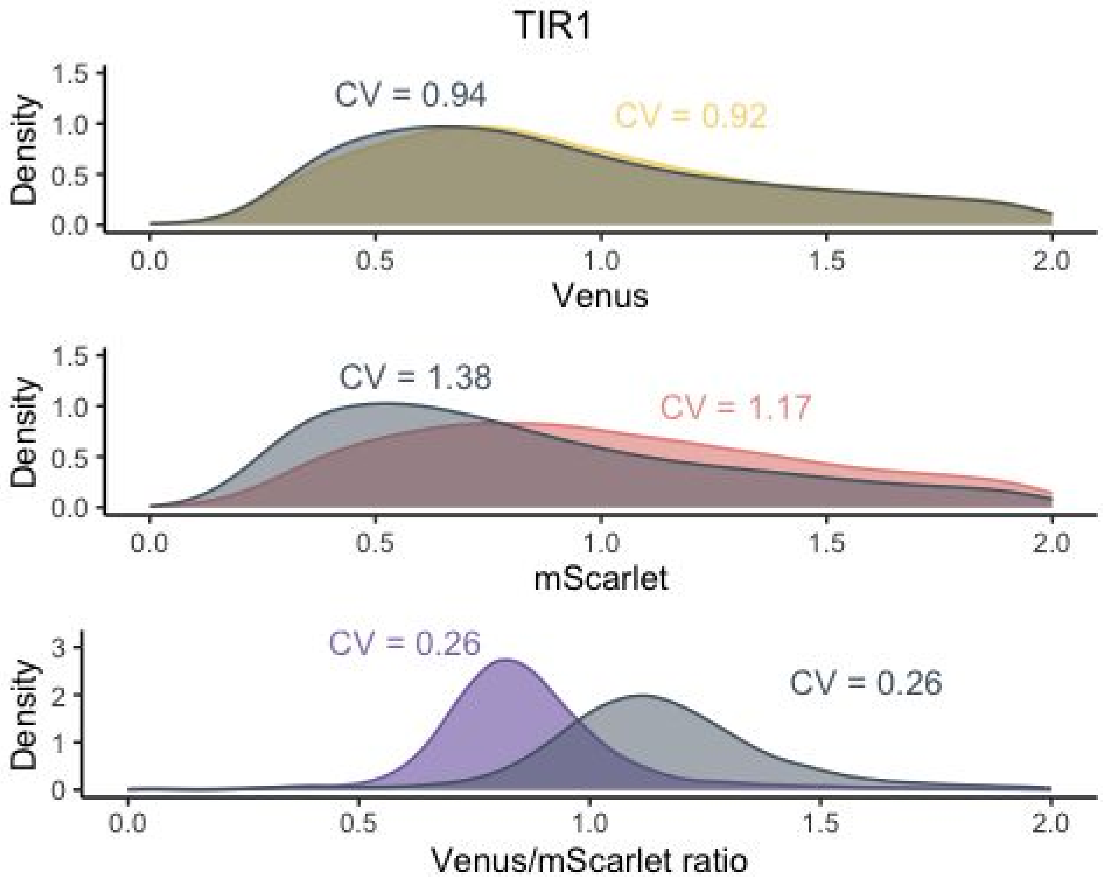


```
plot209_all <- venus209/red209/ratio209 + plot_annotation(title = "TIR1 dual-
fusion (YPH499)") &
    theme(plot.title = element_text(hjust = 0.5))
plot209_all
```

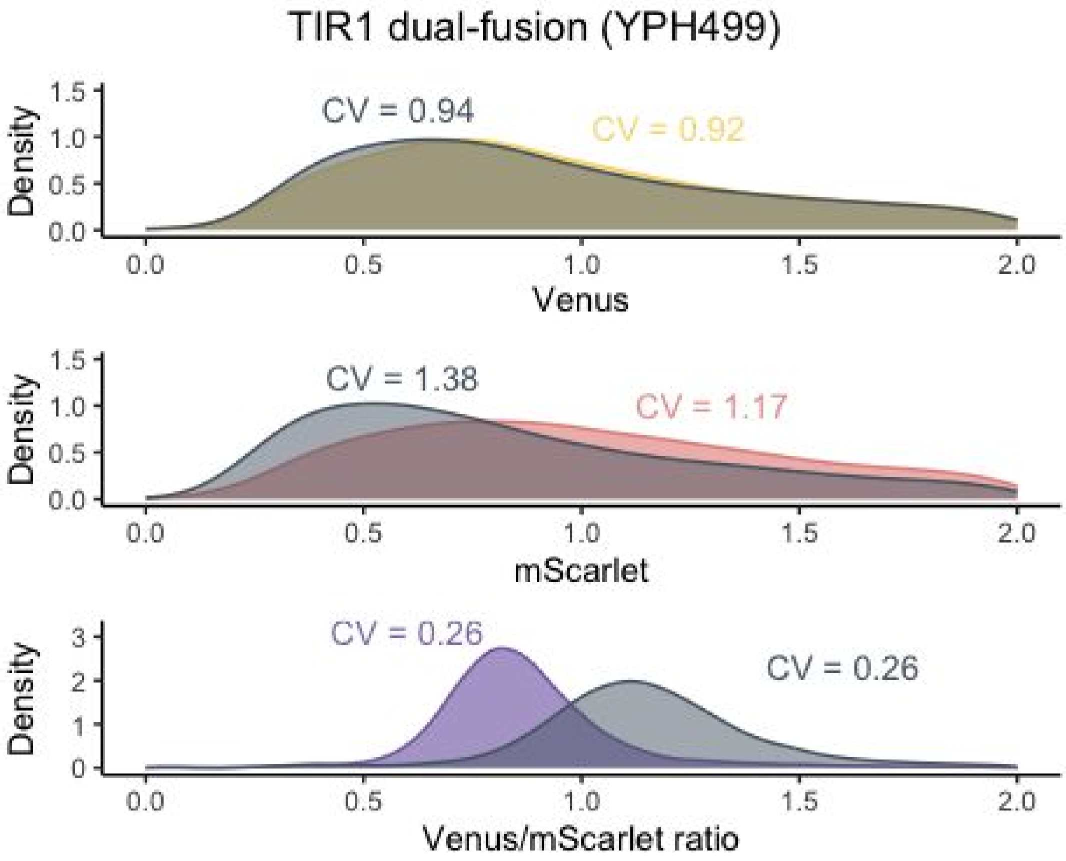


```
# ggsave('plot209_all.png', width = 6, height = 5)
```

Supporting Figure S2D: yWL210 (AFB2 cis in YPH499 yeast)

```
data210 <- steadyState(aplate_03112022, gated = TRUE)
# # [1] "No further gating applied."
# # [1] "Converting events..."
# data <- tidyFlow(aplate_20210619_W303)

data210 <- subset(data210, strain == "yWL210" & name %in% c("803112022-Pat-TCA08_Time-course assay_Auxin_yWL210-C1.fcs",
    "803112022-Pat-TCA08_Time-course assay_Control_yWL210-C1.fcs"))

cv <- function(x) return(round(sd(x)/mean(x), 2))


data210 <- subset(data210, BL1.A > 1 & YL1.A > 1)
data210$FLratio <- data210$BL1.A/data210$YL1.A
range(data210$BL1.A)
# # [1]      25 1048575
cv <- function(x) return(round(sd(x)/mean(x), 2))
```

```
data210$Venus <- data210$BL1.A/median(data210$BL1.A)
data210$mScarlet <- data210$YL1.A/median(data210$YL1.A)
data210$FLratio <- data210$BL1.A/data210$YL1.A
data210$ratio <- data210$FLratio/median(data210$FLratio)



data_long210 <- data210 %>%
    dplyr::select(treatment, Venus, mScarlet, ratio, strain) %>%
    pivot_longer(cols = c(Venus, mScarlet, ratio), names_to = "parameter",
values_to = "value") %>%
    dplyr::mutate(parameter = fct_relevel(parameter, "Venus"))

# need to also format CVs approriately for annotating

CV210 <- data210 %>%
    group_by(treatment) %>%
    dplyr::summarise(across(dplyr::where(is_double), cv)) %>%
    dplyr::select(treatment, Venus, mScarlet, ratio) %>%
    pivot_longer(cols = c(Venus, mScarlet, ratio), names_to = "parameter",
values_to = "value")


plot210 <- ggplot(data = data_long210, mapping = aes(x = value, color =
treatment)) +
    geom_density() + xlim(c(-1, 4)) + labs(x = "median normalized intensity",
color = "treatment") +
    theme_test() + geom_text(data = subset(CV210, treatment == "50 uM
Auxin"), aes(label = paste0("CV = ",
    value)), x = 2, y = 1) + geom_text(data = subset(CV210, treatment ==
"Control"),
    aes(label = paste0("CV = ", value)), x = 0, y = 1) +
scale_color_viridis_d(option = "D",
    end = 0.75, direction = -1)

venus210 <- ggplot(data210, aes(x = data210$Venus, group = treatment, color =
treatment,
    fill = treatment)) + geom_density(adjust = 1.5, alpha = 0.5) + labs(x =
"Venus",
    y = "Density") + xlim(0, 2) + ylim(0, 1.5) + theme_classic() +
theme(legend.position = "none") +
    geom_text(data = subset(CV210, parameter == "Venus" & treatment == "50 uM
Auxin"),
        aes(label = paste0("CV = ", value)), x = 1.2, y = 1.1) +
geom_text(data = subset(CV210,
    parameter == "Venus" & treatment == "Control"), aes(label = paste0("CV =
", value)),
    x = 0.58, y = 1.3) + scale_color_manual(values = c("#f7cb44b2",
"#566573")) +
    scale_fill_manual(values = c("#f7cb44b2", "#566573"))
```

```
red210 <- ggplot(data210, aes(x = data210$mScarlet, group = treatment, color
= treatment,
    fill = treatment)) + geom_density(adjust = 1.5, alpha = 0.5) + labs(x =
"mScarlet",
    y = "Density") + xlim(0, 2) + ylim(0, 1.5) + theme_classic() +
theme(legend.position = "none") +
    geom_text(data = subset(CV210, parameter == "mScarlet" & treatment == "50
uM Auxin"),
        aes(label = paste0("CV = ", value)), x = 1.3, y = 1) + geom_text(data
= subset(CV210,
    parameter == "mScarlet" & treatment == "Control"), aes(label = paste0("CV
= ",
    value)), x = 0.59, y = 1.3) + scale_color_manual(values = c("#de7065b2",
"#566573")) +
    scale_fill_manual(values = c("#de7065b2", "#566573"))


ratio210 <- ggplot(data210, aes(x = data210$ratio, group = treatment, color =
treatment,
    fill = treatment)) + geom_density(adjust = 1.5, alpha = 0.5) + labs(x =
"Venus/mScarlet ratio",
    y = "Density") + xlim(0, 2) + ylim(0, 3.2) + theme_classic() +
theme(legend.position = "none") +
    geom_text(data = subset(CV210, parameter == "ratio" & treatment == "50 uM
Auxin"),
        aes(label = paste0("CV = ", value)), x = 0.6, y = 2.3) +
geom_text(data = subset(CV210,
    parameter == "ratio" & treatment == "Control"), aes(label = paste0("CV =
", value)),
    x = 1.5, y = 1.8) + scale_color_manual(values = c("#593d9cb2",
"#566573")) +
    scale_fill_manual(values = c("#593d9cb2", "#566573"))
ratio210
```

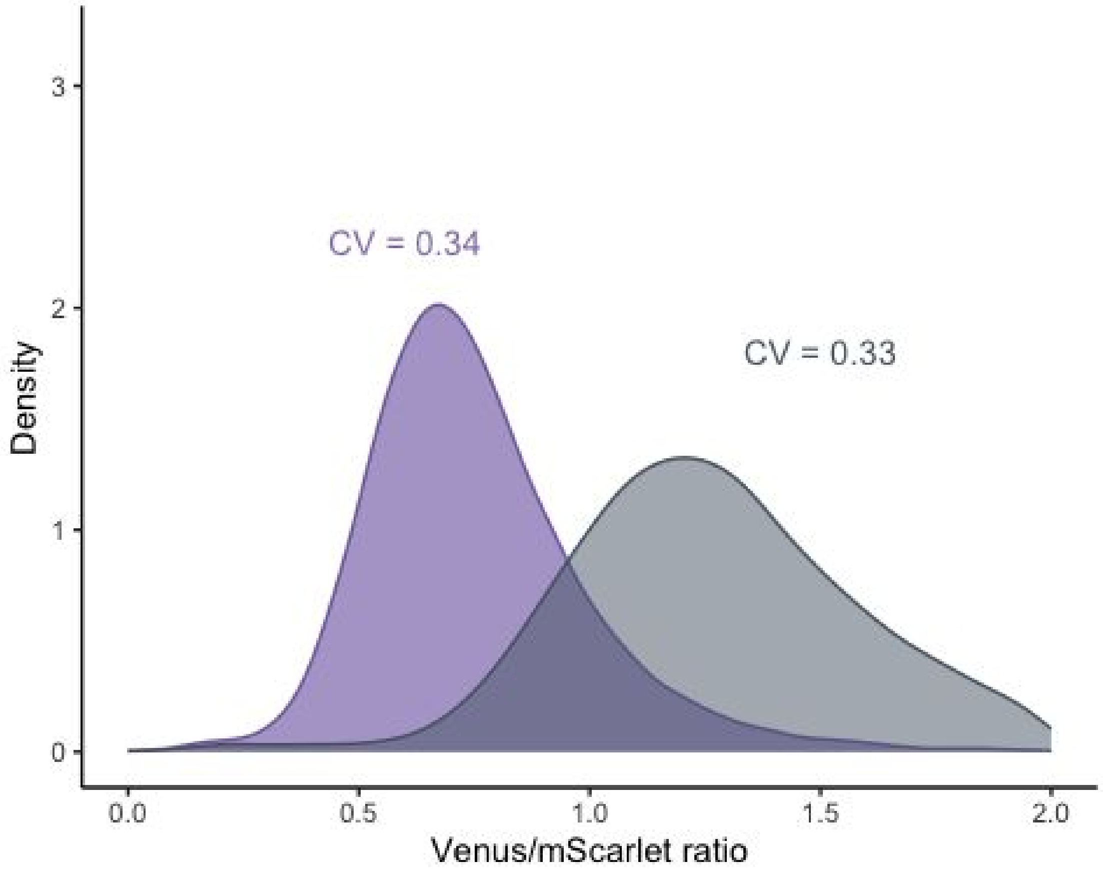


```
plot210 <- grid.arrange(venus210, red210, ratio210, nrow = 3, ncol = 1)
```

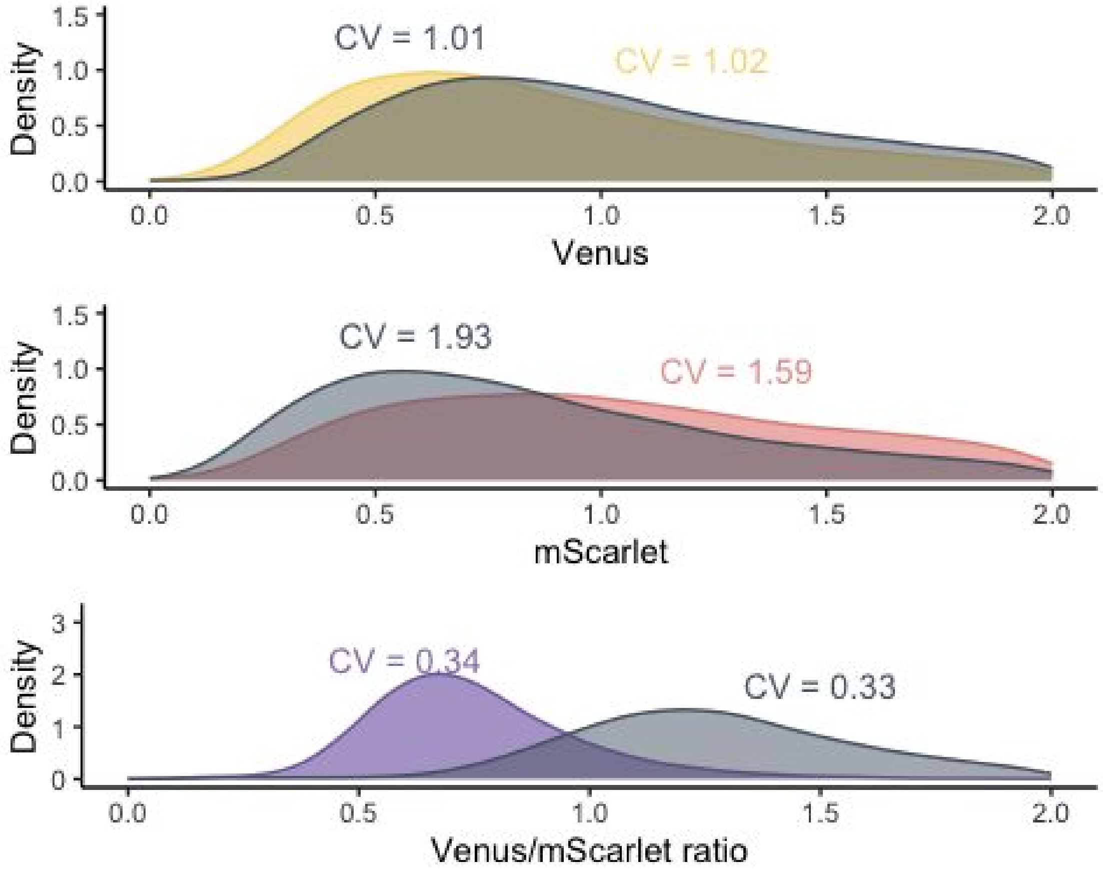


```
plot210 <- annotate_figure(plot210, top = text_grob("AFB2", color = "black",
size = 12))

plot210
```

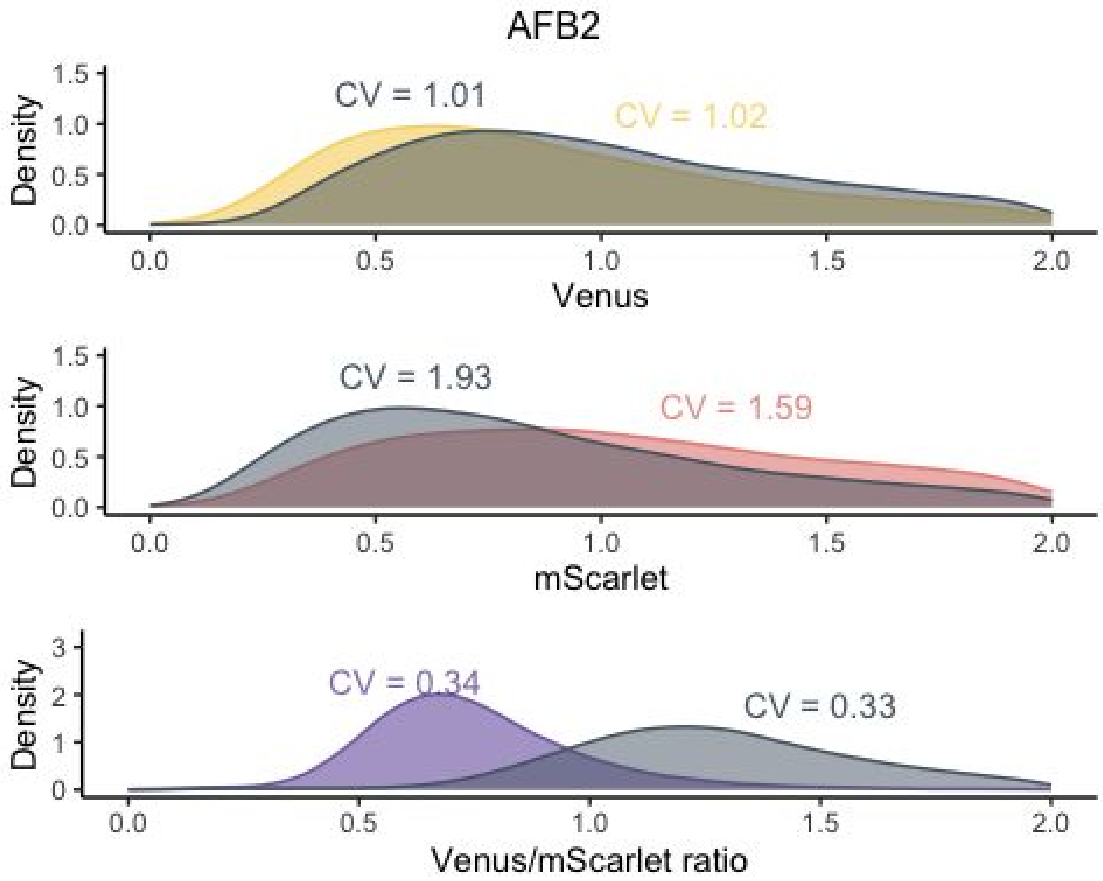


```
plot210_all <- venus210/red210/ratio210 + plot_annotation(title = "AFB2 dual-
fusion (YPH499)") &
    theme(plot.title = element_text(hjust = 0.5))
plot210_all
```

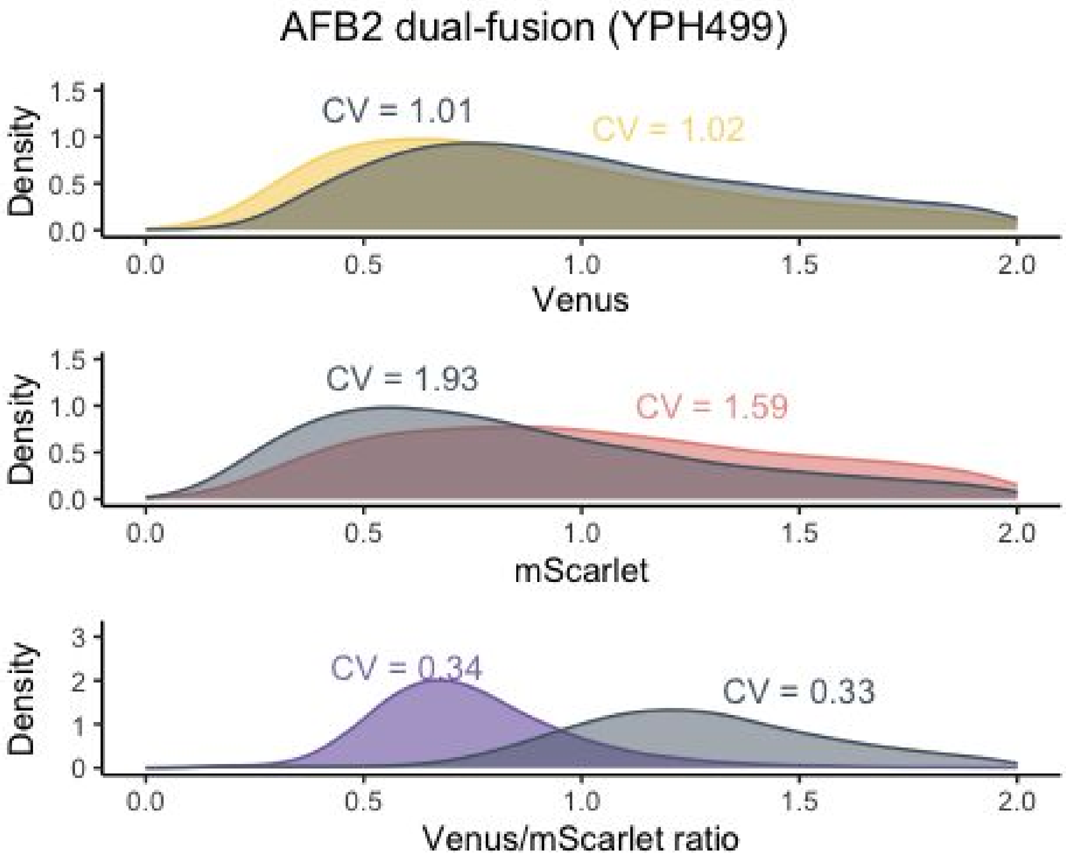


```
# ggsave('plot210_all.png', width = 6, height = 5)

cvplots_all <- ggarrange(plot185_all, plot186_all, plot209_all, plot210_all,
labels = c("A",
    "B", "C", "D"), nrow = 1, ncol = 4)

cvplots_all <- ggarrange(plot185_all, plot186_all, plot209_all, plot210_all,
nrow = 1,
    ncol = 4)
cvplots_all
```

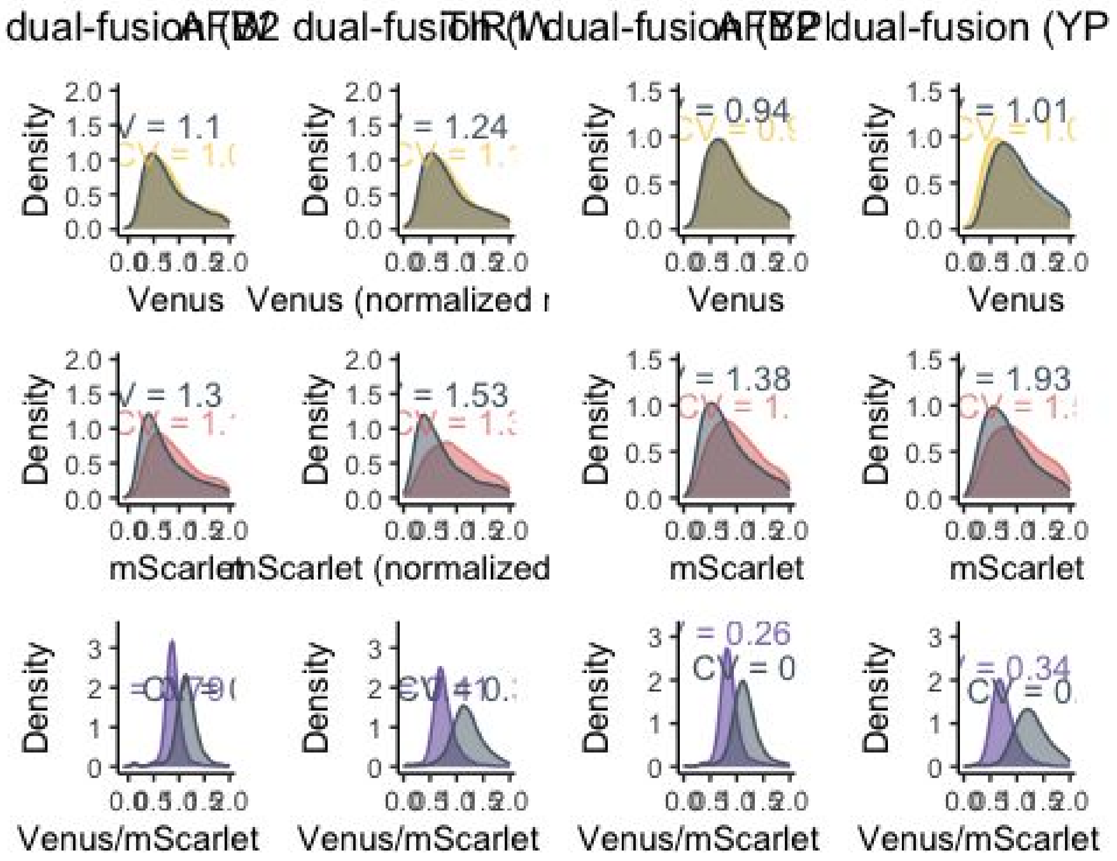


```
# ggsave('cvplots_all.png', width = 12.5, height = 5)
ggsave('Supplement1.pdf',
# width = 12.5, height = 5)
```

Supporting Figure S4: Comparison between the biosensor in cis and trans in the same W303 yeast strain ()

- yWL161 (TIR1 in trans, W303 yeast)
- yWL162 (AFB2 in trans, W303 yeast)
- yWL185 (TIR1 in cis, W303 yeast)
- yWL186 (AFB2in cis, W303 yeast)

```
plate_20210619_W303 <- read.plateSet(path = "Data for
publication/20210619/W303only/",
    pattern = "r*")

annotation <- createAnnotation(yourFlowSet = plate_20210619_W303)
write.csv(annotation, "Data for
publication/20210619/20210619_W303only_annotation.csv")

annotation <- read.csv("Data for
publication/20210619/20210619_W303only_annotation.csv")
aplate_20210619_W303 <- annotateFlowSet(yourFlowSet = plate_20210619_W303,
annotation_df = annotation,
```

```
    mergeBy = "name")
head(rownames(pData(aplate_20210619_W303)))
# # [1] "1B01.fcs" "1B02.fcs" "1B03.fcs" "1B04.fcs" "1B07.fcs" "1B08.fcs"
head(pData(aplate_20210619_W303))
# #                 name folder X strain treatment reading before_after
# # 1B01.fcs 1B01.fcs    r02 1 yWL161   Control       2        before
# # 1B02.fcs 1B02.fcs    r02 2 yWL162   Control       2        before
# # 1B03.fcs 1B03.fcs    r02 3 yWL185   Control       2        before
# # 1B04.fcs 1B04.fcs    r02 4 yWL186   Control       2        before
# # 1B07.fcs 1B07.fcs    r02 5 yWL161     Auxin       2        before
# # 1B08.fcs 1B08.fcs    r02 6 yWL162     Auxin       2        before
# #          design_construct       design_yeast
# # 1B01.fcs       TIR1 trans TIR1 single-fusion
# # 1B02.fcs       AFB2 trans AFB2 single-fusion
# # 1B03.fcs         TIR1 cis   TIR1 dual-fusion
# # 1B04.fcs         AFB2 cis   AFB2 dual-fusion
# # 1B07.fcs       TIR1 trans TIR1 single-fusion
# # 1B08.fcs       AFB2 trans AFB2 single-fusion


plate_20210619_W303_sum <- summarizeFlow(aplate_20210619_W303, channel =
c("FL1.A",
    "FL4.A"), gated = TRUE)
# # [1] "Summarizing all events..."

plate_20210619_W303_sum$treatment <-
factor(plate_20210619_W303_sum$treatment, levels = c("Control",
    "Auxin"))

# The time auxin addition is equal to time zero
time0 <- "4E01.fcs"
# or whatever well was being read when auxin was added

plate_20210619_W303_sum$time <- plate_20210619_W303_sum$btime -
plate_20210619_W303_sum[[which(plate_20210619_W303_sum$name ==
    time0), "btime"]]
# single bracket -->extracting the all name, 2 brackets extract just single
# 'value' or 'values'

library(tidyverse)
# %>% pass the object to the right side

plate_20210619_W303_sum <- plate_20210619_W303_sum %>%
    mutate(design = case_when(strain %in% c("yWL185", "yWL186") ~ "cis",
strain %in%
        c("yWL161", "yWL162") ~ "trans"), fbox = case_when(strain %in%
c("yWL161",
        "yWL185") ~ "TIR1", strain %in% c("yWL162", "yWL186") ~ "AFB2"))

plate_20210619_W303_sum <- plate_20210619_W303_sum %>%
```

```
    mutate(ratio = FL1.Amean/FL4.Amean) %>%
    group_by(design, fbox) %>%
    mutate(normalizedratio = ratio/mean(ratio))

plate_20210619_W303_sum <- plate_20210619_W303_sum %>%
    mutate(green = FL1.Amean) %>%
    group_by(design, fbox) %>%
    mutate(normalized_Greenexpression = FL1.Amean/mean(FL1.Amean))

plate_20210619_W303_sum <- plate_20210619_W303_sum %>%
    mutate(red = FL4.Amean) %>%
    group_by(design, fbox) %>%
    mutate(normalized_Redexpression = FL4.Amean/mean(FL4.Amean))
```

Supporting Figure S4A: Venus-IAA17 expression for single and dual fusion biosensor in the same W303 yeast strain

```
boxvenus <- ggplot(subset(plate_20210619_W303_sum, before_after == "after" &
reading %in%
    c("11", "12", "13", "14")), aes(x = design_construct, y = FL1.Amean, fill
= treatment)) +
    geom_boxplot(aes(color = treatment), alpha = 0.7) + geom_jitter(position
= position_dodge(0.8),
    aes(color = treatment), size = 1) + scale_fill_manual(values =
c("#BFC9CA", "#FDCB26FF")) +
    scale_color_manual(values = c("#7F8C8D", "#FEB72DFF")) + ylab("Venus-
IAA17") +
    xlab("Biosensor Designs") + theme_classic() + theme(legend.position =
"right",
    axis.title.y = element_text(size = 12, face = "bold"), axis.title.x =
element_text(size = 12,
        face = "bold"), axis.text = element_text(size = 10), legend.title =
element_text(size = 10,
        face = "bold"), legend.text = element_text(size = 10)) + labs(fill =
"Treatment",
    color = "Treatment", shape = "Treatment") + stat_compare_means(method =
"t.test",
    label = "..p.signif..", hide.ns = FALSE, size = 5, label.y = 0.2) +
scale_x_discrete(labels = c(`AFB2 cis` = "AFB2\ndual-fusion",
    `AFB2 trans` = "AFB2\nsingle-fusion", `TIR1 cis` = "TIR1\ndual-fusion",
`TIR1 trans` = "TIR1\nsingle-fusion"))


boxvenus
```

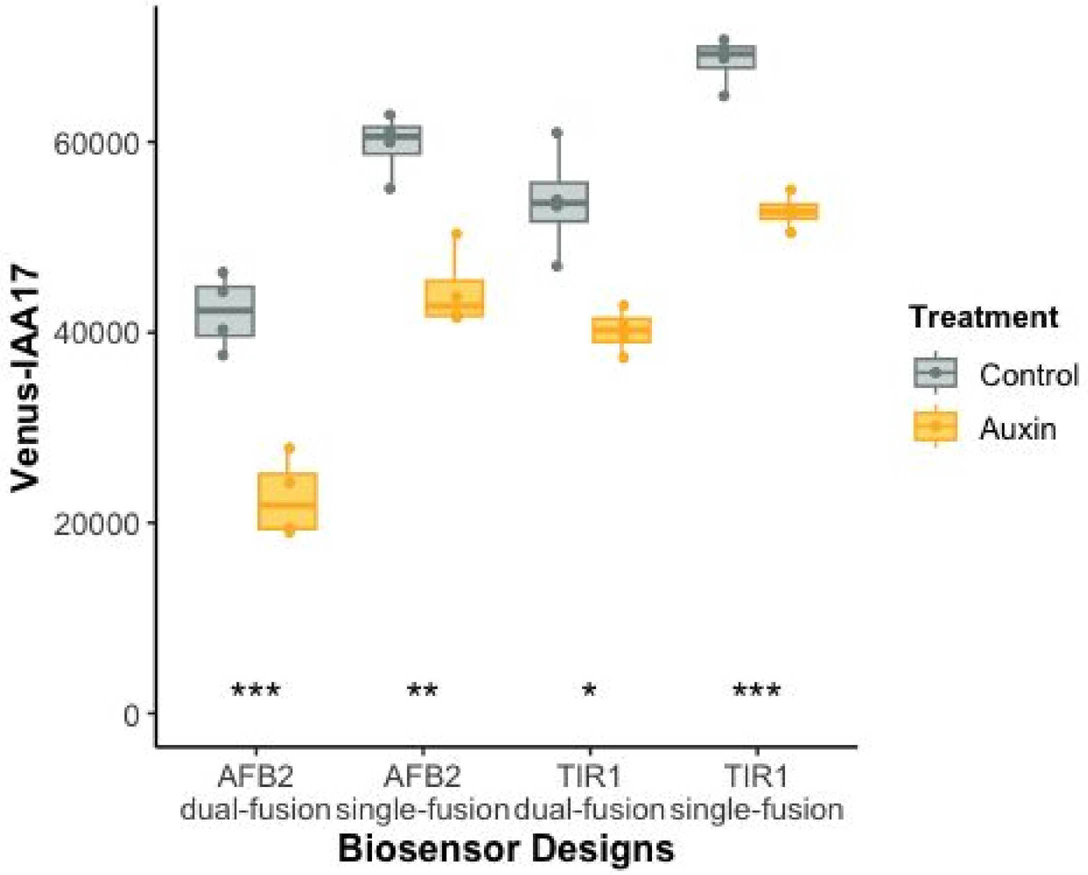

Supporting Figure S4B: TIR1/AFB2- or free mScarlet-I expression for single and dual fusion biosensor in the same W303 yeast strain

```
boxmScarlet <- ggplot(subset(plate_20210619_W303_sum, before_after == "after"
& reading %in%
    c("11", "12", "13", "14")), aes(x = design_construct, y = FL4.Amean, fill
= treatment)) +
    geom_boxplot(aes(color = treatment), alpha = 0.7) + geom_jitter(position
= position_dodge(0.8),
    aes(color = treatment), size = 1) + scale_fill_manual(values =
c("#BFC9CA", "#D14E72FF")) +
    scale_color_manual(values = c("#7F8C8D", "#9C2964FF")) + ylab("TIR1/AFB2-
\n or free mScarlet-I") +
    xlab("Biosensor Designs") + theme_classic() + theme(legend.position =
"right",
    axis.title.y = element_text(size = 12, face = "bold"), axis.title.x =
element_text(size = 12,
        face = "bold"), axis.text = element_text(size = 10), legend.title =
element_text(size = 10,
        face = "bold"), legend.text = element_text(size = 10)) + labs(fill =
"Treatment",
    color = "Treatment", shape = "Treatment") + stat_compare_means(method =
"t.test",
```

```
    label = "..p.signif..", hide.ns = FALSE, size = 5, label.y = 0.2) +
scale_x_discrete(labels = c(`AFB2 cis` = "AFB2\ndual-fusion",
    `AFB2 trans` = "AFB2\nsingle-fusion", `TIR1 cis` = "TIR1\ndual-fusion",
`TIR1 trans` = "TIR1\nsingle-fusion"))



boxmScarlet
```

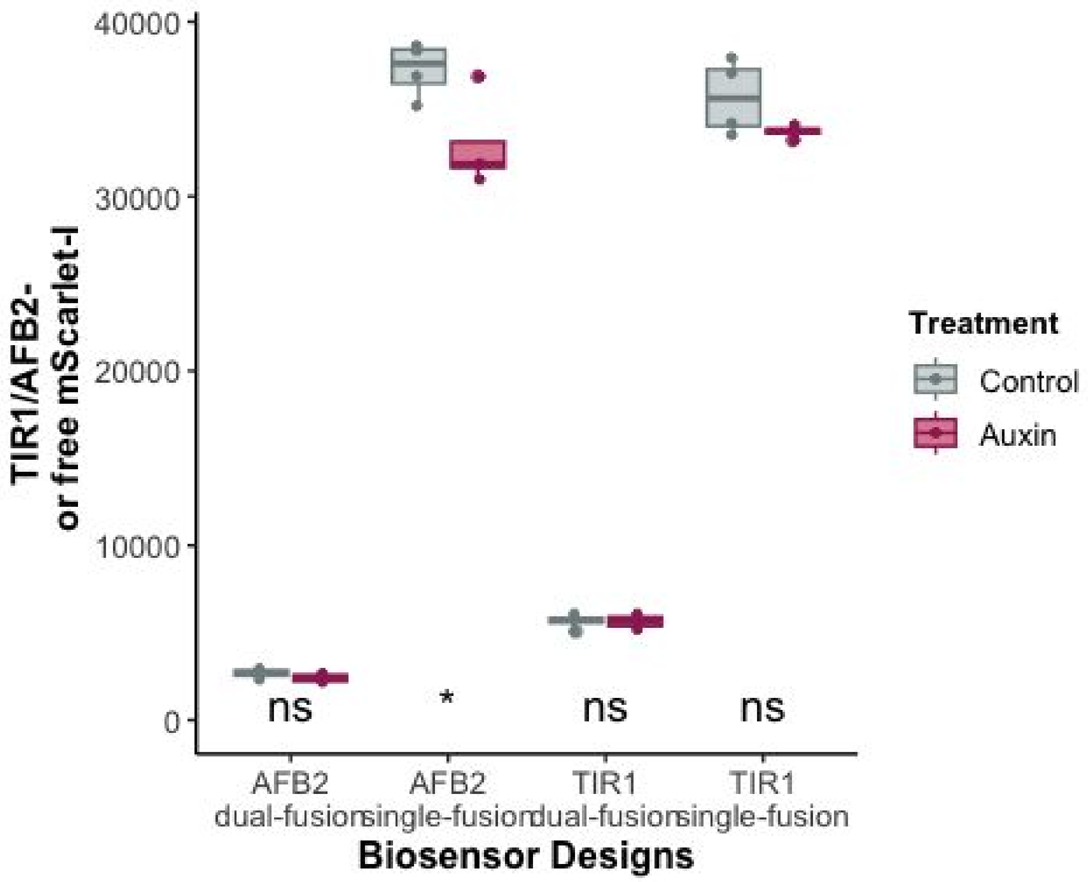

Supporting Figure S4C: Venus/mScarlet-I expression ratio for single and dual fusion biosensor in the same W303 yeast strain

```
boxvenusmScarlet <- ggplot(subset(plate_20210619_W303_sum, before_after ==
"after" &
    reading %in% c("11", "12", "13", "14")), aes(x = design_construct, y =
normalizedratio,
    fill = treatment)) + geom_boxplot(aes(color = treatment), alpha = 0.7) +
geom_jitter(position = position_dodge(0.8),
    aes(color = treatment), size = 1) + scale_fill_manual(values =
c("#BFC9CA", "#6b4596ff")) +
    scale_color_manual(values = c("#7F8C8D", "#6b4596ff")) + ylab("Normalized
Venus/mScarlet-I ") +
```

```
    xlab("Biosensor Designs") + theme_classic() + theme(legend.position =
"right",
    axis.title.y = element_text(size = 12, face = "bold"), axis.title.x =
element_text(size = 12,
        face = "bold"), axis.text = element_text(size = 10), legend.title =
element_text(size = 10,
        face = "bold"), legend.text = element_text(size = 10)) + labs(fill =
"Treatment",
    color = "Treatment", shape = "Treatment") + stat_compare_means(method =
"t.test",
    label = "..p.signif..", hide.ns = FALSE, size = 5, label.y = 0.5) +
scale_x_discrete(labels = c(`AFB2 cis` = "AFB2\ndual-fusion",
    `AFB2 trans` = "AFB2\nsingle-fusion", `TIR1 cis` = "TIR1\ndual-fusion",
`TIR1 trans` = "TIR1\nsingle-fusion"))

boxvenusmScarlet
```

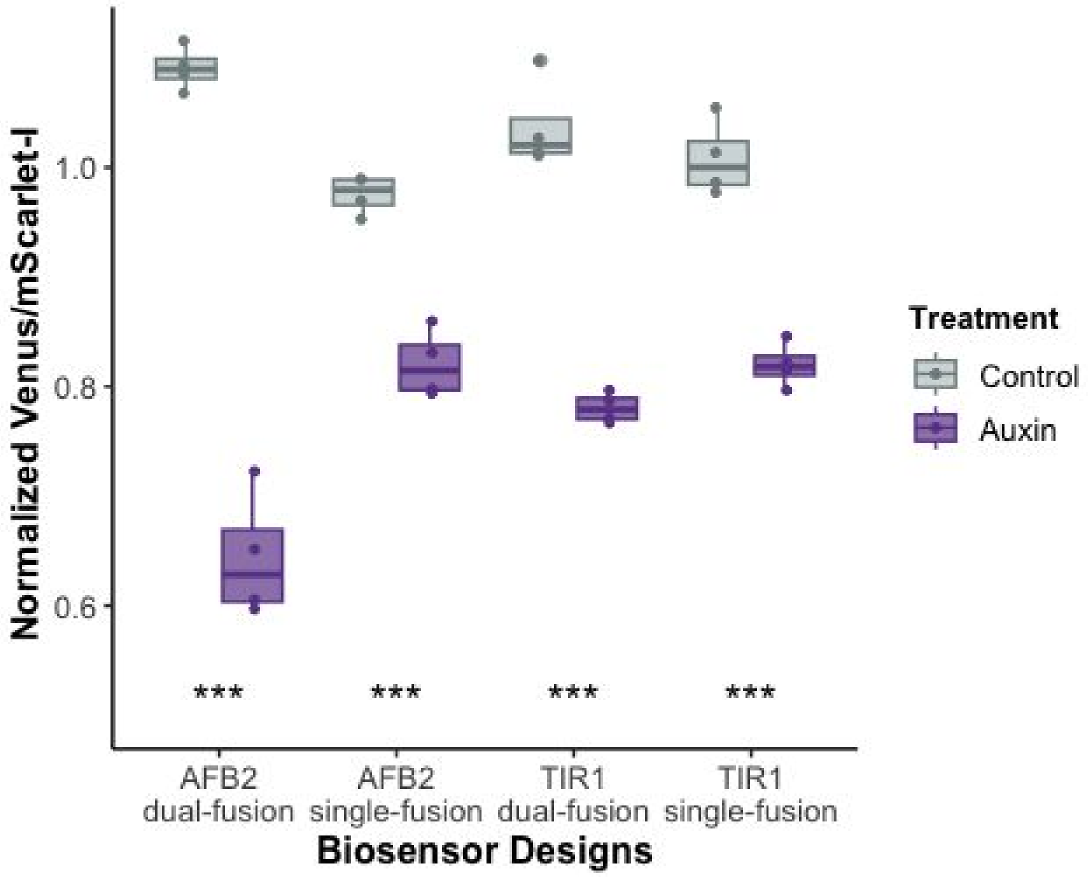


```
boxvenusmScarlet <- ggarrange(boxvenusmScarlet, labels = c("C"), nrow = 1,
ncol = 1)


boxFL303_VenusScarlet <- ggarrange(boxvenus, boxmScarlet, labels = c("A",
```

```
"B"), nrow = 2,
    ncol = 1)
boxFL303_VenusScarlet
```

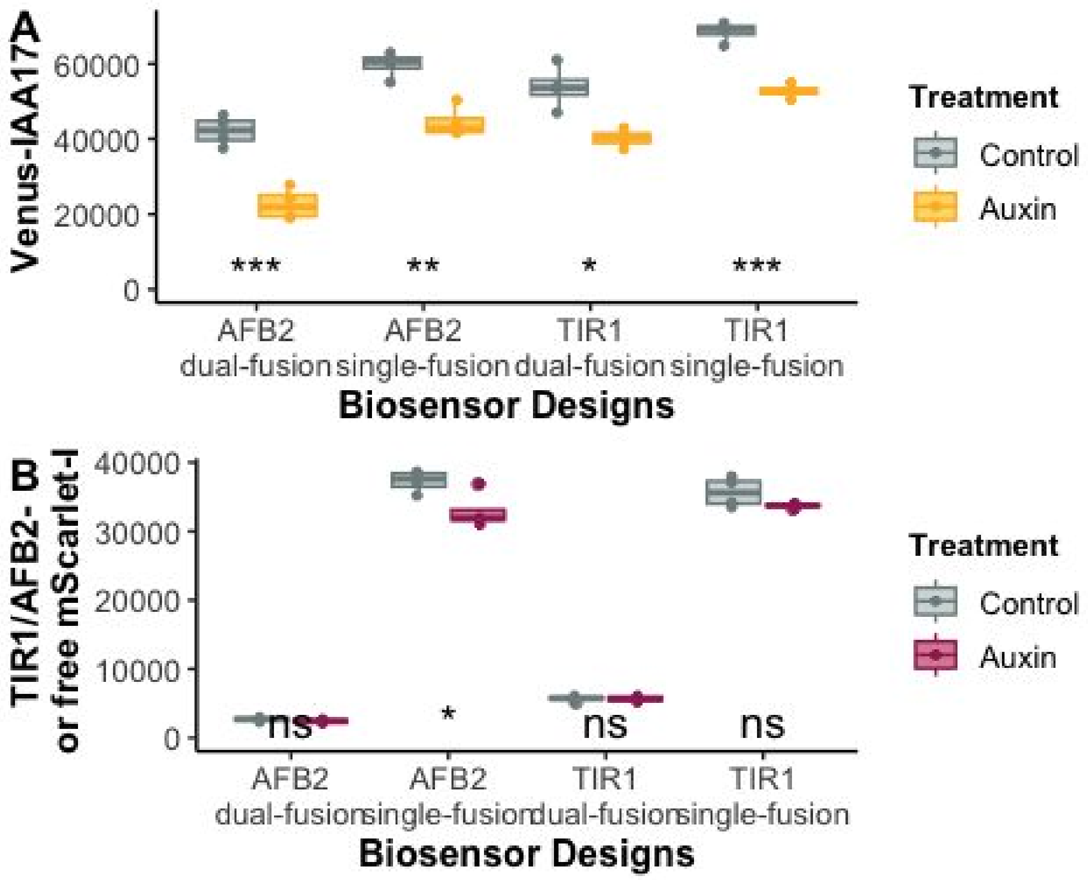


```
Sup3 <- ggarrange(boxFL303_VenusScarlet, boxvenusmScarlet, nrow = 1, ncol =
2, widths = c(1,
    0.8))
Sup3
```

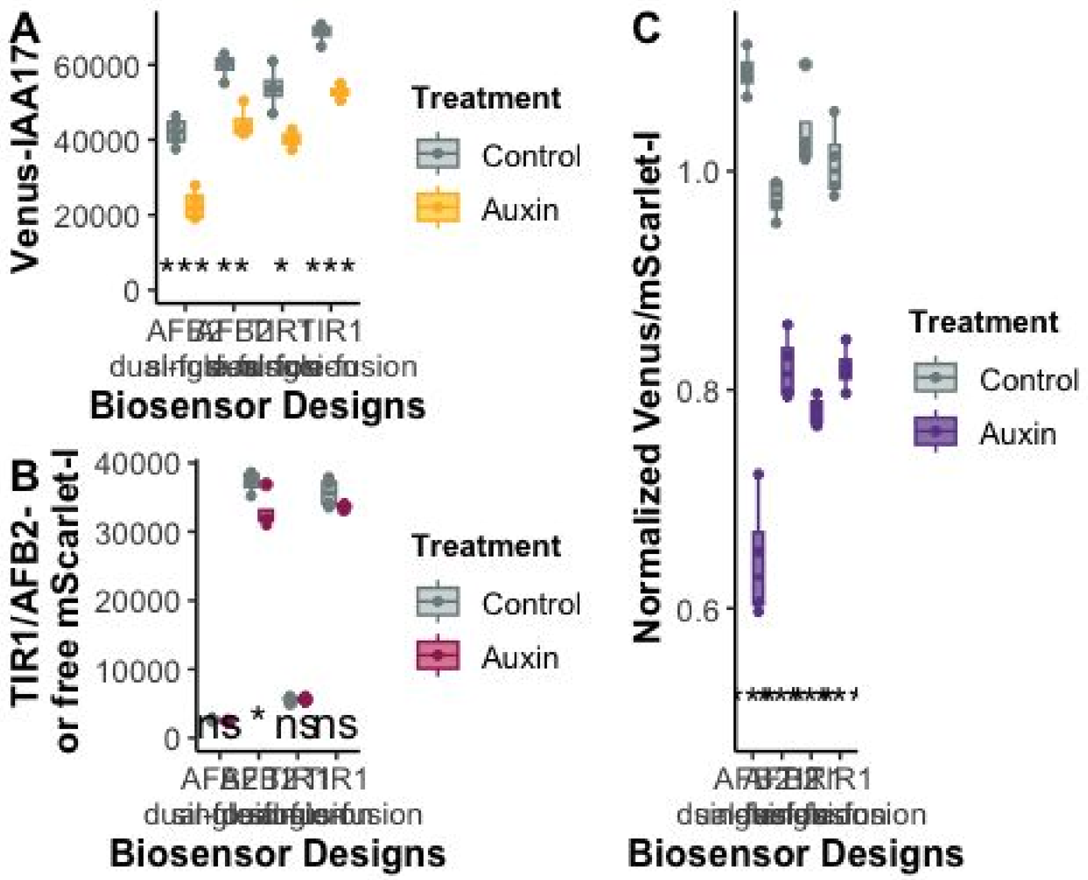


```
# ggsave('Supplment3_renamed.pdf', height = 5, width = 12)
```

Supporting Figure S5: Comparison of biosensor designs in different yeast strains

```
plate_07142023_3readsbefore_after <- read.plateSet(path = "Data for 
publication/07142023_Time-course assay/read123-8910/",
    pattern = "TCA*")

annotation <- createAnnotation(yourFlowSet = 
plate_07142023_3readsbefore_after)
write.csv(annotation, "Data for publication/07142023_Time-course 
assay/3readsbefore_after_annotation.csv")

annotation <- read.csv("Data for publication/07142023_Time-course 
assay/3readsbefore_after_annotation.csv")
aplate_07142023_3readsbefore_after <- annotateFlowSet(yourFlowSet = 
plate_07142023_3readsbefore_after,
    annotation_df = annotation, mergeBy = "name")
head(rownames(pData(aplate_07142023_3readsbefore_after)))
# # [1] "1Pat-07142023-Biosensors_cis-trans_TCA01_Experiment_50uM IAA 
treatment_yWL161.fcs"
# # [2] "1Pat-07142023-Biosensors_cis-trans_TCA01_Experiment_50uM IAA 
treatment_yWL162.fcs"
```

```
# # [3] "1Pat-07142023-Biosensors_cis-trans_TCA01_Experiment_50uM IAA
treatment_yWL185.fcs"
# # [4] "1Pat-07142023-Biosensors_cis-trans_TCA01_Experiment_50uM IAA
treatment_yWL186.fcs"
# # [5] "1Pat-07142023-Biosensors_cis-trans_TCA01_Experiment_50uM IAA
treatment_yWL209.fcs"
# # [6] "1Pat-07142023-Biosensors_cis-trans_TCA01_Experiment_50uM IAA
treatment_yWL210.fcs"
head(pData(aplate_07142023_3readsbefore_after))
# #
name
# # 1Pat-07142023-Biosensors_cis-trans_TCA01_Experiment_50uM IAA
treatment_yWL161.fcs 1Pat-07142023-Biosensors_cis-trans_TCA01_Experiment_50uM
IAA treatment_yWL161.fcs
# # 1Pat-07142023-Biosensors_cis-trans_TCA01_Experiment_50uM IAA
treatment_yWL162.fcs 1Pat-07142023-Biosensors_cis-trans_TCA01_Experiment_50uM
IAA treatment_yWL162.fcs
# # 1Pat-07142023-Biosensors_cis-trans_TCA01_Experiment_50uM IAA
treatment_yWL185.fcs 1Pat-07142023-Biosensors_cis-trans_TCA01_Experiment_50uM
IAA treatment_yWL185.fcs
# # 1Pat-07142023-Biosensors_cis-trans_TCA01_Experiment_50uM IAA
treatment_yWL186.fcs 1Pat-07142023-Biosensors_cis-trans_TCA01_Experiment_50uM
IAA treatment_yWL186.fcs
# # 1Pat-07142023-Biosensors_cis-trans_TCA01_Experiment_50uM IAA
treatment_yWL209.fcs 1Pat-07142023-Biosensors_cis-trans_TCA01_Experiment_50uM
IAA treatment_yWL209.fcs
# # 1Pat-07142023-Biosensors_cis-trans_TCA01_Experiment_50uM IAA
treatment_yWL210.fcs 1Pat-07142023-Biosensors_cis-trans_TCA01_Experiment_50uM
IAA treatment_yWL210.fcs
# #
folder
# # 1Pat-07142023-Biosensors_cis-trans_TCA01_Experiment_50uM IAA
treatment_yWL161.fcs  TCA01
# # 1Pat-07142023-Biosensors_cis-trans_TCA01_Experiment_50uM IAA
treatment_yWL162.fcs  TCA01
# # 1Pat-07142023-Biosensors_cis-trans_TCA01_Experiment_50uM IAA
treatment_yWL185.fcs  TCA01
# # 1Pat-07142023-Biosensors_cis-trans_TCA01_Experiment_50uM IAA
treatment_yWL186.fcs  TCA01
# # 1Pat-07142023-Biosensors_cis-trans_TCA01_Experiment_50uM IAA
treatment_yWL209.fcs  TCA01
# # 1Pat-07142023-Biosensors_cis-trans_TCA01_Experiment_50uM IAA
treatment_yWL210.fcs  TCA01
# #
X
# # 1Pat-07142023-Biosensors_cis-trans_TCA01_Experiment_50uM IAA
treatment_yWL161.fcs 1
# # 1Pat-07142023-Biosensors_cis-trans_TCA01_Experiment_50uM IAA
treatment_yWL162.fcs 2
# # 1Pat-07142023-Biosensors_cis-trans_TCA01_Experiment_50uM IAA
```

```
treatment_yWL185.fcs 3
# # 1Pat-07142023-Biosensors_cis-trans_TCA01_Experiment_50uM IAA
treatment_yWL186.fcs 4
# # 1Pat-07142023-Biosensors_cis-trans_TCA01_Experiment_50uM IAA
treatment_yWL209.fcs 5
# # 1Pat-07142023-Biosensors_cis-trans_TCA01_Experiment_50uM IAA
treatment_yWL210.fcs 6
# #
strain
# # 1Pat-07142023-Biosensors_cis-trans_TCA01_Experiment_50uM IAA
treatment_yWL161.fcs yWL161
# # 1Pat-07142023-Biosensors_cis-trans_TCA01_Experiment_50uM IAA
treatment_yWL162.fcs yWL162
# # 1Pat-07142023-Biosensors_cis-trans_TCA01_Experiment_50uM IAA
treatment_yWL185.fcs yWL185
# # 1Pat-07142023-Biosensors_cis-trans_TCA01_Experiment_50uM IAA
treatment_yWL186.fcs yWL186
# # 1Pat-07142023-Biosensors_cis-trans_TCA01_Experiment_50uM IAA
treatment_yWL209.fcs yWL209
# # 1Pat-07142023-Biosensors_cis-trans_TCA01_Experiment_50uM IAA
treatment_yWL210.fcs yWL210
# #
treatment
# # 1Pat-07142023-Biosensors_cis-trans_TCA01_Experiment_50uM IAA
treatment_yWL161.fcs  50uM IAA
# # 1Pat-07142023-Biosensors_cis-trans_TCA01_Experiment_50uM IAA
treatment_yWL162.fcs  50uM IAA
# # 1Pat-07142023-Biosensors_cis-trans_TCA01_Experiment_50uM IAA
treatment_yWL185.fcs  50uM IAA
# # 1Pat-07142023-Biosensors_cis-trans_TCA01_Experiment_50uM IAA
treatment_yWL186.fcs  50uM IAA
# # 1Pat-07142023-Biosensors_cis-trans_TCA01_Experiment_50uM IAA
treatment_yWL209.fcs  50uM IAA
# # 1Pat-07142023-Biosensors_cis-trans_TCA01_Experiment_50uM IAA
treatment_yWL210.fcs  50uM IAA
# #
design_strain
# # 1Pat-07142023-Biosensors_cis-trans_TCA01_Experiment_50uM IAA
treatment_yWL161.fcs TIR1-trans_W303
# # 1Pat-07142023-Biosensors_cis-trans_TCA01_Experiment_50uM IAA
treatment_yWL162.fcs AFB2-trans_W303
# # 1Pat-07142023-Biosensors_cis-trans_TCA01_Experiment_50uM IAA
treatment_yWL185.fcs   TIR1-cis_W303
# # 1Pat-07142023-Biosensors_cis-trans_TCA01_Experiment_50uM IAA
treatment_yWL186.fcs   AFB2-cis_W303
# # 1Pat-07142023-Biosensors_cis-trans_TCA01_Experiment_50uM IAA
treatment_yWL209.fcs TIR1-cis_YPH499
# # 1Pat-07142023-Biosensors_cis-trans_TCA01_Experiment_50uM IAA
treatment_yWL210.fcs AFB2-cis_YPH499
# #
```

```
receptor
# # 1Pat-07142023-Biosensors_cis-trans_TCA01_Experiment_50uM IAA
treatment_yWL161.fcs     TIR1
# # 1Pat-07142023-Biosensors_cis-trans_TCA01_Experiment_50uM IAA
treatment_yWL162.fcs     AFB2
# # 1Pat-07142023-Biosensors_cis-trans_TCA01_Experiment_50uM IAA
treatment_yWL185.fcs     TIR1
# # 1Pat-07142023-Biosensors_cis-trans_TCA01_Experiment_50uM IAA
treatment_yWL186.fcs     AFB2
# # 1Pat-07142023-Biosensors_cis-trans_TCA01_Experiment_50uM IAA
treatment_yWL209.fcs     TIR1
# # 1Pat-07142023-Biosensors_cis-trans_TCA01_Experiment_50uM IAA
treatment_yWL210.fcs     AFB2
# #
reading
# # 1Pat-07142023-Biosensors_cis-trans_TCA01_Experiment_50uM IAA
treatment_yWL161.fcs       1
# # 1Pat-07142023-Biosensors_cis-trans_TCA01_Experiment_50uM IAA
treatment_yWL162.fcs       1
# # 1Pat-07142023-Biosensors_cis-trans_TCA01_Experiment_50uM IAA
treatment_yWL185.fcs       1
# # 1Pat-07142023-Biosensors_cis-trans_TCA01_Experiment_50uM IAA
treatment_yWL186.fcs       1
# # 1Pat-07142023-Biosensors_cis-trans_TCA01_Experiment_50uM IAA
treatment_yWL209.fcs       1
# # 1Pat-07142023-Biosensors_cis-trans_TCA01_Experiment_50uM IAA
treatment_yWL210.fcs       1
# #
before_after
# # 1Pat-07142023-Biosensors_cis-trans_TCA01_Experiment_50uM IAA
treatment_yWL161.fcs       before
# # 1Pat-07142023-Biosensors_cis-trans_TCA01_Experiment_50uM IAA
treatment_yWL162.fcs       before
# # 1Pat-07142023-Biosensors_cis-trans_TCA01_Experiment_50uM IAA
treatment_yWL185.fcs       before
# # 1Pat-07142023-Biosensors_cis-trans_TCA01_Experiment_50uM IAA
treatment_yWL186.fcs       before
# # 1Pat-07142023-Biosensors_cis-trans_TCA01_Experiment_50uM IAA
treatment_yWL209.fcs       before
# # 1Pat-07142023-Biosensors_cis-trans_TCA01_Experiment_50uM IAA
treatment_yWL210.fcs       before
# #
phase
# # 1Pat-07142023-Biosensors_cis-trans_TCA01_Experiment_50uM IAA
treatment_yWL161.fcs exponential
# # 1Pat-07142023-Biosensors_cis-trans_TCA01_Experiment_50uM IAA
treatment_yWL162.fcs exponential
# # 1Pat-07142023-Biosensors_cis-trans_TCA01_Experiment_50uM IAA
treatment_yWL185.fcs exponential
# # 1Pat-07142023-Biosensors_cis-trans_TCA01_Experiment_50uM IAA
```

```
treatment_yWL186.fcs exponential
# # 1Pat-07142023-Biosensors_cis-trans_TCA01_Experiment_50uM IAA
treatment_yWL209.fcs exponential
# # 1Pat-07142023-Biosensors_cis-trans_TCA01_Experiment_50uM IAA
treatment_yWL210.fcs exponential
# #
design
# # 1Pat-07142023-Biosensors_cis-trans_TCA01_Experiment_50uM IAA
treatment_yWL161.fcs TIR1 single-fusion
# # 1Pat-07142023-Biosensors_cis-trans_TCA01_Experiment_50uM IAA
treatment_yWL162.fcs AFB2 single-fusion
# # 1Pat-07142023-Biosensors_cis-trans_TCA01_Experiment_50uM IAA
treatment_yWL185.fcs   TIR1 dual-fusion
# # 1Pat-07142023-Biosensors_cis-trans_TCA01_Experiment_50uM IAA
treatment_yWL186.fcs   AFB2 dual-fusion
# # 1Pat-07142023-Biosensors_cis-trans_TCA01_Experiment_50uM IAA
treatment_yWL209.fcs   TIR1 dual-fusion
# # 1Pat-07142023-Biosensors_cis-trans_TCA01_Experiment_50uM IAA
treatment_yWL210.fcs   AFB2 dual-fusion
# #
yeast
# # 1Pat-07142023-Biosensors_cis-trans_TCA01_Experiment_50uM IAA
treatment_yWL161.fcs   W303
# # 1Pat-07142023-Biosensors_cis-trans_TCA01_Experiment_50uM IAA
treatment_yWL162.fcs   W303
# # 1Pat-07142023-Biosensors_cis-trans_TCA01_Experiment_50uM IAA
treatment_yWL185.fcs   W303
# # 1Pat-07142023-Biosensors_cis-trans_TCA01_Experiment_50uM IAA
treatment_yWL186.fcs   W303
# # 1Pat-07142023-Biosensors_cis-trans_TCA01_Experiment_50uM IAA
treatment_yWL209.fcs YPH499
# # 1Pat-07142023-Biosensors_cis-trans_TCA01_Experiment_50uM IAA
treatment_yWL210.fcs YPH499
# #
design_yeast
# # 1Pat-07142023-Biosensors_cis-trans_TCA01_Experiment_50uM IAA
treatment_yWL161.fcs TIR1 single-fusion (W303)
# # 1Pat-07142023-Biosensors_cis-trans_TCA01_Experiment_50uM IAA
treatment_yWL162.fcs AFB2 single-fusion (W303)
# # 1Pat-07142023-Biosensors_cis-trans_TCA01_Experiment_50uM IAA
treatment_yWL185.fcs   TIR1 dual-fusion (W303)
# # 1Pat-07142023-Biosensors_cis-trans_TCA01_Experiment_50uM IAA
treatment_yWL186.fcs   AFB2 dual-fusion (W303)
# # 1Pat-07142023-Biosensors_cis-trans_TCA01_Experiment_50uM IAA
treatment_yWL209.fcs TIR1 dual-fusion (YPH499)
# # 1Pat-07142023-Biosensors_cis-trans_TCA01_Experiment_50uM IAA
treatment_yWL210.fcs AFB2 dual-fusion (YPH499)
# #
receptor.1
# # 1Pat-07142023-Biosensors_cis-trans_TCA01_Experiment_50uM IAA
```

```
treatment_yWL161.fcs       TIR1
# # 1Pat-07142023-Biosensors_cis-trans_TCA01_Experiment_50uM IAA
treatment_yWL162.fcs       AFB2
# # 1Pat-07142023-Biosensors_cis-trans_TCA01_Experiment_50uM IAA
treatment_yWL185.fcs       TIR1
# # 1Pat-07142023-Biosensors_cis-trans_TCA01_Experiment_50uM IAA
treatment_yWL186.fcs       AFB2
# # 1Pat-07142023-Biosensors_cis-trans_TCA01_Experiment_50uM IAA
treatment_yWL209.fcs       TIR1
# # 1Pat-07142023-Biosensors_cis-trans_TCA01_Experiment_50uM IAA
treatment_yWL210.fcs       AFB2
# #
design_yeast_treatment
# # 1Pat-07142023-Biosensors_cis-trans_TCA01_Experiment_50uM IAA
treatment_yWL161.fcs TIR1 single-fusion (W303)-50uM IAA
# # 1Pat-07142023-Biosensors_cis-trans_TCA01_Experiment_50uM IAA
treatment_yWL162.fcs AFB2 single-fusion (W303)-50uM IAA
# # 1Pat-07142023-Biosensors_cis-trans_TCA01_Experiment_50uM IAA
treatment_yWL185.fcs   TIR1 dual-fusion (W303)-50uM IAA
# # 1Pat-07142023-Biosensors_cis-trans_TCA01_Experiment_50uM IAA
treatment_yWL186.fcs   AFB2 dual-fusion (W303)-50uM IAA
# # 1Pat-07142023-Biosensors_cis-trans_TCA01_Experiment_50uM IAA
treatment_yWL209.fcs TIR1 dual-fusion (YPH499)-50uM IAA
# # 1Pat-07142023-Biosensors_cis-trans_TCA01_Experiment_50uM IAA
treatment_yWL210.fcs AFB2 dual-fusion (YPH499)-50uM IAA
```

####Figure 5C and Supporting Figure S5: Comparison of biosensor designs in different yeast strains, before and after the treatments

```
#Color codes: Venus #f7cb44b2, mScarlet #b8627db2, ratio #593d9cb2, control
#042333b2

plate_07142023_3readsbefore_after <-
summarizeFlow(aplate_07142023_3readsbefore_after, channel = c("BL1.A",
"YL1.A"), gated = TRUE)
# # [1] "Summarizing all events..."


plate_07142023_3readsbefore_after$treatment <-
factor(plate_07142023_3readsbefore_after$treatment, levels = c("control",
"50uM IAA"))
plate_07142023_3readsbefore_after$before_after <-
factor(plate_07142023_3readsbefore_after$before_after, levels = c("before",
"after"))

boxRatio_expo3reads_norm <- ggplot(plate_07142023_3readsbefore_after,
aes(x=before_after, y=(BL1.Amean/mean(BL1.Amean))/
(YL1.Amean/mean(YL1.Amean)), fill=treatment, shape = treatment)) +
  geom_boxplot() +
```

```
  scale_color_manual(values=c("#b8627dff","#6b4596ff"))  +
  scale_fill_manual(values=c("#b8627dff","#6b4596ff")) +
  geom_jitter(aes(color=treatment),
position=position_jitterdodge(jitter.width = 0.1, dodge.width = 0.7)) +
  theme_classic() +
  theme(legend.position = "bottom", panel.grid.major = element_line(size =
0.3, linetype = 'solid', colour = "white"), panel.grid.minor =
element_line(size = 0.3, linetype = 'solid', colour = "white"), axis.text.x =
element_text(size=10, angle=0), axis.title = element_text(face="bold", size =
12), legend.text = element_text(size = 10)) +
  facet_wrap(~factor(design_yeast, c("AFB2 dual-fusion (W303)", "AFB2 dual-
fusion (YPH499)", "AFB2 single-fusion (W303)", "TIR1 dual-fusion (W303)",
"TIR1 dual-fusion (YPH499)", "TIR1 single-fusion (W303)")), scales =
"free_y") + xlab("Treatment") + ylab("Normalized Venus/mScarlet-I") +
  stat_compare_means(method = "t.test", label = "p.format", hide.ns = FALSE,
size = 3.5, label.y = 0.43) +
  labs(fill = "Treatment", color = "Treatment", shape = "Treatment")

boxRatio_expo3reads_norm
```

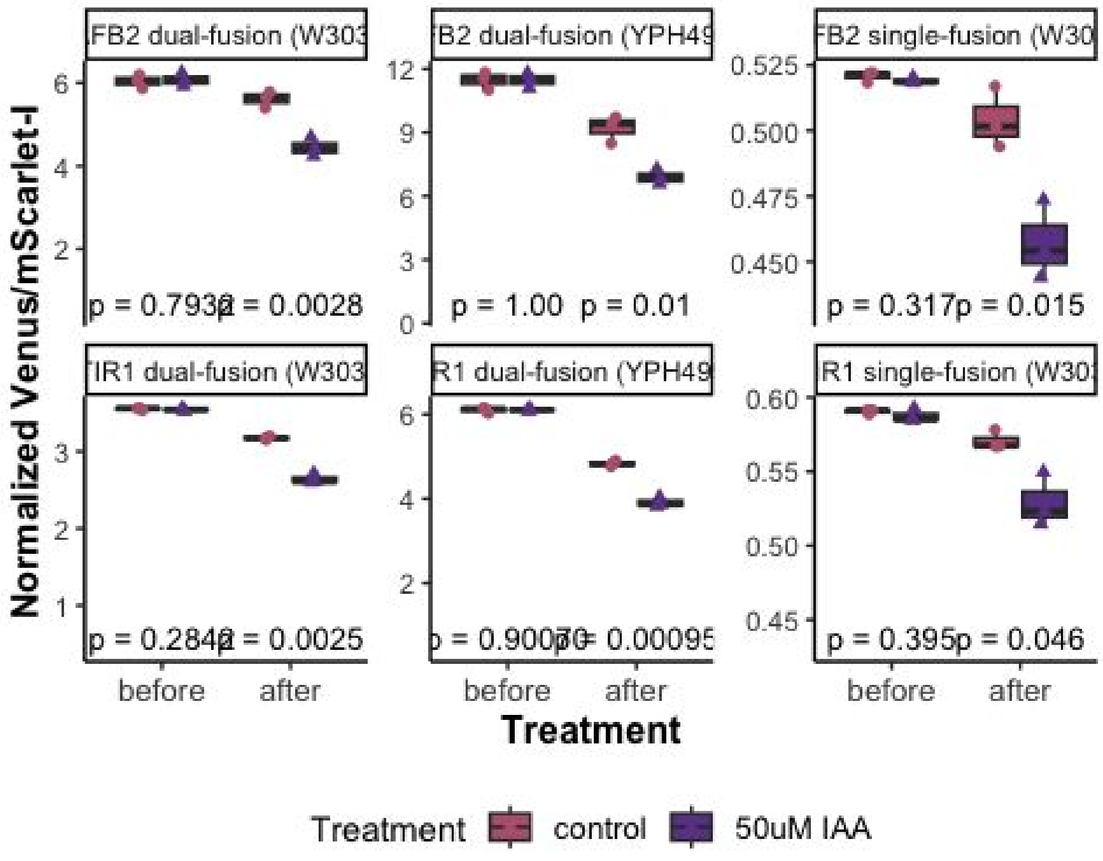


```
#ggsave("boxRatio_expo3reads_norm.png",  width = 6, height = 5)
```

```
boxRatio_expo3reads_norm_afteronly <-
ggplot(subset(plate_07142023_3readsbefore_after, before_after =="after"),
aes(x=design_yeast, y=(BL1.Amean/mean(BL1.Amean))/
(YL1.Amean/mean(YL1.Amean))/15, fill=treatment, shape = treatment)) +
geom_boxplot() + scale_fill_manual(values=c("#b8627dff","#6b4596ff"))  +
scale_color_manual(values=c("#b8627dff","#6b4596ff")) +
geom_jitter(aes(color=treatment), position=position_jitterdodge(jitter.width
= 0.1, dodge.width = 0.7), size = 4)+ theme_classic() + theme(legend.position
= c(0.9, 0.7), axis.title.y = element_text(face="bold", size = 12),
axis.title.x = element_text(face="bold", size = 12), axis.text.y =
element_text(size = 9),axis.text.x = element_text(size = 9),
legend.title=element_text(size=10, face ="bold"),
legend.text=element_text(size=10), legend.background =
element_rect(fill="#E2D9E6", size=0.5, linetype="solid")) +
  xlab("Biosensor designs (strains)") + ylab("Normalized Venus/mScarlet-I")
+
  scale_x_discrete(labels=c(
    "AFB2 dual-fusion (W303)" = "AFB2\ndual-fusion\n(W303)",
    "AFB2 dual-fusion (YPH499)" = "AFB2\ndual-fusion\n(YPH499)",
    "AFB2 single-fusion (W303)" ="AFB2\nsingle-fusion\n(W303)",
    "TIR1 dual-fusion (W303)" = "TIR1\ndual-fusion\n(W303)",
    "TIR1 dual-fusion (YPH499)" = "TIR1\ndual-fusion\n(YPH499)",
    "TIR1 single-fusion (W303)" = "TIR1\nsingle-fusion\n(W303)")) +
  #annotate(geom="text", x=1, y=15, label="p=0.046") + annotate(geom="text",
x=2, y=15, label="p=0.046") + annotate(geom="text", x=3, y=15,
label="p=0.0025")+ annotate(geom="text", x=4, y=15, label="p=0.0028")+
annotate(geom="text", x=5, y=15, label="p=0.00095")+ annotate(geom="text",
x=6, y=15, label="p=0.01") +
  stat_compare_means(method = "t.test", label = "..p.format..", hide.ns =
TRUE, size = 4, label.y = 0.68)

  #geom_signif(comparisons = list(c("compact", "midsize"), c("minivan",
"suv")), map_signif_level = TRUE, textsize=10, family="serif")

boxRatio_expo3reads_norm_afteronly
```

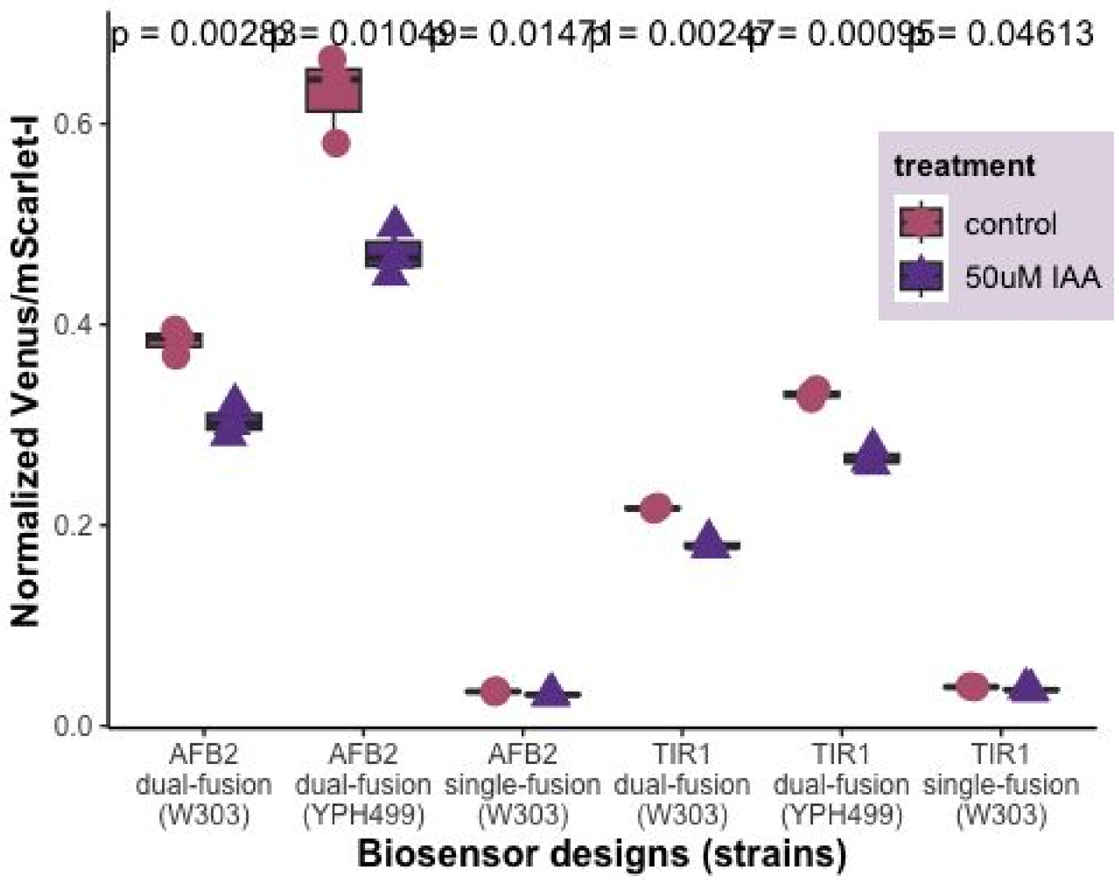


```
#ggsave("Supplement4.pdf",  width = 8, height = 5)
```

Figure 5B: Foldchange between the 50 µM auxin treatment and solvent control for each biosensor design

```
plate_07142023_3readsbefore_after <- plate_07142023_3readsbefore_after %>%
    dplyr::filter(before_after == "after") %>%
    mutate(Ratio = BL1.Amean/YL1.Amean) %>%
    group_by(design, yeast) %>%
    mutate(foldchange_treatment = (mean(Ratio[treatment == "control"]) -
Ratio)/mean(Ratio[treatment ==
        "control"]))


fc <- ggplot(plate_07142023_3readsbefore_after |>
    dplyr::filter(treatment != "control"), aes(x = factor(design_yeast), y =
foldchange_treatment,
    fill = design_yeast, colour = design_yeast)) + geom_boxplot(alpha = 0.9)
+ scale_fill_manual(values = c("#593d9cff",
    "#7e4e90ff", "#a65c85ff", "#cc6a70ff", "#eb8055ff", "#f9a242ff",
"#f7cb44ff")) +
```

```
    scale_color_manual(values = c("#593d9cff", "#7e4e90ff", "#a65c85ff",
"#cc6a70ff",
        "#eb8055ff", "#f9a242ff", "#f7cb44ff")) + theme_classic() +
theme(axis.title.y = element_text(face = "bold",
    size = 12), axis.title.x = element_text(face = "bold", size = 12),
axis.text.y = element_text(size = 10),
    axis.text.x = element_text(size = 9), legend.title = element_text(size =
12,
        face = "bold"), legend.text = element_text(size = 12),
legend.position = "none") +
    xlab("Biosensor designs (strains)") + ylab("Fold Change") +
scale_x_discrete(labels = c(`AFB2 dual-fusion (W303)` = "AFB2\ndual-
fusion\n(W303)",
    `AFB2 dual-fusion (YPH499)` = "AFB2\ndual-fusion\n(YPH499)", `AFB2
single-fusion (W303)` = "AFB2\nsingle-fusion\n(W303)",
    `TIR1 dual-fusion (W303)` = "TIR1\ndual-fusion\n(W303)", `TIR1 dual-
fusion (YPH499)` = "TIR1\ndual-fusion\n(YPH499)",
    `TIR1 single-fusion (W303)` = "TIR1\nsingle-fusion\n(W303)"))
fc
```

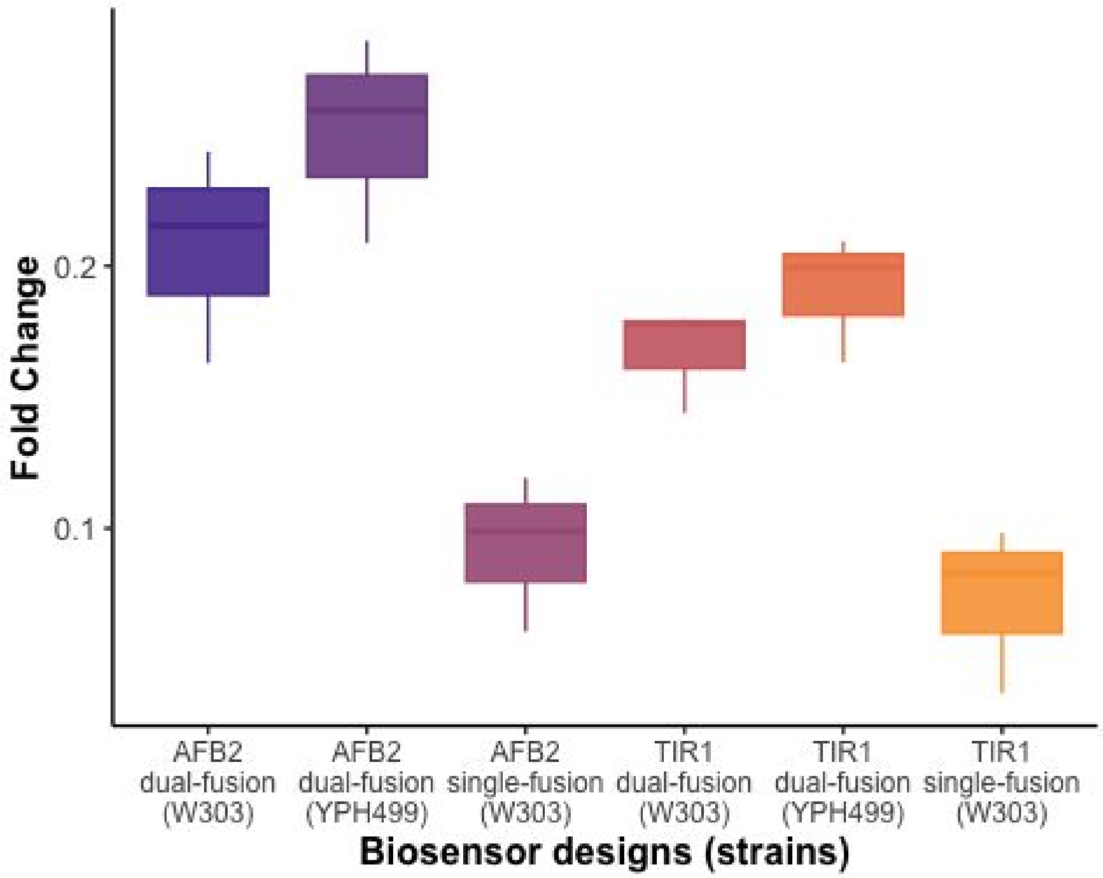

```r
fig5AB <- ggarrange(norm_ratio, fc, ncol = 1, nrow = 2, labels = c("A", "B"),
heights = c(1,
    0.7))
# fig5AB

fig5C <- ggarrange(boxRatio_expo3reads_norm, ncol = 1, nrow = 1, labels =
c("C"))
# fig5C

fig5ABC <- ggarrange(fig5AB, fig5C, nrow = 1, ncol = 2, widths = c(0.8, 1))
fig5ABC
```

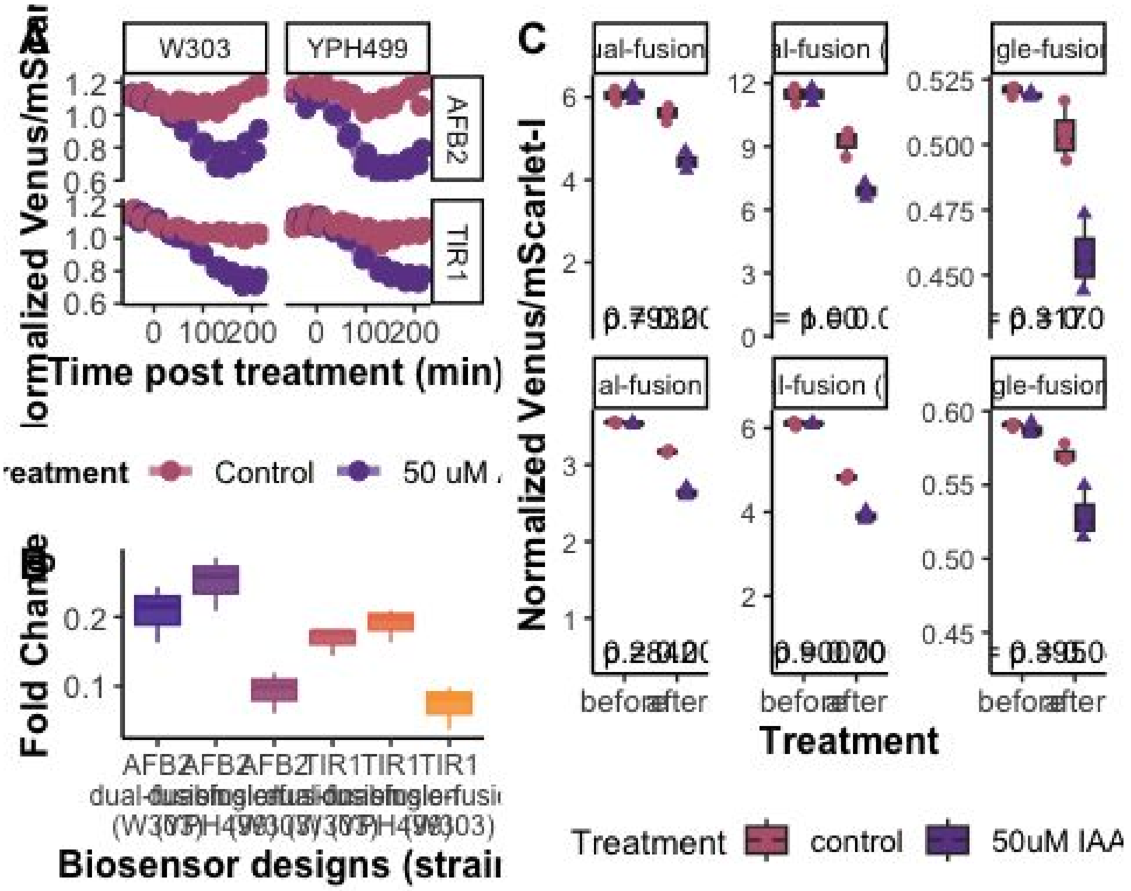


```r
# ggsave('Figure5.png', width = 11, height = 6.5)
```

Supporting Figure S6: Auxin dose-response assay measured by the AFB2 dual-fusion biosensor

```r
plate_08052022 <- read.plateSet(path = "Data for
publication/08052022_yWL210_DRA-LCMS-R2/Alldata/",
    pattern = "DRA*")
```

```
annotation <- createAnnotation(yourFlowSet = plate_08052022)
write.csv(annotation, "Data for publication/08052022_yWL210_DRA-LCMS-
R2/08052022_alldata_annotation.csv")

annotation <- read.csv("Data for publication/08052022_yWL210_DRA-LCMS-
R2/08052022_alldata_annotation.csv")

aplate_08052022 <- annotateFlowSet(yourFlowSet = plate_08052022,
annotation_df = annotation,
    mergeBy = "name")
head(rownames(pData(aplate_08052022)))
# # [1] "1Pat-08052022-Biosensor_LCMS_rep2-DRA01_DRA-LCMS-R2_Exponential_0
uM.fcs"
# # [2] "1Pat-08052022-Biosensor_LCMS_rep2-DRA01_DRA-LCMS-
R2_Exponential_0.0064 uM.fcs"
# # [3] "1Pat-08052022-Biosensor_LCMS_rep2-DRA01_DRA-LCMS-
R2_Exponential_0.032 uM.fcs"
# # [4] "1Pat-08052022-Biosensor_LCMS_rep2-DRA01_DRA-LCMS-R2_Exponential_0.16
uM.fcs"
# # [5] "1Pat-08052022-Biosensor_LCMS_rep2-DRA01_DRA-LCMS-R2_Exponential_0.8
uM.fcs"
# # [6] "1Pat-08052022-Biosensor_LCMS_rep2-DRA01_DRA-LCMS-R2_Exponential_100
uM.fcs"
head(pData(aplate_08052022))
# #
name
# # 1Pat-08052022-Biosensor_LCMS_rep2-DRA01_DRA-LCMS-R2_Exponential_0 uM.fcs
1Pat-08052022-Biosensor_LCMS_rep2-DRA01_DRA-LCMS-R2_Exponential_0 uM.fcs
# # 1Pat-08052022-Biosensor_LCMS_rep2-DRA01_DRA-LCMS-R2_Exponential_0.0064
uM.fcs 1Pat-08052022-Biosensor_LCMS_rep2-DRA01_DRA-LCMS-R2_Exponential_0.0064
uM.fcs
# # 1Pat-08052022-Biosensor_LCMS_rep2-DRA01_DRA-LCMS-R2_Exponential_0.032
uM.fcs   1Pat-08052022-Biosensor_LCMS_rep2-DRA01_DRA-LCMS-
R2_Exponential_0.032 uM.fcs
# # 1Pat-08052022-Biosensor_LCMS_rep2-DRA01_DRA-LCMS-R2_Exponential_0.16
uM.fcs     1Pat-08052022-Biosensor_LCMS_rep2-DRA01_DRA-LCMS-
R2_Exponential_0.16 uM.fcs
# # 1Pat-08052022-Biosensor_LCMS_rep2-DRA01_DRA-LCMS-R2_Exponential_0.8
uM.fcs       1Pat-08052022-Biosensor_LCMS_rep2-DRA01_DRA-LCMS-
R2_Exponential_0.8 uM.fcs
# # 1Pat-08052022-Biosensor_LCMS_rep2-DRA01_DRA-LCMS-R2_Exponential_100
uM.fcs       1Pat-08052022-Biosensor_LCMS_rep2-DRA01_DRA-LCMS-
R2_Exponential_100 uM.fcs
# #
folder
# # 1Pat-08052022-Biosensor_LCMS_rep2-DRA01_DRA-LCMS-R2_Exponential_0 uM.fcs
DRA01
# # 1Pat-08052022-Biosensor_LCMS_rep2-DRA01_DRA-LCMS-R2_Exponential_0.0064
uM.fcs  DRA01
# # 1Pat-08052022-Biosensor_LCMS_rep2-DRA01_DRA-LCMS-R2_Exponential_0.032
```

```
uM.fcs   DRA01
# # 1Pat-08052022-Biosensor_LCMS_rep2-DRA01_DRA-LCMS-R2_Exponential_0.16
uM.fcs    DRA01
# # 1Pat-08052022-Biosensor_LCMS_rep2-DRA01_DRA-LCMS-R2_Exponential_0.8
uM.fcs     DRA01
# # 1Pat-08052022-Biosensor_LCMS_rep2-DRA01_DRA-LCMS-R2_Exponential_100
uM.fcs     DRA01
# #
X
# # 1Pat-08052022-Biosensor_LCMS_rep2-DRA01_DRA-LCMS-R2_Exponential_0 uM.fcs
1
# # 1Pat-08052022-Biosensor_LCMS_rep2-DRA01_DRA-LCMS-R2_Exponential_0.0064
uM.fcs 2
# # 1Pat-08052022-Biosensor_LCMS_rep2-DRA01_DRA-LCMS-R2_Exponential_0.032
uM.fcs  3
# # 1Pat-08052022-Biosensor_LCMS_rep2-DRA01_DRA-LCMS-R2_Exponential_0.16
uM.fcs   4
# # 1Pat-08052022-Biosensor_LCMS_rep2-DRA01_DRA-LCMS-R2_Exponential_0.8
uM.fcs    5
# # 1Pat-08052022-Biosensor_LCMS_rep2-DRA01_DRA-LCMS-R2_Exponential_100
uM.fcs    6
# #
treatment
# # 1Pat-08052022-Biosensor_LCMS_rep2-DRA01_DRA-LCMS-R2_Exponential_0 uM.fcs
0 uM
# # 1Pat-08052022-Biosensor_LCMS_rep2-DRA01_DRA-LCMS-R2_Exponential_0.0064
uM.fcs 0.0064 uM
# # 1Pat-08052022-Biosensor_LCMS_rep2-DRA01_DRA-LCMS-R2_Exponential_0.032
uM.fcs   0.032 uM
# # 1Pat-08052022-Biosensor_LCMS_rep2-DRA01_DRA-LCMS-R2_Exponential_0.16
uM.fcs     0.16 uM
# # 1Pat-08052022-Biosensor_LCMS_rep2-DRA01_DRA-LCMS-R2_Exponential_0.8
uM.fcs       0.8 uM
# # 1Pat-08052022-Biosensor_LCMS_rep2-DRA01_DRA-LCMS-R2_Exponential_100
uM.fcs       100 uM
# #
reading
# # 1Pat-08052022-Biosensor_LCMS_rep2-DRA01_DRA-LCMS-R2_Exponential_0 uM.fcs
1
# # 1Pat-08052022-Biosensor_LCMS_rep2-DRA01_DRA-LCMS-R2_Exponential_0.0064
uM.fcs       1
# # 1Pat-08052022-Biosensor_LCMS_rep2-DRA01_DRA-LCMS-R2_Exponential_0.032
uM.fcs        1
# # 1Pat-08052022-Biosensor_LCMS_rep2-DRA01_DRA-LCMS-R2_Exponential_0.16
uM.fcs         1
# # 1Pat-08052022-Biosensor_LCMS_rep2-DRA01_DRA-LCMS-R2_Exponential_0.8
uM.fcs          1
# # 1Pat-08052022-Biosensor_LCMS_rep2-DRA01_DRA-LCMS-R2_Exponential_100
uM.fcs          1
# #
```

```
dose
# # 1Pat-08052022-Biosensor_LCMS_rep2-DRA01_DRA-LCMS-R2_Exponential_0 uM.fcs
0.0e+00
# # 1Pat-08052022-Biosensor_LCMS_rep2-DRA01_DRA-LCMS-R2_Exponential_0.0064
uM.fcs 6.4e-03
# # 1Pat-08052022-Biosensor_LCMS_rep2-DRA01_DRA-LCMS-R2_Exponential_0.032
uM.fcs  3.2e-02
# # 1Pat-08052022-Biosensor_LCMS_rep2-DRA01_DRA-LCMS-R2_Exponential_0.16
uM.fcs   1.6e-01
# # 1Pat-08052022-Biosensor_LCMS_rep2-DRA01_DRA-LCMS-R2_Exponential_0.8
uM.fcs    8.0e-01
# # 1Pat-08052022-Biosensor_LCMS_rep2-DRA01_DRA-LCMS-R2_Exponential_100
uM.fcs    1.0e+02
# #
collection
# # 1Pat-08052022-Biosensor_LCMS_rep2-DRA01_DRA-LCMS-R2_Exponential_0 uM.fcs
end
# # 1Pat-08052022-Biosensor_LCMS_rep2-DRA01_DRA-LCMS-R2_Exponential_0.0064
uM.fcs        end
# # 1Pat-08052022-Biosensor_LCMS_rep2-DRA01_DRA-LCMS-R2_Exponential_0.032
uM.fcs         end
# # 1Pat-08052022-Biosensor_LCMS_rep2-DRA01_DRA-LCMS-R2_Exponential_0.16
uM.fcs          end
# # 1Pat-08052022-Biosensor_LCMS_rep2-DRA01_DRA-LCMS-R2_Exponential_0.8
uM.fcs           end
# # 1Pat-08052022-Biosensor_LCMS_rep2-DRA01_DRA-LCMS-R2_Exponential_100
uM.fcs           end
# #
before_after
# # 1Pat-08052022-Biosensor_LCMS_rep2-DRA01_DRA-LCMS-R2_Exponential_0 uM.fcs
before
# # 1Pat-08052022-Biosensor_LCMS_rep2-DRA01_DRA-LCMS-R2_Exponential_0.0064
uM.fcs       before
# # 1Pat-08052022-Biosensor_LCMS_rep2-DRA01_DRA-LCMS-R2_Exponential_0.032
uM.fcs        before
# # 1Pat-08052022-Biosensor_LCMS_rep2-DRA01_DRA-LCMS-R2_Exponential_0.16
uM.fcs         before
# # 1Pat-08052022-Biosensor_LCMS_rep2-DRA01_DRA-LCMS-R2_Exponential_0.8
uM.fcs          before
# # 1Pat-08052022-Biosensor_LCMS_rep2-DRA01_DRA-LCMS-R2_Exponential_100
uM.fcs          before

plate_08052022_sum <- summarizeFlow(aplate_08052022, channel = c("BL1.A",
"YL1.A"),
    gated = TRUE)
# # [1] "Summarizing all events..."

# dat_sumGr_08052022 <- summarizeFlow(aplate_08052022, gated = TRUE, channel
=
# 'BL1.A')
```

```r
# pulling data of Red fluorescent dat_sumRd_08052022 <-
# summarizeFlow(aplate_08052022, gated = TRUE, channel = 'YL1.A')

# dat_sumGr_08052022$YL1.Amean <- dat_sumRd_08052022$YL1.Amean

# levels(plate_08052022_sum$dose) plate_08052022_sum$dose <-
# factor(plate_08052022_sum$dose, levels = c('0 uM', '0.0064 uM', '0.032 uM',
# '0.16 uM', '0.8 uM', '4 uM', '20 uM', '100 uM'))

scurve <- ggplot(data = plate_08052022_sum, color = factor(reading), aes(x =
as.numeric(str_remove(treatment,
    " uM")), y = YL1.Amean/BL1.Amean)) + geom_point(aes(color = reading),
size = 3,
    shape = 19) + xlab("Dose of Auxin (μM)") + ylab("mScarlet-I/Venus") +
scale_x_log10(labels = scales::number) +
    theme_classic() + theme(axis.title = element_text(size = 12, face =
"bold"),
    axis.text = element_text(size = 10), legend.title = element_text(size =
10),
    legend.text = element_text(size = 10), legend.position = "bottom") +
scale_color_viridis_c(option = "plasma",
    alpha = 0.7, begin = 0, end = 0.8)

scurve
```

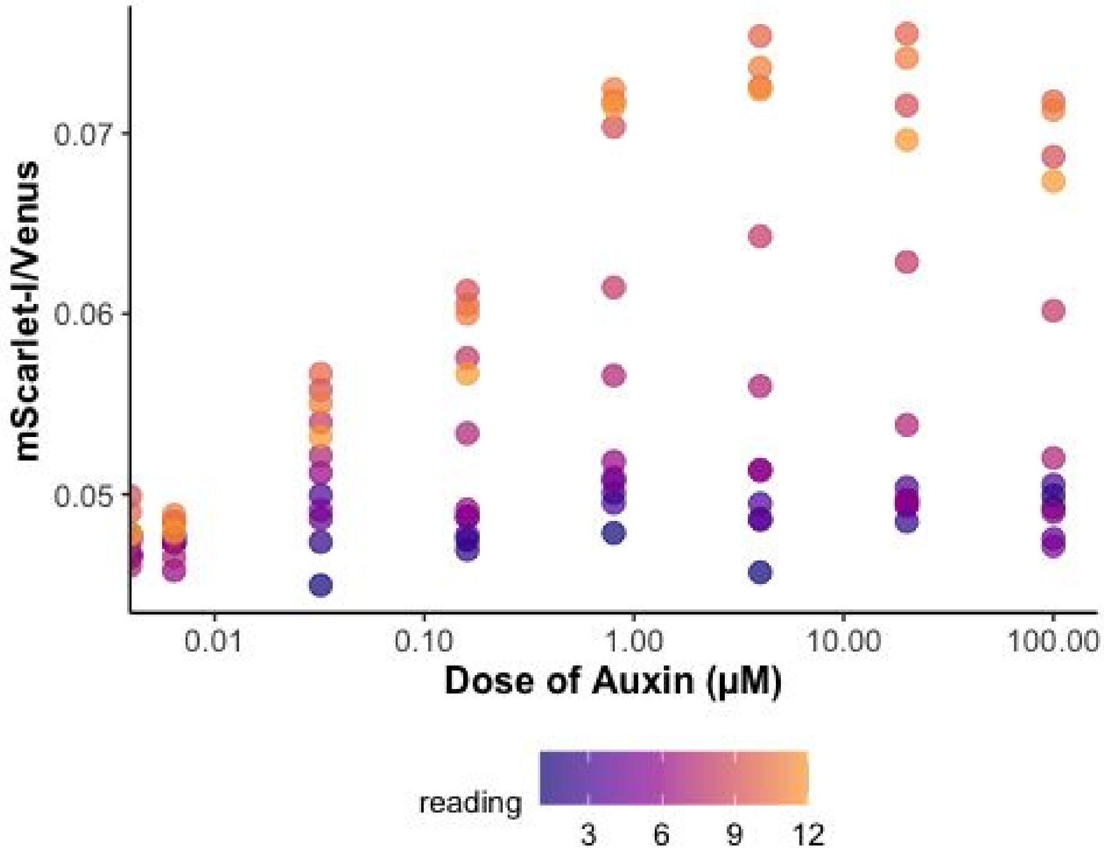


```
ratio080522_wrapread <- ggplot(data = plate_08052022_sum, color =
factor(reading),
    aes(x = as.numeric(str_remove(treatment, " uM")), y =
YL1.Amean/BL1.Amean)) +
    geom_point(aes(color = reading), size = 3, shape = 19) +
geom_line(aes(color = reading),
    size = 2) + xlab("Dose of Auxin (μM)") + ylab("mScarlet-I/Venus") +
scale_x_log10(labels = scales::number) +
    facet_wrap(~reading) + scale_color_viridis_c(option = "plasma", alpha =
0.7,
    begin = 0, end = 0.8) + theme_classic() + theme(axis.title =
element_text(size = 12,
    face = "bold"), axis.text = element_text(size = 10), axis.text.x =
element_text(angle = 90),
    legend.title = element_text(size = 10), legend.text = element_text(size =
10),
    legend.position = "none")

ratio080522_wrapread
```

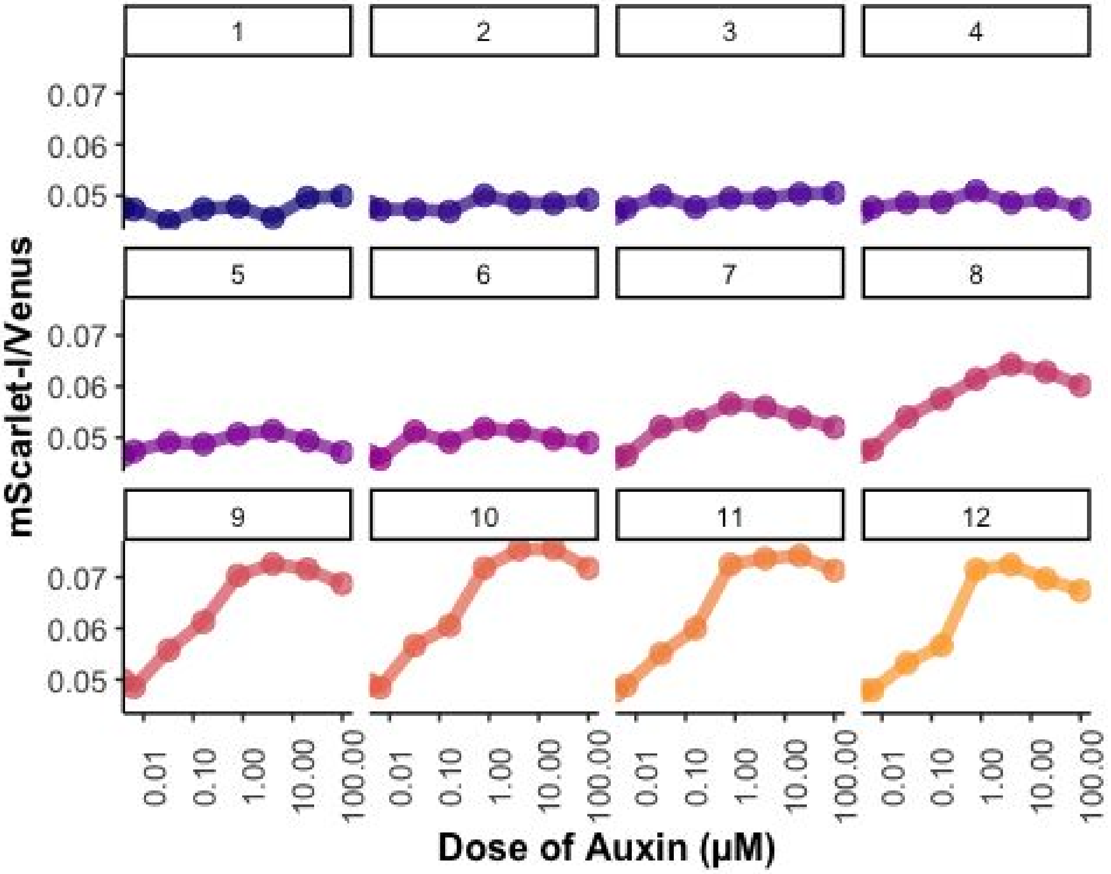


```
DRAplot <- ggarrange(scurve, ratio080522_wrapread, labels = c("A", "B"), nrow
= 1,
    ncol = 2)
DRAplot
```

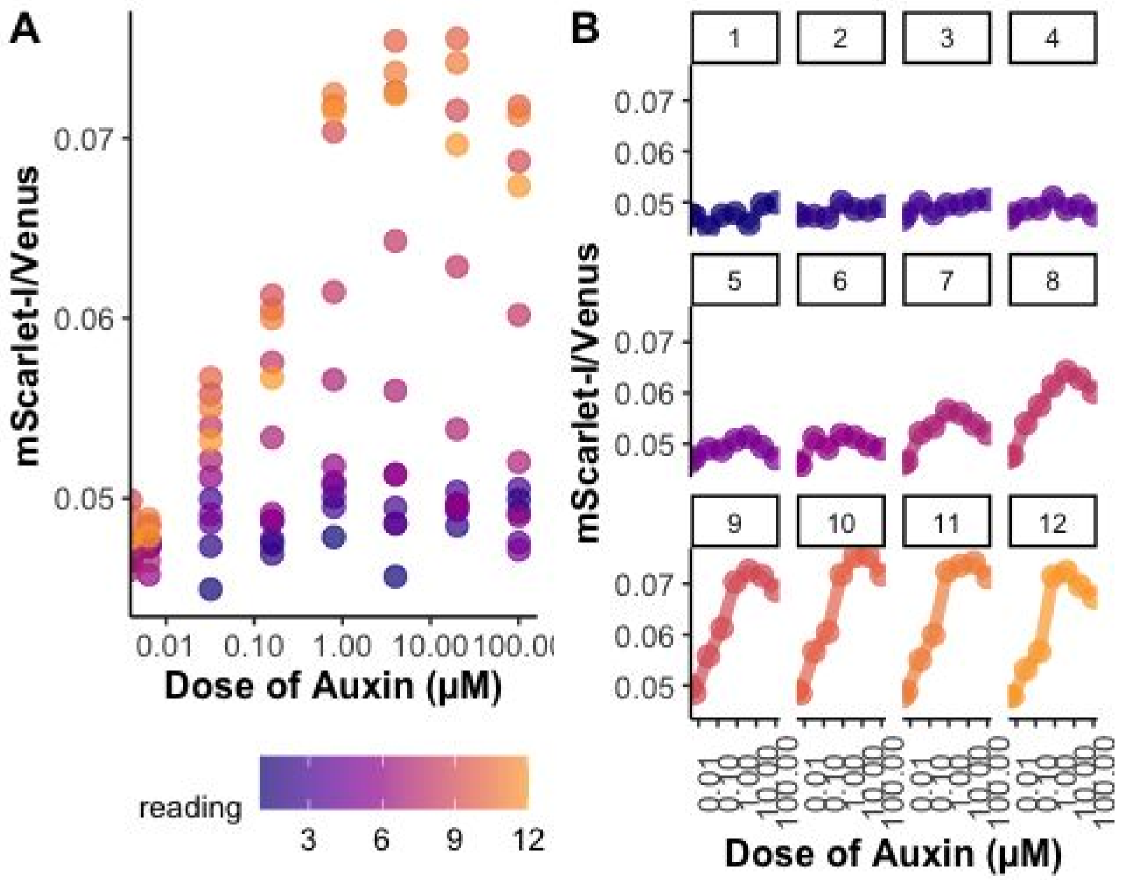


```
# ggsave('Supplement5.pdf',width = 9, height = 4)
```

Figure 6: Dose-response models of biosensors

```
library(tidyverse)
library(drc)

model.LL4_08052022 <- drm(YL1.Amean/BL1.Amean ~ dose, data =
subset(plate_08052022_sum,
    reading == "10"), fct = LL.4(names = c("Slope", "Lower Limit", "Upper
Limit",
    "ED50")))

plot(model.LL4_08052022, type = "all", col = "black", lty = 1, lwd = 3, xlab
= "Extracellular concentrations of IAA (uM)",
    ylab = "mScarlet-I/Venus Ratio")


reading10_08052022 <- drm(YL1.Amean/BL1.Amean ~ dose, data =
subset(plate_08052022_sum,
    reading == "10"), fct = LL.4())
```

```
plot(reading10_08052022, broken = TRUE, add = TRUE, type = "none", col = 2,
lty = 2)

## Add confidence region for the models.
plot(reading10_08052022, broken = TRUE, type = "confidence", col = "blue",
add = TRUE)
```

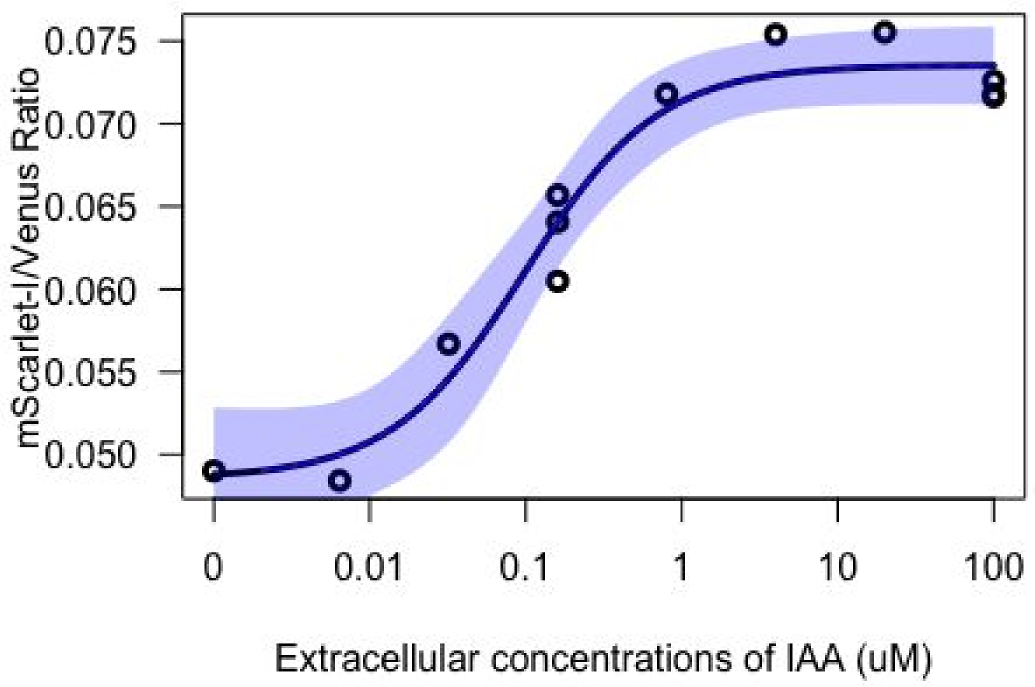


```
pm_08052022 <- expand.grid(treatment = exp(seq(log(0.001), log(100), length =
1000)))
pm_08052022 <- cbind(pm_08052022, predict(model.LL4_08052022, newdata =
pm_08052022,
    interval = "confidence"))

DRAplot_08052022 <- ggplot(data = subset(plate_08052022_sum, reading ==
"10"), aes(x = dose,
    y = YL1.Amean/BL1.Amean)) + scale_x_log10() + geom_ribbon(data =
pm_08052022,
    aes(x = treatment, y = Prediction, ymin = Lower, ymax = Upper), alpha =
0.4) +
```

```
    geom_line(data = pm_08052022, aes(x = treatment, y = Prediction), size =
1.2) +
    xlab("Auxin Treatment (µM)") + ylab("mScarlet-I/Venus") + scale_x_log10()
+
    geom_point(size = 4, color = "#403891b2") + theme_classic() +
theme(legend.position = "none",
    axis.title.y = element_text(size = 20, face = "bold"), axis.title.x =
element_text(size = 20,
        face = "bold"), axis.text = element_text(size = 18), legend.title =
element_text(size = 18,
        face = "bold"), legend.text = element_text(size = 18)) +
scale_y_continuous(breaks = seq(0,
    0.14, 0.01))
# # Scale for x is already present.
# # Adding another scale for x, which will replace the existing scale.


DRAplot_08052022
```

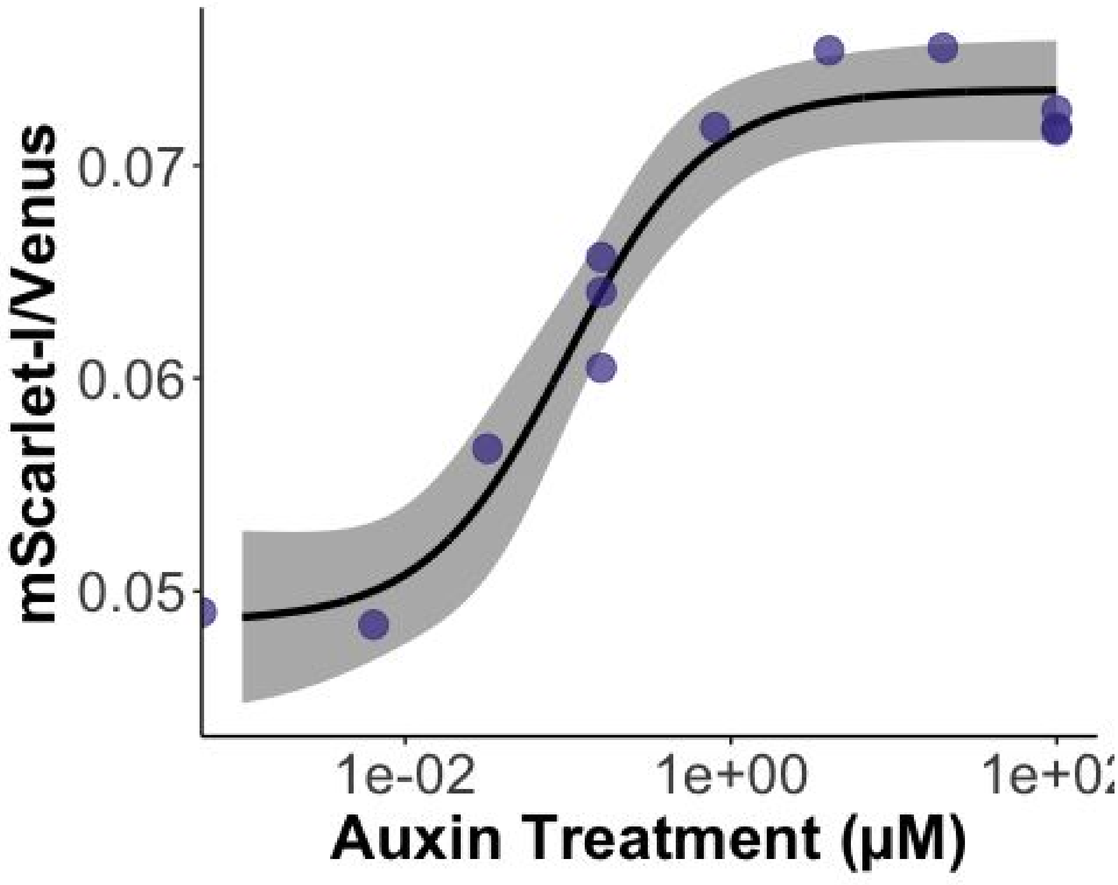

Figure 7: Auxin quantification by AFB2 dual-fustion Biosensor vs LC-MS

```
lcms_data <- read_csv("Data for publication/LCMS_data_forplot.csv")
# # New names:
```

```
# # Rows: 14 Columns: 13
# # ── Column specification
# # ──────────────────────────────────────────────────────── Delimiter: ","
chr
# # (3): Sample, dilution, experiment dbl (10): ...1, Observe_IAA_in_uM,
# # peak_response, Dose_IAA, set, Green, Red,...
# # ℹ Use `spec()` to retrieve the full column specification for this data. ℹ
# # Specify the column types or set `show_col_types = FALSE` to quiet this
message.
# # • `` -> `...1`
```

Model intracellular concentration versus dose of auxin

Begin with a 4-parameter log-logistic model

```
intra_dose4 <- drm(Observe_IAA_in_uM ~ Dose_IAA, data = lcms_data, fct =
LL.4())
summary(intra_dose4)
# #
# # Model fitted: Log-logistic (ED50 as parameter) (4 parms)
# #
# # Parameter estimates:
# #
# #                  Estimate  Std. Error t-value p-value
# # b:(Intercept) -5.9718e-01  1.3912e-01 -4.2925 0.00158 **
# # c:(Intercept)  6.8864e-02  4.6432e-01  0.1483 0.88505
# # d:(Intercept)  9.3514e+01  1.9575e+02  0.4777 0.64311
# # e:(Intercept)  1.3879e+04  6.1640e+04  0.2252 0.82639
# # ---
# # Signif. codes:  0 '***' 0.001 '**' 0.01 '*' 0.05 '.' 0.1 ' ' 1
# #
# # Residual standard error:
# #
# #  1.120679 (10 degrees of freedom)
plot(intra_dose4)
```

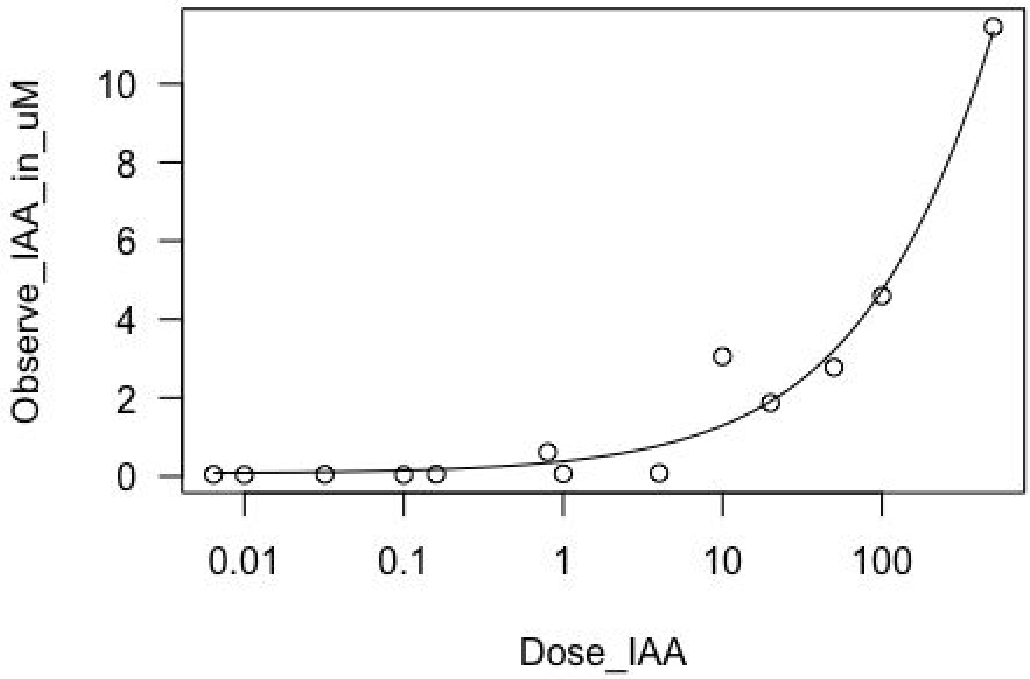

Compare other model forms

```
mselect(intra_dose4, list(LL2.4(), LL.5(), LL2.5(), LL.3(), lnormal(),
L.4()), linreg = TRUE,
    icfct = BIC)
# #            logLik       IC Lack of fit  Res var
# # LL.3    -19.09453 48.74528   0.9990355 1.140053
# # Quad    -20.17634 50.90891          NA 1.330585
# # LL2.4   -19.06996 51.33521   0.9982747 1.249665
# # LL.4    -19.10492 51.40512   0.9981866 1.255921
# # lnormal -19.56407 52.32342   0.9967209 1.341062
# # Cubic   -19.61321 52.42170          NA 1.350510
# # LL2.5   -19.03771 53.90976   0.9969071 1.382133
# # L.4     -20.35741 53.91010   0.9925932 1.501998
# # LL.5    -19.16546 54.16526   0.9963648 1.407589
# # Lin     -23.34430 54.60577          NA 1.917790
```

While a 3-parameter log-logistic model has the lowest value of AIC it does assume that the intracellular auxin concentration goes to zero when extracellular auxin is zero, which we and others have shown to not be true. Therefore we will use the 4-parameter log-logistic model as hypothesized but with a slight change in parameterization.

```
intra_dose <- drm(Observe_IAA_in_uM ~ Dose_IAA, data = lcms_data, fct =
LL2.4())
summary(intra_dose)
# #
# # Model fitted: Log-logistic (log(ED50) as parameter) (4 parms)
# #
# # Parameter estimates:
# #
# #                   Estimate Std. Error t-value  p-value
# # b:(Intercept)  -0.573353   0.152571 -3.7580 0.003735 **
# # c:(Intercept)   0.050624   0.476907  0.1062 0.917562
# # d:(Intercept) 175.849512 780.660654  0.2253 0.826316
# # e:(Intercept)  10.885508   9.140423  1.1909 0.261186
# # ---
# # Signif. codes:  0 '***' 0.001 '**' 0.01 '*' 0.05 '.' 0.1 ' ' 1
# #
# # Residual standard error:
# #
# #  1.117884 (10 degrees of freedom)
plot(intra_dose)
```

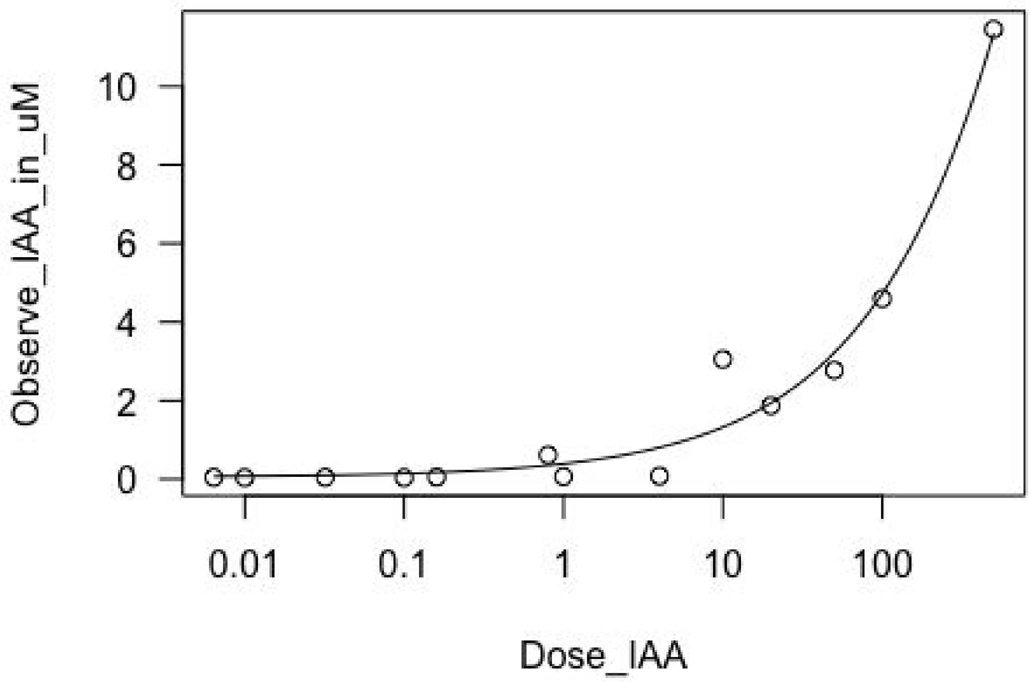


```
# predictions and confidence intervals.
intra_dose.pred <- expand.grid(conc = exp(seq(log(max(lcms_data$Dose_IAA)),
```

```
log(min(lcms_data$Dose_IAA)),
    length = 100)))
# new data with predictions
pm <- predict(intra_dose, newdata = intra_dose.pred, interval = "confidence")
intra_dose.pred$p <- pm[, 1]
intra_dose.pred$pmin <- pm[, 2]
intra_dose.pred$pmax <- pm[, 3]


ggplot(data = lcms_data, mapping = aes(x = Dose_IAA, y = Observe_IAA_in_uM,
color = experiment)) +
    geom_point(alpha = 0.5) + geom_ribbon(data = intra_dose.pred, aes(x =
conc, y = p,
    ymin = pmin, ymax = pmax), alpha = 0.2, inherit.aes = FALSE) +
geom_line(data = intra_dose.pred,
    aes(x = conc, y = p), inherit.aes = FALSE) + scale_x_log10() +
guides(color = "none") +
    labs(x = "Exogenous auxin (μM)", y = "Intracellular\nauxin (μM)") +
scale_color_viridis_d(begin = 0.3,
    end = 0.6, option = "plasma") -> intra_dose
intra_dose
```

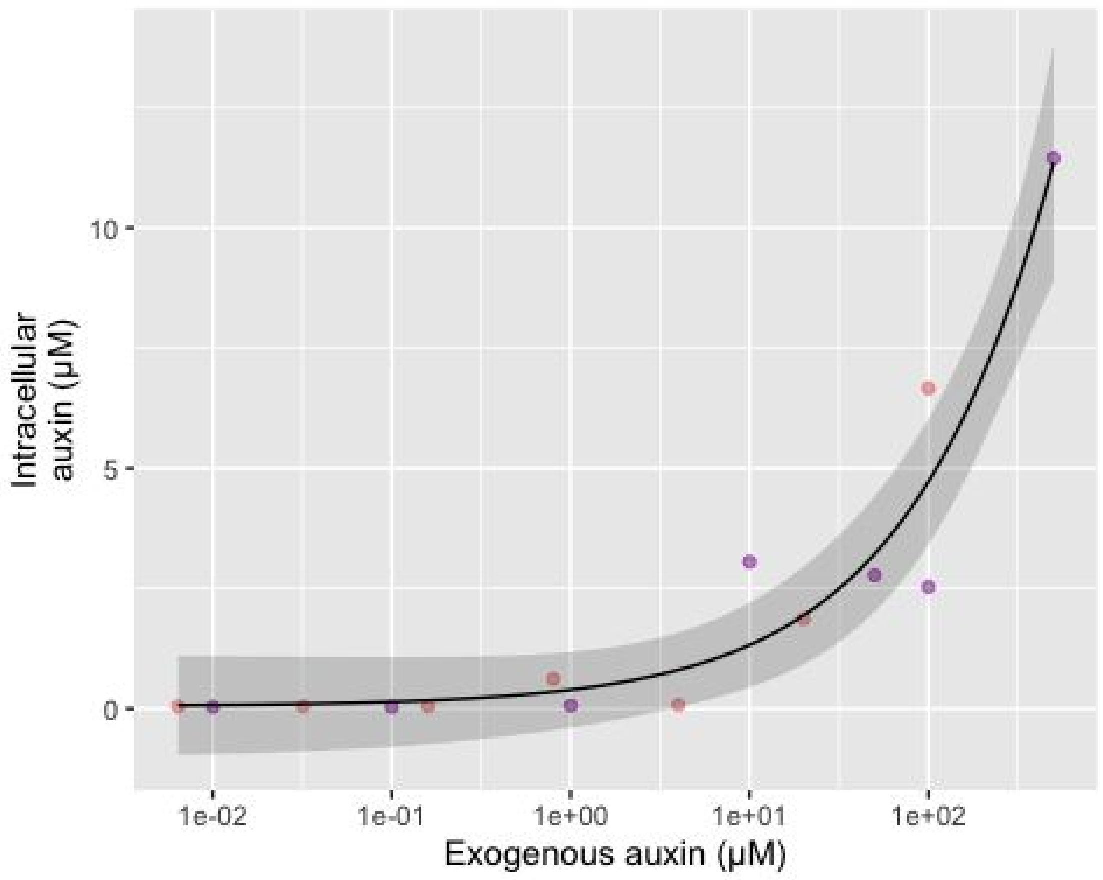

Model sensor response to dose of exogenous auxin

```
ggplot(data = lcms_data, mapping = aes(x = Dose_IAA, y = Red_Green, color =
experiment)) +
    geom_point() + scale_x_log10() + scale_y_log10() +
scale_color_viridis_d(begin = 0.3,
    end = 0.6, option = "plasma")
```

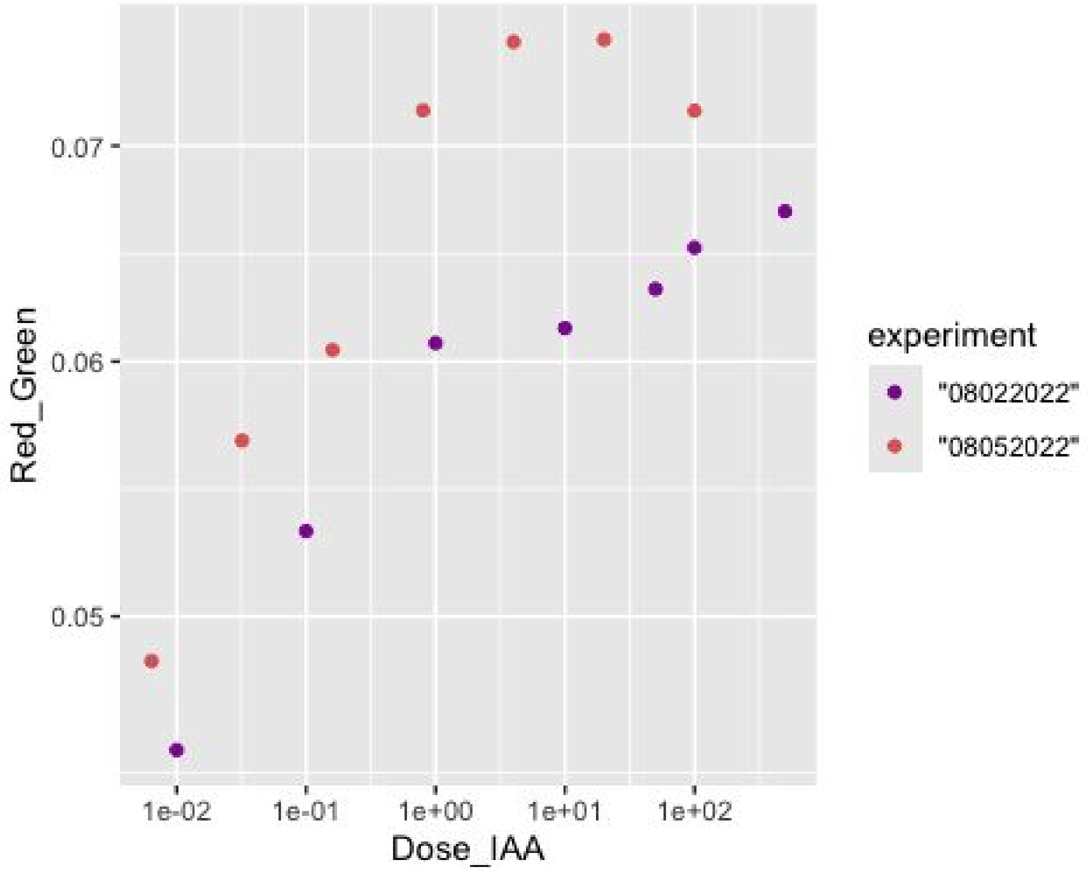

Again we would expect the biosensor to follow a log-logistic sigmoidal model. We will begin with 4-parameter.

```
sensor_dose4 <- drm(Red_Green ~ Dose_IAA, data = lcms_data, fct = LL.4())
summary(sensor_dose4)
# #
# # Model fitted: Log-logistic (ED50 as parameter) (4 parms)
# #
# # Parameter estimates:
# #
# #                  Estimate Std. Error t-value   p-value
# # b:(Intercept) -1.0482881  0.9844180 -1.0649 0.3119662
# # c:(Intercept)  0.0468044  0.0083925  5.5769 0.0002351 ***
# # d:(Intercept)  0.0684222  0.0021741 31.4711 2.466e-11 ***
# # e:(Intercept)  0.1058917  0.1420457  0.7455 0.4731420
# # ---
```

```
## Signif. codes:  0 '***' 0.001 '**' 0.01 '*' 0.05 '.' 0.1 ' ' 1
##
## Residual standard error:
##
##  0.005691664 (10 degrees of freedom)
AIC(sensor_dose4)
## [1] -99.70541
```

Comparing different model forms

```
mselect(sensor_dose4, list(LL2.4(), LL.5(), LL2.5(), LL.3(), lnormal(),
L.4()), linreg = TRUE,
    icfct = AIC)
## Error in optim(startVec, opfct, hessian = TRUE, method = optMethod,
control = list(maxit = maxIt,  :
##   non-finite finite-difference value [3]
##           logLik        IC Lack of fit      Res var
## lnormal 54.90854 -99.81709   0.5664641 3.213764e-05
## LL.4    54.85270 -99.70541   0.5645635 3.239504e-05
## LL2.4   54.83463 -99.66925   0.5639490 3.247880e-05
## LL.5    55.04776 -98.09552   0.5428047 3.500533e-05
## LL2.5   54.78083 -97.56165   0.5339450 3.636598e-05
## Lin     46.32853 -86.65705          NA 9.123359e-05
## Quad    47.17317 -86.34634          NA 8.821448e-05
## Cubic   47.77963 -85.55926          NA 8.898289e-05
## L.4     46.93236 -83.86472   0.3382902 1.004322e-04
## LL.3          NA        NA          NA           NA
```

We see that log-normal model has the lowest AIC, but the limited dose range of this dataset also prevents the bottom of the curve from being well defined. The AIC and residual variance of log-normal and log-logistic models are very similar. Additionally the model form and shape of log-normal and log-logistic models are quite similar with the exception of tail behavior. As the tail of this model is poorly defined, we will use the log-normal model as suggested by AIC.

```
sensor_dose4 <- drm(Red_Green ~ Dose_IAA, data = lcms_data, fct = lnormal())
summary(sensor_dose4)
##
## Model fitted: Log-normal (4 parms)
##
## Parameter estimates:
##
##                Estimate Std. Error t-value   p-value
## b:(Intercept) 0.5423538  0.4291607  1.2638  0.234983
## c:(Intercept) 0.0454775  0.0106813  4.2577  0.001669 **
## d:(Intercept) 0.0684111  0.0021358 32.0302 2.071e-11 ***
## e:(Intercept) 0.0856090  0.1409665  0.6073  0.557189
## ---
## Signif. codes:  0 '***' 0.001 '**' 0.01 '*' 0.05 '.' 0.1 ' ' 1
##
## Residual standard error:
```

```
# #
# #  0.005669007 (10 degrees of freedom)
AIC(sensor_dose4)
# # [1] -99.81709

# predictions and confidence intervals.
sensor_dose4.pred <- expand.grid(conc = exp(seq(log(max(lcms_data$Dose_IAA)),
log(min(lcms_data$Dose_IAA)),
    length = 100)))
# new data with predictions
pm <- predict(sensor_dose4, newdata = sensor_dose4.pred, interval =
"confidence")
sensor_dose4.pred$p <- pm[, 1]
sensor_dose4.pred$pmin <- pm[, 2]
sensor_dose4.pred$pmax <- pm[, 3]


ggplot(data = lcms_data, mapping = aes(x = Dose_IAA, y = Red_Green, color =
experiment)) +
    geom_point(alpha = 0.5) + geom_ribbon(data = sensor_dose4.pred, aes(x =
conc,
    y = p, ymin = pmin, ymax = pmax), alpha = 0.2, inherit.aes = FALSE) +
geom_line(data = sensor_dose4.pred,
    aes(x = conc, y = p), inherit.aes = FALSE) + scale_x_log10() +
guides(color = "none") +
    labs(x = "Exogenous auxin (µM)", y = "mScarlet/Venus") +
scale_color_viridis_d(begin = 0.3,
    end = 0.6, option = "plasma") -> sensor_dose
sensor_dose
```

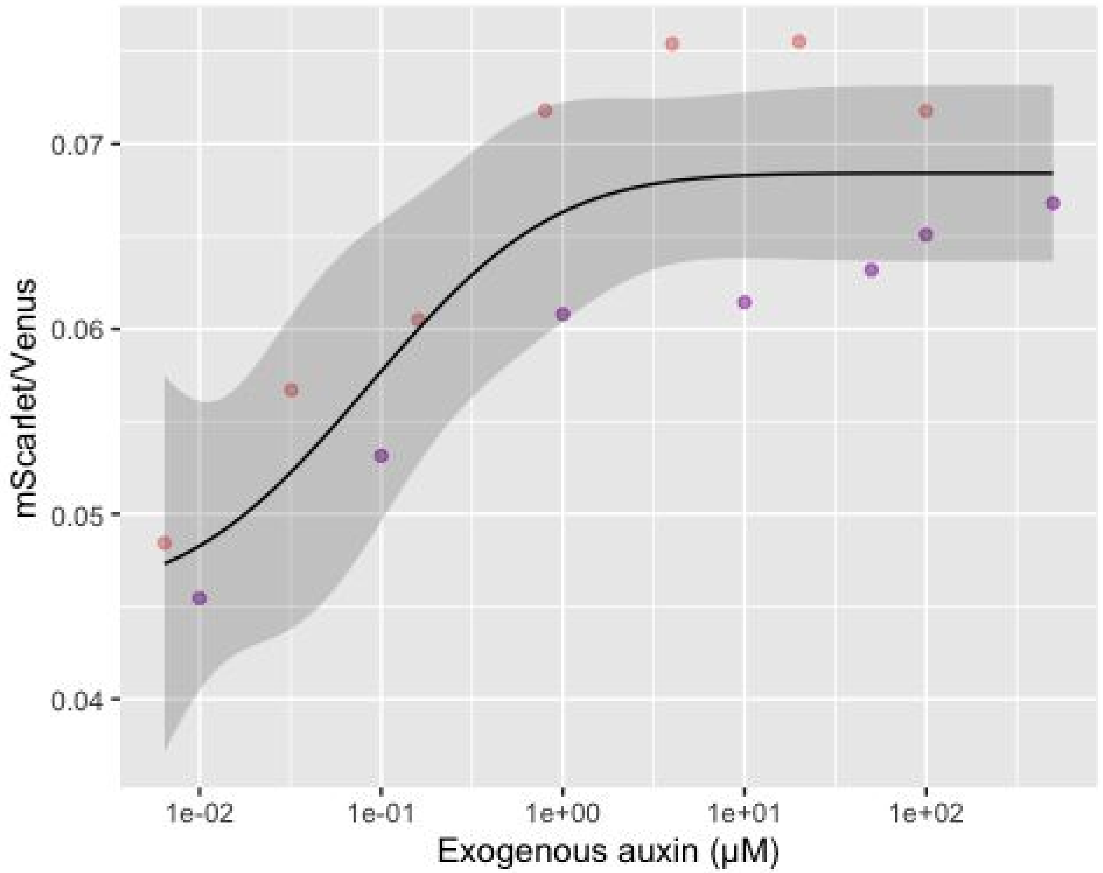

######

Model biosensor response vs measured intracellular auxin

```
ggplot(data = lcms_data, mapping = aes(x = Observe_IAA_in_uM, y = Red_Green,
color = experiment)) +
    geom_point() + scale_x_log10() + scale_y_log10() +
scale_color_viridis_d(begin = 0.3,
    end = 0.6, option = "plasma")
```

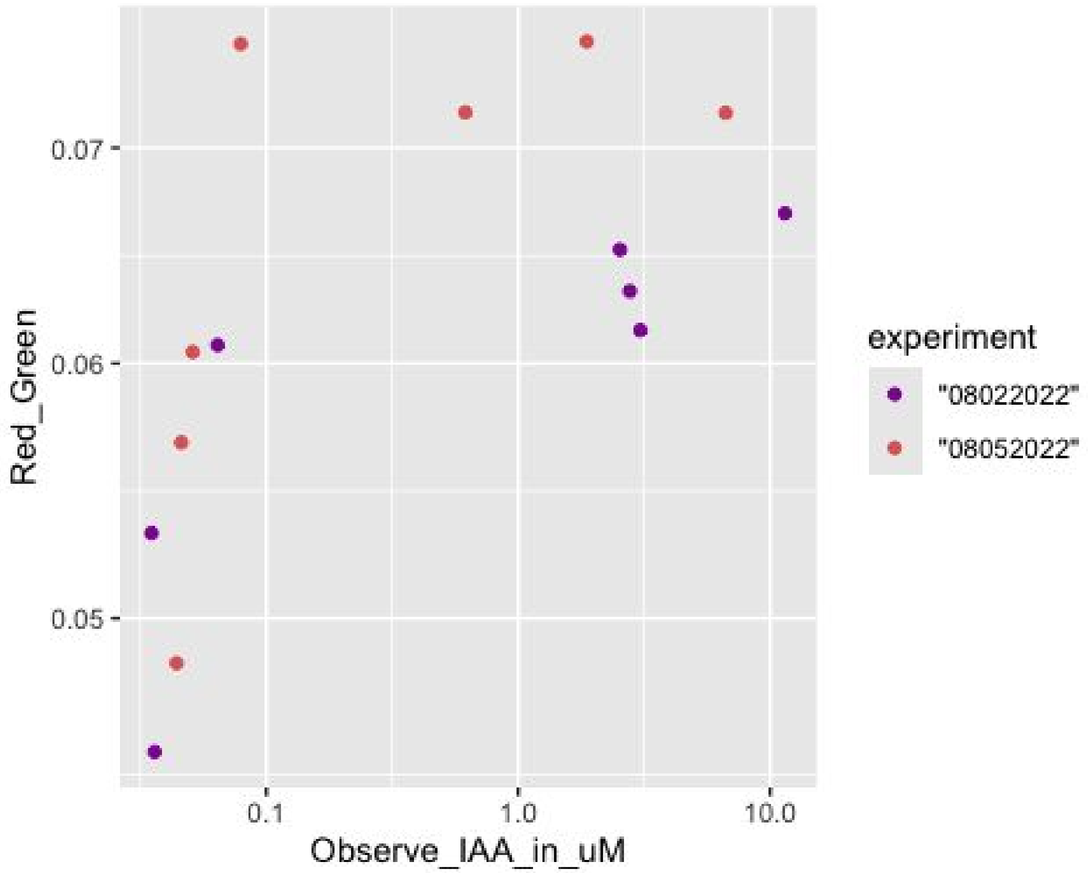

Once again we begin with a log-logistic model as we would expect a sigmoidal curve if observed intracellular auxin could be reduced to zero, perhaps via mutagenesis.

```
sensor_intra <- drm(Red_Green ~ Observe_IAA_in_uM, data = lcms_data, fct =
LL.4())
summary(sensor_intra)
# #
# # Model fitted: Log-logistic (ED50 as parameter) (4 parms)
# #
# # Parameter estimates:
# #
# #                   Estimate Std. Error t-value   p-value
# # b:(Intercept) -8.2406629  6.1221748 -1.3460     0.208
# # c:(Intercept)  0.0479589  0.0052282  9.1731 3.485e-06 ***
# # d:(Intercept)  0.0685034  0.0020055 34.1569 1.095e-11 ***
# # e:(Intercept)  0.0510555  0.0065434  7.8026 1.465e-05 ***
# # ---
# # Signif. codes:  0 '***' 0.001 '**' 0.01 '*' 0.05 '.' 0.1 ' ' 1
# #
# # Residual standard error:
# #
# #  0.005521366 (10 degrees of freedom)
AIC(sensor_intra)
```

```
# # [1] -100.556
plot(sensor_intra)
```

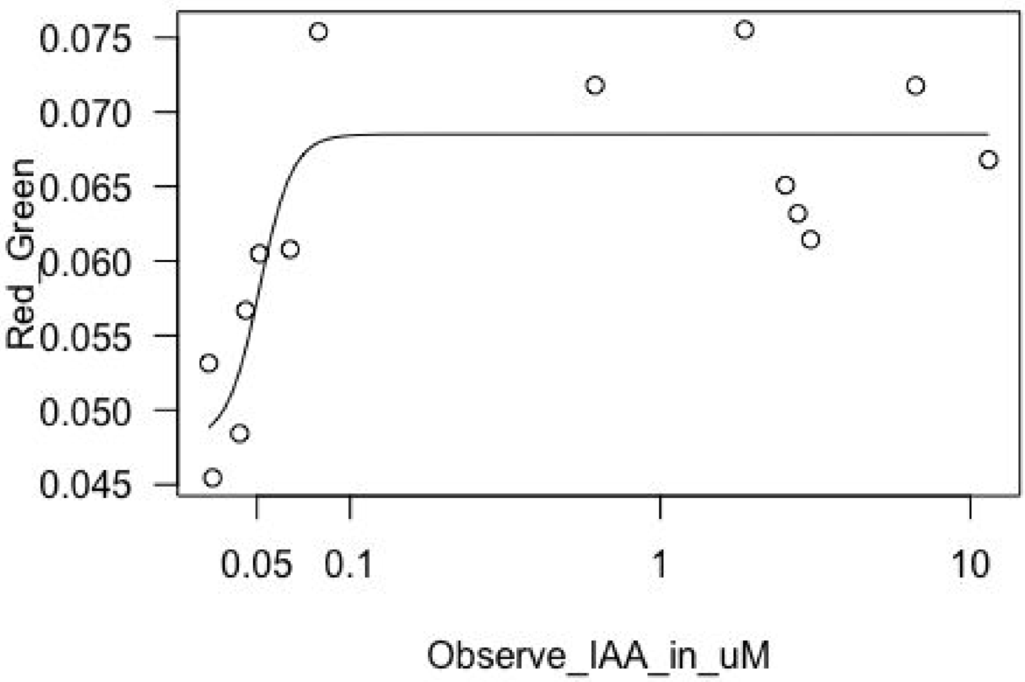


```
mselect(sensor_intra, list(LL2.4(), LL.5(), LL2.5(), LL.3(), lnormal(),
L.4()), linreg = TRUE,
    icfct = AIC)
# #            logLik         IC Lack of fit      Res var
# # LL.4     55.27799 -100.55597          NA 3.048548e-05
# # LL.3     54.09468 -100.18935          NA 3.281825e-05
# # LL.5     55.37599  -98.75197          NA 3.340184e-05
# # lnormal  54.02716  -98.05432          NA 3.644995e-05
# # LL2.4    53.89330  -97.78660          NA 3.715368e-05
# # LL2.5    53.81308  -95.62616          NA 4.175768e-05
# # Lin      46.91282  -87.82564          NA 8.392748e-05
# # Quad     47.78085  -87.56170          NA 8.087944e-05
# # L.4      48.20921  -86.41843          NA 8.368628e-05
# # Cubic    47.88493  -85.76985          NA 8.765442e-05
```

In this case the 4-parameter log-logistic model had the lowest AIC and is still the best model for this dataset.

```
sensor_intra <- drm(Red_Green ~ Observe_IAA_in_uM, data = lcms_data, fct =
LL.4())
```

```
summary(sensor_intra)
# #
# # Model fitted: Log-logistic (ED50 as parameter) (4 parms)
# #
# # Parameter estimates:
# #
# #                   Estimate Std. Error t-value   p-value
# # b:(Intercept) -8.2406629  6.1221748 -1.3460     0.208
# # c:(Intercept)  0.0479589  0.0052282  9.1731 3.485e-06 ***
# # d:(Intercept)  0.0685034  0.0020055 34.1569 1.095e-11 ***
# # e:(Intercept)  0.0510555  0.0065434  7.8026 1.465e-05 ***
# # ---
# # Signif. codes:  0 '***' 0.001 '**' 0.01 '*' 0.05 '.' 0.1 ' ' 1
# #
# # Residual standard error:
# #
# #  0.005521366 (10 degrees of freedom)
AIC(sensor_intra)
# # [1] -100.556

# predictions and confidence intervals.
sensor_intra.pred <- expand.grid(conc =
exp(seq(log(max(lcms_data$Observe_IAA_in_uM)),
    log(min(lcms_data$Observe_IAA_in_uM)), length = 100)))
# new data with predictions
pm <- predict(sensor_intra, newdata = sensor_intra.pred, interval =
"confidence")
sensor_intra.pred$p <- pm[, 1]
sensor_intra.pred$pmin <- pm[, 2]
sensor_intra.pred$pmax <- pm[, 3]


ggplot(data = lcms_data, mapping = aes(x = Observe_IAA_in_uM, y = Red_Green,
color = experiment)) +
    geom_point(alpha = 0.5) + geom_ribbon(data = sensor_intra.pred, aes(x =
conc,
    y = p, ymin = pmin, ymax = pmax), alpha = 0.2, inherit.aes = FALSE) +
geom_line(data = sensor_intra.pred,
    aes(x = conc, y = p), inherit.aes = FALSE) + scale_x_log10() +
guides(color = "none") +
    labs(x = "Intracellular auxin (μM)", y = "mScarlet/Venus") +
scale_color_viridis_d(begin = 0.3,
    end = 0.6, option = "plasma") -> sensor_intra
sensor_intra
```

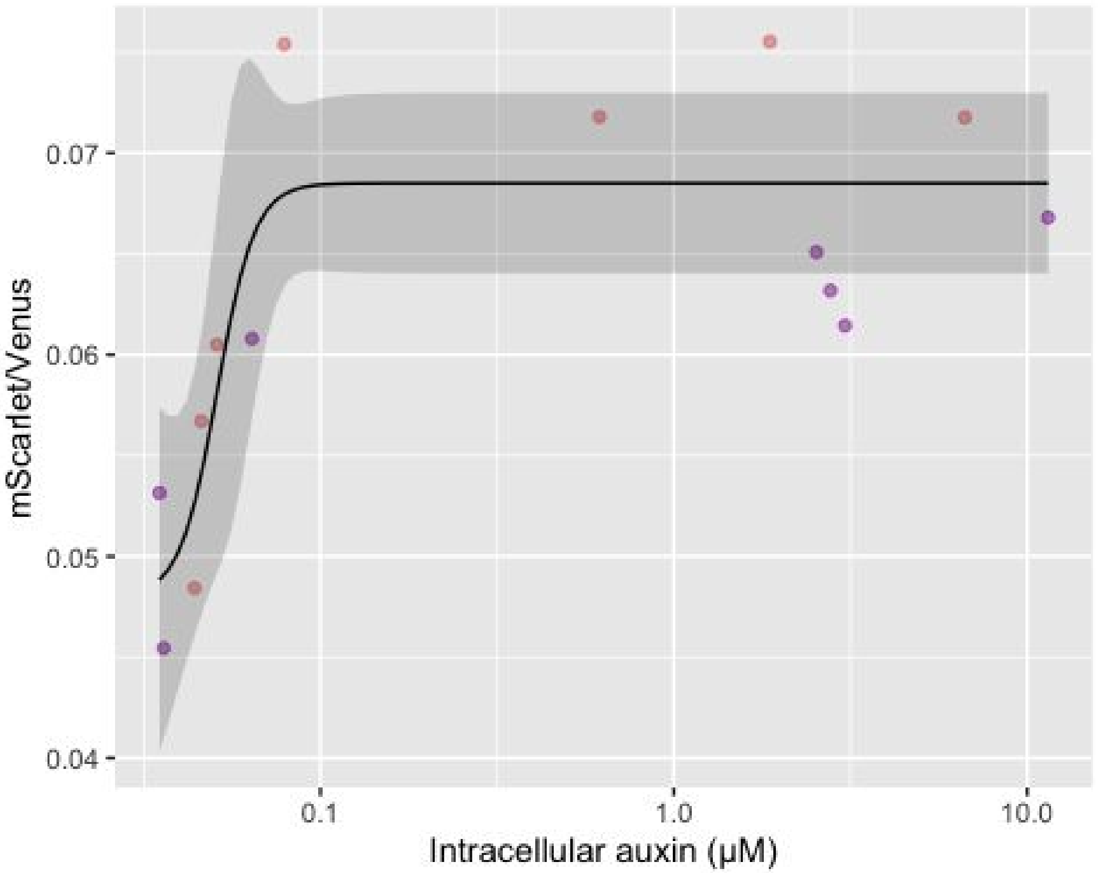


```
library(patchwork)
combined_plot <- intra_dose/sensor_dose/sensor_intra +
plot_annotation(tag_levels = ("A")) &
    theme(text = element_text(size = 8))
combined_plot
```

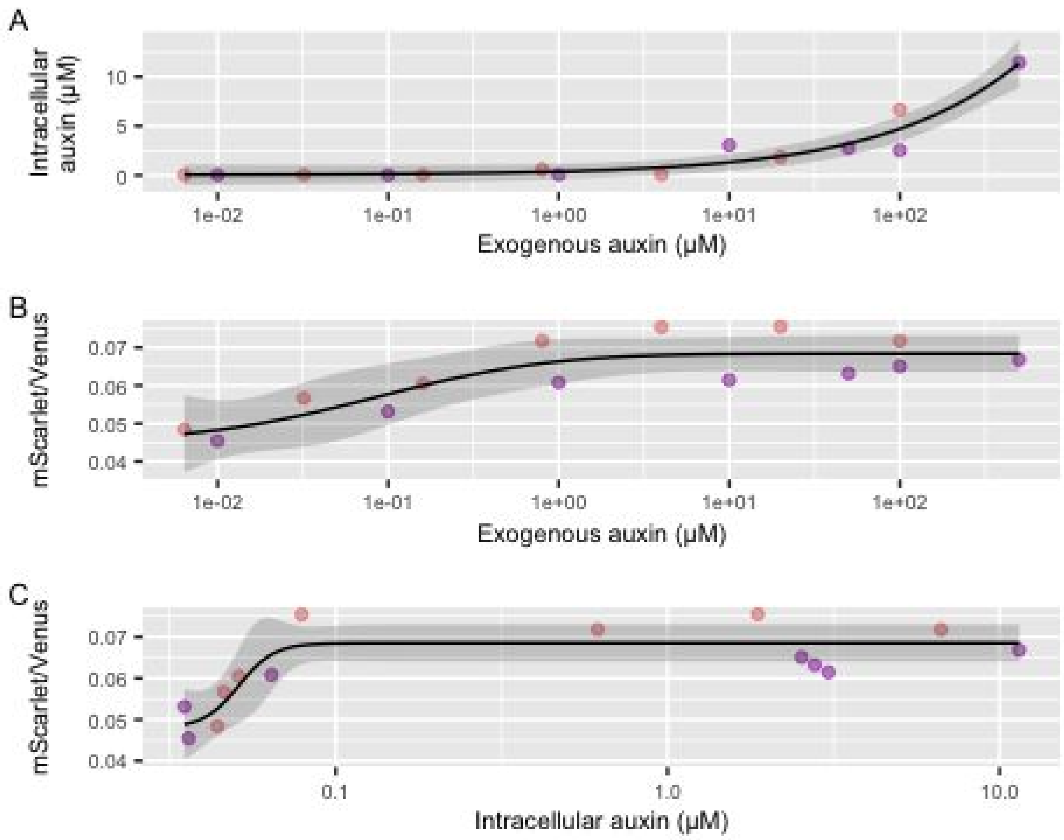


```
# ggsave('combined_plot.png', width = 3.5, height = 5)
```

Figure 8A: Dose-response curve for auxin at early stationary phase, 2 replicates

```
plate_DRA_stat_combined <- read.plateSet(path = "Data for publication/Dose-
response_Stationary_combined/All data combined/",
    pattern = "S-DRA*")

# original cultures, very concentrated, dilute the cultures 'before each
# measurement' with 100 ul SCM, and 10 ul cell culture

annotation_plate_DRA_stat_combined <- createAnnotation(yourFlowSet =
plate_DRA_stat_combined)
write.csv(annotation_plate_DRA_stat_combined, "/Users/patchaisupa/OneDrive -
Virginia Tech/Manuscripts/auxin biosensor/Data for publication/Dose-
response_Stationary_combined/plate_DRA_stat_annotation.csv")


annotation_plate_DRA_stat_combined <- read.csv("Data for publication/Dose-
response_Stationary_combined/plate_DRA_stat_annotation.csv")

aplate_DRA_stat_combined <- annotateFlowSet(yourFlowSet =
plate_DRA_stat_combined,
```

```
    annotation_df = annotation_plate_DRA_stat_combined, mergeBy = "name")
head(rownames(pData(aplate_DRA_stat_combined)))
# # [1] "1Pat-04212024-yWL210_Stationary_DRA06-B_Experiment_Colony 1_IAA 0
uM.fcs"
# # [2] "1Pat-04212024-yWL210_Stationary_DRA06-B_Experiment_Colony 1_IAA
0.000095 uM.fcs"
# # [3] "1Pat-04212024-yWL210_Stationary_DRA06-B_Experiment_Colony 1_IAA
0.00038 uM.fcs"
# # [4] "1Pat-04212024-yWL210_Stationary_DRA06-B_Experiment_Colony 1_IAA
0.0015 uM.fcs"
# # [5] "1Pat-04212024-yWL210_Stationary_DRA06-B_Experiment_Colony 1_IAA
0.0061 uM.fcs"
# # [6] "1Pat-04212024-yWL210_Stationary_DRA06-B_Experiment_Colony 1_IAA
0.0244 uM.fcs"
head(pData(aplate_DRA_stat_combined))
# #
name
# # 1Pat-04212024-yWL210_Stationary_DRA06-B_Experiment_Colony 1_IAA 0 uM.fcs
1Pat-04212024-yWL210_Stationary_DRA06-B_Experiment_Colony 1_IAA 0 uM.fcs
# # 1Pat-04212024-yWL210_Stationary_DRA06-B_Experiment_Colony 1_IAA 0.000095
uM.fcs 1Pat-04212024-yWL210_Stationary_DRA06-B_Experiment_Colony 1_IAA
0.000095 uM.fcs
# # 1Pat-04212024-yWL210_Stationary_DRA06-B_Experiment_Colony 1_IAA 0.00038
uM.fcs   1Pat-04212024-yWL210_Stationary_DRA06-B_Experiment_Colony 1_IAA
0.00038 uM.fcs
# # 1Pat-04212024-yWL210_Stationary_DRA06-B_Experiment_Colony 1_IAA 0.0015
uM.fcs     1Pat-04212024-yWL210_Stationary_DRA06-B_Experiment_Colony 1_IAA
0.0015 uM.fcs
# # 1Pat-04212024-yWL210_Stationary_DRA06-B_Experiment_Colony 1_IAA 0.0061
uM.fcs     1Pat-04212024-yWL210_Stationary_DRA06-B_Experiment_Colony 1_IAA
0.0061 uM.fcs
# # 1Pat-04212024-yWL210_Stationary_DRA06-B_Experiment_Colony 1_IAA 0.0244
uM.fcs     1Pat-04212024-yWL210_Stationary_DRA06-B_Experiment_Colony 1_IAA
0.0244 uM.fcs
# #
folder
# # 1Pat-04212024-yWL210_Stationary_DRA06-B_Experiment_Colony 1_IAA 0 uM.fcs
S-DRA06
# # 1Pat-04212024-yWL210_Stationary_DRA06-B_Experiment_Colony 1_IAA 0.000095
uM.fcs S-DRA06
# # 1Pat-04212024-yWL210_Stationary_DRA06-B_Experiment_Colony 1_IAA 0.00038
uM.fcs  S-DRA06
# # 1Pat-04212024-yWL210_Stationary_DRA06-B_Experiment_Colony 1_IAA 0.0015
uM.fcs   S-DRA06
# # 1Pat-04212024-yWL210_Stationary_DRA06-B_Experiment_Colony 1_IAA 0.0061
uM.fcs   S-DRA06
# # 1Pat-04212024-yWL210_Stationary_DRA06-B_Experiment_Colony 1_IAA 0.0244
uM.fcs   S-DRA06
# #
X
```

```
# # 1Pat-04212024-yWL210_Stationary_DRA06-B_Experiment_Colony 1_IAA 0 uM.fcs
1
# # 1Pat-04212024-yWL210_Stationary_DRA06-B_Experiment_Colony 1_IAA 0.000095
uM.fcs 2
# # 1Pat-04212024-yWL210_Stationary_DRA06-B_Experiment_Colony 1_IAA 0.00038
uM.fcs  3
# # 1Pat-04212024-yWL210_Stationary_DRA06-B_Experiment_Colony 1_IAA 0.0015
uM.fcs   4
# # 1Pat-04212024-yWL210_Stationary_DRA06-B_Experiment_Colony 1_IAA 0.0061
uM.fcs   5
# # 1Pat-04212024-yWL210_Stationary_DRA06-B_Experiment_Colony 1_IAA 0.0244
uM.fcs   6
# #
replicate
# # 1Pat-04212024-yWL210_Stationary_DRA06-B_Experiment_Colony 1_IAA 0 uM.fcs
1
# # 1Pat-04212024-yWL210_Stationary_DRA06-B_Experiment_Colony 1_IAA 0.000095
uM.fcs         1
# # 1Pat-04212024-yWL210_Stationary_DRA06-B_Experiment_Colony 1_IAA 0.00038
uM.fcs          1
# # 1Pat-04212024-yWL210_Stationary_DRA06-B_Experiment_Colony 1_IAA 0.0015
uM.fcs           1
# # 1Pat-04212024-yWL210_Stationary_DRA06-B_Experiment_Colony 1_IAA 0.0061
uM.fcs           1
# # 1Pat-04212024-yWL210_Stationary_DRA06-B_Experiment_Colony 1_IAA 0.0244
uM.fcs           1
# #
dose
# # 1Pat-04212024-yWL210_Stationary_DRA06-B_Experiment_Colony 1_IAA 0 uM.fcs
0.0000000
# # 1Pat-04212024-yWL210_Stationary_DRA06-B_Experiment_Colony 1_IAA 0.000095
uM.fcs 0.0000953
# # 1Pat-04212024-yWL210_Stationary_DRA06-B_Experiment_Colony 1_IAA 0.00038
uM.fcs  0.0003810
# # 1Pat-04212024-yWL210_Stationary_DRA06-B_Experiment_Colony 1_IAA 0.0015
uM.fcs   0.0015250
# # 1Pat-04212024-yWL210_Stationary_DRA06-B_Experiment_Colony 1_IAA 0.0061
uM.fcs   0.0061000
# # 1Pat-04212024-yWL210_Stationary_DRA06-B_Experiment_Colony 1_IAA 0.0244
uM.fcs   0.0244100
# #
treatment
# # 1Pat-04212024-yWL210_Stationary_DRA06-B_Experiment_Colony 1_IAA 0 uM.fcs
0 uM
# # 1Pat-04212024-yWL210_Stationary_DRA06-B_Experiment_Colony 1_IAA 0.000095
uM.fcs 0.0000953 uM
# # 1Pat-04212024-yWL210_Stationary_DRA06-B_Experiment_Colony 1_IAA 0.00038
uM.fcs   0.000381 uM
# # 1Pat-04212024-yWL210_Stationary_DRA06-B_Experiment_Colony 1_IAA 0.0015
uM.fcs    0.001525 uM
```

```
# # 1Pat-04212024-yWL210_Stationary_DRA06-B_Experiment_Colony 1_IAA 0.0061
uM.fcs      0.0061 uM
# # 1Pat-04212024-yWL210_Stationary_DRA06-B_Experiment_Colony 1_IAA 0.0244
uM.fcs     0.02441 uM

plate_DRA_stat_combined_sum <- summarizeFlow(aplate_DRA_stat_combined,
channel = c("BL1.A",
    "YL1.A"), gated = TRUE)
# # [1] "Summarizing all events..."

### Dose-response curve Comparing log-logistic and Weibull models (Figure 2
in
### Ritz (2009))

fitdrc.m1 <- drm(YL1.Amean/BL1.Amean ~ dose, data =
plate_DRA_stat_combined_sum,
    fct = LL.4())



model.LL4_all_stat <- drm(YL1.Amean/BL1.Amean ~ dose, data =
plate_DRA_stat_combined_sum,
    fct = LL.4(names = c("Slope", "Lower Limit", "Upper Limit", "ED50")))
plot(model.LL4_all_stat, broken = TRUE, type = "none", lty = 1, lwd = 5, xlab
= "Extracellular IAA concentration (uM)",
    ylab = "Response Signal")
plot(model.LL4_all_stat, broken = TRUE, type = "confidence", col = "black",
add = TRUE)
summary(model.LL4_all_stat)
# #
# # Model fitted: Log-logistic (ED50 as parameter) (4 parms)
# #
# # Parameter estimates:
# #
# #                              Estimate Std. Error t-value p-value
# # Slope:(Intercept)          -0.4817911  0.1550413 -3.1075 0.00555 **
# # Lower Limit:(Intercept)     0.2380062  0.0081121 29.3398 < 2e-16 ***
# # Upper Limit:(Intercept)     0.2941612  0.0039880 73.7613 < 2e-16 ***
# # ED50:(Intercept)            0.0030387  0.0033813  0.8987 0.37951
# # ---
# # Signif. codes:  0 '***' 0.001 '**' 0.01 '*' 0.05 '.' 0.1 ' ' 1
# #
# # Residual standard error:
# #
# #  0.01050806 (20 degrees of freedom)


replicate1_stat <- drm(YL1.Amean/BL1.Amean ~ dose, data =
subset(plate_DRA_stat_combined_sum,
    replicate == "1"), fct = LL.4())
```

```
replicate2_stat <- drm(YL1.Amean/BL1.Amean ~ dose, data =
subset(plate_DRA_stat_combined_sum,
    replicate == "2"), fct = LL.4())


plot(replicate1_stat, broken = TRUE, add = TRUE, type = "all", col = "dark
green",
    lty = 2)
plot(replicate2_stat, broken = TRUE, add = TRUE, type = "all", col = "dark
blue",
    lty = 2)
```

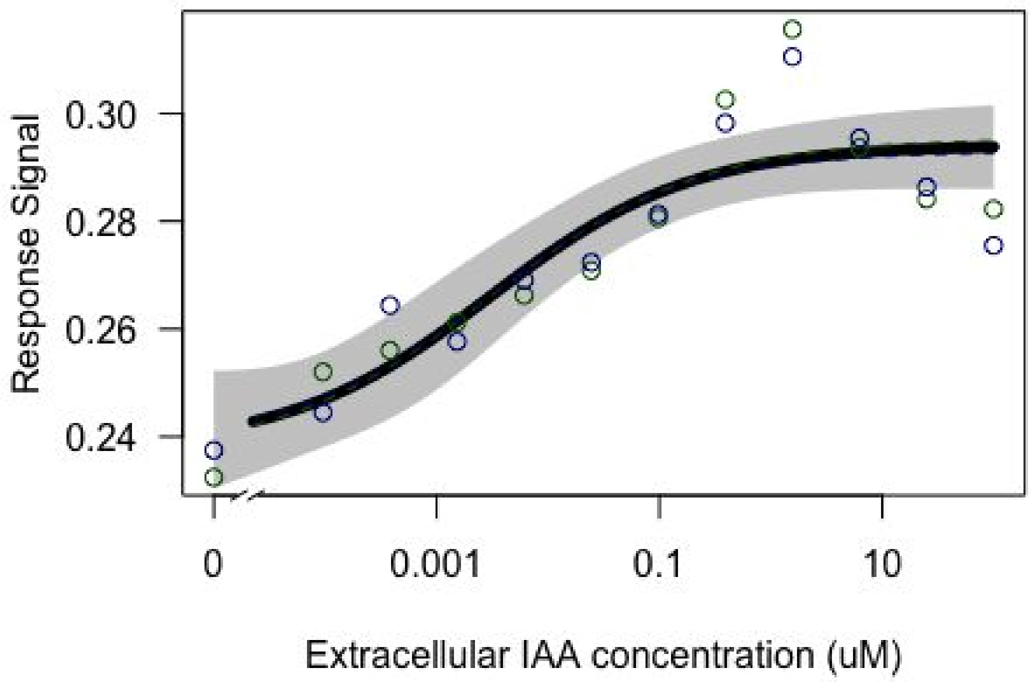


```
# # Scale for x is already present.
# # Adding another scale for x, which will replace the existing scale.
# # Scale for y is already present.
# # Adding another scale for y, which will replace the existing scale.
```

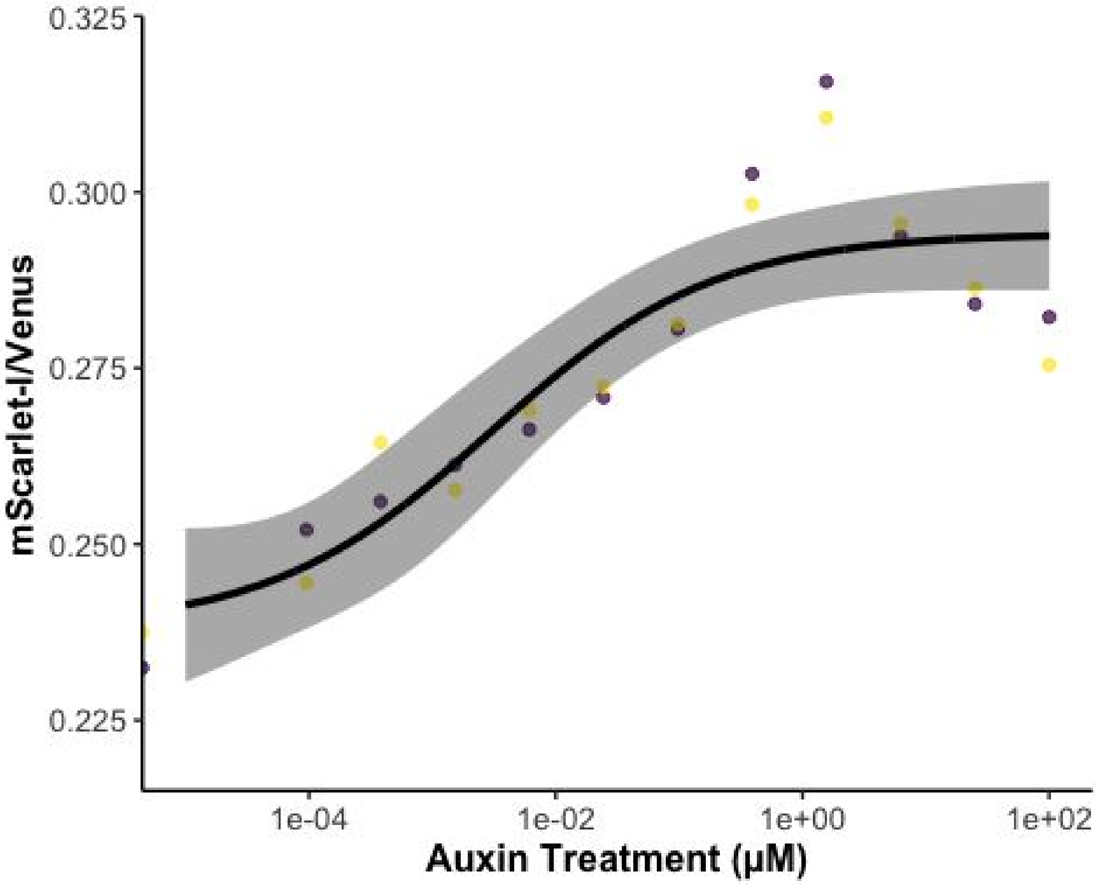

Figure 8B: Auxin accumulation in different yeast cultures measured by the AFB2 dual-fusion biosensor

```
plate_05052023_IAAacc <- read.plateSet(path = "Data for
publication/05052023_yWL209-210_IAAaccumulation/All data/",
    pattern = "Measurement*")

annotation <- createAnnotation(yourFlowSet = plate_05052023_IAAacc)
write.csv(annotation, "Data for publication/05052023_yWL209-
210_IAAaccumulation/05052023_IAAacc_annotation.csv")

annotation <- read.csv("Data for publication/05052023_yWL209-
210_IAAaccumulation/05052023_IAAacc_annotation.csv")
aplate_05052023_IAAacc <- annotateFlowSet(yourFlowSet =
plate_05052023_IAAacc, annotation_df = annotation,
    mergeBy = "name")
head(rownames(pData(aplate_05052023_IAAacc)))
# # [1] "1Pat-05052023-IAAaccumulations_Expo1_IAAaccumulation_Aerobic_yWL209-
Control.fcs"
# # [2] "1Pat-05052023-IAAaccumulations_Expo1_IAAaccumulation_Aerobic_yWL209-
IAAspike.fcs"
# # [3] "1Pat-05052023-IAAaccumulations_Expo1_IAAaccumulation_Aerobic_yWL210-
Control.fcs"
# # [4] "1Pat-05052023-IAAaccumulations_Expo1_IAAaccumulation_Aerobic_yWL210-
```

```
IAAspike.fcs"
# # [5] "1Pat-05052023-
IAAaccumulations_Expo1_IAAaccumulation_Anaerobic_yWL209-Control.fcs"
# # [6] "1Pat-05052023-
IAAaccumulations_Expo1_IAAaccumulation_Anaerobic_yWL209-IAAspike.fcs"
head(pData(aplate_05052023_IAAacc))
# #
name
# # 1Pat-05052023-IAAaccumulations_Expo1_IAAaccumulation_Aerobic_yWL209-
Control.fcs       1Pat-05052023-
IAAaccumulations_Expo1_IAAaccumulation_Aerobic_yWL209-Control.fcs
# # 1Pat-05052023-IAAaccumulations_Expo1_IAAaccumulation_Aerobic_yWL209-
IAAspike.fcs     1Pat-05052023-
IAAaccumulations_Expo1_IAAaccumulation_Aerobic_yWL209-IAAspike.fcs
# # 1Pat-05052023-IAAaccumulations_Expo1_IAAaccumulation_Aerobic_yWL210-
Control.fcs       1Pat-05052023-
IAAaccumulations_Expo1_IAAaccumulation_Aerobic_yWL210-Control.fcs
# # 1Pat-05052023-IAAaccumulations_Expo1_IAAaccumulation_Aerobic_yWL210-
IAAspike.fcs     1Pat-05052023-
IAAaccumulations_Expo1_IAAaccumulation_Aerobic_yWL210-IAAspike.fcs
# # 1Pat-05052023-IAAaccumulations_Expo1_IAAaccumulation_Anaerobic_yWL209-
Control.fcs   1Pat-05052023-
IAAaccumulations_Expo1_IAAaccumulation_Anaerobic_yWL209-Control.fcs
# # 1Pat-05052023-IAAaccumulations_Expo1_IAAaccumulation_Anaerobic_yWL209-
IAAspike.fcs 1Pat-05052023-
IAAaccumulations_Expo1_IAAaccumulation_Anaerobic_yWL209-IAAspike.fcs
# #
folder
# # 1Pat-05052023-IAAaccumulations_Expo1_IAAaccumulation_Aerobic_yWL209-
Control.fcs    Measurement01
# # 1Pat-05052023-IAAaccumulations_Expo1_IAAaccumulation_Aerobic_yWL209-
IAAspike.fcs   Measurement01
# # 1Pat-05052023-IAAaccumulations_Expo1_IAAaccumulation_Aerobic_yWL210-
Control.fcs    Measurement01
# # 1Pat-05052023-IAAaccumulations_Expo1_IAAaccumulation_Aerobic_yWL210-
IAAspike.fcs   Measurement01
# # 1Pat-05052023-IAAaccumulations_Expo1_IAAaccumulation_Anaerobic_yWL209-
Control.fcs  Measurement01
# # 1Pat-05052023-IAAaccumulations_Expo1_IAAaccumulation_Anaerobic_yWL209-
IAAspike.fcs Measurement01
# #
X
# # 1Pat-05052023-IAAaccumulations_Expo1_IAAaccumulation_Aerobic_yWL209-
Control.fcs    1
# # 1Pat-05052023-IAAaccumulations_Expo1_IAAaccumulation_Aerobic_yWL209-
IAAspike.fcs   2
# # 1Pat-05052023-IAAaccumulations_Expo1_IAAaccumulation_Aerobic_yWL210-
Control.fcs    3
# # 1Pat-05052023-IAAaccumulations_Expo1_IAAaccumulation_Aerobic_yWL210-
IAAspike.fcs   4
```

```
# # 1Pat-05052023-IAAaccumulations_Expo1_IAAaccumulation_Anaerobic_yWL209-
Control.fcs  5
# # 1Pat-05052023-IAAaccumulations_Expo1_IAAaccumulation_Anaerobic_yWL209-
IAAspike.fcs 6
# #
condition
# # 1Pat-05052023-IAAaccumulations_Expo1_IAAaccumulation_Aerobic_yWL209-
Control.fcs      Aerobic
# # 1Pat-05052023-IAAaccumulations_Expo1_IAAaccumulation_Aerobic_yWL209-
IAAspike.fcs     Aerobic
# # 1Pat-05052023-IAAaccumulations_Expo1_IAAaccumulation_Aerobic_yWL210-
Control.fcs      Aerobic
# # 1Pat-05052023-IAAaccumulations_Expo1_IAAaccumulation_Aerobic_yWL210-
IAAspike.fcs     Aerobic
# # 1Pat-05052023-IAAaccumulations_Expo1_IAAaccumulation_Anaerobic_yWL209-
Control.fcs  Anaerobic
# # 1Pat-05052023-IAAaccumulations_Expo1_IAAaccumulation_Anaerobic_yWL209-
IAAspike.fcs Anaerobic
# #
treatment
# # 1Pat-05052023-IAAaccumulations_Expo1_IAAaccumulation_Aerobic_yWL209-
Control.fcs      Control
# # 1Pat-05052023-IAAaccumulations_Expo1_IAAaccumulation_Aerobic_yWL209-
IAAspike.fcs    1 uM IAA
# # 1Pat-05052023-IAAaccumulations_Expo1_IAAaccumulation_Aerobic_yWL210-
Control.fcs      Control
# # 1Pat-05052023-IAAaccumulations_Expo1_IAAaccumulation_Aerobic_yWL210-
IAAspike.fcs    1 uM IAA
# # 1Pat-05052023-IAAaccumulations_Expo1_IAAaccumulation_Anaerobic_yWL209-
Control.fcs    Control
# # 1Pat-05052023-IAAaccumulations_Expo1_IAAaccumulation_Anaerobic_yWL209-
IAAspike.fcs  1 uM IAA
# #
reading
# # 1Pat-05052023-IAAaccumulations_Expo1_IAAaccumulation_Aerobic_yWL209-
Control.fcs          1
# # 1Pat-05052023-IAAaccumulations_Expo1_IAAaccumulation_Aerobic_yWL209-
IAAspike.fcs         1
# # 1Pat-05052023-IAAaccumulations_Expo1_IAAaccumulation_Aerobic_yWL210-
Control.fcs          1
# # 1Pat-05052023-IAAaccumulations_Expo1_IAAaccumulation_Aerobic_yWL210-
IAAspike.fcs         1
# # 1Pat-05052023-IAAaccumulations_Expo1_IAAaccumulation_Anaerobic_yWL209-
Control.fcs        1
# # 1Pat-05052023-IAAaccumulations_Expo1_IAAaccumulation_Anaerobic_yWL209-
IAAspike.fcs       1
# #
strain
# # 1Pat-05052023-IAAaccumulations_Expo1_IAAaccumulation_Aerobic_yWL209-
Control.fcs    yWL209
```

```
# # 1Pat-05052023-IAAaccumulations_Expo1_IAAaccumulation_Aerobic_yWL209-
IAAspike.fcs   yWL209
# # 1Pat-05052023-IAAaccumulations_Expo1_IAAaccumulation_Aerobic_yWL210-
Control.fcs    yWL210
# # 1Pat-05052023-IAAaccumulations_Expo1_IAAaccumulation_Aerobic_yWL210-
IAAspike.fcs   yWL210
# # 1Pat-05052023-IAAaccumulations_Expo1_IAAaccumulation_Anaerobic_yWL209-
Control.fcs  yWL209
# # 1Pat-05052023-IAAaccumulations_Expo1_IAAaccumulation_Anaerobic_yWL209-
IAAspike.fcs yWL209
# #
phase
# # 1Pat-05052023-IAAaccumulations_Expo1_IAAaccumulation_Aerobic_yWL209-
Control.fcs    exponential
# # 1Pat-05052023-IAAaccumulations_Expo1_IAAaccumulation_Aerobic_yWL209-
IAAspike.fcs   exponential
# # 1Pat-05052023-IAAaccumulations_Expo1_IAAaccumulation_Aerobic_yWL210-
Control.fcs    exponential
# # 1Pat-05052023-IAAaccumulations_Expo1_IAAaccumulation_Aerobic_yWL210-
IAAspike.fcs   exponential
# # 1Pat-05052023-IAAaccumulations_Expo1_IAAaccumulation_Anaerobic_yWL209-
Control.fcs  exponential
# # 1Pat-05052023-IAAaccumulations_Expo1_IAAaccumulation_Anaerobic_yWL209-
IAAspike.fcs exponential
# #
before_after
# # 1Pat-05052023-IAAaccumulations_Expo1_IAAaccumulation_Aerobic_yWL209-
Control.fcs          before
# # 1Pat-05052023-IAAaccumulations_Expo1_IAAaccumulation_Aerobic_yWL209-
IAAspike.fcs         before
# # 1Pat-05052023-IAAaccumulations_Expo1_IAAaccumulation_Aerobic_yWL210-
Control.fcs          before
# # 1Pat-05052023-IAAaccumulations_Expo1_IAAaccumulation_Aerobic_yWL210-
IAAspike.fcs         before
# # 1Pat-05052023-IAAaccumulations_Expo1_IAAaccumulation_Anaerobic_yWL209-
Control.fcs        before
# # 1Pat-05052023-IAAaccumulations_Expo1_IAAaccumulation_Anaerobic_yWL209-
IAAspike.fcs       before
# #
condition_phase
# # 1Pat-05052023-IAAaccumulations_Expo1_IAAaccumulation_Aerobic_yWL209-
Control.fcs      Aerobic exponential
# # 1Pat-05052023-IAAaccumulations_Expo1_IAAaccumulation_Aerobic_yWL209-
IAAspike.fcs     Aerobic exponential
# # 1Pat-05052023-IAAaccumulations_Expo1_IAAaccumulation_Aerobic_yWL210-
Control.fcs      Aerobic exponential
# # 1Pat-05052023-IAAaccumulations_Expo1_IAAaccumulation_Aerobic_yWL210-
IAAspike.fcs     Aerobic exponential
# # 1Pat-05052023-IAAaccumulations_Expo1_IAAaccumulation_Anaerobic_yWL209-
Control.fcs  Anaerobic exponential
```

```
# # 1Pat-05052023-IAAaccumulations_Expo1_IAAaccumulation_Anaerobic_yWL209-
IAAspike.fcs Anaerobic exponential
# #
X.1
# # 1Pat-05052023-IAAaccumulations_Expo1_IAAaccumulation_Aerobic_yWL209-
Control.fcs     NA
# # 1Pat-05052023-IAAaccumulations_Expo1_IAAaccumulation_Aerobic_yWL209-
IAAspike.fcs    NA
# # 1Pat-05052023-IAAaccumulations_Expo1_IAAaccumulation_Aerobic_yWL210-
Control.fcs     NA
# # 1Pat-05052023-IAAaccumulations_Expo1_IAAaccumulation_Aerobic_yWL210-
IAAspike.fcs    NA
# # 1Pat-05052023-IAAaccumulations_Expo1_IAAaccumulation_Anaerobic_yWL209-
Control.fcs   NA
# # 1Pat-05052023-IAAaccumulations_Expo1_IAAaccumulation_Anaerobic_yWL209-
IAAspike.fcs  NA
# #
design_yeast
# # 1Pat-05052023-IAAaccumulations_Expo1_IAAaccumulation_Aerobic_yWL209-
Control.fcs    TIR1 dual-fusion (YPH499)
# # 1Pat-05052023-IAAaccumulations_Expo1_IAAaccumulation_Aerobic_yWL209-
IAAspike.fcs   TIR1 dual-fusion (YPH499)
# # 1Pat-05052023-IAAaccumulations_Expo1_IAAaccumulation_Aerobic_yWL210-
Control.fcs    AFB2 dual-fusion (YPH499)
# # 1Pat-05052023-IAAaccumulations_Expo1_IAAaccumulation_Aerobic_yWL210-
IAAspike.fcs   AFB2 dual-fusion (YPH499)
# # 1Pat-05052023-IAAaccumulations_Expo1_IAAaccumulation_Anaerobic_yWL209-
Control.fcs  TIR1 dual-fusion (YPH499)
# # 1Pat-05052023-IAAaccumulations_Expo1_IAAaccumulation_Anaerobic_yWL209-
IAAspike.fcs TIR1 dual-fusion (YPH499)
# #
X.2
# # 1Pat-05052023-IAAaccumulations_Expo1_IAAaccumulation_Aerobic_yWL209-
Control.fcs     NA
# # 1Pat-05052023-IAAaccumulations_Expo1_IAAaccumulation_Aerobic_yWL209-
IAAspike.fcs    NA
# # 1Pat-05052023-IAAaccumulations_Expo1_IAAaccumulation_Aerobic_yWL210-
Control.fcs     NA
# # 1Pat-05052023-IAAaccumulations_Expo1_IAAaccumulation_Aerobic_yWL210-
IAAspike.fcs    NA
# # 1Pat-05052023-IAAaccumulations_Expo1_IAAaccumulation_Anaerobic_yWL209-
Control.fcs   NA
# # 1Pat-05052023-IAAaccumulations_Expo1_IAAaccumulation_Anaerobic_yWL209-
IAAspike.fcs  NA
# #
plot
# # 1Pat-05052023-IAAaccumulations_Expo1_IAAaccumulation_Aerobic_yWL209-
Control.fcs      no
# # 1Pat-05052023-IAAaccumulations_Expo1_IAAaccumulation_Aerobic_yWL209-
IAAspike.fcs     no
```

```
# # 1Pat-05052023-IAAaccumulations_Expo1_IAAaccumulation_Aerobic_yWL210-
Control.fcs      no
# # 1Pat-05052023-IAAaccumulations_Expo1_IAAaccumulation_Aerobic_yWL210-
IAAspike.fcs     no
# # 1Pat-05052023-IAAaccumulations_Expo1_IAAaccumulation_Anaerobic_yWL209-
Control.fcs    no
# # 1Pat-05052023-IAAaccumulations_Expo1_IAAaccumulation_Anaerobic_yWL209-
IAAspike.fcs   no


plate_05052023_IAAacc_sum <- summarizeFlow(aplate_05052023_IAAacc, channel =
c("BL1.A",
    "YL1.A"), gated = TRUE)
# # [1] "Summarizing all events..."

timecourse_210stat <- ggplot(subset(plate_05052023_IAAacc_sum, plot == "yes"
& condition_phase %in%
    c("Anaerobic late stationary", "Aerobic late stationary") & strain %in%
c("yWL210")),
    aes(x = time, y = YL1.Amean/BL1.Amean, color = treatment)) +
geom_point(size = 3) +
    geom_line(size = 2, alpha = 0.7) + facet_wrap(~condition_phase, scale =
"free_y") +
    scale_fill_manual(values = c("#a65c85ff", "#f9a242ff")) +
scale_colour_manual(name = "Auxin accumulation",
    limits = c("1 uM IAA", "Control"), labels = c("1 µM Auxin Spike", "Auxin
Production by Yeast"),
    values = c("#a65c85ff", "#f9a242ff")) + ylab("Detected
Auxin\n(mScarlet/Venus ratio)") +
    xlab("Time post treatment (mins)") + theme_classic() +
theme(legend.position = "bottom",
    axis.title.y = element_text(face = "bold", size = 12), axis.title.x =
element_text(face = "bold",
        size = 12), axis.text.y = element_text(size = 10), axis.text.x =
element_text(size = 10),
    legend.title = element_blank(), legend.text = element_text(size = 9))
timecourse_210stat
```

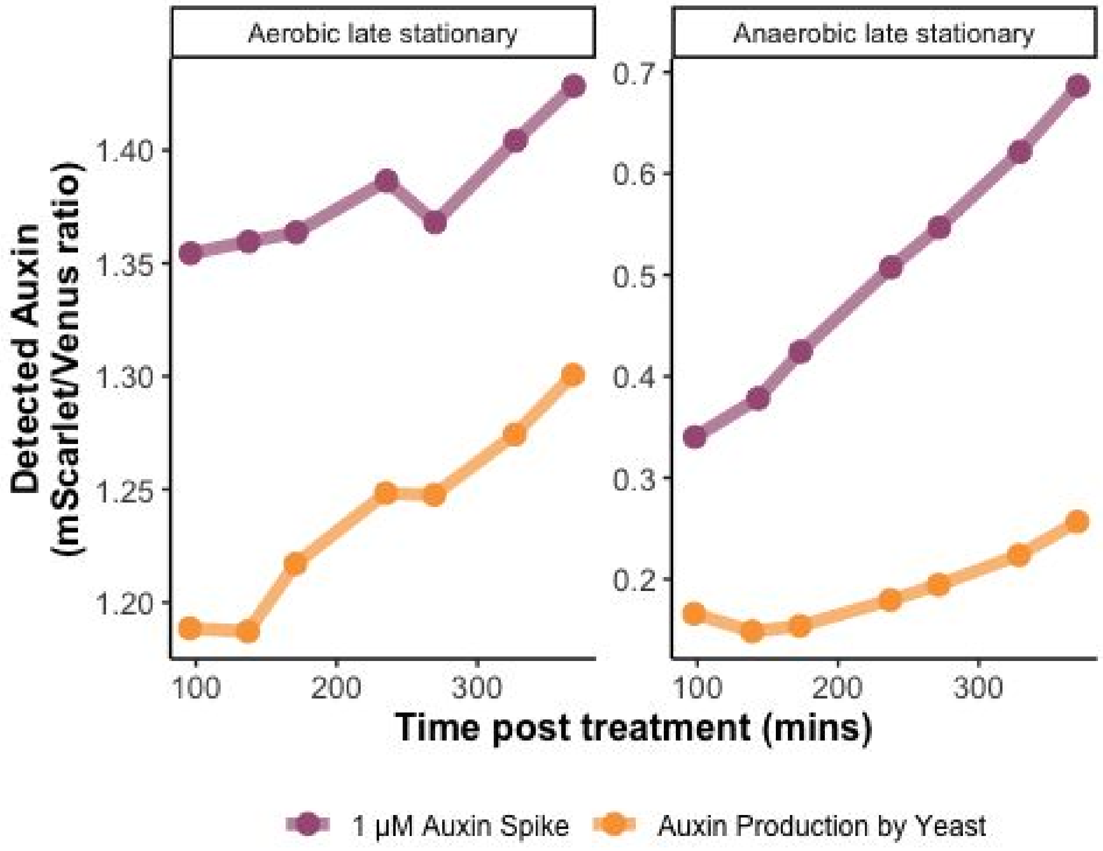

Figure 8C: The dual-fusion AFB2 biosensor predicts that auxin accumulates in stationary phase cultures

```
plate_05052023_IAAacc_sum2 <- steadyState(aplate_05052023_IAAacc, gated =
TRUE)
# # [1] "No further gating applied."
# # [1] "Converting events..."

plate_05052023_IAAacc_sum2 <- subset(plate_05052023_IAAacc_sum2, plot ==
"yes" &
    treatment %in% c("Control") & reading %in% c(6, 9, 16))


plate_05052023_IAAacc_sum2$BL1.A |>
    range()
# # [1]  -18675 1048575
plate_05052023_IAAacc_sum2 <- subset(plate_05052023_IAAacc_sum2, BL1.A > 0 &
BL1.A <
    1e+06 & YL1.A > 0 & YL1.A < 1e+06)

plate_05052023_IAAacc_sum2$condition_phase <-
factor(plate_05052023_IAAacc_sum2$condition_phase,
    levels = c("Aerobic exponential", "Anaerobic exponential", "Aerobic early
stationary",
```

```
        "Anaerobic early stationary", "Aerobic late stationary", "Anaerobic
late stationary"))

plate_05052023_IAAacc_sum2 |>
    group_by(condition_phase, reading, strain) |>
    summarise(count = n())
# # `summarise()` has grouped output by 'condition_phase', 'reading'. You can
# # override using the `.groups` argument.
# # # A tibble: 12 × 4
# # # Groups:   condition_phase, reading [6]
# #    condition_phase            reading strain count
# #    <fct>                        <int> <chr>  <int>
# #  1 Aerobic exponential              6 yWL209 44342
# #  2 Aerobic exponential              6 yWL210 45595
# #  3 Anaerobic exponential            6 yWL209 21808
# #  4 Anaerobic exponential            6 yWL210 17312
# #  5 Aerobic early stationary         9 yWL209 74032
# #  6 Aerobic early stationary         9 yWL210 68406
# #  7 Anaerobic early stationary       9 yWL209 32119
# #  8 Anaerobic early stationary       9 yWL210 22179
# #  9 Aerobic late stationary         16 yWL209 40012
# # 10 Aerobic late stationary         16 yWL210 50930
# # 11 Anaerobic late stationary       16 yWL209 37588
# # 12 Anaerobic late stationary       16 yWL210 30680

ridge210_expostat <- ggplot(subset(plate_05052023_IAAacc_sum2, strain ==
"yWL210"),
    aes(x = YL1.A/BL1.A, y = condition_phase)) + geom_density_ridges(aes(fill
= condition_phase),
    scale = 2, alpha = 0.8, quantile_lines = TRUE, quantiles = 4, color =
"#042333b2") +
    scale_fill_viridis_d(name = "Quartiles", option = "plasma", begin = 0.1,
end = 0.7,
        alpha = 0.3) + scale_color_viridis_d(name = "Quartiles", option =
"plasma",
    begin = 0.1, end = 0.7, alpha = 0.3) + ylab("Phase and growth
conditions") +
    xlab("mScarlet/Venus ratio\n(Accumulated Auxin Production)") +
theme_classic() +
    theme(axis.title.y = element_text(size = 12, face = "bold"), axis.title.x
= element_text(size = 12,
        face = "bold"), axis.text = element_text(size = 10), legend.title =
element_text(size = 12,
        face = "bold"), legend.text = element_text(size = 10),
legend.position = "none") +
    scale_x_log10()
ridge210_expostat
# # Picking joint bandwidth of 0.0139
```

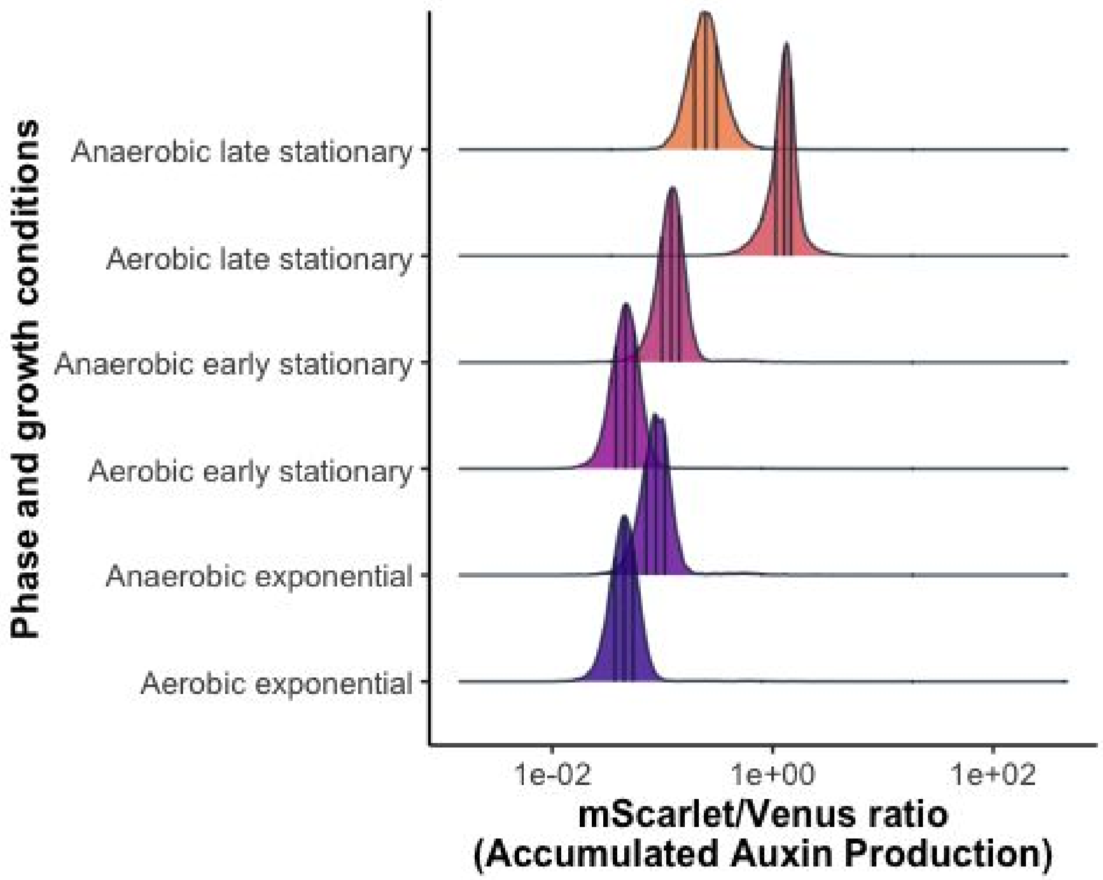


```
ridge210_expostat_fixaxis <- ridge210_expostat + theme(axis.text.y =
element_blank()) +
    annotate(geom = "text", x = 35, y = 6.2, label = "Anaerobic late
stationary",
        size = 4) + annotate(geom = "text", x = 35, y = 5.2, label = "Aerobic
late stationary",
    size = 4) + annotate(geom = "text", x = 35, y = 4.2, label = "Anaerobic
early stationary",
    size = 4) + annotate(geom = "text", x = 35, y = 3.2, label = "Aerobic
early stationary",
    size = 4) + annotate(geom = "text", x = 35, y = 2.2, label = "Anaerobic
exponential",
    size = 4) + annotate(geom = "text", x = 35, y = 1.2, label = "Aerobic
exponential",
    size = 4)
ridge210_expostat_fixaxis
# # Picking joint bandwidth of 0.0139
```

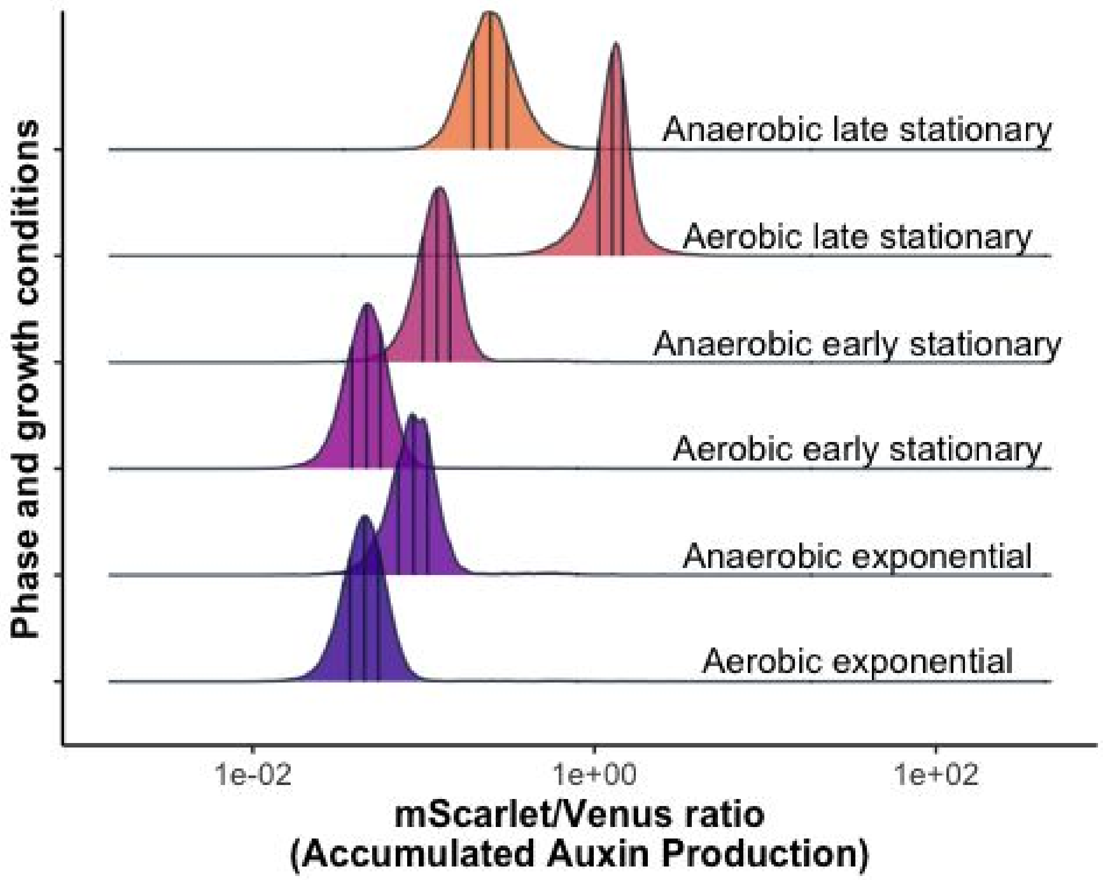


```
fig8AB <- ggarrange(plot210_stat_rep, timecourse_210stat, nrow = 2, ncol = 1,
labels = c("A",
    "B"), heights = c(1, 1))
fig8AB
```

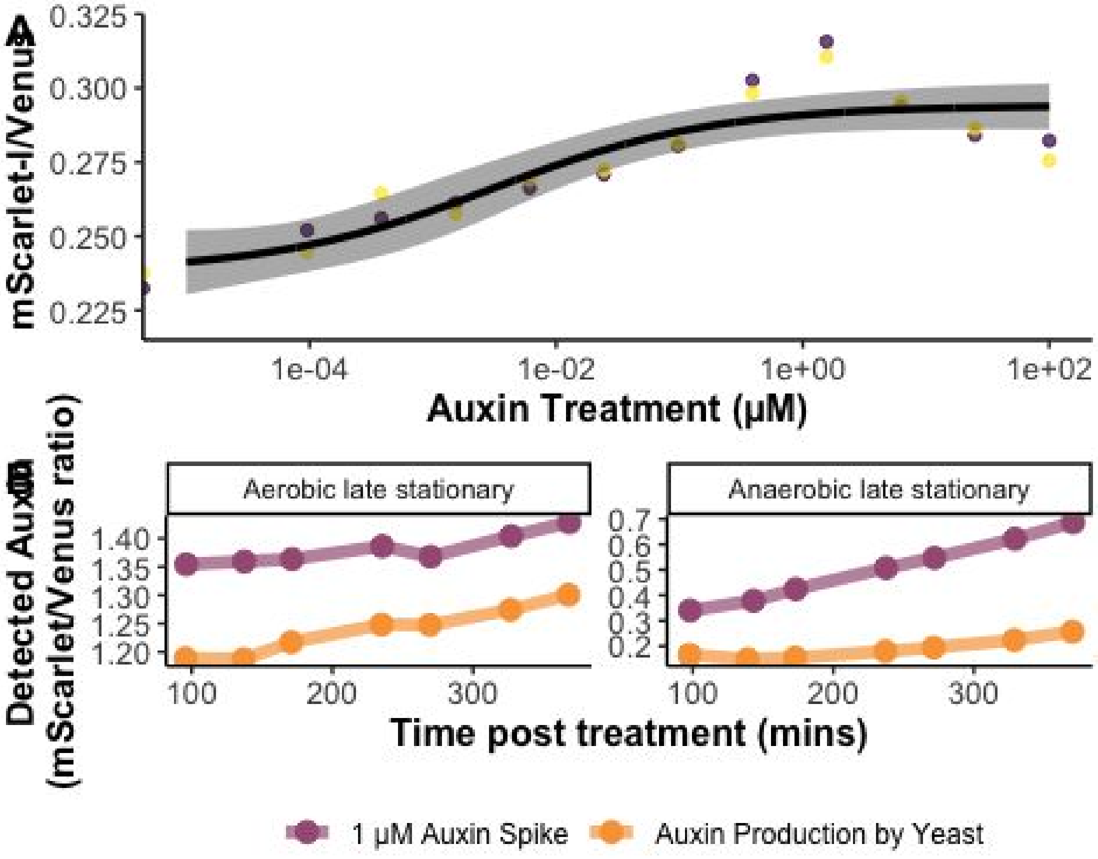


```
fig8C <- ggarrange(ridge210_expostat_fixaxis, nrow = 1, ncol = 1, labels =
c("C"))
# # Picking joint bandwidth of 0.0139
fig8C
```

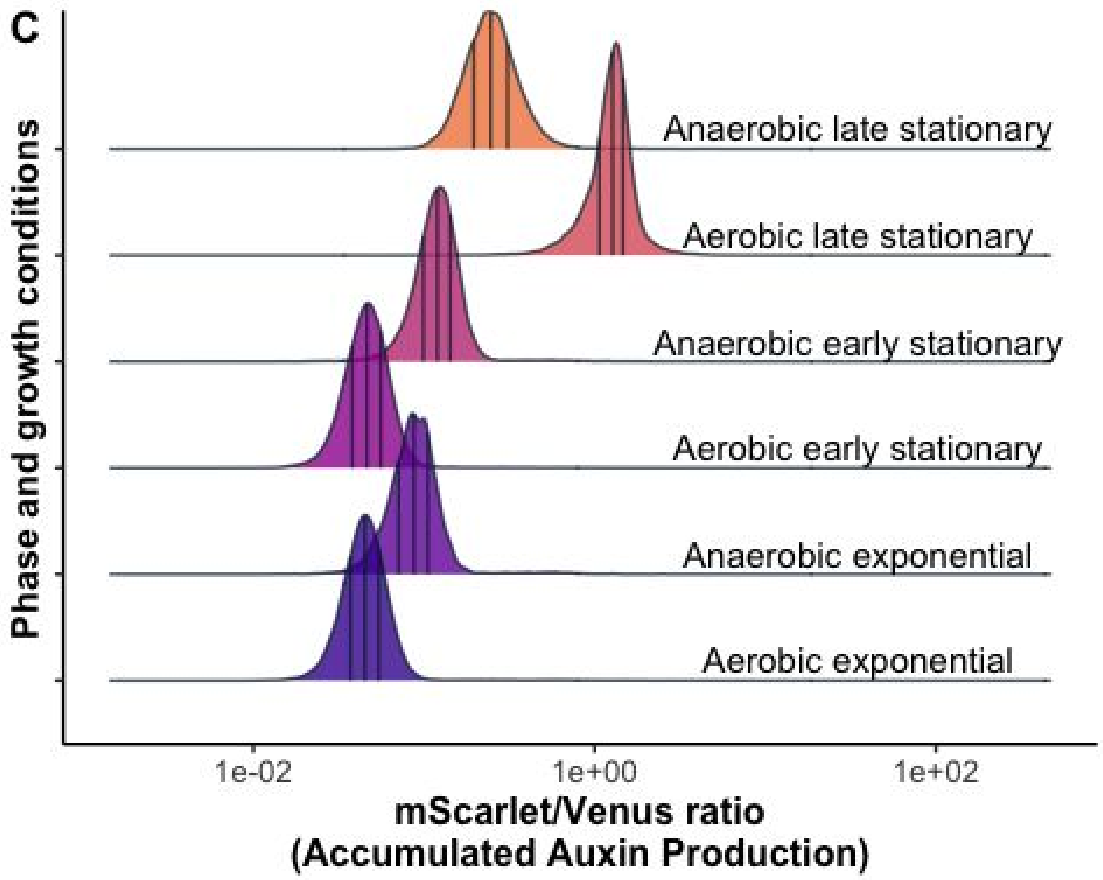


```r
fig8ABC <- ggarrange(fig8AB, fig8C, nrow = 1, ncol = 2, widths = c(0.8, 1))
fig8ABC
```

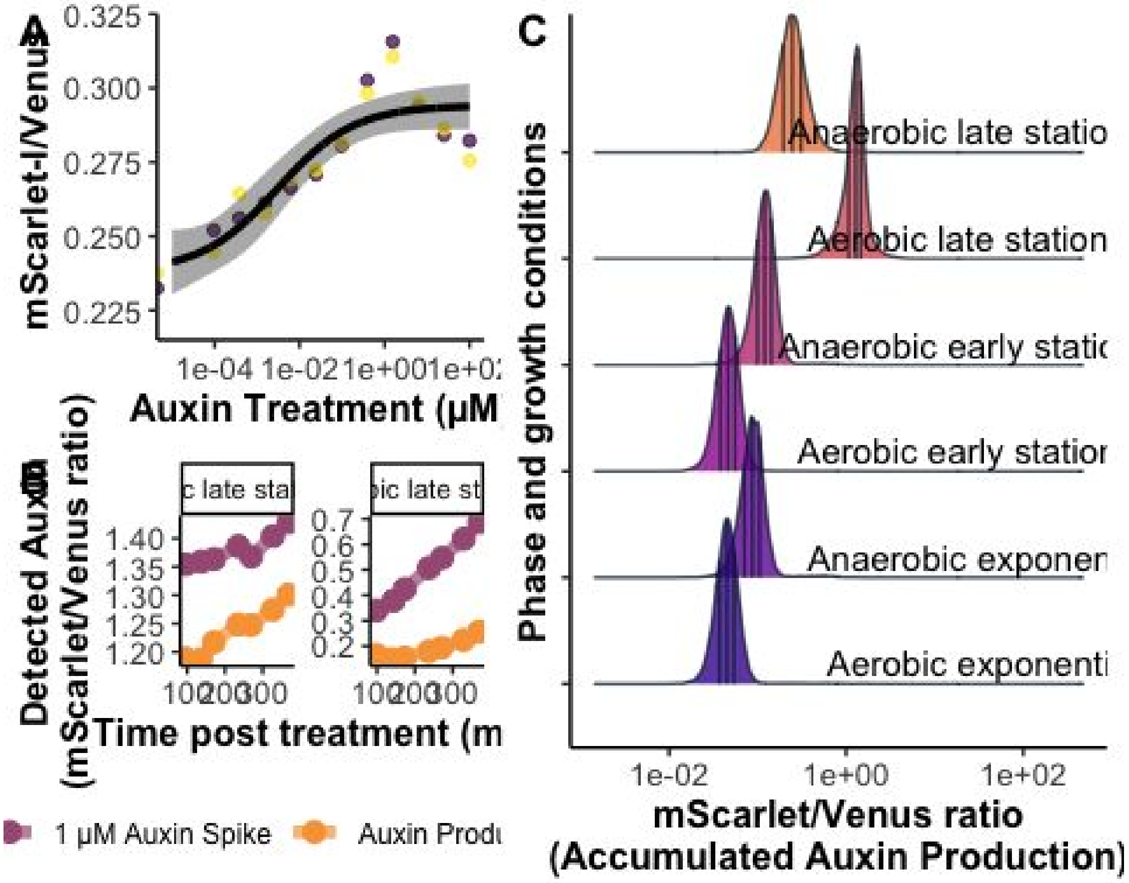


```
# ggsave('Figure8.png', width = 10, height = 6)


fig8new <- ggarrange(plot210_stat_rep, timecourse_210stat,
ridge210_expostat_fixaxis,
    nrow = 1, ncol = 3, labels = c("A", "B", "C"), widths = c(0.8, 1, 1))
# # Picking joint bandwidth of 0.0139
fig8new
```

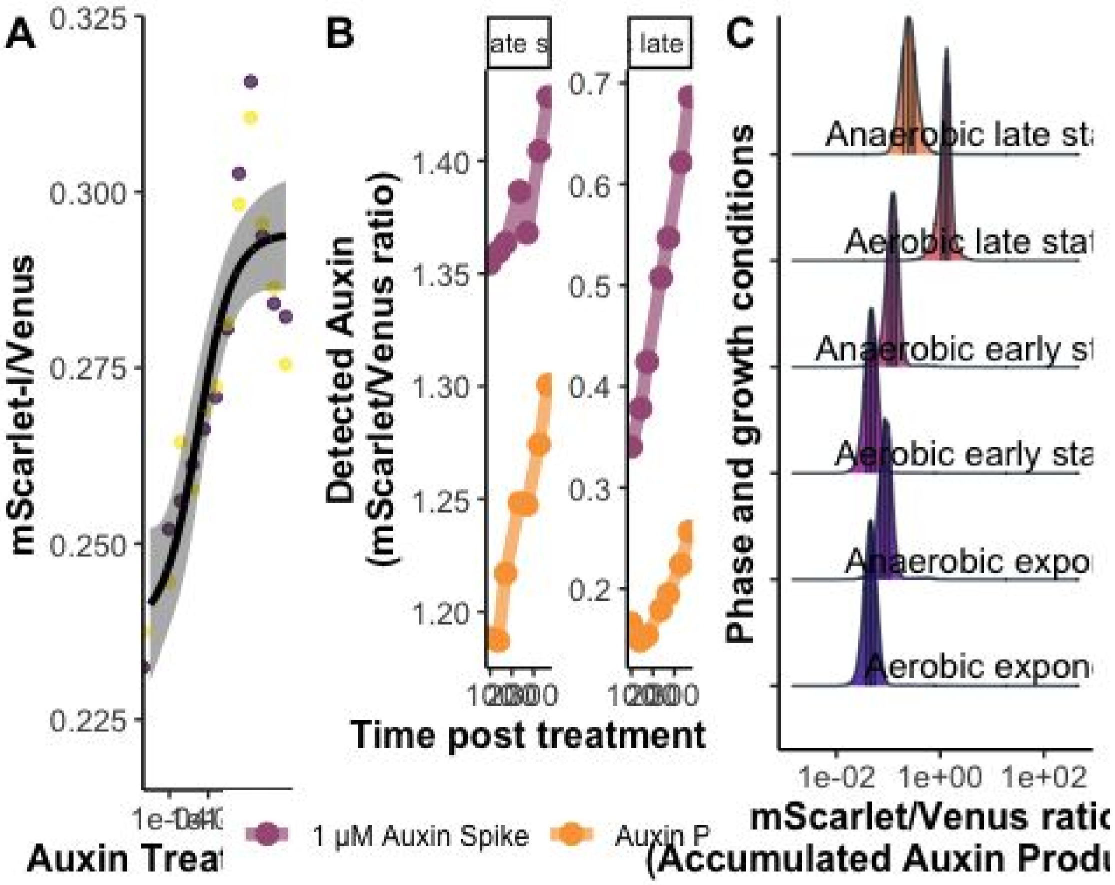


```
# ggsave('fig8new.png', width = 12, height = 4)
```

Supporting Figure S9: Cell growth and conditions during time-course assay

Supporting Figure S9A: At exponential phase

```
plate_05052023_IAAacc_sum <- summarizeFlow(aplate_05052023_IAAacc, channel =
c("BL1.A",
    "YL1.A"), gated = TRUE)
# # [1] "Summarizing all events..."

# plate_05052023_IAAacc_sum$treatment <-
# factor(plate_05052023_IAAacc_sum$treatment, levels = c('Control', '1 uM
# Auxin'))

# The time auxin addition is equal to time zero
time0 <- "4Pat-05052023-
IAAaccumulations_Expo4_IAAaccumulation_Aerobic_yWL209-Control.fcs"
# or whatever well was being read when auxin was added

plate_05052023_IAAacc_sum$time <- plate_05052023_IAAacc_sum$btime -
plate_05052023_IAAacc_sum[[which(plate_05052023_IAAacc_sum$name ==
    time0), "btime"]]
```

```r
# single bracket -->extracting the all name, 2 brackets extract just single
# 'value' or 'values'

growth_expo <- ggplot(subset(plate_05052023_IAAacc_sum, phase ==
"exponential"),
    aes(x = time, y = log(conc), group = treatment)) + geom_line(aes(color =
treatment),
    size = 2, alpha = 0.8) + geom_point(aes(color = treatment), size = 2,
alpha = 0.7) +
    scale_color_manual(values = c("#593d9cff", "#a65c85ff")) +
geom_jitter(aes(color = treatment),
    position = position_jitterdodge(jitter.width = 0.1, dodge.width = 0.7),
size = 2) +
    facet_wrap(design_yeast ~ condition) + ylim(5.5, 8.5) + ylab("Log cell
density") +
    xlab("Time (min)") + theme_classic() + theme(legend.position = "none",
axis.title.y = element_text(size = 12,
    face = "bold"), axis.title.x = element_text(size = 12, face = "bold"),
axis.text = element_text(size = 8),
    legend.title = element_text(size = 10, face = "bold"), legend.text =
element_text(size = 8))
growth_expo
```

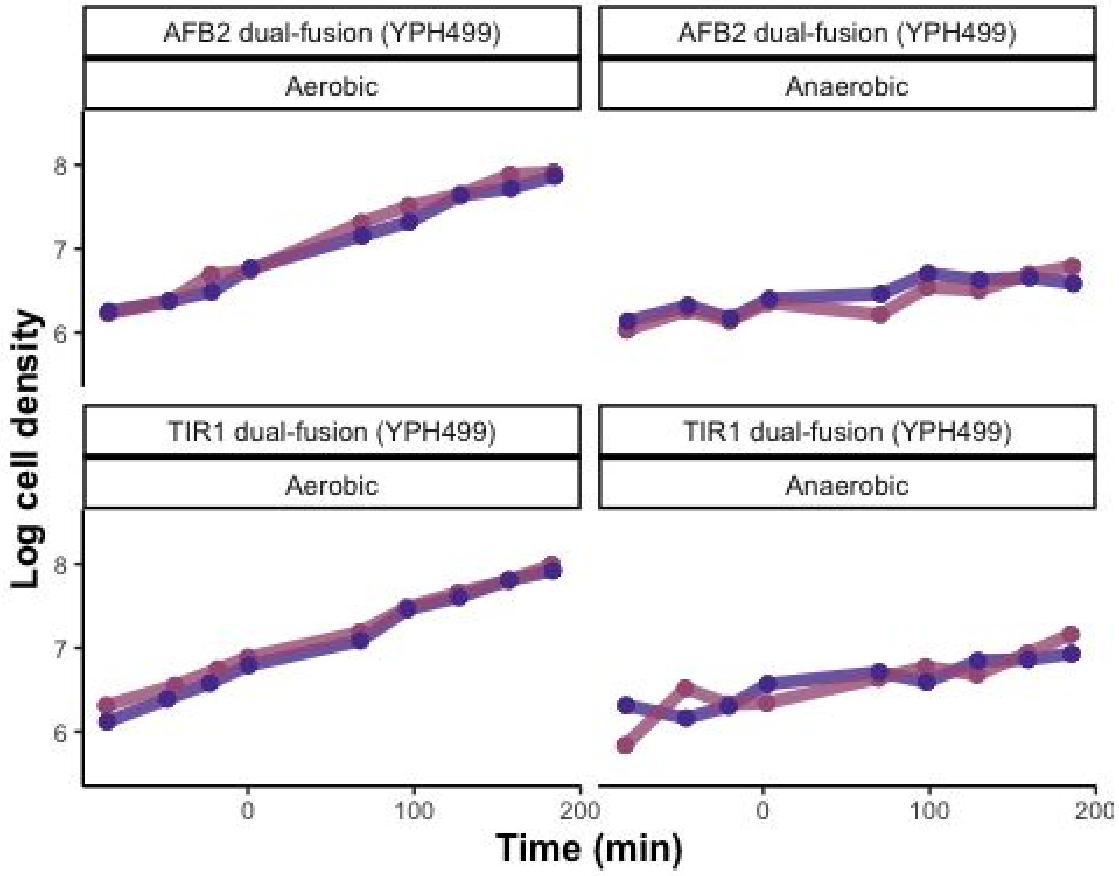

Supporting Figure S9B: At stationary phase

```
growth_stat <- ggplot(subset(plate_05052023_IAAacc_sum, phase ==
"stationary"), aes(x = time,
    y = log(conc), group = treatment)) + geom_line(aes(color = treatment),
size = 2,
    alpha = 0.8) + geom_point(aes(color = treatment), size = 2, alpha = 0.7)
+ scale_color_manual(values = c("#593d9cff",
    "#a65c85ff")) + geom_jitter(aes(color = treatment), position =
position_jitterdodge(jitter.width = 0.1,
    dodge.width = 0.7), size = 2) + facet_wrap(design_yeast ~ condition) +
ylim(5.5,
    8.5) + ylab("Log cell density") + xlab("Time (min)") + theme_classic() +
theme(legend.position = "none",
    axis.title.y = element_text(size = 12, face = "bold"), axis.title.x =
element_text(size = 12,
        face = "bold"), axis.text = element_text(size = 8), legend.title =
element_text(size = 10,
        face = "bold"), legend.text = element_text(size = 8))
growth_stat
```

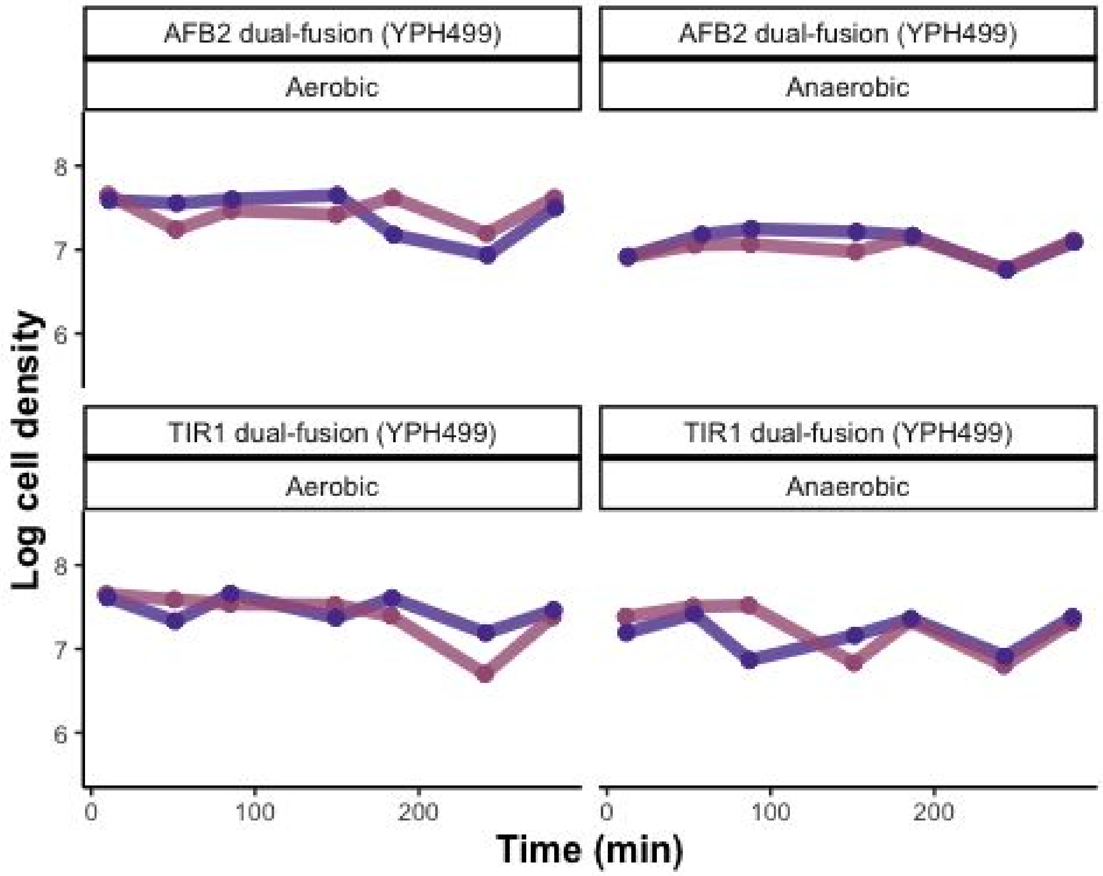


```
cellgrowth <- ggarrange(growth_expo, growth_stat, ncol = 2, nrow = 1, labels
= c("A",
```

```
    "B"), common.legend = TRUE)
# cellgrowth

# ggsave('Supplement7.pdf', height = 5, width = 8)
```

Supporting Figure S8: AFB2 dual-fusion biosensor during dose-response assay

```
plate_08052022_aftertreat_SS <- read.plateSet(path = "Data for
publication/08052022_yWL210_DRA-LCMS-R2/after_treatment_SS/",
    pattern = "DRA*")

annotation <- createAnnotation(yourFlowSet = plate_08052022_aftertreat_SS)
write.csv(annotation, "Data for publication/08052022_yWL210_DRA-LCMS-
R2/08052022_aftertreat_SS_annotation.csv")

annotation <- read.csv("Data for publication/08052022_yWL210_DRA-LCMS-
R2/08052022_aftertreat_SS_annotation.csv")

aplate_08052022_aftertreat_SS <- annotateFlowSet(yourFlowSet =
plate_08052022_aftertreat_SS,
    annotation_df = annotation, mergeBy = "name")
head(rownames(pData(aplate_08052022_aftertreat_SS)))
# # [1] "1Pat-08052022-Biosensor_LCMS_rep2-DRA09_DRA-LCMS-R2_Exponential_0
uM.fcs"
# # [2] "1Pat-08052022-Biosensor_LCMS_rep2-DRA09_DRA-LCMS-
R2_Exponential_0.0064 uM.fcs"
# # [3] "1Pat-08052022-Biosensor_LCMS_rep2-DRA09_DRA-LCMS-
R2_Exponential_0.032 uM.fcs"
# # [4] "1Pat-08052022-Biosensor_LCMS_rep2-DRA09_DRA-LCMS-R2_Exponential_0.16
uM.fcs"
# # [5] "1Pat-08052022-Biosensor_LCMS_rep2-DRA09_DRA-LCMS-R2_Exponential_0.8
uM.fcs"
# # [6] "1Pat-08052022-Biosensor_LCMS_rep2-DRA09_DRA-LCMS-R2_Exponential_100
uM.fcs"
head(pData(aplate_08052022_aftertreat_SS))
# #
name
# # 1Pat-08052022-Biosensor_LCMS_rep2-DRA09_DRA-LCMS-R2_Exponential_0 uM.fcs
1Pat-08052022-Biosensor_LCMS_rep2-DRA09_DRA-LCMS-R2_Exponential_0 uM.fcs
# # 1Pat-08052022-Biosensor_LCMS_rep2-DRA09_DRA-LCMS-R2_Exponential_0.0064
uM.fcs 1Pat-08052022-Biosensor_LCMS_rep2-DRA09_DRA-LCMS-R2_Exponential_0.0064
uM.fcs
# # 1Pat-08052022-Biosensor_LCMS_rep2-DRA09_DRA-LCMS-R2_Exponential_0.032
uM.fcs   1Pat-08052022-Biosensor_LCMS_rep2-DRA09_DRA-LCMS-
R2_Exponential_0.032 uM.fcs
# # 1Pat-08052022-Biosensor_LCMS_rep2-DRA09_DRA-LCMS-R2_Exponential_0.16
uM.fcs     1Pat-08052022-Biosensor_LCMS_rep2-DRA09_DRA-LCMS-
R2_Exponential_0.16 uM.fcs
# # 1Pat-08052022-Biosensor_LCMS_rep2-DRA09_DRA-LCMS-R2_Exponential_0.8
uM.fcs       1Pat-08052022-Biosensor_LCMS_rep2-DRA09_DRA-LCMS-
R2_Exponential_0.8 uM.fcs
```

```
# # 1Pat-08052022-Biosensor_LCMS_rep2-DRA09_DRA-LCMS-R2_Exponential_100
uM.fcs       1Pat-08052022-Biosensor_LCMS_rep2-DRA09_DRA-LCMS-
R2_Exponential_100 uM.fcs
# #
folder
# # 1Pat-08052022-Biosensor_LCMS_rep2-DRA09_DRA-LCMS-R2_Exponential_0 uM.fcs
DRA09
# # 1Pat-08052022-Biosensor_LCMS_rep2-DRA09_DRA-LCMS-R2_Exponential_0.0064
uM.fcs  DRA09
# # 1Pat-08052022-Biosensor_LCMS_rep2-DRA09_DRA-LCMS-R2_Exponential_0.032
uM.fcs   DRA09
# # 1Pat-08052022-Biosensor_LCMS_rep2-DRA09_DRA-LCMS-R2_Exponential_0.16
uM.fcs    DRA09
# # 1Pat-08052022-Biosensor_LCMS_rep2-DRA09_DRA-LCMS-R2_Exponential_0.8
uM.fcs     DRA09
# # 1Pat-08052022-Biosensor_LCMS_rep2-DRA09_DRA-LCMS-R2_Exponential_100
uM.fcs     DRA09
# #
X
# # 1Pat-08052022-Biosensor_LCMS_rep2-DRA09_DRA-LCMS-R2_Exponential_0 uM.fcs
1
# # 1Pat-08052022-Biosensor_LCMS_rep2-DRA09_DRA-LCMS-R2_Exponential_0.0064
uM.fcs 2
# # 1Pat-08052022-Biosensor_LCMS_rep2-DRA09_DRA-LCMS-R2_Exponential_0.032
uM.fcs  3
# # 1Pat-08052022-Biosensor_LCMS_rep2-DRA09_DRA-LCMS-R2_Exponential_0.16
uM.fcs   4
# # 1Pat-08052022-Biosensor_LCMS_rep2-DRA09_DRA-LCMS-R2_Exponential_0.8
uM.fcs    5
# # 1Pat-08052022-Biosensor_LCMS_rep2-DRA09_DRA-LCMS-R2_Exponential_100
uM.fcs    6
# #
treatment
# # 1Pat-08052022-Biosensor_LCMS_rep2-DRA09_DRA-LCMS-R2_Exponential_0 uM.fcs
0 uM
# # 1Pat-08052022-Biosensor_LCMS_rep2-DRA09_DRA-LCMS-R2_Exponential_0.0064
uM.fcs 0.0064 uM
# # 1Pat-08052022-Biosensor_LCMS_rep2-DRA09_DRA-LCMS-R2_Exponential_0.032
uM.fcs   0.032 uM
# # 1Pat-08052022-Biosensor_LCMS_rep2-DRA09_DRA-LCMS-R2_Exponential_0.16
uM.fcs     0.16 uM
# # 1Pat-08052022-Biosensor_LCMS_rep2-DRA09_DRA-LCMS-R2_Exponential_0.8
uM.fcs       0.8 uM
# # 1Pat-08052022-Biosensor_LCMS_rep2-DRA09_DRA-LCMS-R2_Exponential_100
uM.fcs       100 uM
# #
reading
# # 1Pat-08052022-Biosensor_LCMS_rep2-DRA09_DRA-LCMS-R2_Exponential_0 uM.fcs
9
# # 1Pat-08052022-Biosensor_LCMS_rep2-DRA09_DRA-LCMS-R2_Exponential_0.0064
```

```
uM.fcs       9
# # 1Pat-08052022-Biosensor_LCMS_rep2-DRA09_DRA-LCMS-R2_Exponential_0.032
uM.fcs        9
# # 1Pat-08052022-Biosensor_LCMS_rep2-DRA09_DRA-LCMS-R2_Exponential_0.16
uM.fcs         9
# # 1Pat-08052022-Biosensor_LCMS_rep2-DRA09_DRA-LCMS-R2_Exponential_0.8
uM.fcs          9
# # 1Pat-08052022-Biosensor_LCMS_rep2-DRA09_DRA-LCMS-R2_Exponential_100
uM.fcs          9
# #
dose
# # 1Pat-08052022-Biosensor_LCMS_rep2-DRA09_DRA-LCMS-R2_Exponential_0 uM.fcs
0.0e+00
# # 1Pat-08052022-Biosensor_LCMS_rep2-DRA09_DRA-LCMS-R2_Exponential_0.0064
uM.fcs 6.4e-03
# # 1Pat-08052022-Biosensor_LCMS_rep2-DRA09_DRA-LCMS-R2_Exponential_0.032
uM.fcs  3.2e-02
# # 1Pat-08052022-Biosensor_LCMS_rep2-DRA09_DRA-LCMS-R2_Exponential_0.16
uM.fcs   1.6e-01
# # 1Pat-08052022-Biosensor_LCMS_rep2-DRA09_DRA-LCMS-R2_Exponential_0.8
uM.fcs    8.0e-01
# # 1Pat-08052022-Biosensor_LCMS_rep2-DRA09_DRA-LCMS-R2_Exponential_100
uM.fcs    1.0e+02
# #
collection
# # 1Pat-08052022-Biosensor_LCMS_rep2-DRA09_DRA-LCMS-R2_Exponential_0 uM.fcs
end
# # 1Pat-08052022-Biosensor_LCMS_rep2-DRA09_DRA-LCMS-R2_Exponential_0.0064
uM.fcs        end
# # 1Pat-08052022-Biosensor_LCMS_rep2-DRA09_DRA-LCMS-R2_Exponential_0.032
uM.fcs         end
# # 1Pat-08052022-Biosensor_LCMS_rep2-DRA09_DRA-LCMS-R2_Exponential_0.16
uM.fcs          end
# # 1Pat-08052022-Biosensor_LCMS_rep2-DRA09_DRA-LCMS-R2_Exponential_0.8
uM.fcs           end
# # 1Pat-08052022-Biosensor_LCMS_rep2-DRA09_DRA-LCMS-R2_Exponential_100
uM.fcs           end
# #
before_after
# # 1Pat-08052022-Biosensor_LCMS_rep2-DRA09_DRA-LCMS-R2_Exponential_0 uM.fcs
After
# # 1Pat-08052022-Biosensor_LCMS_rep2-DRA09_DRA-LCMS-R2_Exponential_0.0064
uM.fcs        After
# # 1Pat-08052022-Biosensor_LCMS_rep2-DRA09_DRA-LCMS-R2_Exponential_0.032
uM.fcs         After
# # 1Pat-08052022-Biosensor_LCMS_rep2-DRA09_DRA-LCMS-R2_Exponential_0.16
uM.fcs          After
# # 1Pat-08052022-Biosensor_LCMS_rep2-DRA09_DRA-LCMS-R2_Exponential_0.8
uM.fcs           After
```

```
# # 1Pat-08052022-Biosensor_LCMS_rep2-DRA09_DRA-LCMS-R2_Exponential_100
uM.fcs           After
```

Supporting Figure S8A: Cell growth during dose-response assay

```
plate_08052022_sum <- summarizeFlow(aplate_08052022, channel = c("BL1.A",
"YL1.A"),
    gated = TRUE)
# # [1] "Summarizing all events..."

time0 <- "5Pat-08052022-Biosensor_LCMS_rep2-DRA05_DRA-LCMS-R2_Exponential_0
uM.fcs"


plate_08052022_sum$time <- plate_08052022_sum$btime -
plate_08052022_sum[[which(plate_08052022_sum$name ==
    time0), "btime"]]


growth <- subset(plate_08052022_sum, collection == "end") %>%
    mutate(treatment = fct_relevel(treatment, "0 uM", "0.0064 uM", "0.032
uM", "0.16 uM",
        "0.8 uM", "4 uM", "20 uM", "100 uM")) %>%
    ggplot(aes(x = time, y = conc, color = factor(treatment))) +
geom_point(aes(color = treatment),
    size = 3, shape = 19) + scale_colour_viridis_d(option = "plasma", alpha =
0.7,
    begin = 0, end = 0.8) + geom_line(aes(color = treatment), size = 2, alpha
= 0.7) +
    guides(color = guide_legend(title = "Auxin\nTreatments")) + xlab("Time
post treatment (min)") +
    ylab("Cell concentration") + theme_classic() + theme(axis.title =
element_text(size = 12,
    face = "bold"), axis.text = element_text(size = 10), legend.title =
element_text(size = 10),
    legend.text = element_text(size = 11), legend.position = "right")

growth
```

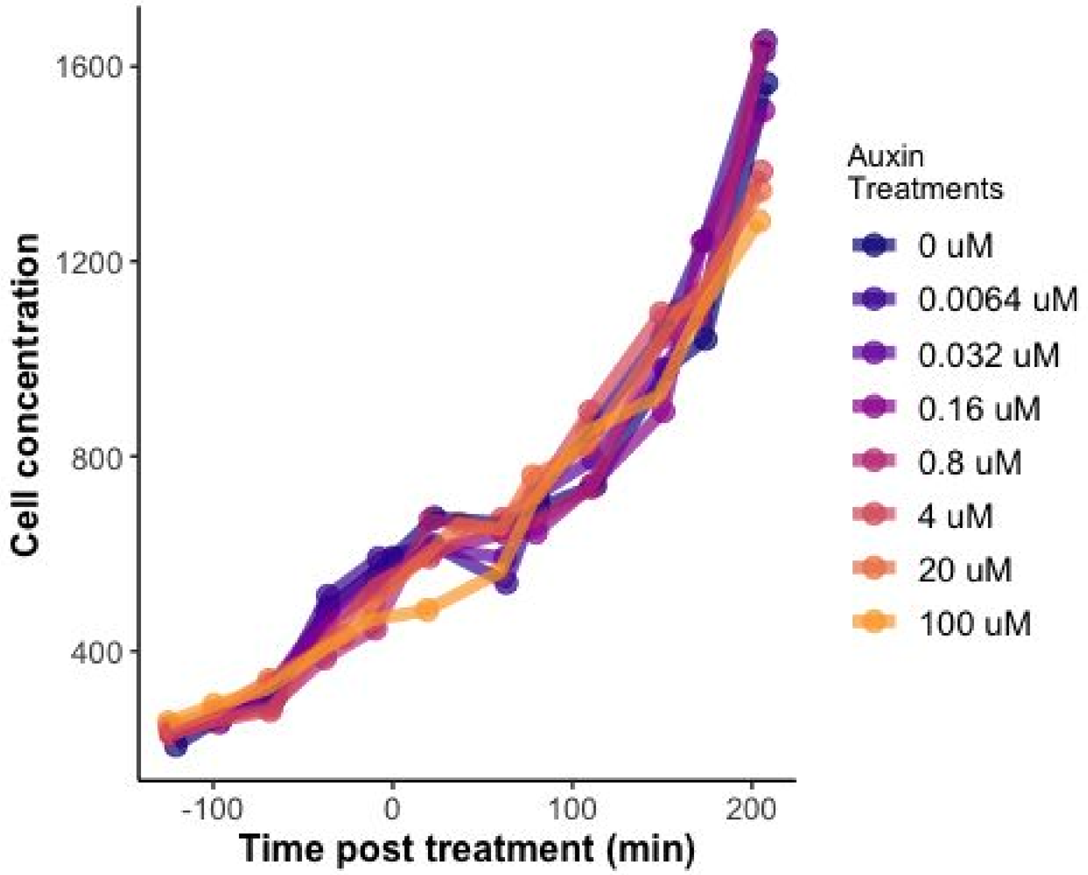


```
log_growth <- subset(plate_08052022_sum, collection == "end") %>%
    mutate(treatment = fct_relevel(treatment, "0 uM", "0.0064 uM", "0.032
uM", "0.16 uM",
        "0.8 uM", "4 uM", "20 uM", "100 uM")) %>%
    ggplot(aes(x = time, y = log(conc), color = factor(treatment))) +
geom_point(aes(color = treatment),
    size = 3, shape = 19) + geom_line(aes(color = treatment), size = 2, alpha
= 0.7) +
    guides(color = guide_legend(title = "Auxin\nTreatments")) +
scale_colour_viridis_d(option = "plasma",
    alpha = 0.7, begin = 0.4, end = 0.8) + xlab("Time post treatment (min)")
+ ylab("Log cell density (cells/µl)") +
    theme_classic() + theme(axis.title = element_text(size = 12, face =
"bold"),
    axis.text = element_text(size = 10), legend.title = element_text(size =
10),
    legend.text = element_text(size = 10), legend.position = "bottom")

log_growth
```

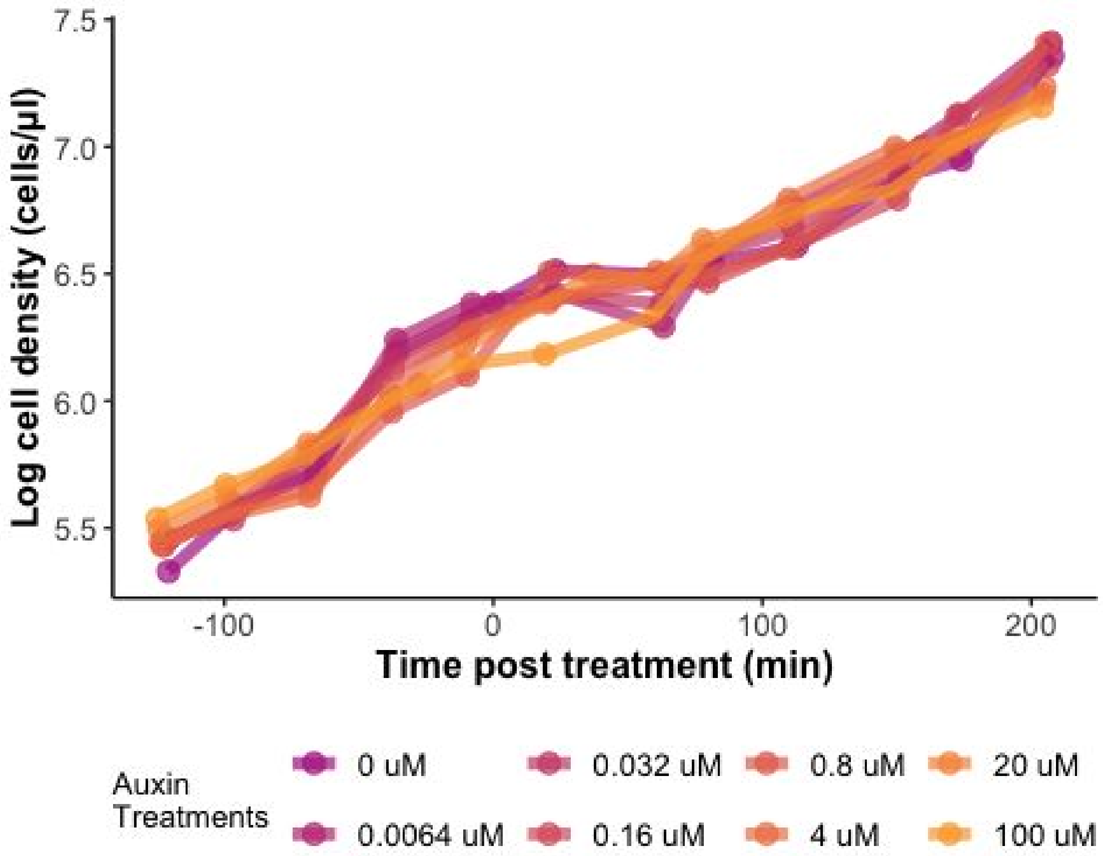

Supporting Figure S8B: Venus-IAA17/mScarlet-AFB2 ratio in response to different doses of auxin

```
ratio_line <- subset(plate_08052022_sum, collection == "end") %>%
    mutate(treatment = fct_relevel(treatment, "0 uM", "0.0064 uM", "0.032
uM", "0.16 uM",
        "0.8 uM", "4 uM", "20 uM", "100 uM")) %>%
    ggplot(aes(x = time, y = YL1.Amean/BL1.Amean, color = factor(treatment)))
+ geom_point(aes(color = treatment),
    size = 3, shape = 19) + scale_colour_viridis_d(option = "plasma", alpha =
0.7,
    begin = 0.4, end = 0.8) + geom_line(aes(color = treatment), size = 2,
alpha = 0.7) +
    guides(color = guide_legend(title = "Auxin\nTreatments")) + xlab("Time
post treatment (min)") +
    ylab("Venus/mScarlet-I ratio") + theme_classic() + theme(axis.title =
element_text(size = 12,
    face = "bold"), axis.text = element_text(size = 10), legend.title =
element_text(size = 10),
    legend.text = element_text(size = 11), legend.position = "bottom") +
ylab("Venus/mScarlet-I") +
    xlab("Time post treatment (min)") + stat_compare_means(label =
```

```
"p.signif", method = "anova",
    ref.group = "0 uM")

ratio_line
```

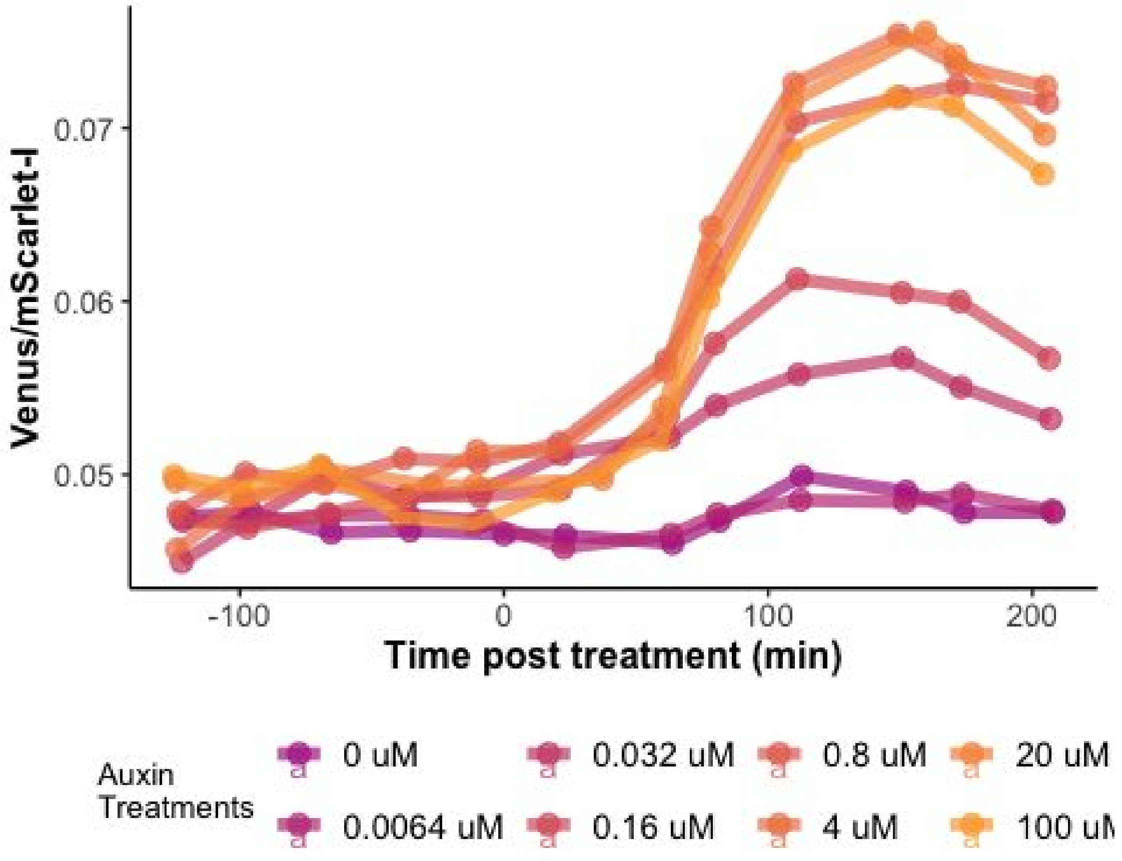


```
levels(plate_08052022_sum$treatment)
# # NULL
plate_08052022_sum$treatment <- factor(plate_08052022_sum$treatment, levels =
c("0 uM",
    "0.0064 uM", "0.032 uM", "0.16 uM", "0.8 uM", "4 uM", "20 uM", "100 uM"))
```

Supplement 6B: The bar plot of the foldchange between different doses of auxin treatment and solvent control

```
DRA_different <- plate_08052022_sum %>%
    dplyr::filter(reading %in% c("10", "11", "12")) %>%
    dplyr::filter(collection == "end") %>%
    mutate(Ratio_DRA = YL1.Amean/BL1.Amean)



# Create a variable to keep track of whether to subtract or not
```

```
subtract_flag <- FALSE
x <- 0

# Iterate through each row
for (i in 1:nrow(DRA_different)) {
    if (DRA_different$treatment[i] == "0 uM") {
        subtract_flag <- TRUE
        x <- DRA_different$Ratio_DRA[i]
        DRA_different$Ratio_DRA[i] <- 0
    } else if (subtract_flag) {
        DRA_different$Ratio_DRA[i] <- DRA_different$Ratio_DRA[i] - x
    }
}

print(DRA_different)
# # # A tibble: 24 × 20
# #    name           time btime atime[,1] events[,1] conc[,1] folder     X
treatment
# #    <chr>         <dbl> <dbl>     <dbl>      <int>    <dbl> <chr>  <int>
<fct>
# #  1 10Pat-08052…  152.  905.     0.292      29348     978. DRA10    109 0
uM
# #  2 10Pat-08052…  152.  904.     0.292      31776    1059. DRA10    110
0.0064 uM
# #  3 10Pat-08052…  151.  903.     0.294      29377     979. DRA10    111
0.032 uM
# #  4 10Pat-08052…  151.  903.     0.293      26773     892. DRA10    112
0.16 uM
# #  5 10Pat-08052…  150.  902.     0.292      31354    1045. DRA10    113
0.8 uM
# #  6 10Pat-08052…  148.  901.     0.294      27722     924. DRA10    114
100 uM
# #  7 10Pat-08052…  159.  912.     0.294      32989    1100. DRA10    115 20
uM
# #  8 10Pat-08052…  149.  902.     0.292      32800    1093. DRA10    116 4
uM
# #  9 11Pat-08052…  174.  926.     0.294      31209    1040. DRA11    121 0
uM
# # 10 11Pat-08052…  174.  926.     0.292      37309    1244. DRA11    122
0.0064 uM
# # # ℹ 14 more rows
# # # ℹ 11 more variables: reading <int>, dose <dbl>, collection <chr>,
# # #   before_after <chr>, BL1.Amean <dbl>, BL1.Amedian <dbl>, BL1.Asd
<dbl>,
# # #   YL1.Amean <dbl>, YL1.Amedian <dbl>, YL1.Asd <dbl>, Ratio_DRA <dbl>

# my_comparisons <- list(c('0 uM', '0.0064 uM'),c('0 uM', '0.032 uM'),c('0
uM',
# '0.16 uM'),c('0 uM', '0.8 uM') ,c('0 uM', '4 uM'), c('0 uM', '20 uM'), c('0
# uM', '100 uM'))
```

```
fc_DRA <- ggplot(DRA_different, aes(x = treatment, y = Ratio_DRA, fill =
treatment,
    colour = treatment)) + geom_boxplot(alpha = 0.5) +
scale_fill_viridis_d(name = "dose",
    option = "plasma", begin = 0.4, end = 0.8, alpha = 0.5) +
scale_color_viridis_d(name = "dose",
    option = "plasma", begin = 0.1, end = 0.8) + theme_classic() +
theme(axis.title.y = element_text(size = 12,
    face = "bold"), axis.title.x = element_text(size = 12, face = "bold"),
axis.text = element_text(size = 10),
    legend.title = element_text(size = 11, face = "bold"), legend.text =
element_text(size = 8),
    legend.position = "none") + ylab("mScarlet-I/Venus") + xlab("Dose of
auxin (μM)") +
    stat_compare_means(label = "p.signif", method = "t.test", ref.group = "0
uM",
        label.y = 0.003, size = 5) + scale_x_discrete(labels = c(`0 uM` =
"0", `0.0064 uM` = "0.0064",
    `0.032 uM` = "0.032", `0.16 uM` = "0.16", `0.8 uM` = "0.8", `4 uM` = "4",
`20 uM` = "20",
    `100 uM` = "100"))

fc_DRA
```

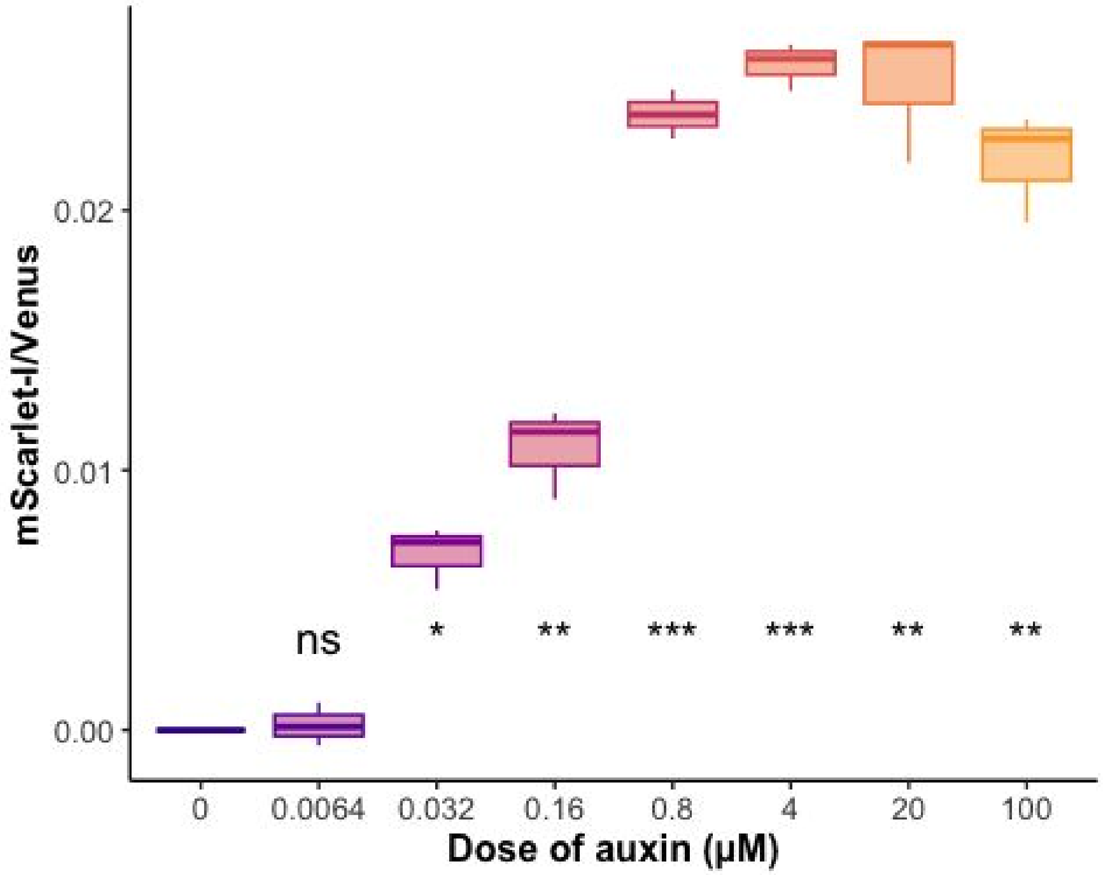


```
Sup6_ABC <- ggarrange(log_growth, ratio_line, fc_DRA, ncol = 3, nrow = 1,
labels = c("A",
    "B", "C"), common.legend = TRUE, widths = c(0.5, 0.5, 1))

Sup6_ABC
```

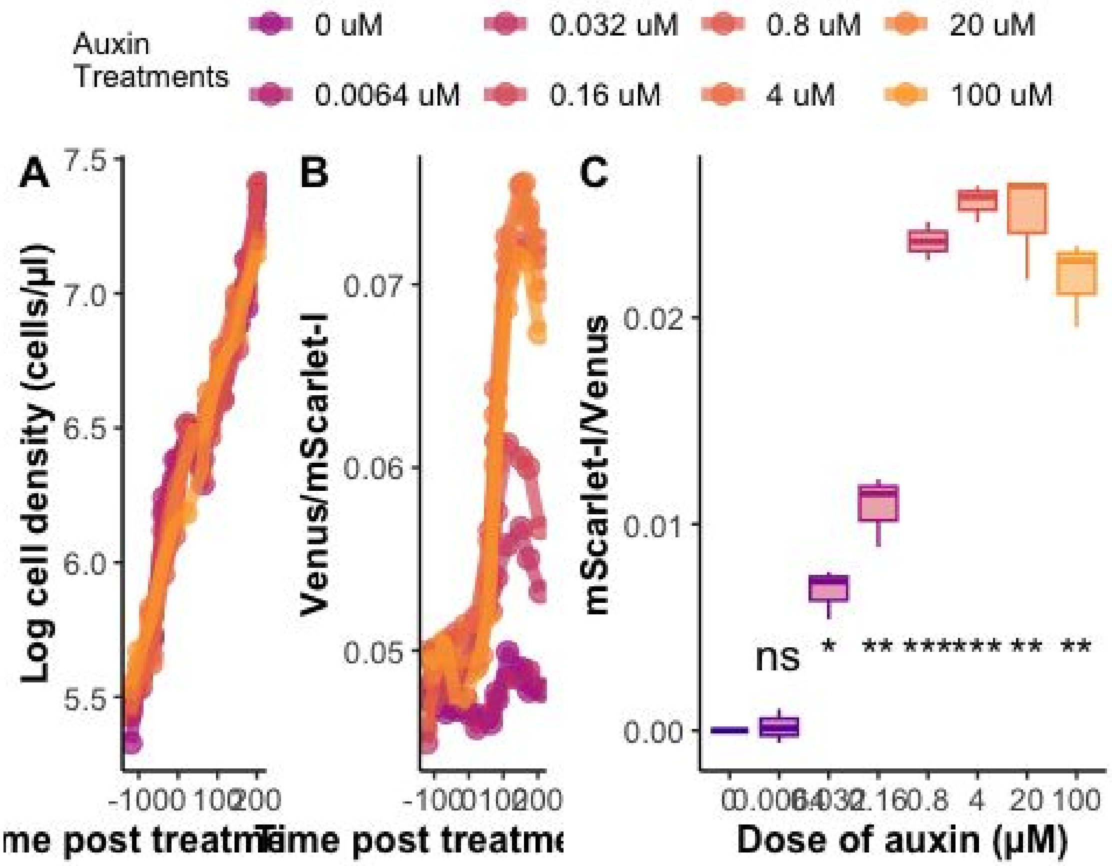


```
# ggsave('Supplement6_new.pdf',width = 12, height = 4)
```

Supporting Figure S6: The reversibility of the dual-fusion AFB2 biosensor

```
plate_05292021_TCA_washed <- read.plateSet(path = "Data for
publication/05292024-Biosensor_Reversible_TCA/All data/Washed/",
    pattern = "TCA*")

annotation <- createAnnotation(yourFlowSet = plate_05292021_TCA_washed)
write.csv(annotation, "Data for publication/05292024-
Biosensor_Reversible_TCA/05292024_TCA_washed_annotation.csv")

annotation <- read.csv("Data for publication/05292024-
Biosensor_Reversible_TCA/05292024_TCA_washed_annotation.csv")
aplate_05292021_TCA_washed <- annotateFlowSet(yourFlowSet =
plate_05292021_TCA_washed,
    annotation_df = annotation, mergeBy = "name")
head(rownames(pData(aplate_05292021_TCA_washed)))
# # [1] "1Pat-04292024-Biosensor_Reversibility-
TCA09_Experiment_yWL209_Orginial 50 uM Auxin.fcs"
# # [2] "1Pat-04292024-Biosensor_Reversibility-
TCA09_Experiment_yWL209_Orginial Control.fcs"
# # [3] "1Pat-04292024-Biosensor_Reversibility-
```

```
TCA09_Experiment_yWL209_Washed-Control-50uM Auxin.fcs"
# # [4] "1Pat-04292024-Biosensor_Reversibility-
TCA09_Experiment_yWL209_Washed-Control-Control.fcs"
# # [5] "1Pat-04292024-Biosensor_Reversibility-
TCA09_Experiment_yWL209_Washed-Orginial 50 uM Auxin-50uM Auxin.fcs"
# # [6] "1Pat-04292024-Biosensor_Reversibility-
TCA09_Experiment_yWL209_Washed-Orginial 50 uM Auxin-Control.fcs"
head(pData(aplate_05292021_TCA_washed))
# #
name
# # 1Pat-04292024-Biosensor_Reversibility-TCA09_Experiment_yWL209_Orginial 50
uM Auxin.fcs                                     1Pat-04292024-
Biosensor_Reversibility-TCA09_Experiment_yWL209_Orginial 50 uM Auxin.fcs
# # 1Pat-04292024-Biosensor_Reversibility-TCA09_Experiment_yWL209_Orginial
Control.fcs                                              1Pat-04292024-
Biosensor_Reversibility-TCA09_Experiment_yWL209_Orginial Control.fcs
# # 1Pat-04292024-Biosensor_Reversibility-TCA09_Experiment_yWL209_Washed-
Control-50uM Auxin.fcs                           1Pat-04292024-
Biosensor_Reversibility-TCA09_Experiment_yWL209_Washed-Control-50uM Auxin.fcs
# # 1Pat-04292024-Biosensor_Reversibility-TCA09_Experiment_yWL209_Washed-
Control-Control.fcs                                 1Pat-04292024-
Biosensor_Reversibility-TCA09_Experiment_yWL209_Washed-Control-Control.fcs
# # 1Pat-04292024-Biosensor_Reversibility-TCA09_Experiment_yWL209_Washed-
Orginial 50 uM Auxin-50uM Auxin.fcs 1Pat-04292024-Biosensor_Reversibility-
TCA09_Experiment_yWL209_Washed-Orginial 50 uM Auxin-50uM Auxin.fcs
# # 1Pat-04292024-Biosensor_Reversibility-TCA09_Experiment_yWL209_Washed-
Orginial 50 uM Auxin-Control.fcs       1Pat-04292024-Biosensor_Reversibility-
TCA09_Experiment_yWL209_Washed-Orginial 50 uM Auxin-Control.fcs
# #
folder
# # 1Pat-04292024-Biosensor_Reversibility-TCA09_Experiment_yWL209_Orginial 50
uM Auxin.fcs                         TCA09
# # 1Pat-04292024-Biosensor_Reversibility-TCA09_Experiment_yWL209_Orginial
Control.fcs                             TCA09
# # 1Pat-04292024-Biosensor_Reversibility-TCA09_Experiment_yWL209_Washed-
Control-50uM Auxin.fcs                   TCA09
# # 1Pat-04292024-Biosensor_Reversibility-TCA09_Experiment_yWL209_Washed-
Control-Control.fcs                      TCA09
# # 1Pat-04292024-Biosensor_Reversibility-TCA09_Experiment_yWL209_Washed-
Orginial 50 uM Auxin-50uM Auxin.fcs   TCA09
# # 1Pat-04292024-Biosensor_Reversibility-TCA09_Experiment_yWL209_Washed-
Orginial 50 uM Auxin-Control.fcs      TCA09
# #
X
# # 1Pat-04292024-Biosensor_Reversibility-TCA09_Experiment_yWL209_Orginial 50
uM Auxin.fcs                        1
# # 1Pat-04292024-Biosensor_Reversibility-TCA09_Experiment_yWL209_Orginial
Control.fcs                            2
# # 1Pat-04292024-Biosensor_Reversibility-TCA09_Experiment_yWL209_Washed-
Control-50uM Auxin.fcs                3
```

```
# # 1Pat-04292024-Biosensor_Reversibility-TCA09_Experiment_yWL209_Washed-Control-Control.fcs                    4
# # 1Pat-04292024-Biosensor_Reversibility-TCA09_Experiment_yWL209_Washed-Orginial 50 uM Auxin-50uM Auxin.fcs 5
# # 1Pat-04292024-Biosensor_Reversibility-TCA09_Experiment_yWL209_Washed-Orginial 50 uM Auxin-Control.fcs    6
# #
treatment
# # 1Pat-04292024-Biosensor_Reversibility-TCA09_Experiment_yWL209_Orginial 50 uM Auxin.fcs                   50 uM Auxin
# # 1Pat-04292024-Biosensor_Reversibility-TCA09_Experiment_yWL209_Orginial Control.fcs                           Control
# # 1Pat-04292024-Biosensor_Reversibility-TCA09_Experiment_yWL209_Washed-Control-50uM Auxin.fcs                  Control
# # 1Pat-04292024-Biosensor_Reversibility-TCA09_Experiment_yWL209_Washed-Control-Control.fcs                     Control
# # 1Pat-04292024-Biosensor_Reversibility-TCA09_Experiment_yWL209_Washed-Orginial 50 uM Auxin-50uM Auxin.fcs 50 uM Auxin
# # 1Pat-04292024-Biosensor_Reversibility-TCA09_Experiment_yWL209_Washed-Orginial 50 uM Auxin-Control.fcs    50 uM Auxin
# #
strain
# # 1Pat-04292024-Biosensor_Reversibility-TCA09_Experiment_yWL209_Orginial 50 uM Auxin.fcs                   yWL209
# # 1Pat-04292024-Biosensor_Reversibility-TCA09_Experiment_yWL209_Orginial Control.fcs                       yWL209
# # 1Pat-04292024-Biosensor_Reversibility-TCA09_Experiment_yWL209_Washed-Control-50uM Auxin.fcs              yWL209
# # 1Pat-04292024-Biosensor_Reversibility-TCA09_Experiment_yWL209_Washed-Control-Control.fcs                 yWL209
# # 1Pat-04292024-Biosensor_Reversibility-TCA09_Experiment_yWL209_Washed-Orginial 50 uM Auxin-50uM Auxin.fcs yWL209
# # 1Pat-04292024-Biosensor_Reversibility-TCA09_Experiment_yWL209_Washed-Orginial 50 uM Auxin-Control.fcs    yWL209
# #
reading
# # 1Pat-04292024-Biosensor_Reversibility-TCA09_Experiment_yWL209_Orginial 50 uM Auxin.fcs                         9
# # 1Pat-04292024-Biosensor_Reversibility-TCA09_Experiment_yWL209_Orginial Control.fcs                             9
# # 1Pat-04292024-Biosensor_Reversibility-TCA09_Experiment_yWL209_Washed-Control-50uM Auxin.fcs                    9
# # 1Pat-04292024-Biosensor_Reversibility-TCA09_Experiment_yWL209_Washed-Control-Control.fcs                       9
# # 1Pat-04292024-Biosensor_Reversibility-TCA09_Experiment_yWL209_Washed-Orginial 50 uM Auxin-50uM Auxin.fcs       9
# # 1Pat-04292024-Biosensor_Reversibility-TCA09_Experiment_yWL209_Washed-Orginial 50 uM Auxin-Control.fcs          9
# #
before_after
```

```
# # 1Pat-04292024-Biosensor_Reversibility-TCA09_Experiment_yWL209_Orginial 50
uM Auxin.fcs                         before
# # 1Pat-04292024-Biosensor_Reversibility-TCA09_Experiment_yWL209_Orginial
Control.fcs                                before
# # 1Pat-04292024-Biosensor_Reversibility-TCA09_Experiment_yWL209_Washed-
Control-50uM Auxin.fcs                    before
# # 1Pat-04292024-Biosensor_Reversibility-TCA09_Experiment_yWL209_Washed-
Control-Control.fcs                       before
# # 1Pat-04292024-Biosensor_Reversibility-TCA09_Experiment_yWL209_Washed-
Orginial 50 uM Auxin-50uM Auxin.fcs       before
# # 1Pat-04292024-Biosensor_Reversibility-TCA09_Experiment_yWL209_Washed-
Orginial 50 uM Auxin-Control.fcs          before
# #
sample
# # 1Pat-04292024-Biosensor_Reversibility-TCA09_Experiment_yWL209_Orginial 50
uM Auxin.fcs                           209-Original-Auxin
# # 1Pat-04292024-Biosensor_Reversibility-TCA09_Experiment_yWL209_Orginial
Control.fcs                                209-Original-Control
# # 1Pat-04292024-Biosensor_Reversibility-TCA09_Experiment_yWL209_Washed-
Control-50uM Auxin.fcs                209-Washed-Control-Auxin
# # 1Pat-04292024-Biosensor_Reversibility-TCA09_Experiment_yWL209_Washed-
Control-Control.fcs                 209-Washed-Control-Control
# # 1Pat-04292024-Biosensor_Reversibility-TCA09_Experiment_yWL209_Washed-
Orginial 50 uM Auxin-50uM Auxin.fcs     209-Washed-Auxin-Auxin
# # 1Pat-04292024-Biosensor_Reversibility-TCA09_Experiment_yWL209_Washed-
Orginial 50 uM Auxin-Control.fcs      209-Washed-Auxin-Control


plate_05292021_TCA_washed_sum <- summarizeFlow(aplate_05292021_TCA_washed,
channel = c("BL1.A",
    "YL1.A"), gated = TRUE)
# # [1] "Summarizing all events..."


time0_washed <- "2Pat-04292024-Biosensor_Reversibility-
TCA10_Experiment_yWL209_Orginial 50 uM Auxin.fcs"
# or whatever well was being read when auxin was added

plate_05292021_TCA_washed_sum$time <- plate_05292021_TCA_washed_sum$btime -
plate_05292021_TCA_washed_sum[[which(plate_05292021_TCA_washed_sum$name ==
    time0_washed), "btime"]]


plate_05292021_TCA_washed_sum <- plate_05292021_TCA_washed_sum %>%
    mutate(ratio_TCA_washed = BL1.Amean/YL1.Amean) %>%
    mutate(normalizedratio_ratio_TCA_washed =
ratio_TCA_washed/mean(ratio_TCA_washed))


plate_05292021_TCA_washed_sum <- summarizeFlow(aplate_05292021_TCA_washed,
```

```
channel = c("BL1.A",
    "YL1.A"), gated = TRUE)
# # [1] "Summarizing all events..."


time0_washed <- "2Pat-04292024-Biosensor_Reversibility-
TCA10_Experiment_yWL209_Orginial 50 uM Auxin.fcs"
# or whatever well was being read when auxin was added

plate_05292021_TCA_washed_sum$time <- plate_05292021_TCA_washed_sum$btime -
plate_05292021_TCA_washed_sum[[which(plate_05292021_TCA_washed_sum$name ==
    time0_washed), "btime"]]


plate_05292021_TCA_washed_sum <- plate_05292021_TCA_washed_sum %>%
    mutate(ratio_TCA_washed = BL1.Amean/YL1.Amean) %>%
    mutate(normalizedratio_ratio_TCA_washed =
ratio_TCA_washed/mean(ratio_TCA_washed))

afterwash_210 <- ggplot(subset(plate_05292021_TCA_washed_sum, strain ==
"yWL210" &
    reading %in% c("9", "10", "11", "12", "13", "14")), aes(x = time, y =
BL1.Amean/YL1.Amean,
    color = sample)) + geom_line(aes(color = sample), alpha = 0.7, size = 2)
+ geom_point(aes(color = sample),
    size = 3) + theme_classic() + ylim(7, 24) + theme(axis.title =
element_text(size = 12,
    face = "bold"), axis.text = element_text(size = 10), legend.title =
element_text(size = 10),
    legend.text = element_text(size = 10), legend.position = "none") +
xlab("Time post treatment (mins)") +
    ylab("VENUS/mScarlet") + scale_fill_manual(values = c("#cc6a70ff",
"#13306dff",
    "#f9b641ff", "#f68f46ff", "#6b4596ff", "#042333ff")) +
scale_color_manual(values = c("#cc6a70ff",
    "#13306dff", "#f9b641ff", "#f68f46ff", "#6b4596ff", "#042333ff"))
afterwash_210
```

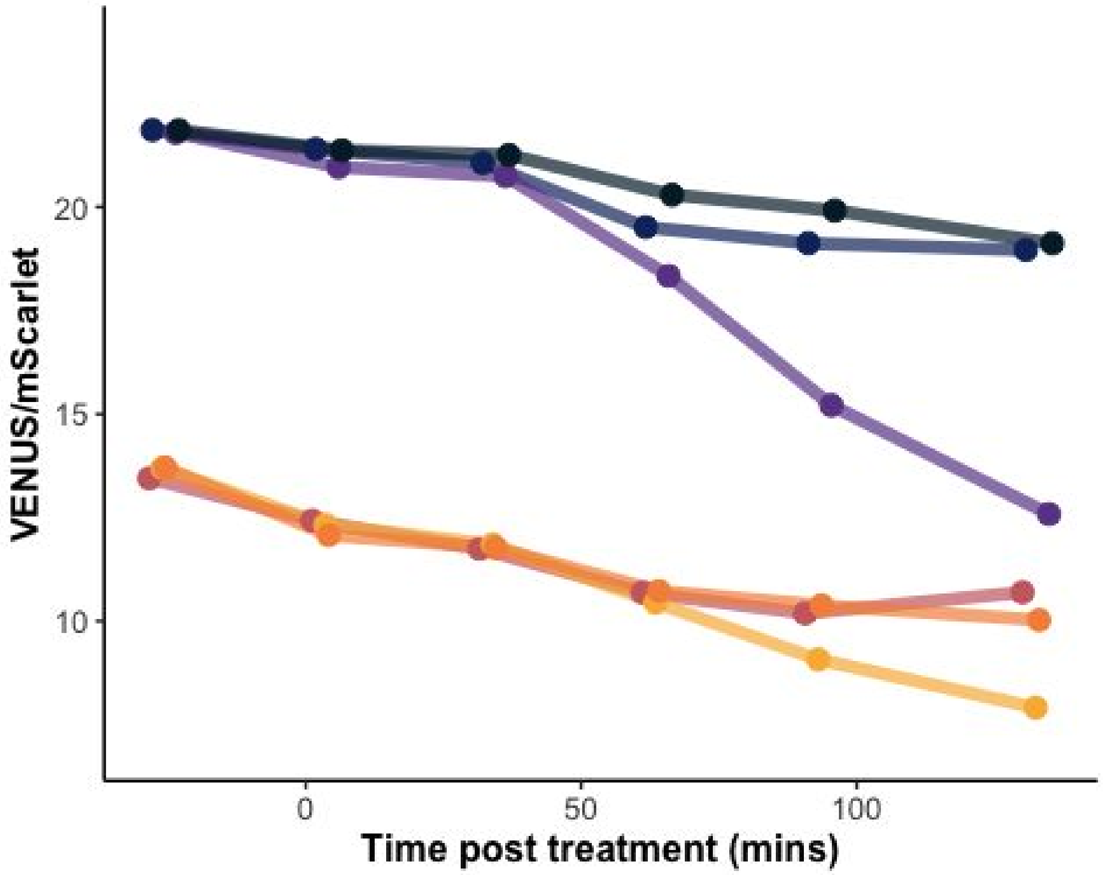


```
plot_washonly_210 <- plate_05292021_TCA_washed_sum %>%
    dplyr::filter(sample %in% c("210-Washed-Control-Auxin", "210-Washed-
Control-Control",
        "210-Washed-Auxin-Auxin", "210-Washed-Auxin-Control"))



washed_ratio <- plot_washonly_210 %>%
    dplyr::filter(reading %in% c("15", "16", "17")) %>%
    mutate(sample = case_when(sample == "210-Washed-Control-Auxin" ~ "Washed
Control treated, \nAuxin spike",
        sample == "210-Washed-Control-Control" ~ "Washed Control treated,
\nControl spike",
        sample == "210-Washed-Auxin-Auxin" ~ "Washed Auxin treated, \nAuxin
spike",
        sample == "210-Washed-Auxin-Control" ~ "Washed Auxin treated,
\nControl spike")) %>%
    ggplot(aes(x = sample, y = BL1.Amean/YL1.Amean, color = sample)) +
geom_point(aes(color = sample),
    alpha = 1, size = 3) + scale_color_manual(values = c("#f9b641ff",
"#f68f46ff",
```

```
    "#6b4596ff", "#042333ff")) + theme_classic() + ylab("Venus/mScarlet
Ratio") +
    xlab("") + theme(legend.position = "none") + stat_summary(fun.data =
mean_se,
    mult = 1, geom = "pointrange", color = "black")
washed_ratio
```

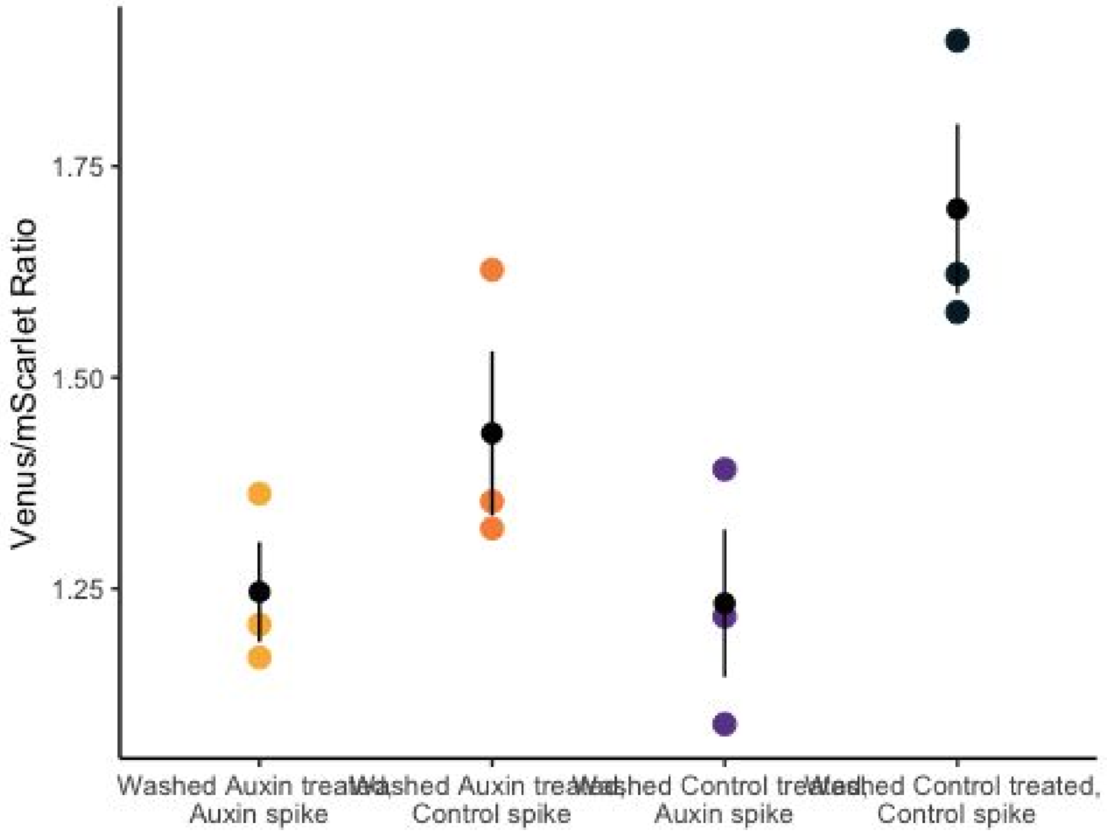


```
washed_Venus <- plot_washonly_210 %>%
    dplyr::filter(reading %in% c("15", "16", "17")) %>%
    mutate(sample = case_when(sample == "210-Washed-Control-Auxin" ~ "Washed
Control treated, \nAuxin spike",
        sample == "210-Washed-Control-Control" ~ "Washed Control treated,
\nControl spike",
        sample == "210-Washed-Auxin-Auxin" ~ "Washed Auxin treated, \nAuxin
spike",
        sample == "210-Washed-Auxin-Control" ~ "Washed Auxin treated,
\nControl spike")) %>%
    ggplot(aes(x = sample, y = BL1.Amean, color = sample)) + geom_point(alpha
= 1,
    size = 3) + scale_color_manual(values = c("#f9b641ff", "#f68f46ff",
"#6b4596ff",
```

```
    "#042333ff")) + theme_classic() + ylab("Venus") + xlab("") +
theme(legend.position = "none") +
    stat_summary(fun.data = mean_se, mult = 1, geom = "pointrange", color =
"black")
washed_Venus
```

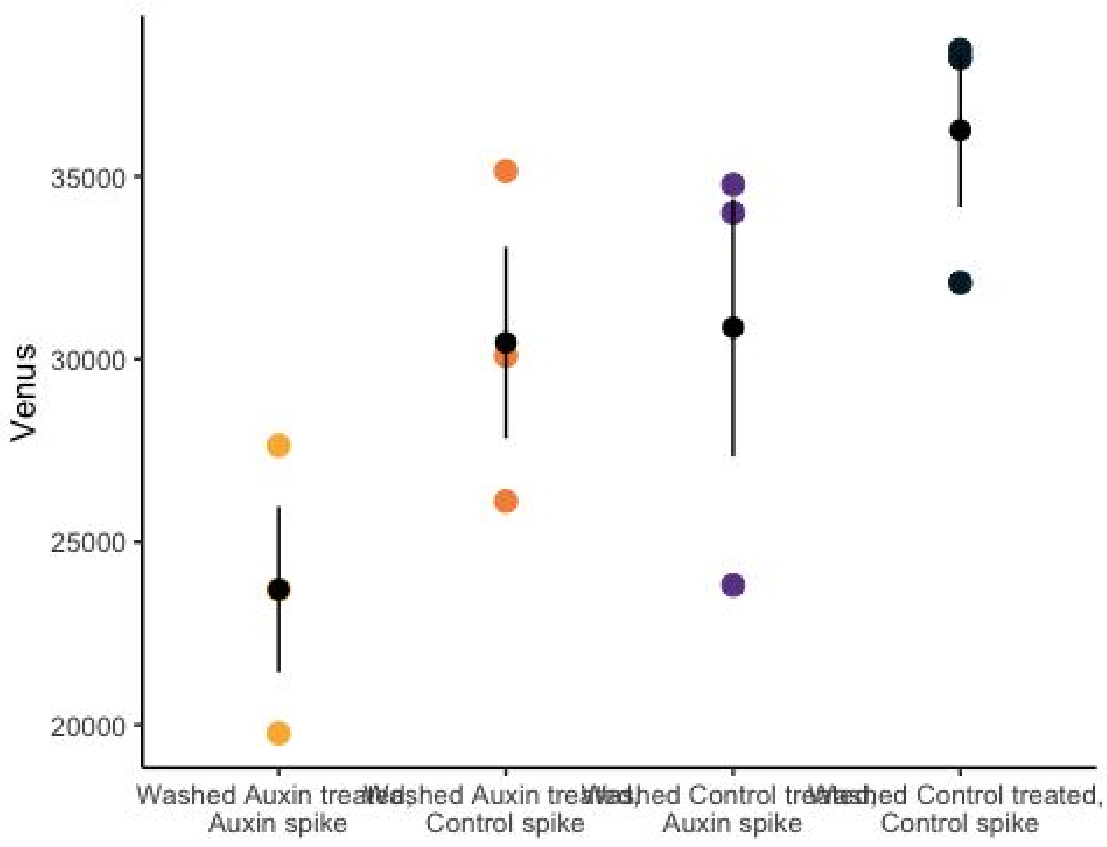


```
fig_ratio_Venus <- ggarrange(washed_ratio, washed_Venus, ncol = 1, nrow = 2,
labels = c("A",
    "B"))
fig_ratio_Venus
```

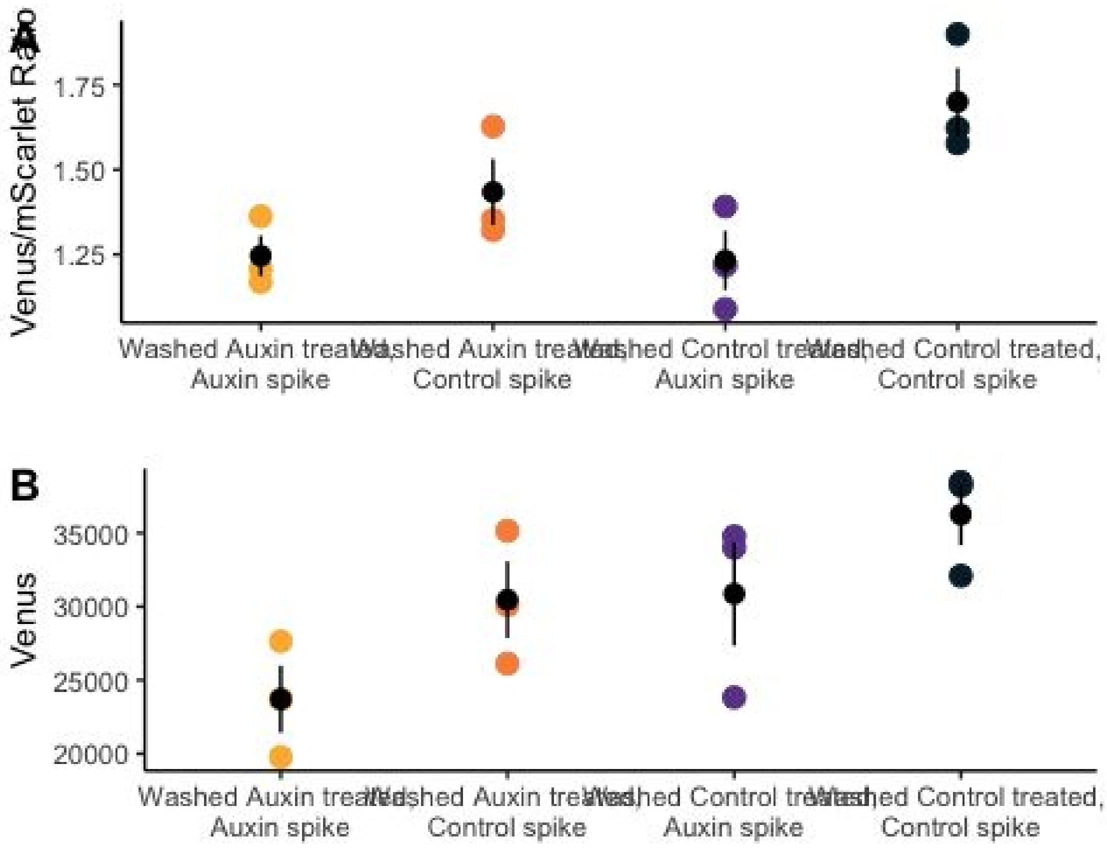


```r
# ggsave('fig_ratio_Venus.png', height = 5, width = 7)
```

Session Info and Packages/Libraries

```
```r
sessionInfo()
# # R version 4.3.2 (2023-10-31)
# # Platform: aarch64-apple-darwin20 (64-bit)
# # Running under: macOS Sonoma 14.5
# #
# # Matrix products: default
# # BLAS:   /Library/Frameworks/R.framework/Versions/4.3-
arm64/Resources/lib/libRblas.0.dylib
# # LAPACK: /Library/Frameworks/R.framework/Versions/4.3-
arm64/Resources/lib/libRlapack.dylib;  LAPACK version 3.11.0
# #
# # locale:
```
```

```
# # [1] en_US.UTF-8/en_US.UTF-8/en_US.UTF-8/C/en_US.UTF-8/en_US.UTF-8
# #
# # time zone: America/New_York
# # tzcode source: internal
# #
# # attached base packages:
# # [1] stats     graphics  grDevices utils     datasets  methods   base
# #
# # other attached packages:
# #  [1] flowClust_3.40.0     ggcyto_1.30.2        flowWorkspace_4.14.3
# #  [4] ncdfFlow_2.48.0      BH_1.84.0-0          openCyto_2.14.0
# #  [7] flowTime_1.26.0      flowCore_2.14.2      flextable_0.9.6
# # [10] ggsignif_0.6.4       multcompView_0.1-10  ggstance_0.3.7
# # [13] ggpubr_0.6.0         patchwork_1.2.0      wesanderson_0.3.7
# # [16] gridExtra_2.3        drc_3.0-1            MASS_7.3-60.0.1
# # [19] ggridges_0.5.6       lubridate_1.9.3      forcats_1.0.0
# # [22] stringr_1.5.1        dplyr_1.1.4          purrr_1.0.2
# # [25] readr_2.1.5          tidyr_1.3.1          tibble_3.2.1
# # [28] ggplot2_3.5.1        tidyverse_2.0.0
# #
# # loaded via a namespace (and not attached):
# #   [1] RColorBrewer_1.1-3      rstudioapi_0.15.0       jsonlite_1.8.8
# #   [4] magrittr_2.0.3          TH.data_1.1-2           farver_2.1.1
# #   [7] rmarkdown_2.26          zlibbioc_1.48.2         ragg_1.2.7
# #  [10] vctrs_0.6.5             askpass_1.2.0           rstatix_0.7.2
# #  [13] htmltools_0.5.8.1       plotrix_3.8-4           curl_5.2.1
# #  [16] broom_1.0.5             plyr_1.8.9              sandwich_3.1-0
# #  [19] zoo_1.8-12              uuid_1.2-0              mime_0.12
# #  [22] lifecycle_1.0.4         pkgconfig_2.0.3         Matrix_1.6-5
# #  [25] R6_2.5.1                fastmap_1.1.1           shiny_1.8.1.1
# #  [28] digest_0.6.34           colorspace_2.1-0        S4Vectors_0.40.2
# #  [31] textshaping_0.3.7       labeling_0.4.3          cytolib_2.14.1
# #  [34] fansi_1.0.6             timechange_0.3.0        abind_1.4-5
# #  [37] compiler_4.3.2          bit64_4.0.5             fontquiver_0.2.1
# #  [40] withr_3.0.0             backports_1.4.1         carData_3.0-5
# #  [43] highr_0.10              hexbin_1.28.3           openssl_2.1.1
# #  [46] gfonts_0.2.0            gtools_3.9.5            tools_4.3.2
# #  [49] zip_2.3.1               httpuv_1.6.15           glue_1.7.0
# #  [52] promises_1.3.0          grid_4.3.2              generics_0.1.3
# #  [55] gtable_0.3.4            tzdb_0.4.0              data.table_1.15.2
# #  [58] hms_1.1.3               xml2_1.3.6              car_3.1-2
# #  [61] utf8_1.2.4              BiocGenerics_0.48.1     pillar_1.9.0
# #  [64] vroom_1.6.5             later_1.3.2             splines_4.3.2
# #  [67] lattice_0.22-5          bit_4.0.5               survival_3.5-8
# #  [70] RProtoBufLib_2.14.1     tidyselect_1.2.0        RBGL_1.78.0
# #  [73] fontLiberation_0.1.0    knitr_1.45
fontBitstreamVera_0.1.1
# #  [76] crul_1.4.2              stats4_4.3.2            xfun_0.42
# #  [79] Biobase_2.62.0          matrixStats_1.3.0       stringi_1.8.3
# #  [82] yaml_2.3.8              evaluate_0.23           codetools_0.2-19
```

```
# #  [85] httpcode_0.3.0         officer_0.6.6          gdtools_0.3.7
# #  [88] Rgraphviz_2.46.0       graph_1.80.0           cli_3.6.2
# #  [91] xtable_1.8-4           systemfonts_1.0.6      munsell_0.5.0
# #  [94] Rcpp_1.0.12            XML_3.99-0.16.1        parallel_4.3.2
# #  [97] ggeasy_0.1.4           viridisLite_0.4.2      mvtnorm_1.2-5
# # [100] scales_1.3.0           crayon_1.5.2           rlang_1.1.3
# # [103] cowplot_1.1.3          multcomp_1.4-25        formatR_1.14
```
